# Supplementary material for: Critical success factors for high routine immunisation performance: a qualitative analysis of interviews and focus groups from Nepal, Senegal, and Zambia
Source: BMJ Open. 2023 Oct 4;13(10):e070541. doi: 10.1136/bmjopen-2022-070541 (PMC10551940; doi:10.1136/bmjopen-2022-070541)

Appendix 1: Coverage data for Nepal, Zambia, and Senegal

DHS DTP3 coverage in Zambia, by Province, 2000 – 2016

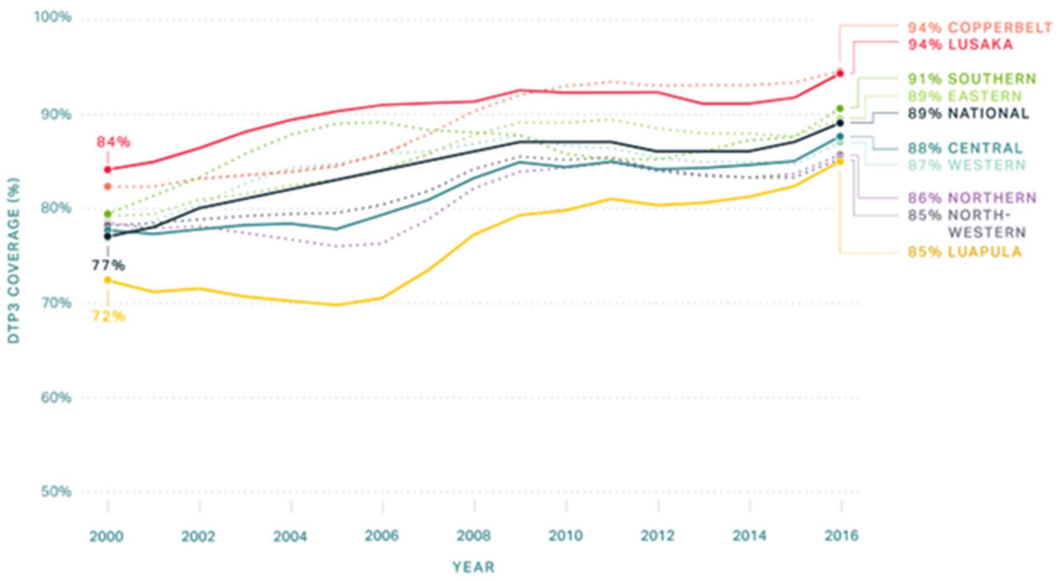

DHS DTP3 coverage in Nepal, by Province, 2000 – 2016

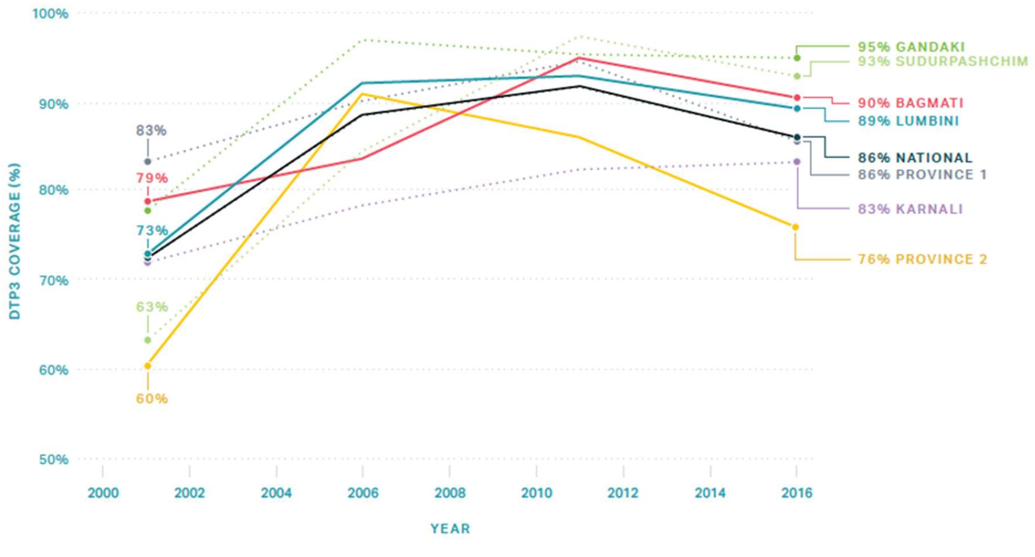

Data Source: Demographic and Health Survey (DHS)

DHS DTP3 coverage in Senegal, by Province, 2000 – 2016

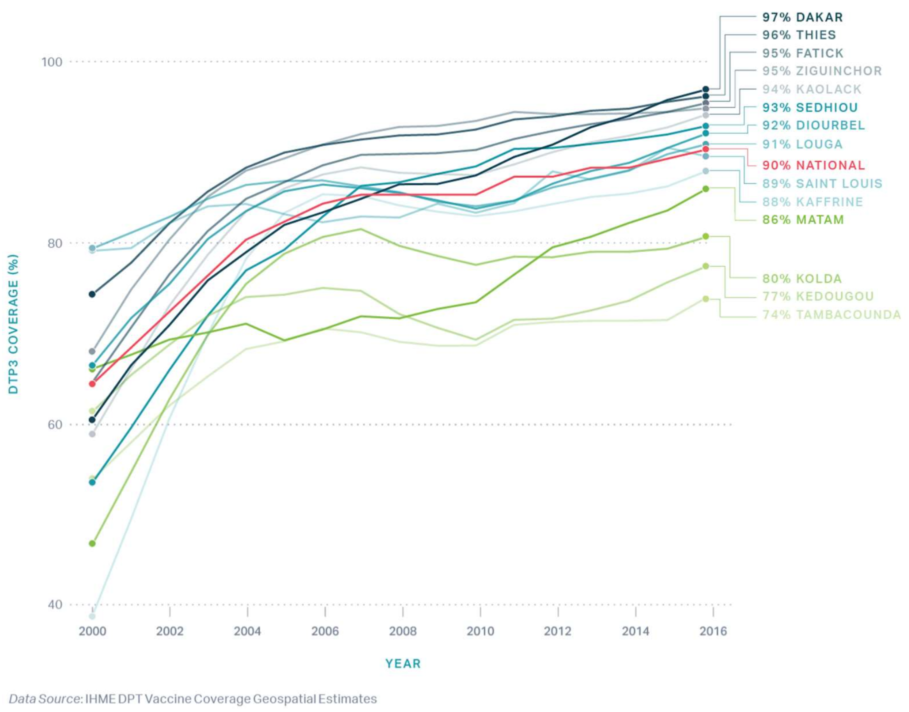

DTP3 coverage from 2000 to 2018 of Nepal, Senegal, and Zambia

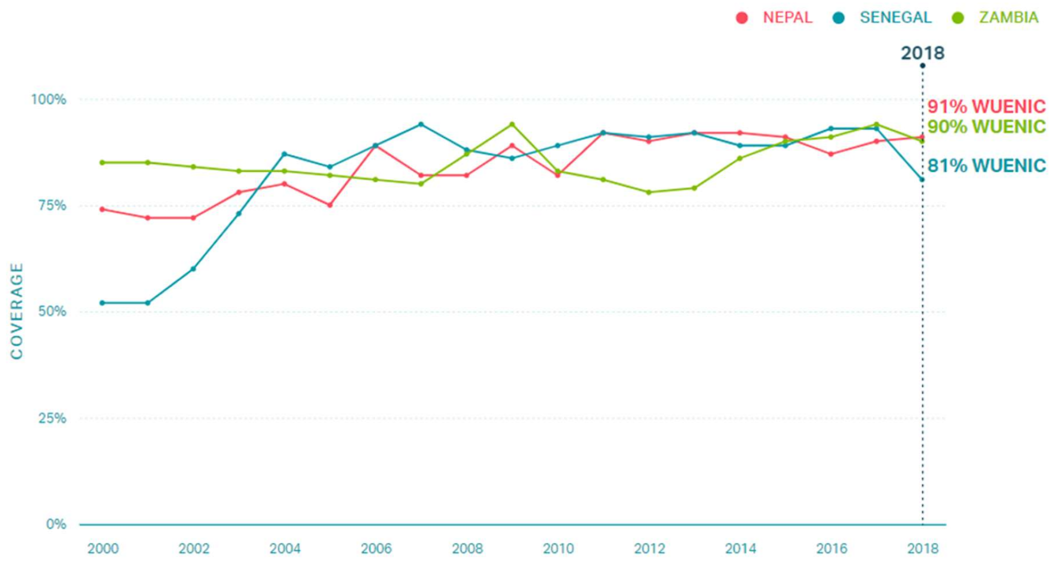

Supplement: Supplementary data [file bmjopen-2022-070541supp001.pdf]
