# Supplementary material for: Critical success factors for high routine immunisation performance: a qualitative analysis of interviews and focus groups from Nepal, Senegal, and Zambia
Source: BMJ Open. 2023 Oct 4;13(10):e070541. doi: 10.1136/bmjopen-2022-070541 (PMC10551940; doi:10.1136/bmjopen-2022-070541)
Supplement: Author's manuscript [file bmjopen-2022-070541.draft_revisions.pdf]

BMJ Open

BMJ Open is committed to open peer review. As part of this commitment we make the peer review history of every article we publish publicly available.

When an article is published we post the peer reviewers' comments and the authors' responses online. We also post the versions of the paper that were used during peer review. These are the versions that the peer review comments apply to.

The versions of the paper that follow are the versions that were submitted during the peer review process. They are not the versions of record or the final published versions. They should not be cited or distributed as the published version of this manuscript.

BMJ Open is an open access journal and the full, final, typeset and author-corrected version of record of the manuscript is available on our site with no access controls, subscription charges or pay-per-view fees (http://bmjopen.bmj.com).

If you have any questions on BMJ Open's open peer review process please email info.bmjopen@bmj.com

BMJ Open

Critical success factors for high routine immunization performance: A multiple case study analysis of Nepal, Senegal, and Zambia

Journal:	*BMJ Open*
Manuscript ID	bmjopen-2022-070541
Article Type:	Original research
Date Submitted by the Author:	23-Jan-2023

Complete List of Authors:	Sakas, Zoe; Rollins School of Public Health<br>Hester, Kyra; Emory University, Rollins School of Public Health<br>Ellis, Anna; Emory University, Rollins School of Public Health 1518 Clifton Rd Atlanta, GA, USA 30322<br>Ogutu, Emily; Emory University, Gangarosa Department of Environmental Health<br>Rodriguez, Katie; Emory University<br>Bednarczyk, Robert ; Emory University, Hubert Department of Global Health<br>Dixit, Sameer; Center for Molecular Dynamics Nepal<br>Kilembe, William; Emory University, Rwanda Zambia HIV Research Group<br>Sarr, Moussa; Institut de Recherche en Santé de Surveillance Epidémiologique et de Formations ,<br>Freeman, Matthew; Emory University, Gangarosa Department of Environmental Health

Keywords:	Paediatric infectious disease & immunisation < PAEDIATRICS, PUBLIC HEALTH, QUALITATIVE RESEARCH

SCHOLARONE™
Manuscripts

BMJ

I, the Submitting Author has the right to grant and does grant on behalf of all authors of the Work (as defined in the below author licence), an exclusive licence and/or a non-exclusive licence for contributions from authors who are: i) UK Crown employees; ii) where BMJ has agreed a CC-BY licence shall apply, and/or iii) in accordance with the terms applicable for US Federal Government officers or employees acting as part of their official duties; on a worldwide, perpetual, irrevocable, royalty-free basis to BMJ Publishing Group Ltd ("BMJ") its licensees and where the relevant Journal is co-owned by BMJ to the co-owners of the Journal, to publish the Work in this journal and any other BMJ products and to exploit all rights, as set out in our licence.

The Submitting Author accepts and understands that any supply made under these terms is made by BMJ to the Submitting Author unless you are acting as an employee on behalf of your employer or a postgraduate student of an affiliated institution which is paying any applicable article publishing charge ("APC") for Open Access articles. Where the Submitting Author wishes to make the Work available on an Open Access basis (and intends to pay the relevant APC), the terms of reuse of such Open Access shall be governed by a Creative Commons licence – details of these licences and which Creative Commons licence will apply to this Work are set out in our licence referred to above.

Other than as permitted in any relevant BMJ Author's Self Archiving Policies, I confirm this Work has not been accepted for publication elsewhere, is not being considered for publication elsewhere and does not duplicate material already published. I confirm all authors consent to publication of this Work and authorise the granting of this licence.

For peer review only

Title: Critical success factors for high routine immunization performance: A multiple case study analysis of Nepal, Senegal, and Zambia

Authors: Zoe Sakas MPH[a], Kyra A. Hester MPH[a], Anna S. Ellis MDP[a], Emily Awino Ogutu MPH[a], Katie Rodriguez MPH[a], Robert A. Bednarczyk PhD[a], Sameer Dixit PhD[b], William Kilembe MD[c], Moussa Sarr MD[d], Matthew C. Freeman PhD[a*]

* Corresponding author:
Matthew C. Freeman, PhD
matthew.freeman@emory.edu

Author affiliations:
a Rollins School of Public Health, Emory University, Georgia, USA
b Center for Molecular Dynamics Nepal, Kathmandu, Nepal
c Center for Family Health Research in Zambia, Lusaka, Zambia
d Institut de Recherche en Santé de Surveillance Epidemiologique et de Formation, Dakar, Senegal

Authors' contributions: KAH, ASE, SD, WK, MS, RAB, MCF: project conceptualization and methodology; KAH, ZS, ASE, KR, SD, WK, MS, RAB, MCF: investigation and data curation; KAH, ZS, ASE, KR, EAO, RAB, MCF: formal analysis; ZS, KAH: writing - original; KAH, ZS, ASE, EAO, KR, SD, WK, MS, RAB, MCF: writing – review and editing; all authors provided approval of the final version.

Abstract (291 words):
INTRODUCTION: Vaccination averts an estimated 2-3 million deaths annually. Although vaccine coverage improvements across Africa and South Asia have remained relatively stagnant and below global targets, several countries have outperformed their peers with significant increases in routine immunization coverage. Examining these countries' vaccination programs provides an opportunity to identify and describe critical success factors that may have supported these improvements.

METHODS: We selected three high-performing countries in regard to childhood vaccination: Nepal, Senegal, and Zambia. This multiple case study analysis was conducted using data from the Exemplars in Vaccine Delivery project within the Exemplars in Global Health program. We used qualitative analysis to investigate factors that contributed to high vaccination coverage through key informant interviews (KIIs) and focus group discussions (FGDs) at the national, regional, district, health facility, and community levels. We triangulated these findings with quantitative analyses using publicly available data, which are published elsewhere.

RESULTS: Our data revealed that the critical success factors for vaccine programming relied on the cultural, historical, and statutory context in which the interventions were delivered. In Nepal, Senegal, and Zambia, high immunization coverage was driven by 1) strong governance structures and healthy policy environments; 2) adjacent successes in health systems strengthening; 3) government-led community engagement initiatives; and 4) adaptation considering contextual factors at all levels of the health system.

CONCLUSION: Throughout the study, our analysis returned to the importance of defining and understanding the context, governance, financing, and health systems within a country, rather than focusing on any one intervention. This paper augments findings from existing literature by highlighting how contextual factors impact implementation decisions that have led to improvements in childhood vaccine delivery. Findings from this research may identify transferable lessons and support actionable recommendations to improve national immunization coverage in other settings.

For peer review only - http://bmjopen.bmj.com/site/about/guidelines.xhtml

Key Messages:

What is already known on this topic:

Immunization is a cost effective and life-saving public health intervention. The essential components of an effective vaccine delivery system are well-established, along with the behaviors related to routine immunization.

What this study adds:

This study highlights how structural and contextual factors impacted the implementation of childhood vaccination programs in several countries with high vaccination coverage. By applying a positive deviant approach, we identify and describe drivers of immunization coverage that programmers and policy makers may utilize to better understand underlying factors within the system.

How this study might affect research, practice, or policy:

Through focusing on countries with high routine immunization coverage, we examined how vaccine delivery systems may leverage components of existing governance structures and health systems to accelerate and sustain coverage. Operational definitions for governance, health systems strengthening, community engagement, and adaptive capacity, along with descriptions of how these processes were implemented in high-performing countries, may help other countries implement similar improvements.

For peer review only - http://bmjopen.bmj.com/site/about/guidelines.xhtml

1. Introduction

Vaccination is recognized as one of the most influential public health interventions, averting an estimated 2-3 million deaths annually [1-4]. The Global Vaccine Action Plan (GVAP) targeted at least 90% country-level coverage of the third dose of diphtheria, tetanus, pertussis vaccine (DTP3) among 1-year-old children [5], a globally recognized proxy for vaccination system performance [6]. However, by 2018 only 95 of the 193 World Health Organization (WHO) Member States achieved national GVAP targets, and less than one third met district level targets of 80% DTP3 [7-9].

In 2019, the African Region reported the lowest DTP3 coverage at 74%, a slight improvement from 71% coverage in 2010 [8]. DTP3 coverage in the South-East Asia Region substantially improved by 2019, to 91%; however, from 2000 to 2016, this region reported one of the lowest coverage rates with an average of 78% [8]. Within both regions, several countries outperformed their peers with significant increases in routine immunization coverage since 2000 [10]. Examining these countries' vaccination programs provides an opportunity to identify and describe critical success factors that may have supported these improvements.

The essential components of an effective vaccine delivery system are well-established and include strong governance and leadership, healthcare financing, human resources, a robust supply chain, community engagement, and information systems for evidence-based decision-making [11-13]. Research on behaviors related to routine immunization focuses on recognized determinants of coverage, including intent to vaccinate, community access, and health facility readiness [14]. This paper augments findings from existing literature by highlighting how structural and contextual factors impact implementation decisions that have led to improvements in childhood vaccine delivery.

Our research describes how countries with high vaccination rates took similar paths to success [15-18]. Through investigating drivers of high routine immunization coverage, our findings revealed the importance of structural factors, including governance processes and collaboration between in-country stakeholders, which more broadly align with requirements for strong national health systems. Global DTP3 coverage has plateaued since 2010 at 86% [19]; identifying factors and mechanisms for accelerating progress is critical for expanding vaccination program reach and retention [9].The purpose of this study was to identify critical success factors that contributed to catalytic growth in childhood routine immunization coverage. Findings from this research may identify transferable lessons and support actionable recommendations to improve national immunization coverage in other settings [20].

For peer review only - http://bmjopen.bmj.com/site/about/guidelines.xhtml

2. Methods

This multiple case study analysis was conducted using data from the Exemplars in Vaccine Delivery project within the Exemplars in Global Health program [10, 20, 21]. We used qualitative analysis to investigate factors that contributed to high vaccination coverage through key informant interviews (KIIs) and focus group discussions (FGDs) at the national, regional, district, health facility, and community levels. We triangulated these findings with quantitative analyses using publicly available data, which are published elsewhere and referenced throughout this paper [10, 21].

Prior to data collection, we developed a conceptual model (Figure 1) to organize factors that impact childhood vaccine coverage globally. This model was based on the work of Phillips et al. and LaFond et al. alongside a broader, a priori, review of the vaccine literature [14, 22].

Figure 1. A priori conceptual model of the drivers of vaccine coverage

2.1. Study sites

Methods for country, region, and district selection are detailed elsewhere [10, 15-17]. Briefly, countries were selected based on DTP1 and DTP3 coverage estimates, which served as proxies of the vaccine delivery system, with DTP1 as a proxy of access and DTP3 as a proxy of continued utilization of immunization services (Appendix 1) [10, 23, 24]. Three regions within each country were identified in consultation with national stakeholders and available data (Table 1). Partner organizations and Ministry of Health (MoH)[1] officials facilitated site selection and data collection activities.

2.2. Qualitative data collection and analysis

Qualitative data were collected between October 2019 and April 2021 at the national, regional, district, health facility, and community levels in Nepal, Senegal, and Zambia (Table 1). The interview guides were informed by the Consolidated Framework for Implementation Research (CFIR) [25] and the Context and Implementation of Complex Interventions (CICI) framework [26]. KII and FGD guides were translated into local languages by research assistants from the respective countries. All interview guides were piloted before use and adjusted iteratively throughout data collection. An initial list of KIIs was developed with local research partners and MoH officials with snowball sampling used to identify additional key informants. Our sampling approach aimed to include a diverse sample of participants in regard to geographic location and demographic qualities. Caregivers and volunteer community health workers were recruited for FGDs from health facility catchment areas with the assistance of local health staff. The duration of KIIs and FGDs averaged one and a half hours. KIIs and FGDs were audio-recorded with the permission of participants. Research files, recordings, and transcriptions were de-identified and password protected. We applied a theory-informed thematic analysis of the transcripts to identify critical success factors. Transcripts were coded and analyzed using MaxQDA2020 software (Berlin, Germany). We considered setting and participant roles while identifying key points and further contextualized data using historical documents and an a priori literature review. Qualitative data collection and analysis tools, including topic guides and codebooks, can be found on our Open Sciences Framework (OSF) page [27].

---

[1] *Note that each country has a slightly different name for their Ministry of Health (e.g., Ministry of Health and Population in Nepal; Ministry of Health and Social Action in Senegal), but all will be referred to as "MoH" throughout for simplicity.*

For peer review only - http://bmjopen.bmj.com/site/about/guidelines.xhtml

For peer review only

For peer review only - http://bmjopen.bmj.com/site/about/guidelines.xhtml

Table 1: Summary of countries, regions, districts selected for research, and data collection activities

Country	Nepal		Senegal		Zambia	
In Country Research Partner	Center for Molecular Dynamics, Nepal		Institut de Recherche en Santé de Surveillance, de Surveillance Epidémiologique et de Formation (IRESSEF)		Center for Family Health Research in Zambia	
Data collection period (MM/YYYY)	8/2019 – 12/2019		12/2020 - 4/2021		10/2019 – 02/2020	
**Regions and Districts**						
Region 1	Madhes*	Dhanusha, Bara, Mahottari	Ziguinchor	Ziguinchor, Oussouye, Diouloulou	Lusaka	Lusaka, Rufunsa, Chongwe
Region 2	Bagmati	Makwanpur, Dolakha, Kathmandu	Dakar	Rufisque, Mbao, Keur Massar	Central	Chibombo, Chitambo, Serenje
Region 3	Gandaki Pradesh	Kaski, Myagdi, Nawalparasi	Tambacounda	Tambacounda, Koumpentoum, Goudiry	Luapula	Chipili, Nchelenge, Samfya
**Key Informant Interviews** *Key: Number of KIIs (Participants)*	**79 (79)**		**62 (63)**		**66 (85)**	
National level government staff	11 (11)		5 (5)		11 (12)	
Partner organization staff	8 (8)		4 (4)		11 (15)	
Regional health staff	5 (5)		7 (7)		6 (8)	
District health staff	15 (15)		38 (38)		10 (19)	
Health facility staff	23 (23)		6 (6)		7 (10)	
Community leaders	15 (15)		2 (2)		10 (10)	
Community health workers **	2 (2)		-		11 (11)	
**Focus Group Discussions** *Key: Number of FGDs (Participants)*	**30 (191)**		**19 (128)**		**22 (132)**	
Community health workers **	9 (60)		10 (65)		10 (60)	
Mothers	9 (60)		9 (63)		8 (48)	
Fathers	6 (36)		-		1 (6)	
Grandparents	6 (35)		-		3 (18)	
**Total (per country)**	**109 (270)**		**81 (191)**		**88 (217)**	
**Total (across countries)**	-		-		**278 (678)**	

** Madhes was referred to as Province 2 prior to 2022 (including during data collection)*
*** Includes volunteer community health workers, female community health volunteers (FCHV) – Nepal; vaccinators – Nepal; bajenu gox – Senegal; and neighborhood health committee members – Zambia.*

For peer review only - http://bmjopen.bmj.com/site/about/guidelines.xhtml

2.3. Supplemental quantitative data analysis

Quantitative data were collected through secondary datasets, which included information from the MOH in Zambia, Nepal, and Senegal among other partners. Data were used to estimate routine immunization coverage from 2000 to 2019 and to uncover trends related to catalytic improvements. Additional analyses were conducted to identify indicators that may be associated with immunization coverage success among low- and lower-middle-income countries using regression models to statistically test financial, development, demographic, and other country-level indicators. These quantitative analyses were published elsewhere. Findings from the quantitative analyses provided additional context for the qualitative findings which informed the results presented here.

2.4. Ethical approval

This study was considered exempt by the Institutional Review Board committee of Emory University, Atlanta, Georgia, USA (IRB00111474); approved by the Nepal Health Research Council (NHRC; Reg. no. 347/2019) in Kathmandu, Nepal; the National Ethical Committee for Health Research (CERNS; Comité National d'Ethique pour la Recherche en Santé) in Dakar, Senegal (00000174); the University of Zambia Biomedical Research Ethics Committee (Federal Assurance No. FWA00000338, REF. No. 166-2019); and the National Health Research Authority in Zambia. All ethics committees abide by the principles of the Declaration of Helsinki; all participants provided written consent.

2.5. Public involvement

The study team ensured the involvement of stakeholders, researchers, and policy makers in the global immunization sector during the design, implementation, and dissemination of this project. We utilized our Technical Advisory Group (TAG) to develop an initial set of questions for scoping visits to our exemplar countries, wherein study members met with in-country partners and experts to further refine the direction and nature of the questions. Scoping visit findings were used to create the final data collection tools [10]. As this is a hypothesis generating study, and no intervention was provided, data collection was targeted to conform to general standards of burden. We adhered to standard best-practices for qualitative research, iteratively implementing feedback from experts and the TAG. While there was community involvement (FGDs), the primary focus of data collection was centered on historical stakeholders who played key roles in the immunization program [10]. Upon completion of each country case study, findings were disseminated to in-country experts and country governments, along with the TAG, allowing for the study team to ground-truth results. Reports, and manuscripts, of findings were sent to those involved.

2.6 Sex and Gender Considerations

This study was hypothesis generating, and did not provide an intervention; regardless, the research team took gender differences into consideration. For our FGDs, we worked with groups of both mothers and fathers; fathers were included as to not fall into the bias of children's health being a 'woman's issue'. FGDs were separated by gender to allow participants to feel comfortable sharing their thoughts.

Due to the historical nature of this study, our KII sample of key stakeholders and leaders leaned male due to prior gender biases and the tendency to place men in positions of importance or power. However, present-day positions were often filled by women, and we sampled women for KIIs as appropriate.

For peer review only - http://bmjopen.bmj.com/site/about/guidelines.xhtml

3. Results

3.1. Drivers of successful vaccination programs in exemplar countries

Following analysis of data from Nepal, Zambia, and Senegal, we revisited the conceptual framework developed during study conception (Figure 1). Although existing literature elaborates on factors related to intent to vaccinate, facility readiness, and community access, there is a gap in knowledge related to *how* policies and programs are operationalized to address these determinants – which relies heavily on the existing governance and health systems, engagement of community members (or end-users), and ability to adapt to the context to improve implementation. Our empirical data from Nepal, Senegal, and Zambia revealed that the critical success factors could be best understood by detailing the cultural, historical, and statutory context in which the interventions were delivered. This is in contrast to any specific modification or adaptation of key interventions or policies. We revised the initial conceptual framework based on findings from empirical data to illustrate a greater focus on these emergent factors (Figure 2). Definitions for the key domains can be found in Table 2.

Figure 2. Revised conceptual framework for drivers of vaccine coverage

Table 2: Definitions for drivers of vaccine coverage

Domain	Definition
**Governance and financing** [a]	The processes that guide policies and operations within the health system, including collaboration (effective working relationships, including alignment of priorities), communication (open and frequent channels for sharing knowledge and feedback), and commitment (dedication to activities and responsibilities).
**Health systems strengthening** [a]	Strategies that are designed to sustainably improve health system performance (across all sectors) through an integrated and horizontal approach, with a focus on equity.
**Community engagement** [a]	Responsibility of the community to implement vaccination program activities through local leadership and volunteers, fostered by government initiatives and cultural values.
**Adaptive capacity** [a]	Capability to sufficiently consider contextual factors, supported by timely evaluation of quality data, to tailor vaccine programming based on local needs and priorities – and improve implementation quality.
**Intent to vaccinate** [b]	Demand for childhood vaccines on the part of the caregiver or guardian that would result in vaccination in the absence of other barriers.
**Facility readiness** [b]	Supply (by the health system) of health and vaccination services to adequately meet demand. Incorporates supplies (vials, syringes etc.), human resources, and the consistency of their availability.
**Community access** [b]	The ability (or inability) to successfully carry out the transaction of vaccine utilization, i.e., barriers and facilitators between Intent and Readiness.

a. *Developed from our research, both via data analysis and desk review of existing literature*
b. *See Figure 1 (and reference Phillips et al. paper)*

For peer review only - http://bmjopen.bmj.com/site/about/guidelines.xhtml

3.2. Operationalization of successful vaccine delivery programs

In Nepal, Senegal, and Zambia, high immunization coverage was driven by 1) strong governance structures and healthy policy environments; 2) adjacent successes in health systems strengthening; 3) government-led community engagement initiatives; and 4) adaptation considering contextual factors at all levels of the health system. Below, we summarize similarities apparent in all three high-performing countries. Appendix 2 includes informative quotes as they relate to the four domains. More information on how policies and programs were implemented and adapted to local context is available elsewhere; quotes from each country and domain can be found in Appendix 2 [15-17].

Strong governance structures and a healthy policy environment

Effective collaboration, open communication, and commitment to health and vaccines supported policy development and implementation of the vaccination programs in Nepal, Senegal, and Zambia.

Collaboration and communication enabled alignment of priorities, division of responsibilities, and coordination between levels. In all three countries, collaboration was apparent between external partners and the government, and between different levels of the health system. National fora were established for frequent and multi-disciplinary communication, including Inter-Agency Coordinating Committees (ICC) [28]. Regional, district, and community level reflection meetings were held to share and review data and gather feedback for strategic planning. Formal channels for supportive supervision and feedback, both top-down and bottom-up, supported service delivery improvements and reporting.

In all three countries, vaccination was a "priority" or "flagship" program recognized by the MOH. Prioritization of vaccination within the health sector supported sufficient resource allocation. In Nepal and Senegal, dedication to the health sector more broadly was codified through defining health as a right in their national constitutions. Commitment to health and vaccines underpinned policy development and enabled consistent service delivery. Community members could expect reliable and equitable access to vaccines due to the prioritization of activities by health personnel and the dedication of volunteer community health workers who supported outreach activities and spearheaded demand generation and educational campaigns.

> *"A provision was made in the constitution, and [vaccines] became a fundamental right, so the government has a responsibility to provide. And management of currently running programs also [focuses on] children who have missed vaccines." (Nepal Health Research Council Staff, Nepal)*

Adjacent successes in health systems strengthening

Improvements in the broader health systems of Nepal, Senegal, and Zambia – including health post expansion, training of volunteer community health workers, general capacity building, and prioritization of equitable health services – supported high immunization coverage.

Health post expansion and capacity building at subnational and community levels were mentioned by most key informants in all three countries as essential to improvements in vaccination coverage. Building health facilities in rural areas especially allowed parents to easily vaccinate their children, without having to travel far distances or rely on outreach. Improvements in vaccine service delivery also relied on improvements in general infrastructure including roads, electricity, and connectivity required for transportation, health facility operations, and data reporting. In all three countries, general health systems strengthening was centered around equity as a priority for decision making. Health post expansion and community health worker programs were strengthened to prioritize service delivery in hard-to-reach communities, minority populations, and remote areas.

For peer review only - http://bmjopen.bmj.com/site/about/guidelines.xhtml

> *"Previously, there was no public health department at a grassroots level, it was just at the National level... So, now the Public Health Specialist is on the ground to try to see that this primary health care activity is promoted. And I think with that supervision, with the introduction of the public health nurses, Health promotion departments, I think these interventions couldn't be achieved higher in terms of immunization activities." (District Health Director, Zambia)*

Ongoing efforts to address equity concerns include: 1) Overcoming geographical barriers through health post expansion and outreach services supported by national-level policy and external partner funding; and 2) Addressing social, economic, and cultural factors through tailored messaging and local ownership of vaccination programming.

Integrated decision making across health and social sectors, through a horizonal rather than vertical approach, facilitated effective allocation of resources (e.g., monetary, human, technical, educational) could be efficiently allocated across programs. For example, national fora and committees in all three countries included representatives from the immunization sector, maternal and child health, education and development, and financial offices. During meetings, sectors would align long-term priorities and discuss how to reach overall goals in terms of health and social outcomes.

Government-led community engagement initiatives

Community ownership of vaccination activities and corresponding health outcomes was apparent in Nepal, Senegal, and Zambia, and even more so in high-performing districts within these countries. Responsibility of the community to implement vaccination programming through local leadership and volunteers was fostered by government initiatives and cultural values. National policies focused on involvement of traditional leaders and all national-level stakeholders reported on the importance of community actors. In some communities, female volunteers would work beyond their mandates to ensure all children were vaccinated – a decision motivated by dedication to their communities at large, empathy for the children in their neighborhoods, and a vague understanding of herd immunity.

Key informants from the national to community level reported that demand for vaccines was critical for improving routine immunization coverage. In addition to the work of volunteer community health workers, demand generation and public awareness were accomplished through national policies, media engagement, involvement of community actors, and school outreach activities.

> *"Community health workers play a very important role – they are in direct contact with the population. They are the ones who live in the community, and they identify more with the community. So, there is a relationship of trust between them and the population." (National level government stakeholder, Senegal)*

Further analysis of community health worker programs and demand generation activities in Nepal, Senegal, and Zambia will be detailed in our upcoming publications [29].

Adaptation at all levels of the health system

In Nepal, Senegal, and Zambia, decision-makers and implementers at the national, subnational, and community levels demonstrated their ability to consider contextual factors, evaluate or reflect on quality data, and tailor vaccine programming based on local needs and priorities.

Quality data were available for evidence-based decision making in all three countries. Data software was implemented at the district level in all countries - or in the case of Senegal, at the health post level - allowing stakeholders at all levels to utilize accurate and up-to-date coverage estimates. Lack of quality

For peer review only - http://bmjopen.bmj.com/site/about/guidelines.xhtml

data in some areas may have hindered this process. However, when data were not available, community actors would adapt programming based on head counts conducted by volunteers and an anecdotal understanding of the contextual and cultural needs in their communities.

> *"We have been advising facilities that they need to own the data; they have to make sure that they utilize it by analyzing… If they are not meeting their targets, they need to reflect on the data and see where they are going wrong… For those [health facilities] that are performing well, we find out what they are doing, and we try to replicate to the other facilities that are not doing as well." (District Health Officer, Zambia)*

Adapting strategies to respond to context-specific needs was essential for reaching children. In all three countries, flexible vaccine delivery days and outreach services supported unique scheduling needs (e.g., in Senegal, vaccinating children in the late afternoon during farming season). Outreach strategies were also tailored to accommodate contextual factors and address barriers to access. Understanding cultural and social values enabled community actors to tailor demand generation activities so that community members would be more apt to adopt positive health behaviors. Some examples of this may be using a local language, involving religious or community leaders in engagement and sensitizing, or addressing cultural or social barriers directly through personal communication.

For peer review only

For peer review only - http://bmjopen.bmj.com/site/about/guidelines.xhtml

3.3. Interventions and programs across high-performing countries

Key informants discussed a myriad of interventions and programs that they believed led to the improved and sustained vaccination coverage in their countries (Table 3). Interventions are organized by implementation level; if an intervention was implemented at multiple levels of the health system, it may be repeated in the table. Although the specific components of vaccine programming were not identical across countries, many of the general themes overlapped (e.g., the scope of community-level health committees varied between countries, but the purpose/objective of these groups was similar).

Table 3: Key interventions and programs implemented to improve routine immunization coverage in Nepal, Senegal, and Zambia from 2000 to 2019, based on KII and FGD data

	**Key for reading the table below:**
[red]	Not mentioned by key informants or community stakeholders who participated in this study
[green]	Implemented; participants noted that there was room for improvement (e.g., gaps or challenges)
[blue]	Implemented; participants described successful implementation (e.g., exceeds recommended guidelines)

Category	Policy or intervention	Zambia	Nepal	Senegal
**National level programming**<br>*Includes external partners and government*	Introduction of new vaccines	[blue]	[blue]	[blue]
	Vaccination as "priority" program	[green]	[blue]	[green]
	Strategic reallocation of targeted funds	[blue]	[blue]	[red]
	Health post expansion	[green]	[green]	[green]
	Constitution codifying health as a right	[red]	[blue]	[green]
	Forums for decision-making (e.g., ICC)	[blue]	[green]	[green]
	Media engagement	[blue]	[blue]	[blue]
**Sub-national programming**<br>*Regional and district level*	RED/REC implementation	[blue]	[green]	[blue]
	Training community health workers	[green]	[green]	[green]
	Meetings for evidence-based decisions	[green]	[green]	[blue]
	Data software, health post level	[red]	[red]	[green]
	Data software, district level	[green]	[green]	[green]
	Cold chain expansion	[blue]	[green]	[green]
**Community programming**<br>*Health post and village/community level*	Community health worker program	[blue]	[blue]	[blue]
	Community-level health committees	[blue]	[green]	[blue]
	Outreach services	[blue]	[blue]	[blue]
	Microplanning in health facilities	[blue]	[green]	[green]
	Engagement of community leaders	[blue]	[blue]	[blue]
	Support groups for caregivers	[green]	[blue]	[green]
	School outreach for health education	[green]	[green]	[green]
	Promotion through media (e.g., radio)	[blue]	[blue]	[blue]

For peer review only - http://bmjopen.bmj.com/site/about/guidelines.xhtml

4. Discussion

Notions of strengthening national governance structures have historically been recognized through country-level application of global guidelines to improve vaccine delivery and health systems; however, existing research lacks concrete descriptions of what this entails. This paper augments findings from existing literature by highlighting how structural and contextual factors impact implementation decisions that have led to improvements in childhood vaccine delivery. We developed evidence-derived operational definitions of structures and processes that we identified as critical to the success of vaccine delivery systems in countries with high routine immunization coverage. We described *how* these processes were utilized in high-performing countries to drive catalytic improvements in coverage. Previous research has not explored the details or importance of governance, financing, health systems, or other contextual factors; our original framework based on preexisting literature (Figure 1) was missing complexity in these components, which is indicative of the limited knowledge in this area. Describing how national governance structures operate with the consideration of contextual factors was essential to our understanding of how global guidelines should be adapted to reflect local priorities and challenges. Ignoring a structured approach to understanding these factors could lead to repeated instances of sub-optimal program performance.

Our data points to the need for programmers and policy makers to better understand underlying governance structures. The structures and processes we examined in Nepal, Senegal, and Zambia supported adaptive decision-making based on context-specific needs and priorities. Adaptation at all levels enabled tailored and targeted programming based on evaluation of high-quality data. While health system improvements led by national governments were essential, our data also highlighted the importance of a deliberate and consistent focus on community engagement, which aligns with existing literature [1,27,28]. Community involvement in vaccine programming facilitated the government's understanding of cultural and social barriers and enablers which contributed to effective adaptation of interventions to increase coverage. Community ownership was fostered through national policies and resources for capacity building, engagement of traditional leaders, and the strength of the community health worker programs.

We found that many improvements in immunization coverage emerged from adjacent successes in health systems strengthening (HSS). While an unsurprising finding that health system strengthening would be an essential ingredient in support of vaccine system strengthening, it points to the need for a more integrated, rather than siloed, approach to child health. HSS has become a priority in recent decades as disruptions due to natural disasters, disease outbreaks, or civil unrest have exposed weaknesses in health systems [30, 31]. Although we focused on examining success factors for vaccine delivery, our data also suggests that there are still gaps and challenges in exemplar countries, including: 1) equitable access (e.g., lack of health posts in rural and remote areas); 2) long-term financing (e.g., most funding comes from external partners); 3) poor infrastructure (e.g., roads, electricity); and 4) a heavy reliance on volunteer community health workers which experienced vacancies and decreased morale in some cases.

In Nepal, Senegal, and Zambia, overall commitment to health and vaccines strengthened governance processes and supported vaccination efforts through prioritization of activities and resource allocation. Following the completion of GVAP in 2020, stakeholders reported that lack of consideration of existing structures and contextual factors limited the plan's success [32]. In addition, the monitoring and evaluation processes proposed through GVAP were often seen as unrealistic [9, 32, 33]. Suggested improvements included engaging with countries to accurately and sufficiently consider local context while setting global goals and targets and promoting country ownership of strategic planning [32, 33]. Our research revealed that national government ownership of strategic planning was critical to these countries' success, which aligns with the critical reflection of GVAP and other existing literature.

For peer review only - http://bmjopen.bmj.com/site/about/guidelines.xhtml

Overall, the strategies implemented by exemplar countries align with the WHO Comprehensive Framework of Strategies and Practices for Routine Immunization, which focus on managing the immunization program (e.g., political commitment, partnerships), mobilizing communities, generating demand, and monitoring progress [1]. Throughout the study, our analysis returned to the importance of defining and understanding the context, governance, financing, and health systems within a country, rather than focusing on any one intervention. This highlights the importance of further research into these structural factors. We examined how these strategies were operationalized to improve coverage in Nepal, Senegal, and Zambia, and how they can be applied in other settings. Understanding how governance processes function in high-performing countries may allow others to build stronger systems, adapt guidelines, and reflect on progress made in the field. Our research introduces areas of inquiry for further investigation, as the critical success factors that primarily emerged in all three exemplar countries are not the current focus within existing literature around determinants for vaccine coverage. as the critical success factors that primarily emerged in all three exemplar countries are not the current focus within existing literature around determinants for vaccine coverage.

5. Limitations

This study has several limitations. First, it was difficult to determine causation because we focused on countries that were successful in vaccine delivery but were unable to carry out a similar analysis in a country with lower vaccination coverage to compare. Second, the research tools focused on the factors that drove catalytic change and did not focus on interventions or policies that were unsuccessful. Third, using qualitative methods to understand historical events was challenging; interviewees often spoke about current experiences rather than discussing historical factors. However, we probed respondents to reflect on longitudinal changes in the immunization program. Additional country-specific limitations can be found elsewhere [15-17].

6. Conclusion

Through focusing on countries with high routine immunization coverage, we examined how vaccine delivery systems may leverage components of existing governance structures and health systems to accelerate and sustain coverage. Operational definitions for governance, health systems strengthening, community engagement, and adaptive capacity, along with descriptions of how these processes were implemented in high-performing countries, may help other countries implement similar improvements. Our findings highlight the need for program managers and policy makers to understand and consider the strengths and limitations of existing structures while adapting to the emergent needs and priorities relevant to the context. Our study looked retroactively on how and why countries succeeded in achieving high early childhood immunization coverage. The COVID-19 pandemic has highlighted the fragility in our health systems, and specifically how robust vaccine systems can rise to the challenge of emergent vaccination needs. While our findings are not immediately applicable to the current COVID vaccination needs, our underlying approach to understand context remain relevant to consider as countries strive to vaccinate their populations in the ongoing pandemic [34].

For peer review only - http://bmjopen.bmj.com/site/about/guidelines.xhtml

Acknowledgements:
We thank the Center for Family Health Research in Zambia, the Center for Molecular Dynamics Nepal, and the Institut de Recherche en Santé de Surveillance Epidemiologique et de Formation in Dakar, Senegal. We gratefully acknowledge the participants who gave their time and insights to help us better understand the vaccine delivery systems of Zambia, Nepal, and Senegal, along with facilitators from their respective Ministries of Health.

In addition, we thank Sarah Chesemore, Anna Rapp, Tove Ryan, and Ethan Wong from the Bill and Melinda Gates Foundation; Kate Buellesbach, Nancy Fullman, Nathaniel Gerthe, Gloria Ikilezi, Caitlyn Mason, David Phillips, and Oliver Rothschild, Jordan-Tate Thomas, and Angela Wang from Gates Ventures; and the Vaccine Exemplars Research Advisory Group for their insights, specifically Agnes Binagwaho, Laura Craw, Carolina Danovaro, Anuradha Gupta, Heidi Larson, Penelope Masumbu, Kate O'Brien, Helen Rees, Lora Shimp, and Aaron Wallace.

More information about how this study addresses local research and policy priorities is included in our Reflexivity Statement (Appendix 3).

Funding:
This work was supported by the Bill & Melinda Gates Foundation, Seattle, WA (OPP1195041) with a planning grant from Gates Ventures, LLC, Kirkland, WA.

Competing interests:
The authors declare that they have no known competing financial interests or personal relationships that could have appeared to influence the research reported in this paper.

Data sharing statement:
Data are not publicly available as all data are confidential. De-identified data may be available upon request.

Contributorship statement: KAH, ASE, SD, WK, MS, RAB, MCF: project conceptualization and methodology; KAH, ZS, ASE, KR, SD, WK, MS, RAB, MCF: investigation and data curation; KAH, ZS, ASE, KR, EAO, RAB, MCF: formal analysis; ZS, KAH: writing - original; KAH, ZS, ASE, EAO, KR, SD, WK, MS, RAB, MCF: writing – review and editing; all authors provided approval of the final version.

Ethics approval statement: This study was considered exempt by the Institutional Review Board committee of Emory University, Atlanta, Georgia, USA (IRB00111474); approved by the Nepal Health Research Council (NHRC; Reg. no. 347/2019) in Kathmandu, Nepal; the National Ethical Committee for Health Research (CERNS; Comité National d'Ethique pour la Recherche en Santé) in Dakar, Senegal (00000174); the University of Zambia Biomedical Research Ethics Committee (Federal Assurance No. FWA00000338, REF. No. 166-2019); and the National Health Research Authority in Zambia. All ethics committees abide by the principles of the Declaration of Helsinki; all participants provided written consent.

For peer review only - http://bmjopen.bmj.com/site/about/guidelines.xhtml

1. World Health Organization, *Immunization Agenda 2030: A Global Strategy to Leave No One Behind*. 2020.
2. Vanderslott, S. and T. Marks, *Charting mandatory childhood vaccination policies worldwide.* Vaccine, 2021.
3. Gavi the Vaccine Alliance. *Sustainable Development Goals*. 2020 18 February, 2020; Available from: https://www.gavi.org/our-alliance/global-health-development/sustainable-development-goals.
4. Piot, P., et al., *Immunization: vital progress, unfinished agenda.* Nature, 2019. **575**(7781): p. 119-129.
5. World Health Organization, *Global Vaccine Action Plan 2011–2020* 2013: USA.
6. World Health Organization and UNICEF, *Global Immunization Vision and Strategy 2006-2015*. 2005: New York, NY.
7. World Health Organization. *Immunization coverage*. 2020 July 15, 2020 August 4, 2020]; Available from: https://www.who.int/news-room/fact-sheets/detail/immunization-coverage.
8. World Health Organization and UNICEF, *WHO-UNICEF estimates of DTP3 coverage*. 2020.
9. World Health Organization, *GVAP review and lessons learned: methodology, analysis and results of the stakeholder consultations*, in *Annex to the Global Vaccine Action Plan 2011-2020.* 2019: Geneva.
10. Bednarczyk, R.A., et al., *Protocol: Identification and evaluation of critical factors in achieving high and sustained childhood immunization coverage in selected low- and lower-middle income countries.* medRxiv, 2021: p. 2021.12.01.21267018.
11. Shen, A.K., R. Fields, and M. McQuestion, *The future of routine immunization in the developing world: challenges and opportunities.* Global health, science and practice, 2014. **2**(4): p. 381-394.
12. World Health Organization, *Global Routine Immunization Strategies and Practices (GRISP): a companion document to the Global Vaccine Action Plan (GVAP)*. 2016, Geneva: World Health Organization.
13. World Health Organization, *Reaching Every District (RED): A guide to increasing coverage and equity in all communities in the African Region*. 2017.
14. Phillips, D.E., et al., *Determinants of effective vaccine coverage in low and middle-income countries: a systematic review and interpretive synthesis.* BMC health services research, 2017. **17**(1): p. 681-681.
15. Rodriguez, K., et al., *Critical success factors for routine immunization performance: A case study of Zambia 2000 to 2018.* medRxiv, 2021: p. 2021.11.30.21267060.
16. Sakas, Z., et al., *Critical success factors for high routine immunization performance: A case study of Senegal.* medRxiv, 2022: p. 2022.01.25.22269847.
17. Hester, K.A., et al., *Critical success factors for high routine immunization performance: A case study of Nepal.* medRxiv, 2022: p. 2022.01.28.22270023.
18. Noor, A.M., *Country ownership in global health.* PLOS Global Public Health, 2022. **2**(2): p. e0000113.
19. WHO and UNICEF, *Immunization, DPT (% of children ages 12-23 months)*, W.a. UNICEF, Editor. 2022.
20. Carter, A., et al., *A framework for identifying and learning from countries that demonstrated exemplary performance in improving health outcomes and systems.* BMJ global health, 2020. **5**(12): p. e002938.
21. Exemplars in Global Health. *Making Better Decisions in Global Health: Understand Positive Outliers to Inform Policy and Practice*. 2021; Available from: https://www.exemplars.health/.
22. LaFond, A., et al., *Drivers of routine immunization coverage improvement in Africa: findings from district-level case studies.* Health policy and planning, 2015. **30**(3): p. 298-308.
23. World Health Organization and UNICEF, *WHO-UNICEF estimates of national immunization coverage (WUENIC)*. 2020: Geneva, Switzerland.
24. *Nepal Demographic Health Survey 2001, 2006, 2011, 2016, 2017*, DHS Program, Editor.
25. Damschroder, L.J., et al., *Fostering implementation of health services research findings into practice: a consolidated framework for advancing implementation science.* Implementation science : IS, 2009. **4**: p. 50-50.

For peer review only - http://bmjopen.bmj.com/site/about/guidelines.xhtml

26. Pfadenhauer, L.M., et al., *Making sense of complexity in context and implementation: the Context and Implementation of Complex Interventions (CICI) framework.* Implementation science : IS, 2017. **12**(1): p. 21-21.

27. Hester K, *Open Sciences Framework (OSF) page: Assessing sustainability factors for rural sanitation coverage following the SSH4A approach. https://osf.io/caz9b/?view_only=a1f16382d78b445ab34cc2afedca0cb0.*

28. Sakas, Z., et al., *The role of Zambia's expansive ICC in supporting efficient, effective, and evidence-based vaccine and health sector programming.* medRxiv, 2021: p. 2021.11.29.21267021.

29. Hester, K.A., et al., *Critical interventions for demand generation in Zambia, Nepal, and Senegal with regards to the 5C psychological antecedents of vaccination.* medRxiv, 2022: p. 2022.04.25.22274035.

30. Ozawa, S., L. Paina, and M. Qiu, *Exploring pathways for building trust in vaccination and strengthening health system resilience.* BMC health services research, 2016. **16**(Suppl 7): p. 639-639.

31. World Health Organization Maximizing Positive Synergies Collaborative Group, *An assessment of interactions between global health initiatives and country health systems.* The Lancet, 2009. **373**(9681): p. 2137-2169.

32. Hwang, A., et al., *Global Vaccine Action Plan Lessons Learned II: Stakeholder Perspectives.* Vaccine, 2020. **38**(33): p. 5372-5378.

33. Cherian, T., et al., *Global Vaccine Action Plan lessons learned III: Monitoring and evaluation/accountability framework.* Vaccine, 2020. **38**(33): p. 5379-5383.

34. Dixit, S.M., et al., *Addressing disruptions in childhood routine immunisation services during the COVID-19 pandemic: perspectives from Nepal, Senegal and Liberia.* BMJ Global Health, 2021. **6**(7): p. e005031.

For peer review only

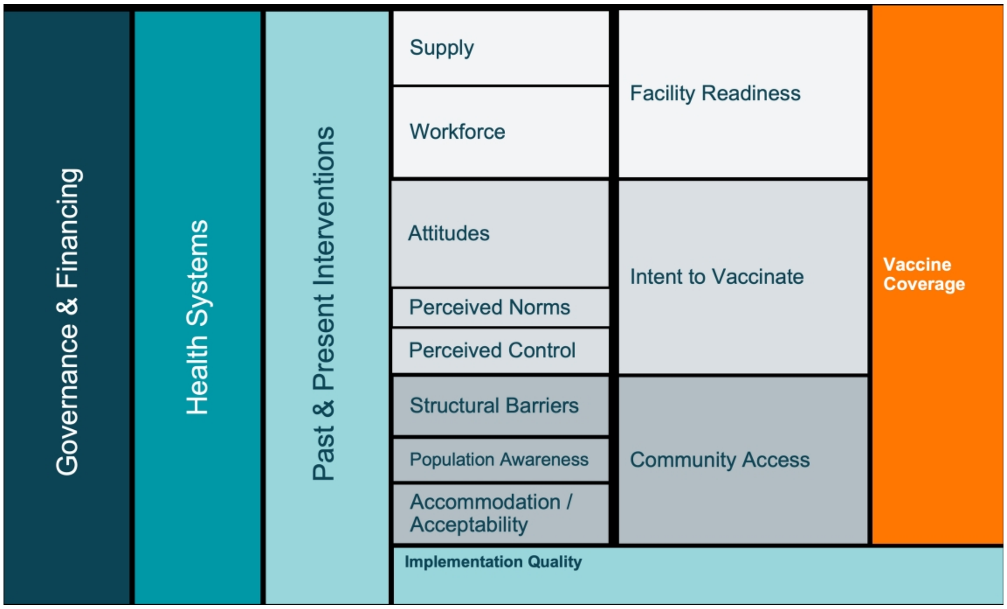

233x140mm (330 x 330 DPI)

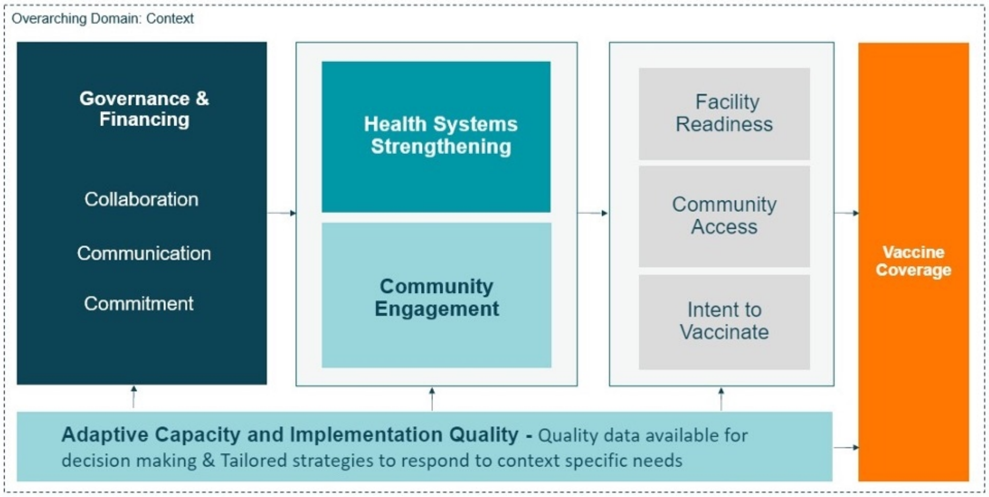

170x87mm (144 x 144 DPI)

Appendix 1: Coverage data for Nepal, Zambia, and Senegal

DHS DTP3 coverage in Zambia, by Province, 2000 – 2016

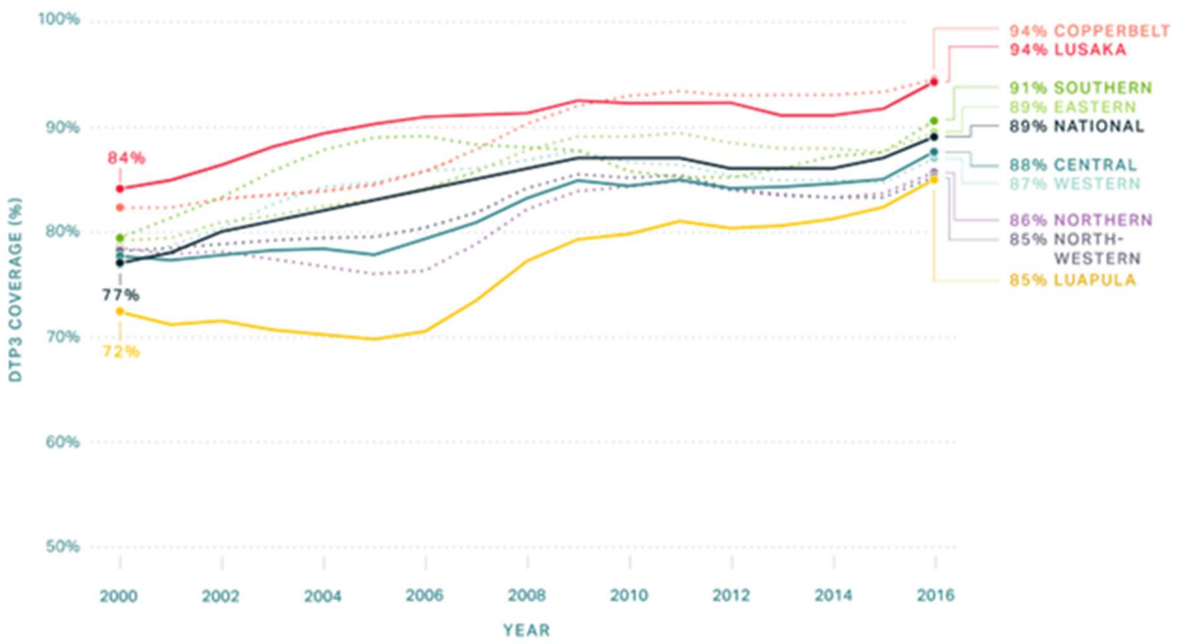

DHS DTP3 coverage in Nepal, by Province, 2000 – 2016

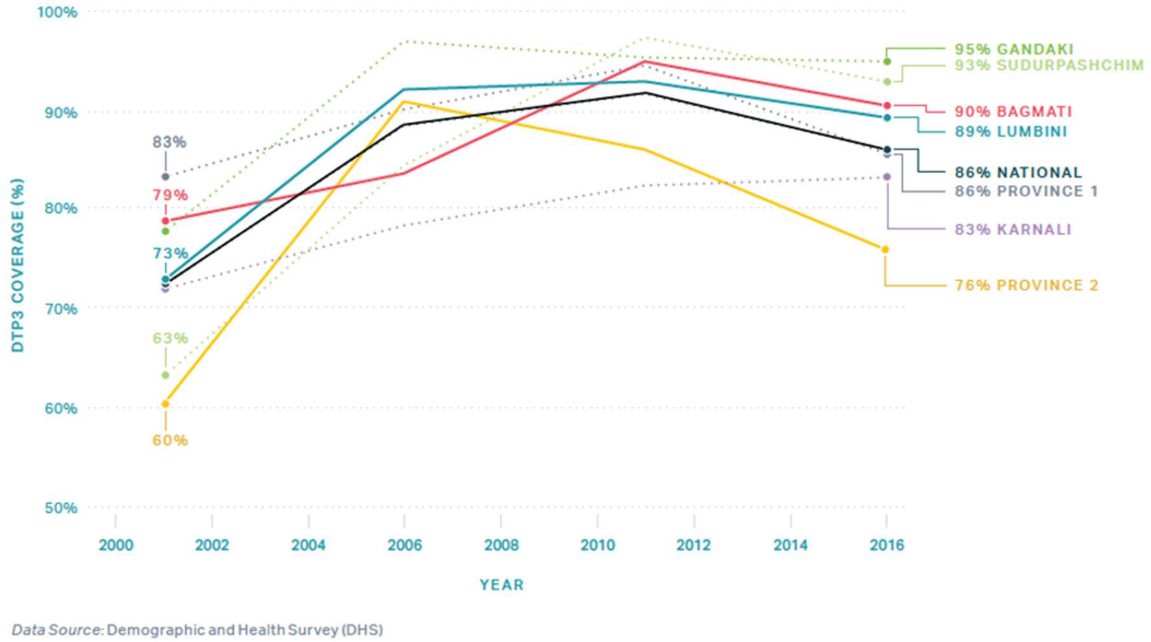

Data Source: Demographic and Health Survey (DHS)

DHS DTP3 coverage in Senegal, by Province, 2000 – 2016

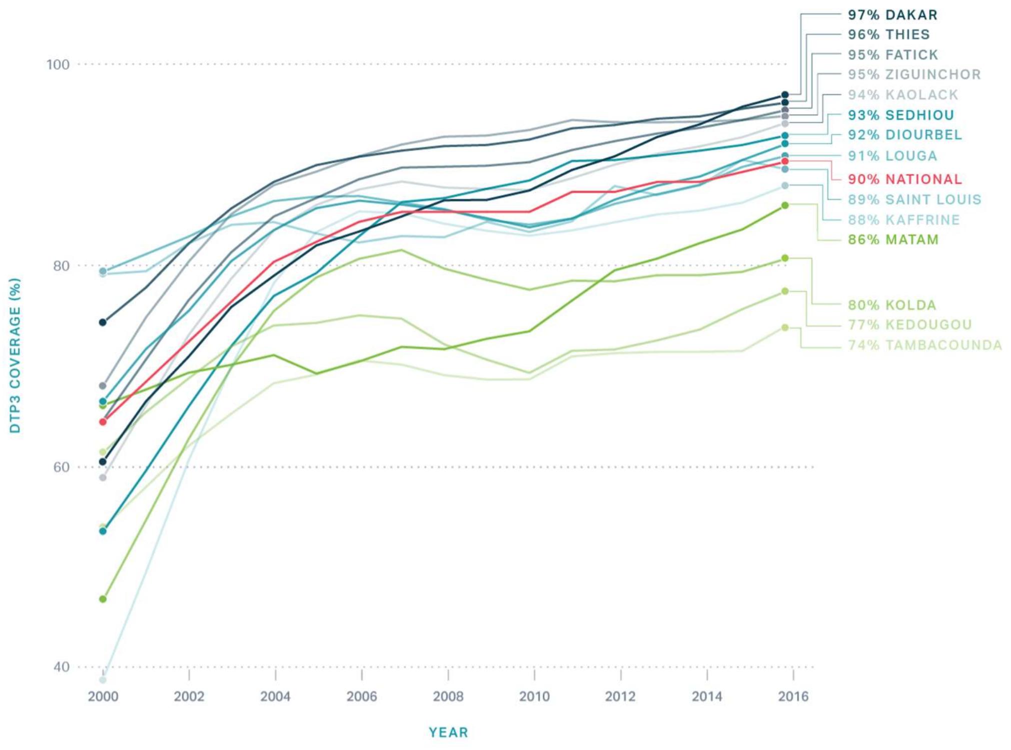

Data Source: IHME DPT Vaccine Coverage Geospatial Estimates

DTP3 coverage from 2000 to 2018 of Nepal, Senegal, and Zambia

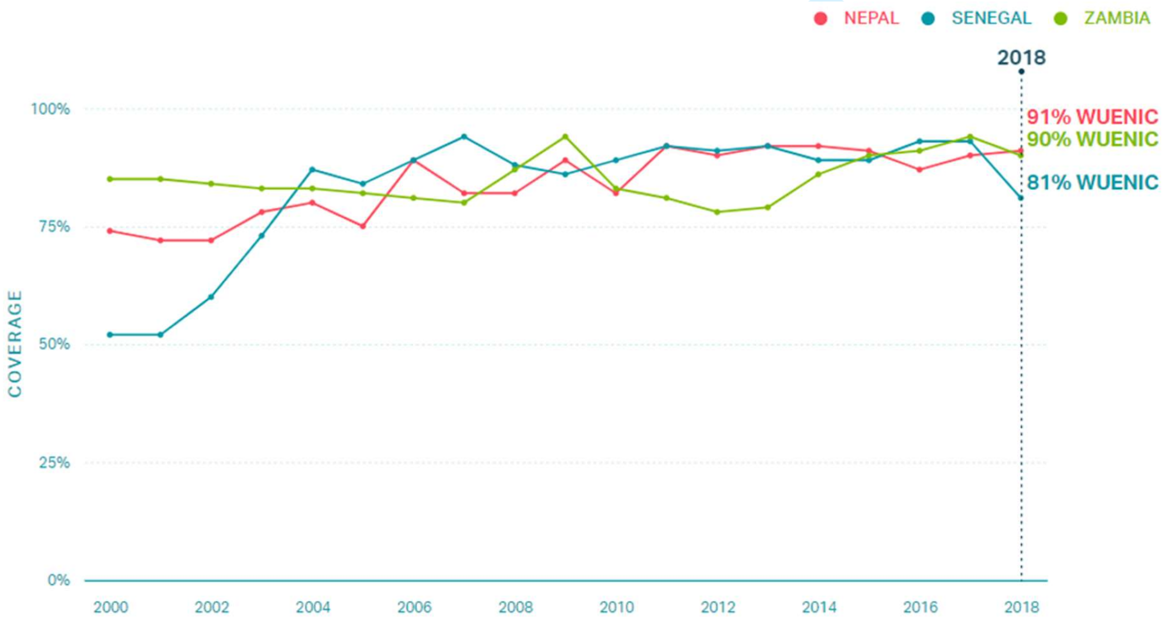

Appendix 2: Additional key informant quotes, by country and domain, illustrating drivers for vaccine coverage from the conceptual framework [15-17]

Domain	Nepal	Senegal	Zambia
Governance and financing<br><br>The processes that guide policies and operations within the health system, including collaboration (effective working relationships, including alignment of priorities) and commitment (dedication to activities and responsibilities).	*"A provision was made in the constitution, and [vaccines] became a fundamental right, so the government has a responsibility to provide. And management of currently running programs also [focuses on] children who have missed vaccines." (Nepal Health Research Council Staff)*	*"When we have a strategic document to work on, the partners get involved according to their area of expertise, and we work together when it comes to implementing and monitoring the vaccine program. In this teamwork, you cannot tell who is the partner or who is from the Ministry of Health, because we work hand in hand. We are in the same boat, we want to have success." (External partner at the national level)*	*"Once you have identified that there is an issue that needs attention, these policies are revised and updated so that they match what is current. [Policies] are more or less like living documents to match the prevailing circumstances. Our planning has improved quite a lot because it's now bottom-up. So, we are the ones that have a lot of input in these documents." (Provincial Health Officer)*
Health systems strengthening<br><br>Strategies that are designed to sustainably improve health system performance (across all sectors) through an integrated and horizontal approach, with a focus on equity.	*"No wards will be deprived of health centers, and if the health center isn't enough to provide health services, then village clinics, immunization centers (outreach clinics), and urban health clinics will be established. If services are provided from these health centers, quality of the vaccines are maintained, and health centers are accessible to all - then good immunization coverage can be achieved." (Department of Health Services within the MOH)*	*"We tried to see what was hidden behind the weak coverage - especially the poor availability of resources including health structures and sufficient staff... a diagnosis was made to improve the indicators, health structures, i.e. creating a lot of health posts, and recruiting staff to be set up in each health post, or at least one vaccination unit to really try to reach out to the entire population in order to have good coverage." (National level stakeholder)*	*"Previously, there was no public health department at a grassroots level, it was just at the National level... So, now the Public Health Specialist is on the ground to try to see that this primary health care activity is promoted. And I think with that supervision, with the introduction of the public health nurses, Health promotion departments, I think these interventions couldn't be achieved higher in terms of immunization activities." (District Health Director)*
Community engagement<br><br>Responsibility of the community to implement vaccination program activities through local leadership and volunteers, fostered by government initiatives and cultural values.	*"Mothers' groups are responsible to give education and information to the parents.... If they don't support the program, then it won't be successful. They are the ones who build awareness and give knowledge and information to the people at the grassroots level." (Department of Health Services in the Ministry of Health and Population)*	*"Community health workers play a very important role – they are in direct contact with the population. They are the ones who live in the community, and they identify more with the community. So, there is a relationship of trust between them and the population." (National level government stakeholder)*	*"We make sure that the community is fully engaged and aware of the benefit of ensuring that their children are vaccinated. It is important that we ensure that within the community, the key gatekeepers are helping us." (Provincial Health Officer)*

For peer review only

Adaptive capacity<br><br>Ability to sufficiently consider contextual factors, supported by timely evaluation of quality data, to tailor vaccine programming based on local needs and priorities.	*"Using HMIS, which is our biggest data source, we send daily, weekly, or monthly reports to districts using phones. We visit districts and contact the responsible person. We understand the situation and we try to solve those problems. We don't depend on the system. We explore, analyze, and address if there are issues outside the system. We do review meetings at the end of the year from the lessons we learned. We also make periodic updates." (Regional MoH Director)*	*"During regional-level meetings, we hear good ideas from the districts. When Dakar had a measles epidemic, we brought together all the districts to tell us how to plan in order to have good measles vaccine coverage. Some of the districts came up with strategies that we found very inspiring. One of them was to hold the vaccination sessions beyond regular work hours to reach the people who are not free during the day. The other strategy was to organize sessions on weekends. As soon as we put these strategies in place, we noticed that the coverage increased right away. It dawned on us that the parents did not refuse the vaccination but that they were busy by their daily occupations." (External partner at the national level)*	*"We have been advising facilities that they need to own the data; they have to make sure that they utilize it by analyzing... If they are not meeting their targets, they need to reflect on the data and see where they are going wrong... For those [health facilities] that are performing well, we find out what they are doing, and we try to replicate to the other facilities that are not doing as well." (District Health Officer)*

For peer review only

Appendix 3 – Reflexivity Statement

1. How does this study address local research and policy priorities?
To address local needs, the Emory study team partnered with local research groups in Nepal, Zambia, and Senegal. This partnership ensured the study was aligned with the context and priorities of each country. Data collection tools were iterative and were created with advice and input from both in-country researchers, local stakeholders, and external technical advisors. The tailoring of the project to each country allowed for this hypothesis generating study to best reflect the main needs and priorities of the countries surveyed.

2. How were local researchers involved in study design?
Local researchers were key to the realization of this project. The in-country study teams helped draw our KII list of key stakeholders and past influential leaders, along with helping to secure meetings. We worked with the local teams to develop our tools, and to edit the questions or procedures as needed based on cultural and political considerations. Partners also helped to determine the best locations for our sample and were responsible for finding individuals for FGDs [1].

3. How has funding been used to support the local research team?
The local research teams were treated as sub-contractors on this project and were paid fairly for their time and effort on this project. We did not have any free or un-funded contributions of local researchers on behalf of the project.

4. How are research staff who conducted data collection acknowledged?
Research staff who conducted data collection were acknowledged as authors in their respective country case studies [2-4].

5. Do all members of the research partnership have access to study data?
In-country research partners have access to all KII and FGD transcripts from their respective countries.

6. How was data used to develop analytical skills within the partnership?
While we did not focus on capacity building during this study, the Emory research staff conducted trainings with each in-country research group. These trainings were over 7 – 14 days, and reviewed the study protocols, data collection guidelines, and general qualitative research best practices.

7. How have research partners collaborated in interpreting study data?
Within each country level case study, our in-country partners were actively engaged in interpretations; the study teams had weekly meetings to discuss the progress and findings of KIIs and FGDs. In addition, partners were involved in the drafting of the manuscripts – the case studies and this cross-cutting synthesis [2-4].

8. How were research partners supported to develop writing skills?
Our research partners collaborated on the manuscript drafts and were added as authors for their contributions. Additionally, several student graduate assistants were not American, and from LMICs; students were added as authors for their contributions.

9. How will research products be shared to address local needs?
Results from this study have already been disseminated to the respective leaders and stakeholders of each country; the meeting was led by the in-country PI and was attended by the Emory research team. A country-specific report, along with the manuscripts, were disseminated. We met with our TAG to disseminate results to global immunization leaders, and continue to collaborate with the in-country PIs on opportunities for additional outreach.

For peer review only

10. How is the leadership, contribution and ownership of this work by LMIC researchers recognised within the authorship?

On this cross-country synthesis, the in-country PIs are added as senior authors. For the individual case studies, all in-country researchers are included as authors [2-4].

11. How have early career researchers across the partnership been included within the authorship team?

In country research partners hired their own staff, and there were different levels of experience represented across the researchers. Early career researchers were included and mentored within each in-country research team, as well as within the Emory team.

12. How has gender balance been addressed within the authorship?

While the Emory and in-country PI's are men, most of the research staff was comprised of women. Guidance for authorship was drafted prior to writing manuscripts, and all were given opportunities to contribute. Those who met the criteria were included as authors; individual contributions, and specific authors, are included on the country case studies [2-4]. This manuscript is the synthesis, and country-level PIs and the Emory team are included as authors.

13. How has the project contributed to training of LMIC researchers?

This project had 7 – 14 day trainings for each country's research team, wherein best practices for qualitative research were taught along with the study's aims and tools. In-country PI's provided additional assistance and training to staff as needed, however, our collaborators were all well-trained professionals and knowledgeable in their areas of expertise.

14. How has the project contributed to improvements in local infrastructure?

This project was a hypothesis generating study, and therefore did not contribute to infrastructure improvement. However, we hope the findings of this study will be of use to program managers and funders in strengthening and building further immunization infrastructure that is both resilient and sustainable.

15. What safeguarding procedures were used to protect local study participants and researchers?

KII and FGD participants gave researchers signed, informed consent; their information was transcribed and de-identified. See our protocol and case study papers for further information [1-4]. There was only minimal risk to both participants and researchers; local PI's were responsible for any context specific safeguarding.

For peer review only

1. Bednarczyk, R.A., et al., *Exemplars in vaccine delivery protocol: a case-study-based identification and evaluation of critical factors in achieving high and sustained childhood immunisation coverage in selected low-income and lower-middle-income countries.* BMJ Open, 2022. **12**.
2. Micek, K., et al., *Critical success factors for routine immunization performance: A case study of Zambia 2000 to 2018.* Vaccine X, 2022. **11**.
3. Hester, K.A., et al., *Critical success factors for high routine immunization performance: A case study of Nepal.* Vaccine X, 2022. **12**(100214).
4. Sakas, Z., et al., *Critical success factors for high routine immunization performance: A case study of Senegal.* medRxiv, 2022: p. 2022.01.25.22269847.

For peer review only

The ENTREQ Checklist

Item	Guide and description	Reported on page #
Aim	State the research question the synthesis addresses	Title & 4
Synthesis methodology	Identify the synthesis methodology or theoretical framework which underpins the synthesis, and describe the rationale for choice of methodology (e.g. meta-ethnography, thematic synthesis, critical interpretive synthesis, grounded theory synthesis, realist synthesis, meta-aggregation, meta-study, framework synthesis).	6-7
Approach to searching	Indicate whether the search was pre-planned (comprehensive search strategies to seek all available studies) or iterative (to seek all available concepts until theoretical saturation is achieved).	5 & supplementary file 1
Inclusion criteria	Specify the inclusion/exclusion criteria (e.g. in terms of population, language, year limits, type of publication, study type).	5
Data sources	Describe the information sources used (e.g. electronic databases (MEDLINE, EMBASE, CINAHL, psychINFO, Econlit), grey literature databases (digital thesis, policy reports), relevant organisational websites, experts, information specialists, generic web searches (Google Scholar), hand searching, reference lists) and when the searches were conducted; provide the rationale for using the data sources.	5
Electronic Search strategy	Describe the literature search (e.g. provide electronic search strategies with population terms, clinical or health topic terms, experiential or social phenomena related terms, filters for qualitative research and search limits).	Supplementary file 1
Study screening methods	Describe the process of study screening and sifting (e.g. title, abstract and full text review, number of independent reviewers who screened studies)	5-6
Study characteristics	Present the characteristics of the included studies (e.g. year of publication, country, population, number of participants, data collection, methodology, analysis, research questions).	6 & 8-10 (Table 1)
Study selection results	Identify the number of studies screened and provide reasons for study exclusion (e.g. for comprehensive searching, provide numbers of studies screened and reasons for exclusion indicated in a figure/flowchart; for iterative searching describe reasons for study exclusion and inclusion based on modifications t the research question and/or contribution to theory development).	8 & Figure 1.

For peer review only

Rationale for appraisal	Describe the rationale and approach used to appraise the included studies or selected findings (e.g. assessment of conduct (validity and robustness), assessment of reporting (transparency), assessment of content and utility of the findings).	5-6
Appraisal items	State the tools, frameworks and criteria used to appraise the studies or selected findings (e.g. Existing tools: CASP, QARI, COREQ, Mays and Pope [25]; reviewer developed tools; describe the domains assessed: research team, study design, data analysis and interpretations, reporting).	5-6
Appraisal process	Indicate whether the appraisal was conducted independently by more than one reviewer and if consensus was required.	6
Appraisal results	Present results of the quality assessment and indicate which articles, if any, were weighted/excluded based on the assessment and give the rationale.	11-13 (Table 2 & 3).
Data extraction	Indicate which sections of the primary studies were analysed and how were the data extracted from the primary studies? (e.g. all text under the headings "results /conclusions" were extracted electronically and entered into a computer software).	6
Software	State the computer software used, if any.	6
Number of reviewers	Identify who was involved in coding and analysis.	6
Coding	Describe the process for coding of data (e.g. line by line coding to search for concepts).	6-7
Study comparison	Describe how were comparisons made within and across studies (e.g. subsequent studies were coded into pre-existing concepts, and new concepts were created when deemed necessary).	6-7
Derivation of themes	Explain whether the process of deriving the themes or constructs was inductive or deductive.	6-7 & 12
Quotations	Provide quotations from the primary studies to illustrate themes/constructs, and identify whether the quotations were participant quotations or the author's interpretation	14-23
Synthesis output	Present rich, compelling and useful results that go beyond a summary of the primary studies (e.g. new interpretation, models of evidence, conceptual models, analytical framework, development of a new theory or construct).	24-29

For peer review only

Search Strategy

1. Falls.mp. or exp falling/
2. Fall*.mp
3. 1 or 2
4. Exp prevention/ or exp primary prevention/ or exp accident prevention/ or prevention.mp
5. Prevent*.mp
6. 4 or 5
7. 3 and 6
8. Exp exercise/ or exercise.mp
9. Physical activity.mp. or exp physical activity/
10. Motor activity.mp or exp motor activity/
11. (exercis* or activit*).mp. [mp=title, abstract, heading word, drug trade name, original title, device manufacturer, drug manufacturer, device trade name, keyword, floating subheading word]
12. 8 or 9 or 10 or 11
13. 7 and 12
14. (patient* adj6 (complian* or participat* or satisf* or adher* or refus* or attitude* or experience* or perception* or engage*)).mp. [mp=abstract, heading words, title]
15. (patient* AND (complian* or participat* or satisf* or adher* or refus* or attitude* or experience* or perception* or engage*)).mp. [mp=abstract, heading words, title]
16. 14 or 15
17. 13 and 16
18. Longitudinal studies.mp. or exp longitudinal study/
19. (long term or long-term).mp. [mp=title, abstract, heading word, drug trade name, original title, device manufacturer, drug manufacturer, device trade name, keyword, floating subheading word]
20. (follow up or follow-up).mp. [mp=title, abstract, heading word, drug trade name, original title, device manufacturer, drug manufacturer, device trade name, keyword, floating subheading word]
21. 18 or 19 or 20
22. 17 and 21
23. Qualitative studies.mp or exp qualitative research/
24. Phenomenology.mp or exp phenomenology/

For peer review only

25. Ethnography.mp or exp ethnography/

26. Grounded theory.mp or exp grounded theory/

27. Narrative.mp. or exp narrative/

28. 23 or 24 or 25 or 26 or 27

29. 22 and 28

For peer review only

BMJ Open

Critical success factors for high routine immunization performance: Qualitative analysis of interviews and focus groups from Nepal, Senegal, and Zambia

Journal:	*BMJ Open*
Manuscript ID	bmjopen-2022-070541.R1
Article Type:	Original research
Date Submitted by the Author:	01-Jun-2023
Complete List of Authors:	Sakas, Zoe; Rollins School of Public Health<br>Hester, Kyra; Emory University, Rollins School of Public Health<br>Ellis, Anna; Emory University, Rollins School of Public Health 1518 Clifton Rd Atlanta, GA, USA 30322<br>Ogutu, Emily; Emory University, Gangarosa Department of Environmental Health<br>Rodriguez, Katie; Emory University<br>Bednarczyk, Robert ; Emory University, Hubert Department of Global Health<br>Dixit, Sameer; Center for Molecular Dynamics Nepal<br>Kilembe, William; Emory University, Rwanda Zambia HIV Research Group<br>Sarr, Moussa; Institut de Recherche en Santé de Surveillance Epidémiologique et de Formations ,<br>Freeman, Matthew; Emory University, Gangarosa Department of Environmental Health
**Primary Subject Heading**:	Global health
Secondary Subject Heading:	Global health
Keywords:	Paediatric infectious disease & immunisation < PAEDIATRICS, PUBLIC HEALTH, QUALITATIVE RESEARCH

SCHOLARONE™
Manuscripts

I, the Submitting Author has the right to grant and does grant on behalf of all authors of the Work (as defined in the below author licence), an exclusive licence and/or a non-exclusive licence for contributions from authors who are: i) UK Crown employees; ii) where BMJ has agreed a CC-BY licence shall apply, and/or iii) in accordance with the terms applicable for US Federal Government officers or employees acting as part of their official duties; on a worldwide, perpetual, irrevocable, royalty-free basis to BMJ Publishing Group Ltd ("BMJ") its licensees and where the relevant Journal is co-owned by BMJ to the co-owners of the Journal, to publish the Work in this journal and any other BMJ products and to exploit all rights, as set out in our licence.

The Submitting Author accepts and understands that any supply made under these terms is made by BMJ to the Submitting Author unless you are acting as an employee on behalf of your employer or a postgraduate student of an affiliated institution which is paying any applicable article publishing charge ("APC") for Open Access articles. Where the Submitting Author wishes to make the Work available on an Open Access basis (and intends to pay the relevant APC), the terms of reuse of such Open Access shall be governed by a Creative Commons licence – details of these licences and which Creative Commons licence will apply to this Work are set out in our licence referred to above.

Other than as permitted in any relevant BMJ Author's Self Archiving Policies, I confirm this Work has not been accepted for publication elsewhere, is not being considered for publication elsewhere and does not duplicate material already published. I confirm all authors consent to publication of this Work and authorise the granting of this licence.

For peer review only

Title: Critical success factors for high routine immunization performance: Qualitative analysis of interviews and focus groups from Nepal, Senegal, and Zambia

Authors: Zoe Sakas MPH[a], Kyra A. Hester MPH[a], Anna S. Ellis MDP[a], Emily Awino Ogutu MPH[a], Katie Rodriguez MPH[a], Robert A. Bednarczyk PhD[a], Sameer Dixit PhD[b], William Kilembe MD[c], Moussa Sarr MD[d], Matthew C. Freeman PhD[a*]

* Corresponding author:
Matthew C. Freeman, PhD
matthew.freeman@emory.edu

Author affiliations:
a Rollins School of Public Health, Emory University, Georgia, USA
b Center for Molecular Dynamics Nepal, Kathmandu, Nepal
c Center for Family Health Research in Zambia, Lusaka, Zambia
d Institut de Recherche en Santé de Surveillance Epidemiologique et de Formation, Dakar, Senegal

Abstract (291 words):
INTRODUCTION: Vaccination averts an estimated 2-3 million deaths annually. Although vaccine coverage improvements across Africa and South Asia have remained relatively stagnant and below global targets, several countries have outperformed their peers with significant increases in routine immunization coverage. Examining these countries' vaccination programs provides an opportunity to identify and describe critical success factors that may have supported these improvements.

METHODS: We selected three high-performing countries in regard to childhood vaccination: Nepal, Senegal, and Zambia. This multiple case study analysis was conducted using data from the Exemplars in Vaccine Delivery project within the Exemplars in Global Health program. We used qualitative analysis to investigate factors that contributed to high vaccination coverage through 66 key informant interviews (KIIs) and 22 focus group discussions (FGDs) at the national, regional, district, health facility, and community levels – with a total of 678 participants. Qualitative data were collected from October 2019 to April 2021. We triangulated these findings with quantitative analyses using publicly available data, which are published elsewhere.

RESULTS: Our data revealed that the critical success factors for vaccine programming relied on the cultural, historical, and statutory context in which the interventions were delivered. In Nepal, Senegal, and Zambia, high immunization coverage was driven by 1) strong governance structures and healthy policy environments; 2) adjacent successes in health systems strengthening; 3) government-led community engagement initiatives; and 4) adaptation considering contextual factors at all levels of the health system.

CONCLUSION: Throughout the study, our analysis returned to the importance of defining and understanding the context, governance, financing, and health systems within a country, rather than focusing on any one intervention. This paper augments findings from existing literature by highlighting how contextual factors impact implementation decisions that have led to improvements in childhood vaccine delivery. Findings from this research may identify transferable lessons and support actionable recommendations to improve national immunization coverage in other settings.

Strengths and limitations of this study:

- This study was conducted by a multi-disciplinary team with authors and advisors from a variety of sectors, including immunization, epidemiology, political science, implementation science, and behavioral science.

For peer review only - http://bmjopen.bmj.com/site/about/guidelines.xhtml

52 - Our team ensured the involvement of stakeholders, researchers, and policy makers in the global immunization sector during the design, implementation, and dissemination of this project. On an ongoing basis, findings were disseminated to in-country experts and government officials, along with our Technical Advisory Group, for feedback and contextualization.
56 - Through extensive qualitative data collection, we were able to gain insight from stakeholders at all levels of the vaccine delivery systems in our target countries – from mothers to community health workers, to national-level leadership.
59 - It was difficult to determine causation because we focused on countries that were successful in vaccine delivery but were unable to carry out a similar analysis in countries with lower vaccination coverage to compare.
62 - Using qualitative methods to understand historical events was challenging; interviewees often spoke about current experiences rather than discussing historical factors.

For peer review only

For peer review only - http://bmjopen.bmj.com/site/about/guidelines.xhtml

1. Introduction

Vaccination is recognized as one of the most influential public health interventions, averting an estimated 2-3 million deaths annually [1-4]. The Global Vaccine Action Plan (GVAP) targeted at least 90% country-level coverage of the third dose of diphtheria, tetanus, pertussis vaccine (DTP3) among 1-year-old children [5], a globally recognized proxy for vaccination system performance [6]. However, by 2018 only 95 of the 193 World Health Organization (WHO) Member States achieved national GVAP targets, and less than one third met district level targets of 80% DTP3 [7-9]. The COVID-19 pandemic sent shocks through vaccination programs worldwide, impacting coverage and causing decreases in overall coverage from 2020 on. However, the global impact of the pandemic was not examined as part of this study as a pre-pandemic system was our focus.

In 2019, the African Region reported the lowest DTP3 coverage at 74%, a slight improvement from 71% coverage in 2010 [8]. DTP3 coverage in the South-East Asia Region substantially improved by 2019, to 91%; however, from 2000 to 2016, this region reported one of the lowest coverage rates with an average of 78% [8]. Within both regions, several countries outperformed their peers with significant increases in routine immunization coverage since 2000 [10]. Examining these countries' vaccination programs provides an opportunity to identify and describe critical success factors that may have supported these improvements.

The essential components of an effective vaccine delivery system are well-established and include strong governance and leadership, healthcare financing, human resources, a robust supply chain, community engagement, and information systems for evidence-based decision-making [11-13]. Research on behaviors related to routine immunization focuses on recognized determinants of coverage, including intent to vaccinate, community access, and health facility readiness [14]. Although existing literature describes vaccine delivery systems, there is a gap in knowledge related to how programs and policies are implemented, how strategies are operationalized, and how context influences programming. By closely examining the vaccine delivery systems of three countries with high vaccination rates, we can draw conclusions about *how* and *why* vaccine programs work in specific contexts, and how they may be tailored for success elsewhere. This paper augments findings from existing literature by highlighting how structural and contextual factors – including governance, collaboration, the environment, and cultural norms - impact implementation decisions that have led to improvements in childhood vaccine delivery.

Our research describes how countries with high vaccination rates took similar paths to success [15-18]. Through investigating drivers of high routine immunization coverage, our findings revealed the importance of structural factors, including governance processes and collaboration between in-country stakeholders, which more broadly align with requirements for strong national health systems. Global DTP3 coverage has plateaued since 2010 at 86% [19]; identifying factors and mechanisms for accelerating progress is critical for expanding vaccination program reach and retention [9].The purpose of this study was to identify critical success factors that contributed to catalytic growth in childhood routine immunization coverage. Findings from this research may identify transferable lessons and support actionable recommendations to improve national immunization coverage in other settings [20].

For peer review only - http://bmjopen.bmj.com/site/about/guidelines.xhtml

2. Methods

This multiple case study analysis was conducted using data from the Exemplars in Vaccine Delivery project within the Exemplars in Global Health program, funded by the Gates Foundation and executed through Gates Ventures [10, 20, 21]. The Exemplars in Vaccine Delivery project involved qualitative examination of the national vaccine delivery systems of three low- and middle-income countries – namely Nepal, Senegal, and Zambia – with high childhood routine immunization coverage, compared to their peers.

This paper comprises a multiple-case study, which compares the findings from three individual country-level case studies. This comparison was design to review which success factors were operationalized in all three exemplary countries, as well as highlight how differences in context and resources impacted operationalization of the key strategies that emerged from our qualitative data. Here, we outline the main factors that impacted successful vaccine delivery in Nepal, Senegal, and Zambia; the individual country-level papers, provide additional detail regarding country-specific implementation.

We used qualitative analysis to investigate factors that contributed to high vaccination coverage through key informant interviews (KIIs) and focus group discussions (FGDs) at the national, regional, district, health facility, and community levels. We triangulated these findings with quantitative analyses using publicly available data, which are published elsewhere and referenced throughout this paper [10, 21].

Prior to data collection, we developed a conceptual model (Figure 1) to organize factors that impact childhood vaccine coverage globally. This model was based on the work of Phillips et al. and LaFond et al. alongside a broader review of the vaccine literature [14, 22]. Details regarding the development of this model are available elsewhere [10].

Figure 1. A priori conceptual model of the drivers of vaccine coverage

2.1. Study sites

Methods for country, region, and district selection are detailed elsewhere [10, 15-17]. Briefly, countries were selected based on DTP1 and DTP3 coverage estimates, which served as proxies of the vaccine delivery system, with DTP1 as a proxy of access and DTP3 as a proxy of continued utilization of immunization services (Appendix 1) [10, 23, 24]. Nepal, Senegal, and Zambia were selected as exemplary countries in terms of their routine immunization coverage due to relatively high DTP1 and DTP3 coverage during the timeframe assessed in this study. Three regions within each country were identified in consultation with national stakeholders and available data (Table 1). Partner organizations and Ministry of Health (MoH)[1] officials facilitated site selection and data collection activities.

2.2. Qualitative data collection and analysis

Qualitative data were collected between October 2019 and April 2021 at the national, regional, district, health facility, and community levels in Nepal, Senegal, and Zambia (Table 1). The interview guides were informed by the Consolidated Framework for Implementation Research (CFIR) [25] and the Context and Implementation of Complex Interventions (CICI) framework [26]. KII and FGD guides were translated

---

[1] *Note that each country has a slightly different name for their Ministry of Health (e.g., Ministry of Health and Population in Nepal; Ministry of Health and Social Action in Senegal), but all will be referred to as "MoH" throughout for simplicity.*

For peer review only - http://bmjopen.bmj.com/site/about/guidelines.xhtml

into local languages by research assistants from the respective countries. All interview guides were piloted before use and adjusted iteratively throughout data collection. For each country, an initial list of KIIs was developed with local research partners and MoH officials with snowball sampling used to identify additional key informants. Our sampling approach aimed to include a diverse sample of participants regarding geographic location and demographic qualities. Caregivers and volunteer community health workers were recruited for FGDs from health facility catchment areas with the assistance of local health staff. The duration of KIIs and FGDs averaged one and a half hours. KIIs and FGDs were audio-recorded with the permission of participants. Research files, recordings, and transcriptions were de-identified and password protected. Data analysis processes were generally consistent in all three countries examined in order to allow for thoughtful comparison.

We applied a theory-informed thematic analysis of the transcripts to identify critical success factors. Data analysis was initially conducted individually for each case study, with separate members of our research team leading analysis for each country. However, to ensure consistency and coherence, all data analysts were involved in the review, coding, and summarization of data for all three case studies. We met frequently as a team to discuss overlap and to compare empirical findings. Transcripts were coded and analyzed using MaxQDA2020 software (Berlin, Germany). Coding was completed by researchers at Emory, some of whom also developed and revised the codebook throughout. Coding meetings were held frequently to discuss code definitions and to discuss emerging themes. Researchers from in-country teams were involved through additional meetings to discuss data and ground-truth findings. We considered setting and participant roles while identifying key points and further contextualized data using historical documents and an a priori literature review. Qualitative data collection and analysis tools, including topic guides and codebooks, can be found in Appendix 2 [27].

Following analysis of individual case studies, we explored and discussed the similarities and differences between our findings. Using a multiple-case study approach, we developed conceptual ideas that pulled from all three countries in order to highlight the key components required for successful vaccine delivery (as per analysis of factors from three exemplary countries). Our discussions were rooted in the empirical data, and we supported all conclusions with informative quotations and data summaries. When there were discrepancies, we revisited the raw data from individual case studies as needed.

For peer review only - http://bmjopen.bmj.com/site/about/guidelines.xhtml

Table 1: Summary of countries, regions, districts selected for research, and data collection activities

Country	Nepal		Senegal		Zambia	
In Country Research Partner	Center for Molecular Dynamics, Nepal		Institut de Recherche en Santé de Surveillance, de Surveillance Epidémiologique et de Formation (IRESSEF)		Center for Family Health Research in Zambia	
Data collection period (MM/YYYY)	8/2019 – 12/2019		12/2020 - 4/2021		10/2019 – 02/2020	
**Regions and Districts**						
Region 1	Madhes*	Dhanusha, Bara, Mahottari	Ziguinchor	Ziguinchor, Oussouye, Diouloulou	Lusaka	Lusaka, Rufunsa, Chongwe
Region 2	Bagmati	Makwanpur, Dolakha, Kathmandu	Dakar	Rufisque, Mbao, Keur Massar	Central	Chibombo, Chitambo, Serenje
Region 3	Gandaki Pradesh	Kaski, Myagdi, Nawalparasi	Tambacounda	Tambacounda, Koumpentoum, Goudiry	Luapula	Chipili, Nchelenge, Samfya
**Key Informant Interviews**<br>*Key: Number of KIIs (Participants)*	**79 (79)**		**62 (63)**		**66 (85)**	
National level government staff	11 (11)		5 (5)		11 (12)	
Partner organization staff	8 (8)		4 (4)		11 (15)	
Regional health staff	5 (5)		7 (7)		6 (8)	
District health staff	15 (15)		38 (38)		10 (19)	
Health facility staff	23 (23)		6 (6)		7 (10)	
Community leaders	15 (15)		2 (2)		10 (10)	
Community health workers **	2 (2)		-		11 (11)	
**Focus Group Discussions**<br>*Key: Number of FGDs (Participants)*	**30 (191)**		**19 (128)**		**22 (132)**	
Community health workers **	9 (60)		10 (65)		10 (60)	
Mothers	9 (60)		9 (63)		8 (48)	
Fathers	6 (36)		-		1 (6)	
Grandparents	6 (35)		-		3 (18)	
**Total (per country)**	**109 (270)**		**81 (191)**		**88 (217)**	
**Total (across countries)**	-		-		**278 (678)**	

** Madhes was referred to as Province 2 prior to 2022 (including during data collection)*
*** Includes volunteer community health workers, female community health volunteers (FCHV) – Nepal; vaccinators – Nepal; bajenu gox – Senegal; and neighborhood health committee members – Zambia.*

For peer review only - http://bmjopen.bmj.com/site/about/guidelines.xhtml

2.3. Supplemental quantitative data analysis

Quantitative data were collected through secondary datasets, which included information from the MOH in Zambia, Nepal, and Senegal among other partners. Data were used to estimate routine immunization coverage from 2000 to 2019 and to uncover trends related to significant improvements. Additional analyses were conducted to identify indicators that may be associated with immunization coverage success among low- and lower-middle-income countries using regression models to statistically test financial, development, demographic, and other country-level indicators. These quantitative analyses were published elsewhere [28, 29] . Findings from the quantitative analyses provided additional context for the qualitative findings which informed the results presented here.

2.4. Ethical approval

This study was considered exempt by the Institutional Review Board committee of Emory University, Atlanta, Georgia, USA (IRB00111474); approved by the Nepal Health Research Council (NHRC; Reg. no. 347/2019) in Kathmandu, Nepal; the National Ethical Committee for Health Research (CERNS; Comité National d'Ethique pour la Recherche en Santé) in Dakar, Senegal (00000174); the University of Zambia Biomedical Research Ethics Committee (Federal Assurance No. FWA00000338, REF. No. 166-2019); and the National Health Research Authority in Zambia. All ethics committees abide by the principles of the Declaration of Helsinki; all participants provided written consent.

2.5. Patient and public involvement

The study team ensured the involvement of stakeholders, researchers, and policy makers in the global immunization sector during the design, implementation, and dissemination of this project. We utilized our Technical Advisory Group (TAG) to develop an initial set of questions for scoping visits to our exemplar countries, wherein study members met with in-country partners and experts to further refine the direction and nature of the questions. Scoping visit findings were used to create the final data collection tools [10]. As this is a hypothesis generating study, and no intervention was provided, data collection was targeted to conform to general standards of burden. We adhered to standard best-practices for qualitative research, iteratively implementing feedback from experts and the TAG. While there was community involvement (FGDs), the primary focus of data collection was centered on historical stakeholders who played key roles in the immunization program [10]. Upon completion of each country case study, findings were disseminated to in-country experts and country governments, along with the TAG, allowing for the study team to ground-truth results. The research team at Emory University co-hosted regular meetings with the in-country research partners, experts in a variety of relevant fields, government officials, and other advisors to ask for feedback and contextualization. Researchers would present on methods, findings, and implications following an engaging discussion with advisors that would lead to improvements in our data analysis processes or presentation of findings. Revisions would be distributed through follow-up email conversations and subsequent phone calls with select advisors. Reports, and manuscripts, of findings were sent to those involved.

More information about how this study addresses local research and policy priorities is included in our Reflexivity Statement (Appendix 3).

2.6 Sex and Gender Considerations

Although we did not provide an intervention, the research team took gender differences into consideration. For our FGDs, we worked with groups of both mothers and fathers; fathers were included as to not fall into the bias of children's health being a 'woman's issue'. FGDs were separated by gender to allow participants to feel comfortable sharing their thoughts.

For peer review only - http://bmjopen.bmj.com/site/about/guidelines.xhtml

Due to the historical nature of this study, our KII sample of key stakeholders and leaders leaned male due to prior gender biases and the tendency to place men in positions of importance or power. However, present-day positions were often filled by women, and we sampled women for KIIs as appropriate.

3. Results

3.1. Drivers of successful vaccination programs in exemplar countries

Following analysis of data from Nepal, Zambia, and Senegal, we revisited the conceptual framework developed during study conception (Figure 1). Although existing literature elaborates on factors related to intent to vaccinate, facility readiness, and community access, there is a gap in knowledge related to *how* policies and programs are operationalized to address these determinants – which relies heavily on the existing governance and health systems, engagement of community members (or end-users), and ability to adapt to the context to improve implementation. Our empirical data from Nepal, Senegal, and Zambia revealed that the critical success factors could be best understood by understanding and considering the cultural, historical, and statutory context in which the interventions were delivered, which is highlighted in our revised framework as the "Overarching Domain: Context" encompasses all components of the vaccine delivery system. Details about the cultural, historical, and statutory factors that each country's general "context" was composed of are outlined in the individual case study papers [15,16,17]. This is in contrast to any specific modification or adaptation of key interventions or policies. We revised the initial conceptual framework based on findings from empirical data to illustrate a greater focus on these emergent factors (Figure 2). Definitions for the key domains can be found in Table 2.

Figure 2. Revised conceptual framework for drivers of vaccine coverage

Table 2: Definitions for drivers of vaccine coverage

Domain	Definition
**Governance and financing** [a]	The processes that guide policies and operations within the health system, including collaboration (effective working relationships, including alignment of priorities), communication (open and frequent channels for sharing knowledge and feedback), and commitment (dedication to activities and responsibilities).
**Health systems strengthening** [a]	Strategies that are designed to sustainably improve health system performance (across all sectors) through an integrated and horizontal approach, with a focus on equity.
**Community engagement** [a]	Responsibility of the community to implement vaccination program activities through local leadership and volunteers, fostered by government initiatives and cultural values.
**Adaptive capacity** [a]	Capability to sufficiently consider contextual factors, supported by timely evaluation of quality data, to tailor vaccine programming based on local needs and priorities – and improve implementation quality.
**Intent to vaccinate** [b]	Demand for childhood vaccines on the part of the caregiver or guardian that would result in vaccination in the absence of other barriers.

For peer review only - http://bmjopen.bmj.com/site/about/guidelines.xhtml

**Facility readiness** [b]	Supply (by the health system) of health and vaccination services to adequately meet demand. Incorporates supplies (vials, syringes etc.), human resources, and the consistency of their availability.
**Community access** [b]	The ability (or inability) to successfully carry out the transaction of vaccine utilization – for example, a community member's ability to access vaccines should they decide to get vaccinated - i.e., barriers and facilitators between Intent and Readiness.

a. *Developed from our research, both via data analysis and desk review of existing literature*
b. *See Figure 1 (and reference Phillips et al. paper)*

3.2. Operationalization of successful vaccine delivery programs

In Nepal, Senegal, and Zambia, high immunization coverage was driven by 1) strong governance structures and healthy policy environments; 2) adjacent successes in health systems strengthening; 3) government-led community engagement initiatives; and 4) adaptation considering contextual factors at all levels of the health system. Below, we summarize similarities apparent in all three high-performing countries. Appendix 4 includes informative quotes as they relate to the four domains. More information on how policies and programs were implemented and adapted to local context is available elsewhere [15-17].

Strong governance structures and a healthy policy environment
Effective collaboration, open communication, and commitment to health and vaccines supported policy development and implementation of the vaccination programs in Nepal, Senegal, and Zambia.

Collaboration and communication enabled alignment of priorities, division of responsibilities, and coordination between levels. In all three countries, collaboration was apparent between external partners and the government, and between different levels of the health system. National fora were established for frequent and multi-disciplinary communication, including Inter-Agency Coordinating Committees (ICC) [30]. Regional, district, and community level reflection meetings were held to share and review data and gather feedback for strategic planning. Formal channels for supportive supervision and feedback, both top-down and bottom-up, supported service delivery improvements and reporting.

In all three countries, vaccination was a "priority" or "flagship" program recognized by the MOH. Prioritization of vaccination within the health sector supported sufficient resource allocation. In Nepal and Senegal, dedication to the health sector more broadly was codified through defining health as a right in their national constitutions. Commitment to health and vaccines underpinned policy development and enabled consistent service delivery. Community members could expect reliable and equitable access to vaccines due to the prioritization of activities by health personnel and the dedication of volunteer community health workers who supported outreach activities and spearheaded demand generation and educational campaigns.

> *"A provision was made in the constitution, and [vaccines] became a fundamental right, so the government has a responsibility to provide. And management of currently running programs also [focuses on] children who have missed vaccines." (Nepal Health Research Council Staff, Nepal)*

Adjacent successes in health systems strengthening
Improvements in the broader health systems of Nepal, Senegal, and Zambia – including health post expansion, training of volunteer community health workers, general capacity building, and prioritization of equitable health services – supported high immunization coverage.

For peer review only - http://bmjopen.bmj.com/site/about/guidelines.xhtml

Health post expansion and capacity building at subnational and community levels were mentioned by most key informants in all three countries as essential to improvements in vaccination coverage. Building health facilities in rural areas especially allowed parents to easily vaccinate their children, without having to travel far distances or rely on outreach. Improvements in vaccine service delivery also relied on improvements in general infrastructure including roads, electricity, and connectivity required for transportation, health facility operations, and data reporting. In all three countries, general health systems strengthening was centered around equity as a priority for decision making. Health post expansion and community health worker programs were strengthened to prioritize service delivery in hard-to-reach communities, minority populations, and remote areas.

> *"Previously, there was no public health department at a grassroots level, it was just at the National level… So, now the Public Health Specialist is on the ground to try to see that this primary health care activity is promoted. And I think with that supervision, with the introduction of the public health nurses, Health promotion departments, I think these interventions couldn't be achieved higher in terms of immunization activities." (District Health Director, Zambia)*

Ongoing efforts to address equity concerns include: 1) Overcoming geographical barriers through health post expansion and outreach services supported by national-level policy and external partner funding; and 2) Addressing social, economic, and cultural factors through tailored messaging and local ownership of vaccination programming.

Integrated decision making across health and social sectors, through a horizonal rather than vertical approach, facilitated effective allocation of resources (e.g., monetary, human, technical, educational) could be efficiently allocated across programs. For example, national fora and committees in all three countries included representatives from the immunization sector, maternal and child health, education and development, and financial offices. During meetings, sectors would align long-term priorities and discuss how to reach overall goals in terms of health and social outcomes.

Government-led community engagement initiatives

Community ownership of vaccination activities and corresponding health outcomes was apparent in Nepal, Senegal, and Zambia, and even more so in high-performing districts within these countries. Responsibility of the community to implement vaccination programming through local leadership and volunteers was fostered by government initiatives and cultural values. National policies focused on involvement of traditional leaders and all national-level stakeholders reported on the importance of community actors. In some communities, female volunteers would work beyond their mandates to ensure all children were vaccinated – a decision motivated by dedication to their communities at large, empathy for the children in their neighborhoods, and a vague understanding of herd immunity.

Key informants from the national to community level reported that demand for vaccines was critical for improving routine immunization coverage. In addition to the work of volunteer community health workers, demand generation and public awareness were accomplished through national policies, media engagement, involvement of community actors, and school outreach activities.

> *"Community health workers play a very important role – they are in direct contact with the population. They are the ones who live in the community, and they identify more with the community. So, there is a relationship of trust between them and the population." (National level government stakeholder, Senegal)*

For peer review only - http://bmjopen.bmj.com/site/about/guidelines.xhtml

Further analysis of community health worker programs and demand generation activities in Nepal, Senegal, and Zambia will be detailed in our upcoming publications [31].

Adaptation at all levels of the health system

In Nepal, Senegal, and Zambia, decision-makers and implementers at the national, subnational, and community levels demonstrated their ability to consider contextual factors, evaluate or reflect on quality data, and tailor vaccine programming based on local needs and priorities.

Quality data were available for evidence-based decision making in all three countries. Data software was implemented at the district level in all countries - or in the case of Senegal, at the health post level - allowing stakeholders at all levels to utilize accurate and up-to-date coverage estimates. Lack of quality data in some areas may have hindered this process. However, when data were not available, community actors would adapt programming based on head counts conducted by volunteers and an anecdotal understanding of the contextual and cultural needs in their communities.

> *"We have been advising facilities that they need to own the data; they have to make sure that they utilize it by analyzing… If they are not meeting their targets, they need to reflect on the data and see where they are going wrong… For those [health facilities] that are performing well, we find out what they are doing, and we try to replicate to the other facilities that are not doing as well." (District Health Officer, Zambia)*

Adapting strategies to respond to context-specific needs was essential for reaching children. In all three countries, flexible vaccine delivery days and outreach services supported unique scheduling needs (e.g., in Senegal, vaccinating children in the late afternoon during farming season). Outreach strategies were also tailored to accommodate contextual factors and address barriers to access. Understanding cultural and social values enabled community actors to tailor demand generation activities so that community members would be more apt to adopt positive health behaviors. Some examples of this may be using a local language, involving religious or community leaders in engagement and sensitizing, or addressing cultural or social barriers directly through personal communication.

For peer review only - http://bmjopen.bmj.com/site/about/guidelines.xhtml

3.3. Interventions and programs across high-performing countries

Key informants discussed a myriad of interventions and programs that they believed led to the improved and sustained vaccination coverage in their countries (Table 3). Interventions are organized by implementation level; if an intervention was implemented at multiple levels of the health system, it may be repeated in the table. Although the specific components of vaccine programming were not identical across countries, many of the general themes overlapped (e.g., the scope of community-level health committees varied between countries, but the purpose/objective of these groups was similar).

Table 3: Key interventions and programs implemented to improve routine immunization coverage in Nepal, Senegal, and Zambia from 2000 to 2019, based on KII and FGD data

	**Key for reading the table below:**
[red]	Not mentioned by key informants or community stakeholders who participated in this study
[green]	Implemented; participants noted that there was room for improvement (e.g., gaps or challenges)
[blue]	Implemented; participants described successful implementation (e.g., exceeds recommended guidelines)

**Category**	**Policy or intervention**	**Zambia**	**Nepal**	**Senegal**
**National level programming**<br>*Includes external partners and government*	Introduction of new vaccines	[blue]	[blue]	[blue]
	Vaccination as "priority" program	[green]	[blue]	[green]
	Strategic reallocation of targeted funds	[blue]	[blue]	[red]
	Health post expansion	[green]	[green]	[green]
	Constitution codifying health as a right	[red]	[blue]	[green]
	Forums for decision-making (e.g., ICC)	[blue]	[green]	[green]
	Media engagement	[blue]	[blue]	[blue]
**Sub-national programming**<br>*Regional and district level*	RED/REC implementation	[blue]	[green]	[blue]
	Training community health workers	[green]	[green]	[green]
	Meetings for evidence-based decisions	[green]	[green]	[blue]
	Data software, health post level	[red]	[red]	[green]
	Data software, district level	[green]	[green]	[green]
	Cold chain expansion	[blue]	[green]	[green]
**Community programming**<br>*Health post and village/community level*	Community health worker program	[blue]	[blue]	[blue]
	Community-level health committees	[blue]	[green]	[blue]
	Outreach services	[blue]	[blue]	[blue]
	Microplanning in health facilities	[blue]	[green]	[green]
	Engagement of community leaders	[blue]	[blue]	[blue]
	Support groups for caregivers	[green]	[blue]	[green]
	School outreach for health education	[green]	[green]	[green]
	Promotion through media (e.g., radio)	[blue]	[blue]	[blue]

For peer review only - http://bmjopen.bmj.com/site/about/guidelines.xhtml

4. Discussion

Notions of strengthening national governance structures have historically been recognized through country-level application of global guidelines to improve vaccine delivery and health systems; however, existing research lacks concrete descriptions of what this entails. This paper augments findings from existing literature by highlighting how structural and contextual factors – including governance, collaboration, the environment, and cultural norms - impact implementation decisions that have led to improvements in childhood vaccine delivery. We developed evidence-derived operational definitions of structures and processes that we identified as critical to the success of vaccine delivery systems in countries with high routine immunization coverage. We described *how* these processes were utilized in high-performing countries to drive catalytic improvements in coverage. Previous research has not explored the details or importance of governance, financing, health systems, or other contextual factors; our original framework based on preexisting literature (Figure 1) was missing complexity in these components, which is indicative of the limited knowledge in this area. In order to understand how global guidelines should be adapted to reflect local priorities and challenges, it was important for us to describe how national governance structures operate with the consideration of contextual factors. Ignoring a structured approach to understanding these factors could lead to repeated instances of sub-optimal program performance.

Our data points to the need for programmers and policy makers to better understand underlying governance structures. The structures and processes we examined in Nepal, Senegal, and Zambia supported adaptive decision-making based on context-specific needs and priorities. Adaptation at all levels enabled tailored and targeted programming based on evaluation of high-quality data. While health system improvements led by national governments were essential, our data also highlighted the importance of a deliberate and consistent focus on community engagement, which aligns with existing literature [1,27,28]. Community involvement in vaccine programming facilitated the government's understanding of cultural and social barriers and enablers which contributed to effective adaptation of interventions to increase coverage. Community ownership was fostered through national policies and resources for capacity building, engagement of traditional leaders, and the strength of the community health worker programs.

We found that many improvements in immunization coverage emerged from adjacent successes in health systems strengthening (HSS). While an unsurprising finding that health system strengthening would be an essential ingredient in support of vaccine system strengthening, it points to the need for a more integrated, rather than siloed, approach to child health. HSS has become a priority in recent decades as disruptions due to natural disasters, disease outbreaks, or civil unrest have exposed weaknesses in health systems [32, 33]. Although we focused on examining success factors for vaccine delivery, our data also suggests that there are still gaps and challenges in exemplar countries, including: 1) equitable access (e.g., lack of health posts in rural and remote areas); 2) long-term financing (e.g., most funding comes from external partners); 3) poor infrastructure (e.g., roads, electricity); and 4) a heavy reliance on volunteer community health workers which experienced vacancies and decreased morale in some cases.

In Nepal, Senegal, and Zambia, overall commitment to health and vaccines strengthened governance processes and supported vaccination efforts through prioritization of activities and resource allocation. Following the completion of GVAP in 2020, stakeholders reported that lack of consideration of existing structures and contextual factors limited the plan's success [34]. In addition, the monitoring and evaluation processes proposed through GVAP were often seen as unrealistic [9, 34, 35]. Suggested improvements included engaging with countries to accurately and sufficiently consider local context while setting global goals and targets and promoting country ownership of strategic planning [34, 35]. Our research revealed that national government ownership of strategic planning was critical to these countries' success, which aligns with the critical reflection of GVAP and other existing literature.

For peer review only - http://bmjopen.bmj.com/site/about/guidelines.xhtml

Overall, the strategies implemented by exemplar countries align with the WHO Comprehensive Framework of Strategies and Practices for Routine Immunization, which focus on managing the immunization program (e.g., political commitment, partnerships), mobilizing communities, generating demand, and monitoring progress [1]. Throughout the study, our analysis returned to the importance of defining and understanding the context, governance, financing, and health systems within a country, rather than focusing on any one intervention. This highlights the importance of further research into these structural factors. We examined how these strategies were operationalized to improve coverage in Nepal, Senegal, and Zambia, and how they can be applied in other settings. Understanding how governance processes function in high-performing countries may allow others to build stronger systems, adapt guidelines, and reflect on progress made in the field. Our research introduces areas of inquiry for further investigation, as the critical success factors that primarily emerged in all three exemplar countries are not the current focus within existing literature around determinants for vaccine coverage. as the critical success factors that primarily emerged in all three exemplar countries are not the current focus within existing literature around determinants for vaccine coverage.

5. Limitations

This study has several limitations. First, it was difficult to determine causation because we focused on countries that were successful in vaccine delivery but were unable to carry out a similar analysis in a country with lower vaccination coverage to compare. Second, the research tools focused on the factors that drove catalytic change and did not focus on interventions or policies that were unsuccessful. Third, using qualitative methods to understand historical events was challenging; interviewees often spoke about current experiences rather than discussing historical factors. However, we probed respondents to reflect on longitudinal changes in the immunization program. Additional country-specific limitations can be found elsewhere [15-17].

6. Conclusion

Through focusing on countries with high routine immunization coverage, we examined how vaccine delivery systems may leverage components of existing governance structures and health systems to accelerate and sustain coverage. Operational definitions for governance, health systems strengthening, community engagement, and adaptive capacity, along with descriptions of how these processes were implemented in high-performing countries, may help other countries implement similar improvements. Our findings highlight the need for program managers and policy makers to understand and consider the strengths and limitations of existing structures while adapting to the emergent needs and priorities relevant to the context. Our study looked retroactively on how and why countries succeeded in achieving high early childhood immunization coverage. The COVID-19 pandemic has highlighted the fragility in our health systems, and specifically how robust vaccine systems can rise to the challenge of emergent vaccination needs. While our findings are not immediately applicable to the current COVID vaccination needs, our underlying approach to understand context remain relevant to consider as countries strive to vaccinate their populations in the ongoing pandemic [36].

For peer review only - http://bmjopen.bmj.com/site/about/guidelines.xhtml

Acknowledgements:
We gratefully acknowledge the participants who gave their time and insights to help us better understand the vaccine delivery systems of Zambia, Nepal, and Senegal, along with facilitators from their respective Ministries of Health.

Contributions were also made by Sarah Chesemore, Anna Rapp, Tove Ryan, and Ethan Wong from the Bill & Melinda Gates Foundation; Kate Buellesbach, Nancy Fullman, Nathaniel Gerthe, Gloria Ikilezi, Caitlyn Mason, David Phillips, and Oliver Rothschild, Jordan-Tate Thomas, and Angela Wang from Gates Ventures; and the Vaccine Exemplars Research Advisory Group, including Agnes Binagwaho, Laura Craw, Carolina Danovaro, Anuradha Gupta, Heidi Larson, Penelope Masumbu, Kate O'Brien, Helen Rees, Lora Shimp, and Aaron Wallace.

More information about how this study addresses local research and policy priorities is included in our Reflexivity Statement.

Funding:
This work was supported by the Bill & Melinda Gates Foundation, Seattle, WA (OPP1195041) with a planning grant from Gates Ventures, LLC, Kirkland, WA.

Competing interests:
The authors declare that they have no known competing financial interests or personal relationships that could have appeared to influence the research reported in this paper.

Data sharing statement:
Data are not publicly available as all data are confidential. De-identified data may be available upon request.

Contributorship statement: KAH, ASE, SD, WK, MS, RAB, MCF: project conceptualization and methodology; KAH, ZS, ASE, KR, SD, WK, MS, RAB, MCF: investigation and data curation; KAH, ZS, ASE, KR, EAO, RAB, MCF: formal analysis; ZS, KAH: writing - original; KAH, ZS, ASE, EAO, KR, SD, WK, MS, RAB, MCF: writing – review and editing; all authors provided approval of the final version.

Ethics approval statement: This study was considered exempt by the Institutional Review Board committee of Emory University, Atlanta, Georgia, USA (IRB00111474); approved by the Nepal Health Research Council (NHRC; Reg. no. 347/2019) in Kathmandu, Nepal; the National Ethical Committee for Health Research (CERNS; Comité National d'Ethique pour la Recherche en Santé) in Dakar, Senegal (00000174); the University of Zambia Biomedical Research Ethics Committee (Federal Assurance No. FWA00000338, REF. No. 166-2019); and the National Health Research Authority in Zambia. All ethics committees abide by the principles of the Declaration of Helsinki; all participants provided written consent.

For peer review only - http://bmjopen.bmj.com/site/about/guidelines.xhtml

References

1. World Health Organization, *Immunization Agenda 2030: A Global Strategy to Leave No One Behind*. 2020.
2. Vanderslott, S. and T. Marks, *Charting mandatory childhood vaccination policies worldwide.* Vaccine, 2021.
3. Gavi the Vaccine Alliance. *Sustainable Development Goals*. 2020 18 February, 2020; Available from: https://www.gavi.org/our-alliance/global-health-development/sustainable-development-goals.
4. Piot, P., et al., *Immunization: vital progress, unfinished agenda.* Nature, 2019. **575**(7781): p. 119-129.
5. World Health Organization, *Global Vaccine Action Plan 2011–2020* 2013: USA.
6. World Health Organization and UNICEF, *Global Immunization Vision and Strategy 2006-2015*. 2005: New York, NY.
7. World Health Organization. *Immunization coverage*. 2020 July 15, 2020 August 4, 2020]; Available from: https://www.who.int/news-room/fact-sheets/detail/immunization-coverage.
8. World Health Organization and UNICEF, *WHO-UNICEF estimates of DTP3 coverage*. 2020.
9. World Health Organization, *GVAP review and lessons learned: methodology, analysis and results of the stakeholder consultations*, in *Annex to the Global Vaccine Action Plan 2011-2020.* 2019: Geneva.
10. Bednarczyk, R.A., et al., *Protocol: Identification and evaluation of critical factors in achieving high and sustained childhood immunization coverage in selected low- and lower-middle income countries.* BMJ Open, 2021. **12**(e058321).
11. Shen, A.K., R. Fields, and M. McQuestion, *The future of routine immunization in the developing world: challenges and opportunities.* Global health, science and practice, 2014. **2**(4): p. 381-394.
12. World Health Organization, *Global Routine Immunization Strategies and Practices (GRISP): a companion document to the Global Vaccine Action Plan (GVAP)*. 2016, Geneva: World Health Organization.
13. World Health Organization, *Reaching Every District (RED): A guide to increasing coverage and equity in all communities in the African Region*. 2017.
14. Phillips, D.E., et al., *Determinants of effective vaccine coverage in low and middle-income countries: a systematic review and interpretive synthesis.* BMC health services research, 2017. **17**(1): p. 681-681.
15. Rodriguez, K., et al., *Critical success factors for routine immunization performance: A case study of Zambia 2000 to 2018.* Vaccine X, 2021. **11**(4): p. 100166.
16. Sakas, Z., et al., *Critical success factors for high routine immunization performance: A case study of Senegal.* Vaccine X, 2023. **14**(100296).
17. Hester, K.A., et al., *Critical success factors for high routine immunization performance: A case study of Nepal.* Vaccine X, 2022(7781): p. 100214.
18. Noor, A.M., *Country ownership in global health.* PLOS Global Public Health, 2022. **2**(2): p. e0000113.
19. WHO and UNICEF, *Immunization, DPT (% of children ages 12-23 months)*, W.a. UNICEF, Editor. 2022.
20. Carter, A., et al., *A framework for identifying and learning from countries that demonstrated exemplary performance in improving health outcomes and systems.* BMJ global health, 2020. **5**(12): p. e002938.
21. Exemplars in Global Health. *Making Better Decisions in Global Health: Understand Positive Outliers to Inform Policy and Practice*. 2021; Available from: https://www.exemplars.health/.
22. LaFond, A., et al., *Drivers of routine immunization coverage improvement in Africa: findings from district-level case studies.* Health policy and planning, 2015. **30**(3): p. 298-308.
23. World Health Organization and UNICEF, *WHO-UNICEF estimates of national immunization coverage (WUENIC)*. 2020: Geneva, Switzerland.
24. *Nepal Demographic Health Survey 2001, 2006, 2011, 2016, 2017*, DHS Program, Editor.
25. Damschroder, L.J., et al., *Fostering implementation of health services research findings into practice: a consolidated framework for advancing implementation science.* Implementation science : IS, 2009. **4**: p. 50-50.

For peer review only - http://bmjopen.bmj.com/site/about/guidelines.xhtml

26. Pfadenhauer, L.M., et al., *Making sense of complexity in context and implementation: the Context and Implementation of Complex Interventions (CICI) framework.* Implementation science : IS, 2017. **12**(1): p. 21-21.
27. Hester K, *Open Sciences Framework (OSF) page: Assessing sustainability factors for rural sanitation coverage following the SSH4A approach.* *https://osf.io/caz9b/?view_only=a1f16382d78b445ab34cc2afedca0cb0.*
28. Castillo Zunino, F., et al., *Association of childhood vaccination with family planning, healthcare access, and women education: analysis of Nepal, Senegal and Zambia.* medRxiv, 2023.
29. Smalley, H.K., et al., *Factors associated with vaccine coverage improvements in Senegal.* medRxiv, 2023.
30. Sakas, Z., et al., *The role of Zambia's expansive ICC in supporting efficient, effective, and evidence-based vaccine and health sector programming.* medRxiv, 2021: p. 2021.11.29.21267021.
31. Hester, K.A., et al., *Critical interventions for demand generation in Zambia, Nepal, and Senegal with regards to the 5C psychological antecedents of vaccination.* medRxiv, 2022: p. 2022.04.25.22274035.
32. Ozawa, S., L. Paina, and M. Qiu, *Exploring pathways for building trust in vaccination and strengthening health system resilience.* BMC health services research, 2016. **16**(Suppl 7): p. 639-639.
33. World Health Organization Maximizing Positive Synergies Collaborative Group, *An assessment of interactions between global health initiatives and country health systems.* The Lancet, 2009. **373**(9681): p. 2137-2169.
34. Hwang, A., et al., *Global Vaccine Action Plan Lessons Learned II: Stakeholder Perspectives.* Vaccine, 2020. **38**(33): p. 5372-5378.
35. Cherian, T., et al., *Global Vaccine Action Plan lessons learned III: Monitoring and evaluation/accountability framework.* Vaccine, 2020. **38**(33): p. 5379-5383.
36. Dixit, S.M., et al., *Addressing disruptions in childhood routine immunisation services during the COVID-19 pandemic: perspectives from Nepal, Senegal and Liberia.* BMJ Global Health, 2021. **6**(7): p. e005031.

For peer review only - http://bmjopen.bmj.com/site/about/guidelines.xhtml

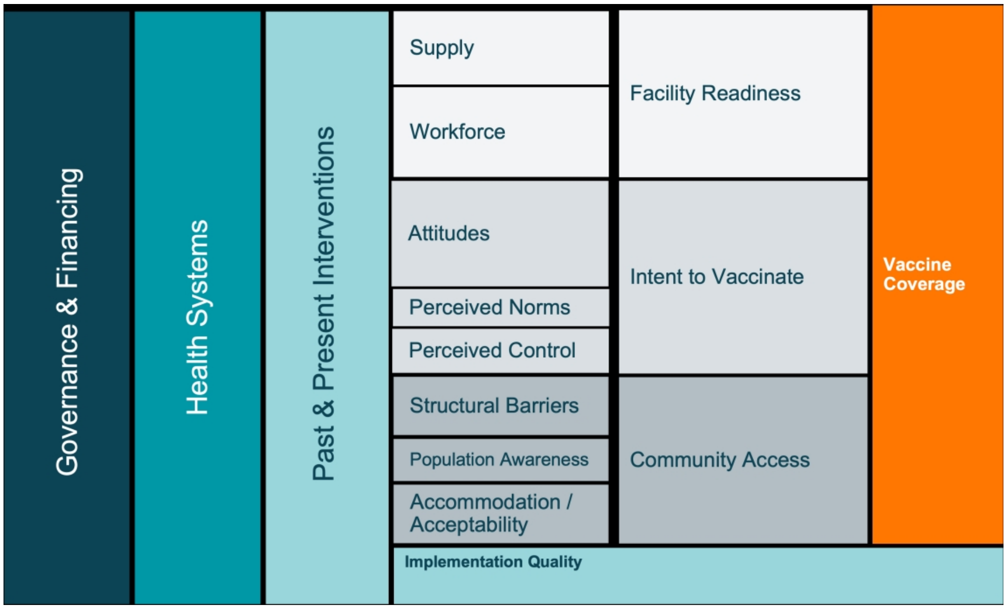

233x140mm (330 x 330 DPI)

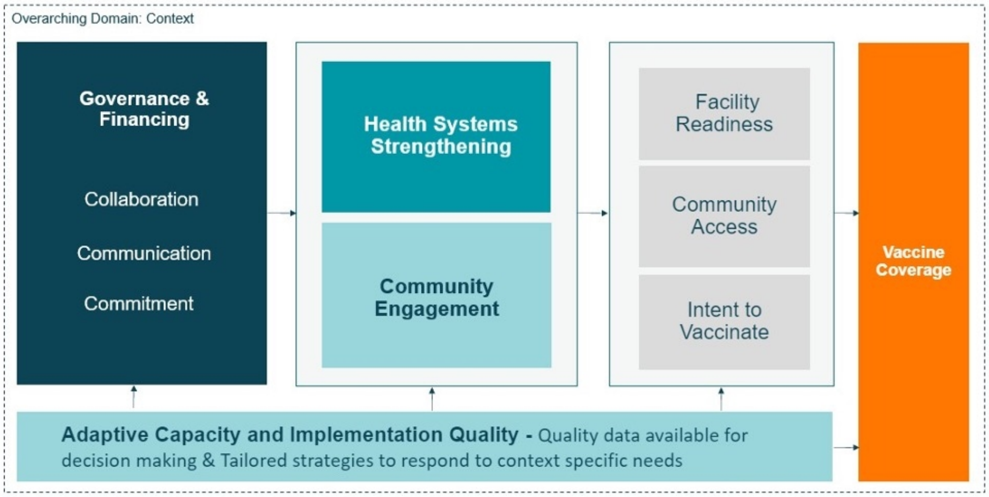

170x87mm (144 x 144 DPI)

Appendix 1: Coverage data for Nepal, Zambia, and Senegal

DHS DTP3 coverage in Zambia, by Province, 2000 – 2016

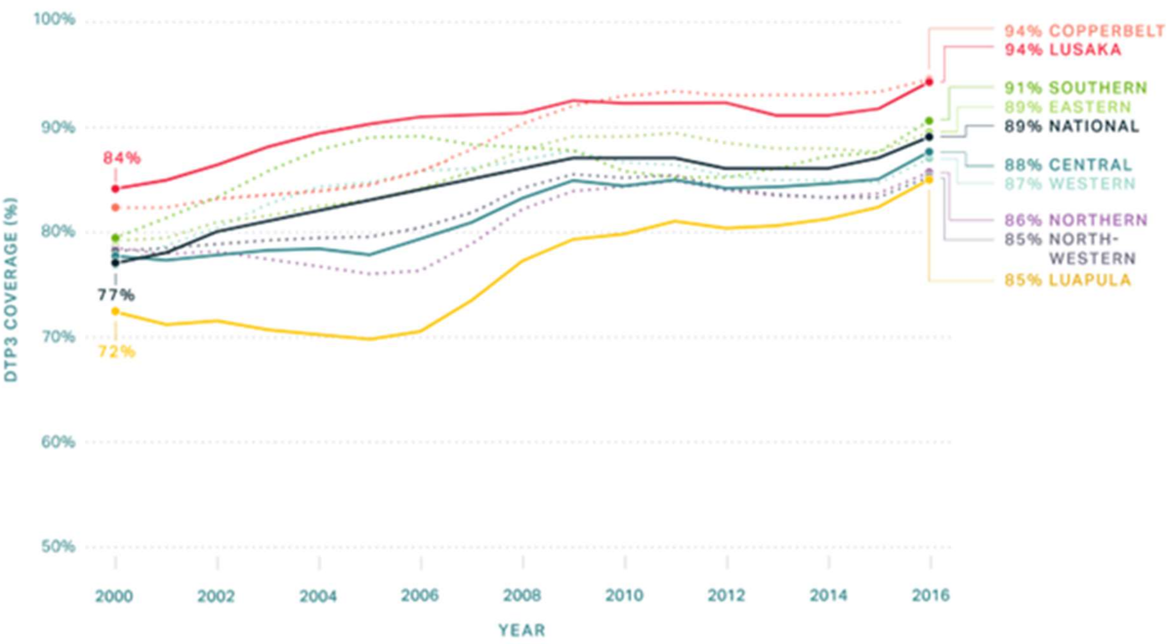

DHS DTP3 coverage in Nepal, by Province, 2000 – 2016

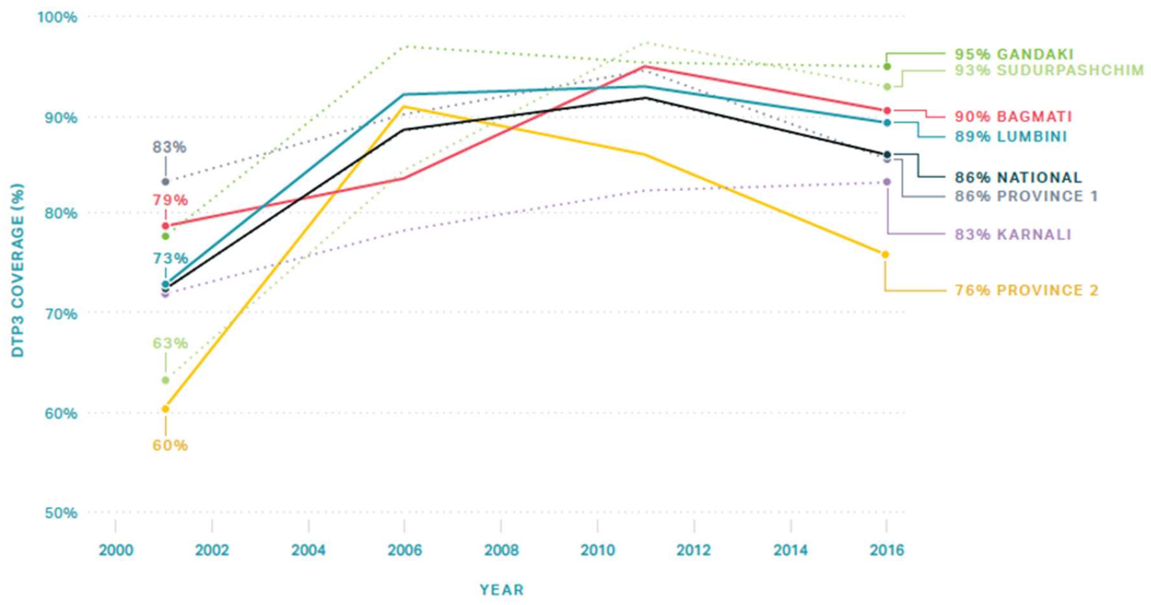

Data Source: Demographic and Health Survey (DHS)

DHS DTP3 coverage in Senegal, by Province, 2000 – 2016

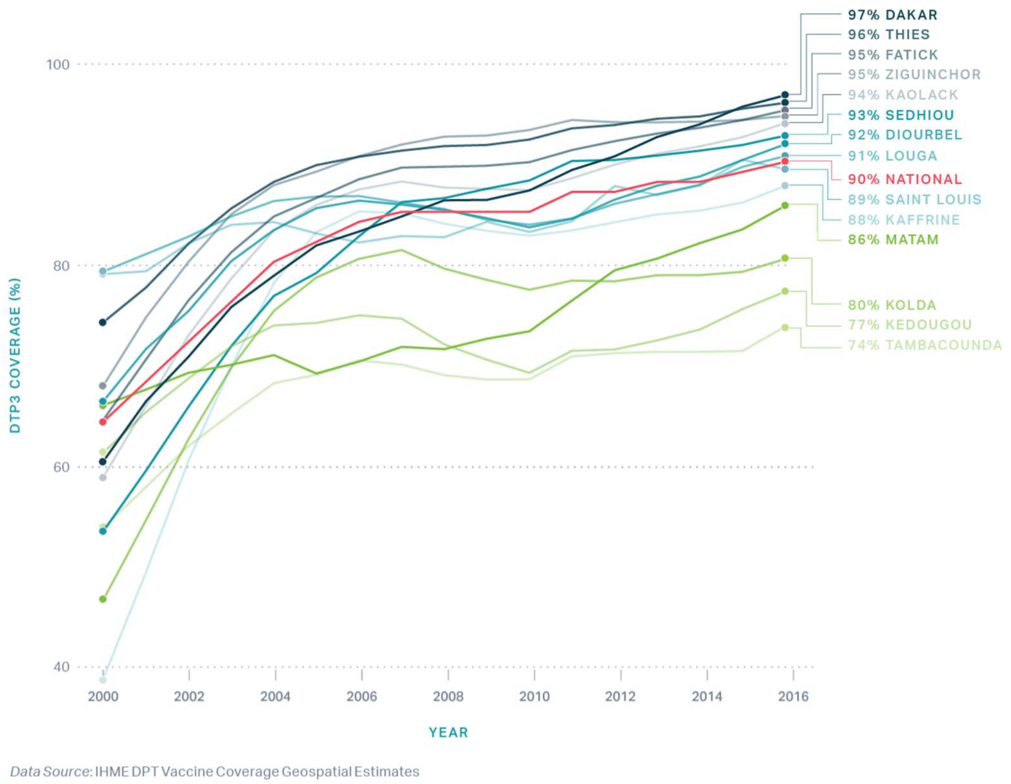

DTP3 coverage from 2000 to 2018 of Nepal, Senegal, and Zambia

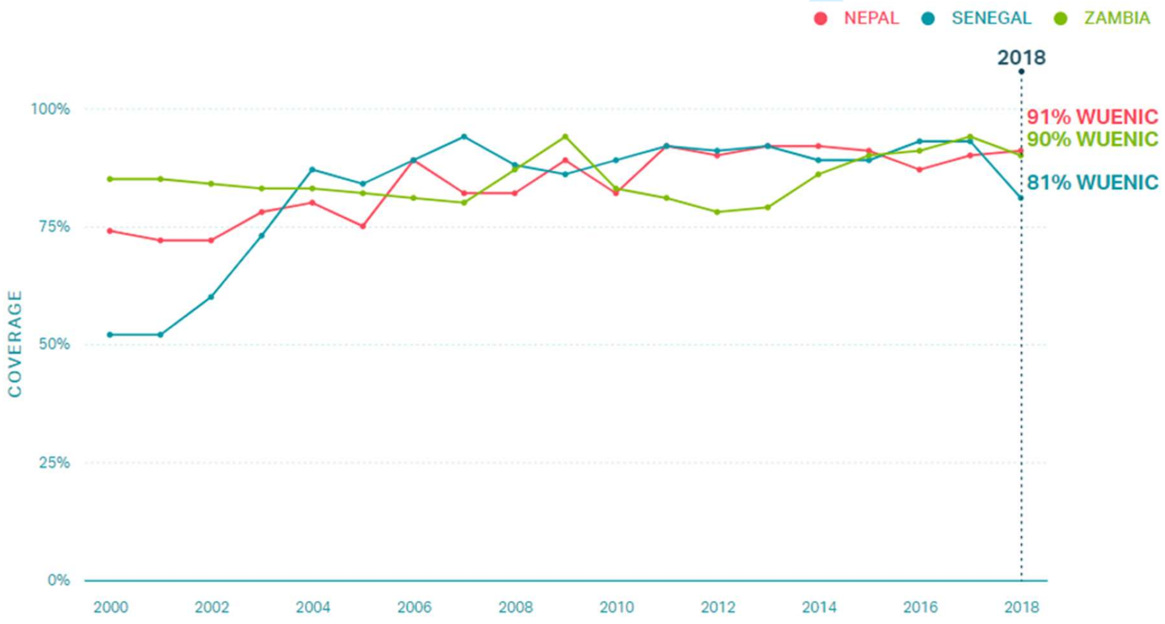

Date: ____________________________ *Interview Code:* ______ - ______ - ______

VACCINE EXEMPLARS

Key Informant Interview Guide – Tool 1b ALL COUNTRIES

Objectives:

- To understand the vaccine intervention characteristics during country-specific change points in DTP1/DTP3 coverage
- To identify key internal and external actors, stakeholders, and partnerships in the immunization program both now and in the past
- To identify the strategies behind current immunization programs to inform current context
- To identify previous immunization activities that were key during change points in the DTP1/DTP3 curve
- To understand key barriers and facilitators of interventions in the immunization sector

**PART I – DEMOGRAPHIC AND BASIC INFORMATION**<br>*Can be asked by interviewer or filled out by note taker.*	
**A01. Researcher/Interviewer name:**	
**A01b. Note taker(s) name(s):**	
**A01c. Translator name:**	
**A01d. Copy belongs to:**	
**A02. Consent given:** Y / N<br>Time of consent ____ : ____ am/pm	
**A03.** Date (dd/mm/yy): _____ /_____ / _____	**A04a.** Interview start time ____ : ____ am/pm<br>**A04b.** Interview end time ____ : ____ am/pm<br>**A05**. Recorder(s) used:
**A06.** Name of Respondent interviewed:	**A07.** Designation/title of Respondent interviewed:
**A08.** Organization name:	**A09.** Years in Organization:

For peer review only - http://bmjopen.bmj.com/site/about/guidelines.xhtml

Date: ___________________________ *Interview Code:* ______ - ______ - ______

PART II – INTERVIEW INTRODUCTION

<u>Interviewer Script</u>: Thank you very much for your willingness to meet with us today and share your experience and knowledge regarding the immunization program in ________ (*COUNTRY*). I'd like to begin by learning about you and your role related to the immunization program.

1. Can you tell me about your role in ________ (*ORGANIZATION*)?

~ Individual Setting ~

Follow up:

- *How does your role relate to the immunization program?*
- *Responsibilities (past and present)?*
- *How long have you been working on vaccines/with the immunization program(s)?*
- *What drew your interest to vaccines/immunization programs? What type of projects have you worked on?*

PART IIa – IMMUNIZATION PROGRAM

Read the following script aloud:

<u>Interviewer Script</u>: Now I'd like to learn about the immunization program, and about the increase in coverage since the year 2000. I'd also like to know about what relevant events that may have contributed to this change, and how the immunization program is funded.

2. Will you tell me a bit about the current state of the vaccination program in ________ (*COUNTRY*). ?

~ Outer Setting ~

Follow up:

- *Who are the main decision makers at this point in time?*

3. Who are the main government actors involved in the current immunization program?

~ Outer Setting ~
~ Engaging; Process ~
~ RQ2. Political Will ~
~ RQ5. Knowledge and Resource Sharing ~
~RQ6. Workforce ~

Follow up:

- *If there were multiple governmental agencies, how were they related and what responsibility does each take in regard to vaccination efforts?*
- *What other activities did governmental agencies engaged in vaccination undertake?*
- *Which organizations or ministries are responsible for how the immunization system performs from top to bottom, inclusive of the regional level?*

For peer review only - http://bmjopen.bmj.com/site/about/guidelines.xhtml

Date: ___________________________ *Interview Code:* ______ - ______ - ______

4. How does the chain of command/structure within the immunization sector work?
Follow up:<br>• *What kind of directives come from the ministry?*<br>• *How do you report changes that are made in programming?*<br>• *Who do you report this to?*<br>• *What kind of changes in programming do people at different levels (national, provincial, district, health facility) contribute to?*<br>• *How are you made aware of changes in personnel within the immunization program?*<br>• *Who within the program reports to you?*<br>• *Who do you report to?*
5. Who are the main partner organizations involved in the current immunization program?<br>***(NOTE: DON'T EXPECT TOO MUCH FOLLOW UP FOR G, H, I)***<br>***~ Outer Setting ~***<br>***~ Engaging; Process ~***<br>*~ RQ5. Knowledge and Resource Sharing ~*<br>*~RQ6. Workforce ~*
Follow up:<br>• *What are their roles?*<br>• *What are the local donor, individuals, and local NGOs that contribute to the sector?*<br>• *Who makes up the internal and external-workgroups? Can you define these actors - Ministries, INGOs, local NGOs, private sector?*<br>• *How do the organizations/stakeholders (individual actors) coordinate with each other?*
6. When do you think the vaccination program started to show improvements?<br>***~ Outer Setting ~***<br>***~ Inner Setting~***
Follow up:<br>• *What happened in* ______ (*YEAR(S)*) *to affect vaccine coverage?*<br>• *Why do you think this is when the program improved? What are the events?*<br>• *How would you describe the environment surrounding the vaccine program during this period? How did this make the change possible?*<br>• *What do you think catalyzed this change? (If examples needed, use beneficiary needs, policy, legislation, individual champions, politicians?*<br>• *Who were the main decision makers at this point in time? What were their incentives for performing well? Which party was in power at this time?*<br>• *Do other individuals agree with this assessment, or is there something else that is believed to be behind change? For example, do politicians think this is also the driver behind the increase in coverage?*<br>• *Which, of the reasons you gave me, do you consider the most important in improving immunization coverage? Why?*
7. ***(NOTE: ONLY SHOW/THIS to CONNECT TO PREVIOUS ANSWER)*** This graph shows the DTP1/DTP3 coverage trend since 2000 in ______ (*COUNTRY*). In addition to what you've already said, what else can you tell us about these time points where coverage increased? What important or relevant events do you believe contributed to the increases?
8. Who were the key stakeholders during the time of improvement?

For peer review only - http://bmjopen.bmj.com/site/about/guidelines.xhtml

Date: ____________________________ *Interview Code:* ______ - ______ - ______

~ Outer Setting ~
~ RQ2. Political Will ~
~ RQ5. Knowledge and Resource Sharing ~
~ RQ6. Workforce ~

Follow up:

- *Ministries, partner organizations?*
- *What role did they play?*
- *If there were multiple governmental agencies, how were they related and what responsibility does each take in regard to vaccination efforts?Who made up the internal and external-workgroups?*
  - *Can you define these actors* **-** *Ministries, INGOs, local NGOs, private sector?*
- *What other activities did governmental agencies engaged in vaccination undertake?*
- *What has the turnover in these positions been like?*
- *Have the key people identified been in the same position for extended periods? What was the mandated term?*
  - *What are their motivations in involvement?*
  - *Can you connect me to XXX?*

-

9. What do you consider to be the main barriers to the improvement in immunization coverage in ______ (*YEAR(S)*) before the increase?

~ Outer Setting ~

Follow up:

- *How was each barrier addressed?* ***(NOTE: REFER BACK TO SPECIFIC BARRIERS MENTIONED BY PARTICIPANT)***
- *What was the motivation behind addressing the barriers?*

10. What were the main sources of funding for immunization programming during this time period? ***(NOTE: FOR TOOLS H and I, JUST ASK THE MAIN QUESTION. DON'T EXPECT TO PROBE)***

~ Available Resources; Inner Setting ~
~ RQ2. Political Will ~

For peer review only - http://bmjopen.bmj.com/site/about/guidelines.xhtml

Date: ___________________________ *Interview Code: ______ - ______ - ______*

Follow up:

Ask these questions at each level -

- *Is there a fund specifically for vaccines, or is there just a general health fund?*
    - *How are these funds spent?*
- *How much of the overall health budget is allocated to vaccination activities?*
- *Is there an overall vaccine budget, or is it separated by vaccine?*
- *Are there financial barriers to increasing the vaccination rates? If so, what?*
    - *Which specific barrier(s) require more funding?*
- *Which level of government is responsible for spending the resources* ***? (EXAMPLES: Province, district, health facility)***
    - *How much power do you have to spend the money the way that you want to?*
- *Is there an advisory board/audit that checks how money is spent? Does the advisory board check the expenses for each level; regional, district, health center?*
- *Are there any restrictions on how contracts are assigned?* ***(EXAMPLES: Grants, distributors for supplies or vaccine, etc.)***
- *Can you share supporting documentation with me? If not, where can I access this, and do you know who may be able to assist me?*

For peer review only

For peer review only - http://bmjopen.bmj.com/site/about/guidelines.xhtml

Date: ___________________________ *Interview Code:* ______ - ______ - ______

PART III – INTERVIEWER TO ASK SECTIONS BELOW AS DETERMINED BY TITLE/DEPARTMENT OF RESPONDENT:

Central -

A. Minister of Health, Minister Representative, or General Secretary …………………...….…. 7
B. Ministry Departments of Epidemiology, Surveillance, EPI, Vaccines, Family Health…….. 10
C. Ministry Departments (or liaisons) of Planning, Statistics, Research, Human Resources, Communication and Development …………………………………………………….… .20
D. Ministry Departments (or liaisons) of Infrastructure, Equipment, Finance ……………...…. 24
E. Partner Organizations, such as WHO, UNICEF, CDC, USAID etc.; NITAG, Professional Organization members ……………………………………………………...………….. 26

Subnational -

G. Region Head, Surveillance, Prevention, and Vaccination Personnel ……………….….… 30
H. District Head, Surveillance, Prevention, Vaccination Personnel ………………………...….38
I. Clinic Head ……………………………………………………………………….….46
J. Community Leader……………………………………………………………………53

PART IV – CONCLUSION ……………………………………………………………………57

For peer review only

For peer review only - http://bmjopen.bmj.com/site/about/guidelines.xhtml

Date: ___________________________ *Interview Code:* ______ - ______ - ______

**SECTION A.** MINISTER OF HEALTH, MINISTER REPRESENTATIVE, OR GENERAL SECRETARY<br><br>**Objectives:**<br>• To understand the vaccine intervention characteristics during country-specific change points in DTP1/DTP3 coverage<br>• To identify key internal and external actors, stakeholders, and partnerships in the immunization program both now and in the past<br>• To explore decision-making process in selecting the strategies and activities in the immunization program that created change points in DTP1/DTP3 coverage<br>• To identify the strategies behind current immunization programs to inform current context<br>• To identify previous immunization activities that were key during change points in the DTP1/DTP3 curve<br>• To understand key barriers and facilitators of interventions in the immunization sector
**PART A1 – POLICY**
1. What is the current state of legislation regarding vaccines and immunizations (laws, constitutional requirements)?<br><br>***~ External Policies and Incentives; Outer Setting ~***<br>*~ RQ2. Political Will ~*
Follow up:<br><br>• *Where would I find copies/documentation of that legislation?*<br>• ***If there is no knowledge of current legislation:*** *Is there past legislation that is being followed?*
2. How was legislation on vaccines and immunization developed?<br><br>***~ External Policies and Incentives; Outer Setting ~***<br>*~ RQ2. Political Will ~*
NOTE: BASE QUESTION ON THE PREVIOUS ANSWER.
Follow up:<br><br>• *When was key legislation passed?*<br>• *Who played a role in this (organization or person)?*
3. What national policies do you think were important in increasing vaccine coverage?<br><br>***~ External Policies and Incentives; Outer Setting ~***<br>*~ RQ2. Political Will ~*<br>*~ RQ7. Vaccine Policy and Policy Enforcement ~*
Follow up:<br>• *Which do you think has been the most important piece of legislation in contributing to the current rates of immunization coverage?*<br>• *What rule/regulation did you find to be most important/effective in increasing vaccine coverage?*<br>• *What role does the Ministry of Finance have in determining these rules and regulations?*<br>• *What role does the Ministry of Finance have in implementing the rules and regulations?*<br>• *Which supporting documents could you share with me?*
**PART A2 – STAKEHOLDERS**
4. How have financial investment decisions regarding the immunization sector been made?<br>***~ Outer Setting ~***<br>*~ RQ1b. Immunization System Context Within the Health Sector ~*

For peer review only - http://bmjopen.bmj.com/site/about/guidelines.xhtml

Date: ___________________________ *Interview Code: ______ - ______ - ______*

<table>
<tr><td>Follow up:
How is this relative to other areas of the healthcare system/sector?
Who determines this?
What percentage/amount of funding is spent on vaccinations as opposed to other health sector areas, including disease surveillance, coverage measurement, accountability?
(NOTE: Some probes may have been addressed in Part 2. Make sure to follow up on relevant information but don't repeat questions)</td></tr>
<tr><td>PART A3 – IMMUNIZATION PROGRAM</td></tr>
<tr><td>5. What were key activities in <u>planning</u> changes for the immunization program? (NOTE: Refer to answers in section 2).

~ Planning; Process ~
~ RQ5. Knowledge and Resource Sharing ~
~ RQ6. Workforce ~</td></tr>
<tr><td>Follow up:
Meeting, working group
Did you have an action plan?
A theory of change or log frame?</td></tr>
<tr><td>6. What challenges did you face in the early planning phases?

~ Planning; Process ~</td></tr>
<tr><td>Follow up:
How were these challenges overcome (EX: Internal to agency/external to agency)?</td></tr>
<tr><td>7. What kind of evidence or information (EX: outbreaks, surveillance data) informed changes in programming or interventions?

~ Planning; Process ~
~ RQ5. Knowledge and Resource Sharing ~</td></tr>
<tr><td>Follow up:
You mentioned ______________ (DATA SOURCE NAME(S)). What role did this information have in the push to increase vaccine coverage?
Can you explain how ______________ (DATA SOURCE NAME(S)) is collected?
How is this ______________ (DATA SOURCE NAME(S)) integrated into other health systems?
[If surveillance system not mentioned] Does ______________ (COUNTRY) have a surveillance system?
~ Planning; Process~
~ RQ5. Knowledge and Resource Sharing~</td></tr>
<tr><td>8. What areas of the country have seen the greatest improvement in vaccine rates?

~ Reflecting and Evaluating; Process ~
~ RQ3. Information Systems ~
~ RQ4. Intent/Demand for Vaccines ~</td></tr>
</table>

For peer review only

For peer review only - http://bmjopen.bmj.com/site/about/guidelines.xhtml

Date: ____________________________ *Interview Code:* ______ - ______ - ______

Follow up:<br>• *Why do you think these areas had the greatest improvement?*<br>○ *Was a project/intervention specifically tailored to this location?*<br>○ *Was it targeted as a critical area for change?*<br>• *What areas saw the quickest improvement?*<br>○ *Was a project/intervention specifically tailored to this location?*<br>○ *Was it targeted as a critical area for change?*
9. What areas of the country did not see significant changes?<br>***~ Reflecting and Evaluating; Process ~***<br>*~ RQ3. Information Systems ~*<br>*~ RQ4. Intent/Demand for Vaccines ~*
Follow up:<br>• *Why do you think this is?* (**NOTE: May have already had high coverage, may not relate to challenges)**<br>• *What were the challenges?*<br>○ *Why?*<br>○ *Was a project/intervention specifically tailored to this location?*<br>▪ *Implementation failure*<br>▪ *Theory failure*<br>○ *Was it targeted as a critical area for change?*<br>• *What has been/might be done to mitigate this?*
10. What are the greatest barriers to sustained performance?<br>***~ Reflecting and Evaluating; Process~***
11. What are the perceptions of corruption in the public sector?
Follow up:<br>• *Are you aware of any anti-corruption measures that have been put in places?*<br>• *What kind of accountability measures are in place that would prevent corruption?*<br>• *Perceptions of quality of electoral processes?*<br>• *Judicial institutions?*<br>• *Anti-corruption efforts?*

For peer review only

For peer review only - http://bmjopen.bmj.com/site/about/guidelines.xhtml

Date: ___________________________ *Interview Code:* ______ - ______ - ______

SECTION B. MINISTRY DEPARTMENTS OF EPIDEMIOLOGY, SURVEILLANCE, EPI, VACCINES, FAMILY HEALTH, GAVI REPRESENTATIVE

Objectives:

- To understand the vaccine intervention characteristics during country-specific change points in DTP1/DTP3 coverage
- To identify key internal and external actors, stakeholders, and partnerships in the immunization program both now and in the past
- To explore decision-making process in selecting the strategies and activities in the immunization program that created change points in DTP1/DTP3 coverage
- To identify the strategies behind current immunization programs to inform current context
- To identify previous immunization activities that were key during change points in the DTP1/DTP3 curve
- To understand key barriers and facilitators of interventions in the immunization sector

PART B1 – POLICY

1. What is the current state of legislation regarding vaccines and immunization?

~ External Policies; Outer Setting ~
~ RQ2. Political Will ~

NOTE: EXAMPLES INCLUDE LAWS, CONSTITUTIONAL REQUIRMENTS

Follow up:

- *Where would I find copies/documentation of that legislation?*

2. How was legislation on vaccines and immunization developed? What year was it started?

~ External Policies; Outer Setting ~
~ RQ2. Political Will ~

Follow up:

- *When was key legislation passed?*
- *Which do you think has been the most important piece of legislation in contributing to the current rates of DTP3 coverage?*
- *Where would I find copies/documentation of that legislation?*

3. What policies were made at the national level that you think were important in increasing vaccine coverage during $\overline{}_{YEAR(S)}$?

~ External Policies; Outer Setting ~
~ RQ2. Political Will ~
~ RQ7. Vaccine Policy and Policy Enforcement ~

Follow up:

- *How were these policies informed?*
- *What was the reason/rationale for creating these new policies?*
- *What is the policy making process, and how are new policies or recommendations disseminated and implemented once approved?*
- *How do they work in both vertical (e.g. across ministries) and horizontal (e.g. within different levels of a particular ministry)?*
- *What led to the selection of these policies?*

For peer review only - http://bmjopen.bmj.com/site/about/guidelines.xhtml

Date: ___________________________ *Interview Code: ______ - ______ - ______*

• *What challenges did you encounter with any new policies made?*
4. Are there relevant polices at the subnational/regional level? If so, what?<br><br>***~ External Policies; Outer Setting ~***<br>*~ RQ2. Political Will ~*<br>*~ RQ7. Vaccine Policy and Policy Enforcement ~*
Follow up:<br><br>• *If there are no policies at the subnational/regional level, why?*<br>○ *Do all regions need to follow the national law, or can they create their own?*<br>• *How were these policies informed?*<br>• *What led to the selection of these policies?*<br>• *What data were most important in setting policy?*<br>• *What kind of global/regional resources were utilized?*<br>• *How were policies implemented?*<br>•
**PART B2 – STAKEHOLDERS**
5. How have decisions on types of financial investments in the immunization sector been made, relative to other areas of the healthcare system/sector?<br><br>***~ Outer Setting ~***<br>*~ RQ1b. Immunization System Context Within the Health Sector ~*
Follow up:<br><br>• *Who determines this?*<br>• *What percentage/amount of funding is spent on vaccinations as opposed to other health sector areas, including disease surveillance, coverage measurement, accountability?*
**PART B3 – IMMUNIZATION PROGRAM**
6. What sort of intervention(s) do you attribute to the increase in immunization coverage?<br>***~ Planning; Process ~***<br>*~ RQ4. Intent/Demand for Vaccines ~*<br>*~ RQ5. Knowledge and Resource Sharing ~*<br>*~ RQ6. Workforce ~*
Follow up:<br><br>• *Prior to the intervention, what kind of piloting or testing of the intervention occurred?*<br>• *Did the service delivery of vaccinations change after the intervention? How?*<br>• *Where did this take place nationally?*<br>• *What was learned in this process?*

For peer review only - http://bmjopen.bmj.com/site/about/guidelines.xhtml

Date: ___________________________ *Interview Code:* ______ - ______ - ______

7. What were key activities in planning changes for the program?

~ Planning; Process ~
~ RQ5. Knowledge and Resource Sharing ~
~ RQ6. Workforce ~

Follow up:

- *What was your role?*
-
- *Did you have an action plan?*
- *A theory of change or log frame?*

8. What challenges did you face in the early planning phases?

~ Planning; Process ~

Follow up:

- *How were these challenges overcome* ***(EX: Internal to agency/external to agency)****?*

9. What were key training activities of [intervention/activities mentioned in Q6]?

~ Planning & Executing; Process ~
~ RQ5. Knowledge and Resource Sharing ~
~ RQ6. Workforce ~

Follow up:
Do you have the following information, and can we access it?

- *Who was involved in trainings* ***(NOTE: at all levels)****?*
- *Who taught the training?*
- *How were they selected*
  - *Trainers*
  - *Trainees?*
- *What was their motivation/incentive for involvement?*
- *How were the trainings conducted?*
- *Were individuals participating in the training given opportunities to provide feedback?*
  - *If so, how?*
  - *If not, why?*
- *If so, did this change the implementation of the program in the region*?

10. If no training activities, what modality did the intervention have?

Follow up:

- *What modalities were used for different aspects of the project, generally?*

(NOTE: FOLLOW UP IF THE INTERVENTION SEEMS UNCLEAR, E.G. IS MORE FUNDING AVAILABLE? LOW TURNOVER OF STAFF? ETC.)

11. What kind of evidence/information (***EX: outbreaks, surveillance data***) did you use to inform changes/interventions?

~ Planning; Process ~
~ RQ5. Knowledge and Resource Sharing ~

For peer review only - http://bmjopen.bmj.com/site/about/guidelines.xhtml

Date: ___________________________ *Interview Code: ______ - ______ - ______*

Follow up:<br>• *You mentioned ___________ (DATA SOURCE NAME(S)). What role did this information have in the push to increase vaccine coverage?*<br>• *Can you explain how ___________ (DATA SOURCE NAME(S)) is collected?*<br>• *How is this ___________ (DATA SOURCE NAME(S)) integrated into other health systems?*<br>• *[IF SURVEILLANCE SYSTEM NOT MENTIONED] How does the surveillance system in ___________ (COUNTRY) work?*<br>***~ Planning; Process ~***<br>*~ RQ5. Knowledge and Resource Sharing~*
12. What measures did you find critical for success when implementing interventions?<br>***~ Reflecting and Evaluating; Process ~***<br>*~ Q6. Workforce ~*
Follow up:<br>• *What advice would you give to another person/region/country who was going to implement a similar program/project to yours?*
13. What challenges/difficulties did you encounter when implementing interventions?<br>***~ Reflecting and Evaluating; Process ~***
Follow up:<br>• *How were these challenges overcome?*
14. What were the greatest challenges in project implementation?<br>***~ Reflecting and Evaluating; Process ~***
Follow up:<br>• *Of those challenges you mentioned, which was the greatest?*<br>• *Was the project specifically tailored to this location?*<br>• *Was it targeted as a critical area for change?*<br>• *How were decisions made about what to do?*<br>• *Who contributed to this decision-making? Why?*
15. What areas of the country have seen the greatest improvement in vaccine coverage that you would attribute to this intervention?<br>***~ Evaluation; Process~***<br>*~ RQ3. Information Systems ~*<br>*~ RQ4. Intent/Demand for Vaccines ~*
Follow up:<br>• *Was the project specifically tailored to this location?*<br>• *Was it targeted as a critical area for change?*<br>• *What areas saw the quickest improvement?*<br>• *Why do you think these areas had the greatest improvement?*
16. During the improvement period, did you recommend any new vaccine introductions ***(EX: PCV, rotavirus)***?<br>***~ Planning; Process ~***

For peer review only

For peer review only - http://bmjopen.bmj.com/site/about/guidelines.xhtml

Date: ____________________________ *Interview Code:* ______ - ______ - ______

~ RQ4. Intent/Demand for Vaccines ~<br>*~ RQ5. Knowledge and Resource Sharing ~*<br>*~ RQ6. Workforce ~*
Follow up:<br>• *How did they affect the overall delivery system?*
•
17. What areas did not experience significant change in coverage after implementation of the intervention?<br>***~ Evaluation; Process~***<br>*~ RQ3. Information Systems ~*<br>*~ RQ4. Intent/Demand for Vaccines ~*
Follow up:<br>• *Why do you think this is?* ***(EX: May have already had high coverage, may not relate to challenges)***<br>• *What were the challenges? Why?*<br>• *What has been/might be done to mitigate this?*
18. What populations are currently the most vulnerable to low coverage?<br>***~ Evaluation; Process~***
19. What populations historically were the most vulnerable to low coverage?<br>***~ Evaluation; Process~***
Follow up:<br>• *How did you identify these populations as vulnerable?*<br>○ *Both previously and now*<br>• *How did you reach out to these populations?*<br>• *What strategy/strategies do you use to reduce inequities?*<br>• *What strategy did you previously use to reduce inequities?*<br>• *What kind of data supports this?*<br>• *Is this data you can share with me?*

For peer review only - http://bmjopen.bmj.com/site/about/guidelines.xhtml

Date: ___________________________ *Interview Code:* ______ - ______ - ______

•
20. What interventions were offered through community health workers (CHWs)?<br><br>***~ Executing; Process ~***<br>*~ RQ6. Workforce ~*
Follow up:<br><br>• *How were/are CHWs motivated/incentivized for involvement?*<br>○ *Financial*<br>○ *Respect*<br>○ *Opportunities for learning/capacity building*<br>○ *Recruited by trusted person*<br>▪ *Unable to say*<br>• *What kind of trainings are offered to:*<br>○ *Increase learning?*<br>○ *Build capacity*<br>• *What are the qualifications to be a CHW?*<br>• *Are their responsibilities increased over time?*<br>• *How do they prioritize responsibilities?*<br>• *How do CHWs become better at what they do over time?*
21. What interventions were offered at various levels of the health system? To providers?<br><br>***~ Execution; Process ~***<br>*~ RQ3. Information Systems ~*<br>*~ RQ6. Workforce ~*
Follow up:<br><br>• *Province/district/health post*<br>• *How were these developed?*<br>• *How were they implemented?*<br>○ *Training?*<br>• *Why were they implemented in the way that they were?*<br>• *Who oversaw these interventions?*<br>• *What led to the selection of these interventions?*<br>• *Was there variation in different geographic regions etc. If so, why?*
•
•
22. What programs were offered to parents or first-time parents?<br>***~ Intervention Characteristics~***<br>***~Outer Setting ~***<br>*~ RQ4. Intent/Demand for Vaccines ~*
Follow up:<br><br>• *Were these tailored to different areas/cultures/religions/languages/etc.?*

For peer review only - http://bmjopen.bmj.com/site/about/guidelines.xhtml

Date: ___________________________ *Interview Code: ______ - ______ - ______*

<table>
<tr><td>

- *How were these targeted specifically to mothers, fathers or alternative caretakers?*
- *How were they developed?*
- *Where did you get the idea for these interventions?*
  - *Did you use a framework or intervention to inform your intervention?*
- *How were they delivered?*
- *Who was engaged in delivery?*
- *What determined their involvement?*
- *What kinds of modalities were used?*
- *What kind of communication materials (if any) were utilized?*
  - *Mass media?*
  - *Dissemination through health centers?*
  - *Other?*
- *How did you get individuals to participate in this program?*
- *What led to their selection?*
- *Could you share any materials with us?*

**IF PARTICIPANT IS UNABLE TO ANSWER, NOT NECESSARY TO GO THROUGH EACH PROBE.*

~ Beneficiary needs and resources ~
</td></tr>
<tr><td>

23. Were the programs used in combination with other health interventions?

~ Outer Setting ~
~ RQ4. Intent/Demand for Vaccines ~
</td></tr>
<tr><td>

Follow up:

- *Were there any communication materials, strategies, or campaigns in conjunction with the other healthcare interventions?*
- *Or communications (IEC materials, mass media, etc.)?*
- *How was this coordinated?*
- *Who took ultimate responsibility for outcomes?*
</td></tr>
<tr><td>

24. Did you make any changes to the supply chain in ______________________
YEAR(S) or during specific interventions mentioned

~ Execution; Process ~
~ Q6. Workforce ~
</td></tr>
<tr><td>

Follow up:

- *How were these changes made?*
- *Why was this change made?*
  - *Has this change had the anticipated impact (on coverage or otherwise)?*
</td></tr>
<tr><td>

25. Were there any changes made to the data systems in ______________________ ?
YEAR(S)or specific intervetions mentioned

~ Execution; Process ~
~ Q3. Information Systems ~
~ Q6. Workforce ~
</td></tr>
</table>

For peer review only - http://bmjopen.bmj.com/site/about/guidelines.xhtml

Date: ____________________________ *Interview Code: ______ - ______ - ______*

Follow up:

- *How were these changes made?*
- *Why was this change made?*
  - *Has this change had the anticipated impact (on coverage or otherwise)?*
- *Did you develop or already have in place a clinic-based immunization record keeping system to track immunizations administered to individuals?*
- *Who kept these records? Did parents hold records?*
- *What are parents' involvement in record keeping for their child's vaccinations?*
- *To what extent was technology a component of record keeping (computer, phone data collection)?*

26. During those years, did you have any kind of reminder/recall system for children's vaccinations?

~ Execution; Process~
~ Q3. Information Systems ~
~ Q6. Workforce ~

NOTE: REMINDER SYSTEM DEFINED AS A MESSAGE SENT BEFORE APPOINTMENTS TO REMIND PARENTS TO COME; RECALL SYSTEM DEFINED AS A NOTICE SENT TO PARENTS WHO FAIL TO KEEP THEIR APPOINTMENTS.

Follow up:

- *If so, how was this implemented?*
- *What kind of system was this?*
  - *Cell phone text reminders?*
  - *Community health worker in-person reminders?*
  - *Church/religious institution reminders?*
- *Was technology a component of this system?*
- *Who carries out the reminders or recall?*
- *Were there interventions offered through the CHWs?*

For peer review only - http://bmjopen.bmj.com/site/about/guidelines.xhtml

Date: ___________________________ *Interview Code:* ______ - ______ - ______

-

-

27. How do you monitor the performance of immunization programs?

~ Evaluation; Process~
~ RQ3. Information Systems ~

28. How does this differ from how immunization programs were monitored in the past?

~ Evaluation; Process ~
~ RQ3. Information Systems ~

Follow up:

- *Who is responsible for collecting data related to measures?*
- *How is data used?*
- *What measures are included?*
- *How often is data collected/shared?*
- *How is this information communicated on a local/regional/national level?*
- *How do you perceive the quality of data collected?*
- *If data quality is poor, is it/how is it used? If poor, what would improve the quality?*
- *Are there "independent" checks of the reported data to ensure they are valid?*
- *Would any other types of data have been helpful in managing the program?*
- *Is there an independent group that evaluates the entire program periodically? If so, please give some details regarding the composition of the group, how it is chosen, and what activities they undertake to evaluate the program?*

29. Is there any recognition for being at the top levels of coverage in the country or for making major improvements? If so, can you give some examples of recognition/incentives?

Follow up:

- *If yes, have you noticed any issues of misreporting due to the possibility of recognition/incentives?*

30. How do you measure the impact of immunization programs?

~ Evaluation; Process ~
~ RQ3. Information Systems ~

For peer review only - http://bmjopen.bmj.com/site/about/guidelines.xhtml
For peer review only

Date: ___________________________ *Interview Code:* ______ - ______ - ______

31. Is there public opinion data on vaccination?<br><br>***~ Evaluation; Process ~***<br>*~ RQ3. Information Systems ~*
Follow up:<br>• *If yes, how can we access this data?*
32. What factors have maintained the sustainability of the immunization program?<br><br>***~ Evaluation; Process ~***
a. **Partnerships:** Does the program have diverse organizations invested in its success?<br>b. **Partnerships**: What does the collaboration between these diverse organizations look like?<br>c. **Organizational capacity**: Were there organizational systems in place to support the program needs?<br>d. **Champion**: Does the program have strong champions? ***(EX: Political or advocacy support outside of the program?)***<br>e. **Funding**: Does the program have sustained funding?<br>i. *Follow up: How important was support of the legislature to program success* ***(EX. appropriating additional funding not initially requesting by the executive branch)****?*<br>f. **Evaluation**: How do evaluation results inform future program planning, implementation, and funding?<br>i. *Follow up: What kind of data drives this?*<br>ii. *Can you share any data with us?*
g. **Adaptation**: How does the immunization program adapt to new evidence?<br><br>***~ Evaluation; Process~***<br>*~RQ1. Why and How? ~*<br>*~ RQ 3. Information Systems ~*
Follow up:<br>• *Where do you learn about new evidence?*
h. **Adaptation**: How does the immunization program adapt to new information?
i. **Communication**: How does the immunization program secure community support or awareness of the issue of childhood vaccines?<br><br>***~ Evaluation; Process~***<br>*~ RQ3. Information Systems ~*<br>*~ RQ4. Intent/Demand for Vaccines ~*<br>*~ RQ5. Knowledge and Resource Sharing ~*<br>*~ RQ6. Workforce ~*
Follow up:<br>• *How does the immunization program maintain community support of childhood vaccines?*<br>• *How does the immunization program maintain awareness of childhood vaccines?*

For peer review only

For peer review only - http://bmjopen.bmj.com/site/about/guidelines.xhtml

Date: ___________________________ *Interview Code: ______ - ______ - ______*

• *To what extent is the immunization program resilient from rumors and misinformation regarding immunizations?*<br>• *How could resilience be improved?*
j. **Media**: How is vaccination portrayed and discussed by the media?<br>***~ Outer Setting ~***<br>*~ RQ3. Information Systems ~*<br>*~ RQ4. Intent/Demand for Vaccines ~*<br>*~ RQ5. Knowledge and Resource Sharing ~*
Follow up:<br>• *What role did the media play in improved coverage?*<br>• *How did the media become involved and how was that involvement sustained?*
33. What kind of planning is there for future resource needs?<br>***~ Evaluation; Process~***<br>*~ RQ5. Knowledge and Resource Sharing ~*
34. What are the greatest barriers to sustained coverage?<br>***~ Reflection; Process~***
35. What are the perceptions of corruption in the public sector?<br>***~ Inner Setting ~***<br>*~ RQ2. Political Will ~*
Follow up:<br>• *Perceptions of quality of electoral processes?*<br>• *Judicial institutions?*<br>• *Anti-corruption efforts?*

For peer review only

Date: ___________________________ *Interview Code:* ______ - ______ - ______

SECTION C. MINISTRY DEPARTMENTS (OR LIAISONS) OF PLANNING, STATISTICS, RESEARCH, HUMAN RESOURCES, COMMUNICATION AND DEVELOPMENT

Objectives:

- To understand the vaccine intervention characteristics during country-specific change points in DTP1/DTP3 coverage
- To identify key internal and external actors, stakeholders, and partnerships in the immunization program both now and in the past
- To explore decision-making process in selecting the strategies and activities in the immunization program that created change points in DTP1/DTP3 coverage
- To identify the strategies behind current immunization programs to inform current context
- To identify previous immunization activities that were key during change points in the DTP1/DTP3 curve
- To understand key barriers and facilitators of interventions in the immunization sector

PART C1 – IMMUNIZATION PROGRAM

1. What were key activities in planning changes for the immunization program?

~ Planning; Process ~
~ RQ5. Knowledge and Resource Sharing ~
~ RQ6. Workforce ~

NOTE: REFER TO ANSWERS GIVEN IN SECTION 2.
IF NOT INVOLVED, SKIP TO Q4.

Follow up:

- *Did you have an action plan?*
- *A theory of change or log frame?*

2. What challenges did you face in the early planning phases?

~ Planning; Process ~

Follow up:

- *How were these challenges overcome* **(EX. Internal to agency/external to agency)***?*

3. What kind of evidence/information **(EX: outbreaks, surveillance data)** did you use to determine changes/interventions?

~ Planning; Process~
~ RQ5. Knowledge and Resource Sharing ~

Follow up:

- *You mentioned* ______________ (DATA SOURCE NAME(S)). *What role did this information have in the push to increase vaccine coverage?*
- *Can you explain how* ______________ (DATA SOURCE NAME(S)) *is collected?*
- *How is this* ______________ (DATA SOURCE NAME(S)) *integrated into other systems?*
- *[If surveillance system not mentioned] How does the surveillance system in* ______________ (COUNTRY) *work?*

4. Prior to the critical changes/intervention, what kind of piloting/testing of the intervention occurred?

~ Planning; Process~
~ RQ4. Intent/Demand for Vaccines ~
~ RQ5. Knowledge and Resource Sharing ~

For peer review only - http://bmjopen.bmj.com/site/about/guidelines.xhtml

Date: ___________________________ *Interview Code:* ______ - ______ - ______

~ RQ6. Workforce ~
Follow up:<br>• *Did the service delivery of vaccinations change?*<br>• *Where did this take place?*<br>• *What was learned in this process?*
5. What were key training or technical assistance activities of the intervention?<br>***~ Planning, Executing; Process ~***<br>*~ RQ5. Knowledge and Resource Sharing ~*<br>*~ RQ6. Workforce ~*
Follow up:<br>• *Who was involved in trainings* ***(NOTE: at all levels)****?*<br>• *Who taught the training?*<br>• *How were they selected (trainers and trainees)?*<br>• *What was their motivation/incentive for involvement?*<br>• *How were the trainings conducted?*
6. If no training activities, what modality did the intervention have?
Follow up:<br>• *What modalities were used for different aspects of the project, generally?*
7. Did you make any changes to the supply chain in $\frac{}{YEAR(S)}$? If so, tell us how.<br>***~ Execution; Process~***<br>*~ Q6. Workforce ~*
Follow up:<br>• *What worked well?*<br>• *What were the challenges?*
8. Did you make any changes to data systems or their use in $\frac{}{YEAR(S)}$? If so, what kind of changes were made and how?<br>***~ Execution; Process~***<br>*~ Q3. Information Systems ~*<br>*~ Q6. Workforce ~*
9. How do you monitor the performance of immunization programs?<br>***~ Evaluation; Process~***<br>*~ RQ3. Information Systems ~*
10. How does this differ from how immunization programs were monitored in the past?<br>***~ Evaluation; Process~***<br>*~ RQ3. Information Systems ~*

For peer review only - http://bmjopen.bmj.com/site/about/guidelines.xhtml

Date: ___________________________ *Interview Code:* ______ - ______ - ______

Follow up:

- *Who is responsible for collecting data related to measures?*
- *How is data used?*
- *What measures are included?*
- *How often is data collected/shared?*
- *How is this information communicated on a local/regional/national level?*
- *How do you perceive the quality of data collected?*
- *If data quality is poor, is it/how is it used? If poor, what would improve the quality?*
- *Are there "independent" checks of the reported data to ensure they are valid?*
- *Would any other types of data have been helpful in managing the program?*
- *Is there an independent group that evaluates the entire program periodically? If so, please give some details regarding the composition of the group, how it is chosen, and what activities they undertake to evaluate the program?*

11. Is there any recognition for being at the top levels of coverage in the country or for making major improvements? If so, can you give some examples of recognition/incentives?

Follow up:

- *If yes, have you noticed any issues of misreporting due to the possibility of recognition/incentives?*

12. What changes have occurred in programming since $\underline{\hspace{3cm}}_{YEAR(S)}$?

13. What factors have helped to ensure the sustainability of the immunization program?

~ Evaluation; Process~

a. **Partnerships:** Does the program have diverse organizations invested in its success? *What does the collaboration between these diverse organizations look like?*

b. **Organizational capacity:** Were there organizational systems in place to support the program needs?

c. **Evaluation:** How do evaluation results inform future program planning, implementation, and funding?

d. **Communication:** How does the immunization program secure or maintain community support of childhood vaccines?
How does the immunization program secure or maintain awareness of childhood vaccines?
How do you define awareness?
To what extent is the immunization program resilient from rumors and misinformation regarding immunizations?
How could resilience be improved?

e. **Funding**: Does the program have sustained funding?
*How important was support of the legislature to program success (**EX. appropriating additional funding not initially requesting by the executive branch**)?*

f. **Adaptation**: How does the immunization program adapt to new evidence?

For peer review only - http://bmjopen.bmj.com/site/about/guidelines.xhtml

Date: ____________________________ *Interview Code:* ______ - ______ - ______

Where do you learn about new evidence?<br><br>g. **Adaptation**: How does the immunization program adapt to new information?<br><br>h. **Media:** How is vaccination portrayed and discussed by the media?<br>*What role did the media play in improved coverage?*<br>*How did the media become involved?*<br>*How was that involvement sustained?*
i. **Strategic planning:** What kind of planning is there for future resource needs?<br><br>***~ Evaluation; Process~***<br>*~ RQ5. Knowledge and Resource Sharing ~*
14. What are the perceptions of corruption in the public sector?<br><br>***~ Inner Setting ~***<br>*~ RQ2. Political Will ~*
Follow up:<br>• *Perceptions of quality of electoral processes?*<br>• *Judicial institutions?*<br>• *Anti-corruption efforts?*

For peer review only

For peer review only - http://bmjopen.bmj.com/site/about/guidelines.xhtml

Date: ___________________________ *Interview Code:* ______ - ______ - ______

SECTION D. MINISTRY DEPARTMENTS (OR LIAISONS) OF INFRASTRUCTURE, EQUIPMENT, FINANCE

Objectives:

- To understand the vaccine intervention characteristics during country-specific change points in DTP1/DTP3 coverage
- To identify key internal and external actors, stakeholders, and partnerships in the immunization program both now and in the past
- To explore decision-making process in selecting the strategies and activities in the immunization program that created change points in DTP1/DTP3 coverage
- To identify the strategies behind current immunization programs to inform current context
- To identify previous immunization activities that were key during change points in the DTP1/DTP3 curve
- To understand key barriers and facilitators of interventions in the immunization sector
- To understand current and historical investments in the immunization sector

PART D1 – POLICY

1. What are the current regulations related to expenditures in health services?

~ External Policies; Outer Setting ~
~ RQ2. Political Will ~

Follow up:

- *What are the current regulations related to vaccination in particular?*

2. How have these regulations changed since 2000?

~ External Policies; Outer Setting ~
~ RQ2. Political Will ~

Follow up:

- *What regulation did you find to be most important/effective in increasing vaccine coverage?*
- *Which supporting documents could you share with me?*
- *What role does the Ministry of Finance have in determining these rules and regulations?*
- *What role does the Ministry of Finance have in implementing the rules and regulations?*
- *What barriers exist?*
- *How are these barriers addressed?*
- *How does this differ from the past?*

3. How have decisions on types of investments in the immunization sector been made?

~ Outer Setting ~
~ RQ1b. Immunization System Context Within the Health Sector ~

Follow up:

- *How are these decisions relative to other investments in other healthcare sectors?*
- *Who determines this?*
- *What percentage/amount of funding is spent on vaccinations as opposed to other health sector areas, including disease surveillance, coverage measurement, accountability?*

PART D2 – IMMUNIZATION PROGRAM

4. Did you make any changes to the supply chain in $\frac{}{YEAR(S)}$? If so, tell us how.

~ Execution; Process~
~ Q6. Workforce ~

For peer review only - http://bmjopen.bmj.com/site/about/guidelines.xhtml

Date: ___________________________ *Interview Code:* ______ - ______ - ______

5. What changes have occurred in immunization programming since $\overline{\quad YEAR(S) \quad}$?	*~ Evaluation; Process ~*
6. What factors have maintained the sustainability of the immunization program?	*~ Evaluation; Process~*
a. **Funding**: Does the program have sustained funding?	*~ Evaluation; Process~* *~ RQ7. Vaccine Policy and Policy Enforcement ~*
b. **Adaptation**: How does the immunization program adapt to new evidence?	*~ Evaluation; Process~* *~RQ1. Why and How? ~* *~ RQ 3. Information Systems ~*
Probes: • *Where do you learn about new evidence?*	
c. **Adaptation**: How does the immunization program adapt to new information?	
Probes: • *Where do you learn about new information?*	
d. **Evaluation**: How do evaluation results inform future program planning, and implementation, and funding?	*~ Evaluation; Process~* *~ RQ3. Information Systems ~*
Follow up: • *What kind of data drives this?* • *Can you share any data with us?*	
j. What are the perceptions of corruption in the public sector?	*~ Inner Setting ~* *~ RQ2. Political Will ~*
Follow up: • *Perceptions of quality of electoral processes?* • *Judicial institutions?* • *Anti-corruption efforts?*	

For peer review only

For peer review only - http://bmjopen.bmj.com/site/about/guidelines.xhtml

Date: ___________________________ *Interview Code:* ______ - ______ - ______

SECTION E. PARTNER ORGANIZATIONS, SUCH AS WHO, UNICEF, CDC, USAID, ETC; NITAG, PROFESSIONAL ORGANIZATION MEMBERS

Objectives:

- To understand the vaccine intervention characteristics during country-specific change points in DTP1/DTP3 coverage
- To identify key internal and external actors, stakeholders, and partnerships in the immunization program both now and in the past
- To explore decision-making process in selecting the strategies and activities in the immunization program that created change points in DTP1/DTP3 coverage
- To identify the strategies behind current immunization programs to inform current context
- To identify previous immunization activities that were key during change points in the DTP1/DTP3 curve
- To understand key barriers and facilitators of interventions in the immunization sector

PART E1 – STAKEHOLDERS

1. What determines opportunities for career advancement for stakeholders involved in vaccination efforts?

~ Relative Priority, Incentives and Rewards; Process ~
~ RQ2. Political Will ~

Follow up:

- *Is there a way of systematically knowing partisan affiliations or who appointed them? Specifically, those involved in agencies relevant to vaccination.*

PART E2 – POLICY

2. What national policies do you think were important in increasing vaccination coverage during ______________?
   YEAR(S)

~ External Policies; Outer Setting~
~ RQ2. Political Will ~
~ RQ7. Vaccine Policy and Policy Enforcement ~

Follow up:

- *How were these policies informed?*
- *What was the reason/rationale for creating these new policies?*
- *What is the policy making process, and how are new policies or recommendations disseminated and implemented once approved?*
- *How do they work in both vertical* ***(EX. across ministries)*** *and horizontal* ***(EX. within different levels of a particular ministry)****?*
- *What led to the selection of these policies?*

PART E3 – IMMUNIZATION PROGRAM

3. What areas of the country have seen the greatest improvement in vaccine rates?

~ Evaluation; Process ~

For peer review only - http://bmjopen.bmj.com/site/about/guidelines.xhtml

Date: ___________________________ *Interview Code:* ______ - ______ - ______

~ RQ3. Information Systems ~<br>*~ RQ4. Intent/Demand for Vaccines ~*
Follow up:<br>• *Was the project specifically tailored to this location?*<br>• *Was it targeted as a critical area for change?*<br>• *What areas saw the quickest improvement?*<br>• *Why do you think these areas had the greatest improvement?*
4. What areas of the country did not see significant changes?<br>***~ Evaluation; Process~***<br>*~ RQ3. Information Systems ~*<br>*~ RQ4. Intent/Demand for Vaccines ~*
Follow up:<br>• *Why do you think this is?* ***(EX: May have already had high coverage, may not relate to challenges)***<br>• *What were the challenges?*<br>○ *Why?*<br>• *What has been/might be done to mitigate this?*
5. What populations are currently the most vulnerable to low coverage?<br>***~ Evaluation; Process~***
6. What populations historically were the most vulnerable to low coverage?<br>***~ Evaluation; Process~***
Follow up:<br>• *How did you identify these populations as vulnerable?*<br>○ *Both previously and now*<br>• *How did you reach out to these populations?*<br>• *What strategy/strategies do you use to reduce inequities?*<br>• *What strategy did you previously use to reduce inequities?*<br>• *What kind of data supports this?*<br>• *Is this data you can share with me?*
7. How do you monitor the performance of immunization programs?
8. How does this differ from how immunization programs were monitored in the past?
Follow up:<br>• *Who is responsible for collecting data related to measures?*<br>• *How is data used?*<br>• *What measures are included?*<br>• *How often is data collected/shared?*<br>• *How is this information communicated on a local/regional/national level?*<br>• *How do you perceive the quality of data collected?*

For peer review only

For peer review only - http://bmjopen.bmj.com/site/about/guidelines.xhtml

Date: ____________________________ Interview Code: ______ - ______ - ______

- *If data quality is poor, is it/how is it used? If poor, what would improve the quality?*
- *Are there "independent" checks of the reported data to ensure they are valid?*
- *Would any other types of data have been helpful in managing the program?*
- *Is there an independent group that evaluates the entire program periodically? If so, please give some details regarding the composition of the group, how it is chosen, and what activities they undertake to evaluate the program?*

9. Is there any recognition for being at the top levels of coverage in the country or for making major improvements? If so, can you give some examples of recognition/incentives?

Follow up:

- *If yes, have you noticed any issues of misreporting due to the possibility of recognition/incentives?*

10. How do you measure the impact of immunization programs?

11. What factors have helped to ensure the sustainability of the immunization program?

~ Evaluation; Process~

a. **Partnerships:** Does the program have diverse organizations invested in its success?
b. **Partnerships**: What does the collaboration between these diverse organizations look like?
c. **Organizational capacity**: Were there organizational systems in place to support the program needs?
d. **Champion**: Does the program have strong champions? ***(EX: Political or advocacy support outside of the program?)***
e. **Funding**: Does the program have sustained funding?
   ii. *Follow up: How important was support of the legislature to program success (**EX. appropriating additional funding not initially requesting by the executive branch**)?*
f. **Evaluation**: How do evaluation results inform future program planning, implementation, and funding?
   iii. *Follow up: What kind of data drives this?*
   iv. *Can you share any data with us?*

g. **Media**: How is vaccination portrayed and discussed by the media?

~ Outer Setting ~
~ RQ3. Information Systems ~
~ RQ4. Intent/Demand for Vaccines ~
~ RQ5. Knowledge and Resource Sharing ~

Follow up:

- *What role did the media play in improved coverage?*
- *How did the media become involved?*
- *How was that involvement sustained?*

h. **Adaptation**: How does the immunization program adapt to new evidence?

~ Evaluation; Process~
~RQ1. Why and How? ~
~ RQ 3. Information Systems ~

For peer review only - http://bmjopen.bmj.com/site/about/guidelines.xhtml

Date: ___________________________ *Interview Code:* ______ - ______ - ______

Follow up:<br><br>• *Where do you learn about new evidence?*
i. **Adaptation**: How does the immunization program adapt to new information?
j. **Communication**: How does the immunization program secure community support or awareness of the issue of childhood vaccines?<br><br>***~ Evaluation; Process~***<br>*~ RQ3. Information Systems ~*<br>*~ RQ4. Intent/Demand for Vaccines ~*<br>*~ RQ5. Knowledge and Resource Sharing ~*<br>*~ RQ6. Workforce ~*
Follow up:<br><br>• *How do you define awareness?*<br>• *How does the immunization program maintain community support of childhood vaccines?*<br>• *How does the immunization program maintain awareness of childhood vaccines?*<br>• *To what extent is the immunization program resilient from rumors and misinformation regarding immunizations?*<br>• *How could resilience be improved?*
12. **Strategic planning**: What kind of planning is there for future resource needs?<br><br>***~ Evaluation; Process~***<br>*~ RQ5. Knowledge and Resource Sharing ~*
13. What are the perceptions of corruption in the public sector?<br>***~ Inner Setting ~***<br>*~ RQ2. Political Will ~*
Follow up:<br><br>• *Perceptions of quality of electoral processes?*<br>• *Judicial institutions?*<br>• *Anti-corruption efforts?*

For peer review only

For peer review only - http://bmjopen.bmj.com/site/about/guidelines.xhtml

Date: ___________________________ *Interview Code:* ______ - ______ - ______

**SECTION G.** PROVINCIAL HEALTH DIRECTOR, SURVEILLANCE, PREVENTION, AND VACCINATION PERSONNEL<br><br>**Objectives:**<br>• To understand the vaccine intervention characteristics during country-specific change points in DTP1/DTP3 coverage<br>• To identify key internal and external actors, stakeholders, and partnerships in the immunization program both now and in the past<br>• To identify the strategies behind current immunization programs to inform current context<br>• To identify previous immunization activities that were key during change points in the DTP1/DTP3 curve<br>• To understand key barriers and facilitators of interventions in the immunization sector<br>• To understand health system characteristics, currently and in the past<br>• To identify and/or confirm project district selection
**PART G1 – HEALTH SYSTEM**
1. Previously, we poke about general success/increase in coverage at the national level. What are some successes in the districts?<br>a.
Follow up:<br>• *Which specific districts would you classify as exemplar districts?*<br>• *Why?*<br>• *Which districts have sustained high coverage from 2011 – 2017?*<br>○ *Was the project specifically tailored to this location?*<br>○ *Was it targeted as a critical area for change?? Why?*<br>○ *What areas saw the quickest improvement?*<br>○ *Why do you think these areas had the greatest improvement?*<br>• *Which areas did not experience significant change?*<br>• *Which populations area most vulnerable to low coverage??*<br>○ *How did you identify these populations as vulnerable? Both previously and now?*<br>○ *How did you reach out to these populations?*<br>○ *What strategy/strategies do you use to reduce inequities?*<br>○ *What kind of data supports this?*<br>○ *Is this data you can share with me?*
2. Here are some districts we have selected based on heterogeneity within the province (with a focus on greater populations). What do you think about these selections?
Follow up:<br>• *Would you make an argument for a different selected/districts to be included? Why?*
3. How many health facilities are available per 1,000 people?
**PART G2 – IMMUNIZATION PROGRAM**
4. What is a <u>specific intervention</u> that contributed to the increase in vaccine coverage in your province?
Follow up:

For peer review only - http://bmjopen.bmj.com/site/about/guidelines.xhtml

Date: ____________________________ *Interview Code:* ______ - ______ - ______

• *Some people have mentioned specific interventions/stakeholders as being critical to the improved coverage, such as* _____________
5. What was your involvement in this intervention(s) and the changes that followed? (IF NO DIRECT INVOLVEMENT IN PLANNING ACTIVITIES, SKIP TO Q9)
Follow up:<br>• *Were these directives from the ministry?*<br>• *How did you decide which changes to make?*<br>• *How did this intervention affect people's roles and responsibilities at different levels in the health system?*<br>• *How were these changes received at different levels of the health system, including parents/end-users?*
6. What were key activities in planning changes for the program?<br>***~ Planning; Process ~***<br>*~ RQ5. Knowledge and Resource Sharing ~*<br>*~ RQ6. Workforce ~*
Follow up:<br>• *Did you have an action plan?*<br>• *A theory of change or log frame?*<br>• *What challenges did you face in the early planning phases?*<br>○ *How were these challenges overcome* ***(EX: Internal to agency/external to agency)****?*
7. Prior to the intervention, what kind of piloting or testing of the intervention occurred?<br>***~ Planning; Process~***<br>*~ RQ4. Intent/Demand for Vaccines ~*<br>*~ RQ5. Knowledge and Resource Sharing ~*<br>*~ RQ6. Workforce ~*
Follow up:<br>• *Did the service delivery of vaccinations change?*<br>• *Where did this take place?*<br>• *What was learned in this process?*
8. What were key training activities?<br>***~ Planning, Executing; Process ~***<br>*~ RQ5. Knowledge and Resource Sharing ~*<br>*~ RQ6. Workforce ~*
Follow up:<br>• *If no training activities, what modality did the intervention have?*<br>• *If training activities, do you have the following information, and can we access it?*<br>• *Who was involved in trainings* ***(NOTE: at all levels)****?*<br>• *Who taught the training?*<br>• *How were they selected*<br>• *Trainers*<br>• *Trainees?*<br>• *What was their motivation/incentive for involvement?*

For peer review only

For peer review only - http://bmjopen.bmj.com/site/about/guidelines.xhtml

Date: ______________________________ Interview Code: _______ - _______ - _______

• *How were the trainings conducted?*<br>• *What were the expectations of the participants in training?*
9. What kind of evidence did you use to determine [changes/interventions]?<br>***~ Planning; Process~***<br>*~ RQ5. Knowledge and Resource Sharing ~*
Follow up:<br>*(E.g., outbreaks, surveillance data).*<br>• *You mentioned ________ (DATA SOURCE NAME(S)). What role did this information have in the push to increase vaccine coverage?*<br>• *Can you explain how ________ (DATA SOURCE NAME(S)) is collected?*<br>• *How is this ________ (DATA SOURCE NAME(S)) integrated into other systems?*<br>• *How does the surveillance system in ________ (COUNTRY). work?*<br>***~ Planning; Process~***<br>*~ RQ5. Knowledge and Resource Sharing~*
10. What do you see as critical for implementation of the [intervention(s)] to succeed?<br>***~ Reflecting and Evaluating; Process ~***<br>*~ Q6. Workforce ~*
Follow up:<br>• *When people give commands from top down, what authority do you have to make changes?*<br>• *What advice would you give to another person/region/country who was going to implement a similar program/project to yours?What are you most proud of in terms of this work?*
11. What challenges/difficulties did you encounter when implementing interventions?<br>***~ Reflecting and Evaluating; Process~***
Follow up:<br>• *How were these challenges overcome?*<br>○ *What contributed to these challenges?*<br>○ *How were decisions made about what to do?*<br>○ *What challenges did you encounter with any new policies made?*<br>○ *Of these challenges, which challenge was the greatest in this province?*<br>○ *Was the project specifically tailored to this location?*<br>• *Who contributed to this decision-making? Why?*
12. What interventions were offered through community health workers?<br>***~ Execution; Process~***<br>*~ RQ6. Workforce ~*
Follow up:<br>• *Outreach activities? Training?*<br>• *Including any kind of reminder system for people to get vaccinations?*

For peer review only - http://bmjopen.bmj.com/site/about/guidelines.xhtml

Date: ___________________________ *Interview Code: ______ - ______ - ______*

• *What kind of system was this? (Cellphone texts, in-person reminders (by who?), church/religious institution reminders?)*<br>• *How was technology a component of this system?*<br>• *Did you develop or already have in place a clinic-based immunization record keeping system to track immunizations administered to individuals?*<br>• *Tools used?*<br>• *How were these developed?*<br>• *What led to the selection of these interventions?*<br>• *Was there variation in different geographic regions etc. If so, why?*<br>• *How were/are CHWs motivated/incentivized for involvement?*<br>• *What are the job requirements to be a CHW?*<br>• *How have responsibilities shifted over time? How do you think this has impacted their performance? Their motivation?*<br>• *What kind of current involvement does the Neighborhood Health Committee have in current CHW programming and responsibilities? What kind of role did they have in the past?*<br>• *What kinds of continued training/capacity building are provided to CHWs?*<br>***~ Beneficiary Needs and Resources; Outer Setting ~***
13. What interventions were offered at various levels of the health system? To providers?<br>***~ Execution; Process~***<br>*~ RQ3. Information Systems ~*<br>*~ RQ6. Workforce ~*
Follow up:<br>• *How were these developed?*<br>• *How were they implemented? Training?*<br>• *Why were they implemented in the way that they were?*<br>• *What led to the selection of these interventions?*<br>• *Was there variation in different geographic regions etc. If so, why?*
14. What programs were offered to parents or soon-to-be parents?<br>***~ Intervention Characteristics~***<br>***~ Outer Setting ~***<br>*~ RQ4. Intent/Demand for Vaccines ~*
Follow up:<br>• *Were these tailored to different areas/cultures/religions/languages/etc.?*<br>• *How were these targeted specifically to mothers, fathers or alternative caretakers?*<br>• *How were they developed?*<br>• *Where did you get the idea for these interventions?*<br>○ *Did you use some other framework or intervention as to inform your intervention?*<br>• *How were they delivered?*

For peer review only

For peer review only - http://bmjopen.bmj.com/site/about/guidelines.xhtml

Date: ___________________________ Interview Code: ______ - ______ - ______

• *Who was engaged in delivery?*<br>• *What determined their involvement?*<br>• *What kinds of modalities were used?*<br>• *What kind of communication materials (if any) were utilized? Mass media? Dissemination through health centers? Other?*<br>• *How did you get individuals to participate in this program?*<br>• *What led to their selection?*<br>• *Could you share any materials with us?*<br>*~ Beneficiary needs and resources ~*
15. Did you make any changes to the supply chain in $\frac{}{YEAR(S)}$?
Follow up:<br>• *What worked well?*<br>• *What were challenges?*
16. Are there relevant polices at the subnational/regional level? If so, what?<br>***~ External Policies; Outer Setting ~***<br>*~ RQ2. Political Will ~*<br>*~ RQ7. Vaccine Policy and Policy Enforcement ~*
Follow up:<br>• *If there no policies at the subnational/regional level, do all regions need to follow the national law, or can they create their own?*<br>• *How were these policies informed?*<br>• *What data were most important in setting policy?*<br>• *What kind of global/regional resources were utilized?*<br>• *How were policies implemented?*<br>• *What led to the selection of these policies?*
17. What policies were made at the local level?<br>***~ External Policies; Outer Setting ~***<br>*~ RQ2. Political Will ~*<br>*~ RQ7. Vaccine Policy and Policy Enforcement ~*
Follow up:<br>• *How were these policies informed?*<br>• *How were they implemented?*<br>• *What led to the selection of these policies?*
18. Did you make any changes to data systems in $\frac{}{YEAR(S)}$?<br>***~ Execution; Process~***<br>*~ Q3. Information Systems ~*<br>*~ Q6. Workforce ~*

For peer review only - http://bmjopen.bmj.com/site/about/guidelines.xhtml

Date: ___________________________ *Interview Code: ______ - ______ - ______*

Follow up:<br>• *How were these changes made?*<br>• *Why was this change made?*<br>○ *Has this change had the anticipated impact* ***(EX: on coverage or otherwise)****?*<br>• *Did you develop or already have in place a clinic-based immunization record keeping system to track immunizations administered to individuals?*<br>• *Were parents responsible for keeping track of records?*<br>• *To what extent was technology a component of record keeping* ***(EX: computer, phone data collection)****?*
NOTE: REMINDER SYSTEM DEFINED AS A MESSAGE SENT BEFORE APPOINTMENTS TO REMIND PARENTS TO COME; RECALL SYSTEM DEFINED AS A NOTICE SENT TO PARENTS WHO FAIL TO KEEP THEIR APPOINTMENTS.
19. What changes were made to human resources in ____________ ?<br>YEAR(S)<br>***~ Execution; Process~***<br>*~ Q6. Workforce ~*
Follow up:<br>• *If so, what kind of changes were made and how?*
20. How do you monitor the performance of immunization programs?<br>***~ Evaluation; Process ~***<br>*~ RQ3. Information Systems ~*
Follow up:<br>• *How is this different from other health systems?*
21. How does this differ from how immunization programs were monitored in the past?<br>***~ Evaluation; Process ~***<br>*~ RQ3. Information Systems ~*
Follow up:<br>• *Who is responsible for collecting data related to measures?*<br>• *How is data used?*<br>• *What measures are included?*<br>• *How often is data collected/shared?*<br>• *How is this information communicated on a local/regional/national level?*<br>• *How do you perceive the quality of data collected?*<br>• *If data quality is poor, is it/how is it used? If poor, what would improve the quality?*<br>• *Are there "independent" checks of the reported data to ensure they are valid?*<br>• *Would any other types of data have been helpful in managing the program?*<br>• *Is there an independent group that evaluates the entire program periodically? If so, please give some details regarding the composition of the group, how it is chosen, and what activities they undertake to evaluate the program?*

For peer review only - http://bmjopen.bmj.com/site/about/guidelines.xhtml

Date: ___________________________ *Interview Code:* ______ - ______ - ______

22. Is there any recognition for being at the top levels of coverage in ____________ (COUNTRY). or for making major improvements? If so, can you give some examples of recognition/incentives?

Follow up:

- *If yes, have you noticed any issues of misreporting due to the possibility of recognition/incentives?*

23. How do you measure the impact of immunization programs?

~ Evaluation; Process~
~ RQ3. Information Systems ~

24. What factors have maintained the sustainability of the immunization program?

~ Evaluation; Process~

Follow up:

- ***Partnerships:*** *Diverse organizations? Collaboration?*
- ***Champion:*** *Political advocacy or support?*
- ***Funding:*** *Sustained funding? What will happen to the program when Gavi funding is no longer present? What do you think about this planning?*
- ***Evaluation:*** *Informed future funding?*
- ***Adaptation:*** *How does new evidence and information impact the program?*

25. **Communication**: How does the immunization program maintain community support of childhood vaccines?

~ Evaluation; Process~
~ RQ3. Information Systems ~
~ RQ4. Intent/Demand for Vaccines ~
~ RQ5. Knowledge and Resource Sharing ~
~ RQ6. Workforce ~

Follow up:

- *To what extent is the immunization program resilient from rumors and misinformation regarding immunizations?*
- *What kind of misinformation regarding immunizations exist?*
- *How could resilience be improved?*

26. **Media**: How is vaccination portrayed and discussed by the media?

~ Outer Setting ~
~ RQ3. Information Systems ~
~ RQ4. Intent/Demand for Vaccines ~
~ RQ5. Knowledge and Resource Sharing ~

Follow up:

- *What role did the media play in improved coverage?*
- *How did the media become involved?*
- *How is that involvement sustained?*

27. **Strategic planning:** What kind of planning is there for future resource needs?

~ Evaluation; Process ~
~ RQ5. Knowledge and Resource Sharing ~

28. What are the perceptions of corruption in the public sector?

~ Inner Setting ~
~ RQ2. Political Will ~

Follow up:

- *Perceptions of quality of electoral processes?*

For peer review only - http://bmjopen.bmj.com/site/about/guidelines.xhtml

Date: ___________________________ *Interview Code: ______ - ______ - ______*

- *Judicial institutions?*
- *Anti-corruption efforts?*

For peer review only - http://bmjopen.bmj.com/site/about/guidelines.xhtml

Date: ___________________________ *Interview Code:* ______ - ______ - ______

SECTION H. DISTRICT HEAD, SURVEILLANCE, PREVENTION, VACCINATION PERSONNEL

Objectives:

- To understand the vaccine intervention characteristics during country-specific change points in DTP1/DTP3 coverage
- To identify key internal and external actors, stakeholders, and partnerships in the immunization program both now and in the past
- To identify the strategies behind current immunization programs to inform current context
- To identify previous immunization activities that were key during change points in the DTP1/DTP3 curve
- To understand key barriers and facilitators of interventions in the immunization sector
- To understand health system characteristics, currently and in the past

PART H1 – HEALTH SYSTEM

1. What is the general condition of the health facilities in your district? How does this vary at different levels?
2. Are vaccines available when they are needed? Are vaccines available where they are needed?

Follow up:

- *Are staff available when and where needed?*
- *Are supplies available when and where needed?* ***(EX: Syringes, other supplies?)***
- *Are vaccines availability tracked?*
  - *By location?*
  - *Quantity?*
  - *Timing?*
- *How do vaccines arrive? Are they in ideal conditions?*
- *Where are the vaccines usually stored, and at what temperature?*
- *What levels or conditions prompt restocking?*
  - *Are the minimum supply levels maintained?*
- *How long does it take to restock your supply of vaccines?*

3. How many clinics are available per 1,000 people?

Follow up:

- *What is the average distance/length of time that a person must travel to get to the clinic?*
- *On average, how long does someone wait to receive vaccines?*
- *Are there other services included in vaccine visits?*

PART H2 – IMMUNIZATION PROGRAM

4. What is a specific intervention that contributed to the increase in vaccine coverage?
5. What was your involvement in the intervention(s) and the changes that followed? *(IF NO DIRECT INVOLVEMENT, SKIP TO Q7)*

Follow up:

- *Were these directives from the ministry?*
- *How did you decide which changes to make?*
- *How did this intervention affect people's roles and responsibilities at different levels in the health system?*

For peer review only - http://bmjopen.bmj.com/site/about/guidelines.xhtml

Date: ____________________________ *Interview Code:* ______ - ______ - ______

6. What were key activities in <u>planning</u> changes for the program?<br><br>***~ Planning; Process ~***<br>*~ RQ5. Knowledge and Resource Sharing ~*<br>*~ RQ6. Workforce ~*
Follow up:<br><br>• *Did you have an action plan?*<br>• *A theory of change or log frame?*<br>• *What challenges did you face in the early planning phases?*<br>○ *How were these challenges overcome* ***(EX: Internal to agency/external to agency)****?*
7. What were key training activities?<br><br>***~ Planning, Executing; Process ~***<br>*~ RQ5. Knowledge and Resource Sharing ~*<br>*~ RQ6. Workforce ~*
Follow up:<br><br>• *If no training activities, what modality did the intervention have?*<br>• *If training activities, do you have the following information, and can we access it?*<br>• *Who was involved in trainings* ***(NOTE: at all levels)****?*<br>• *Who taught the training?*<br>• *How were they selected*<br>• *Trainers*<br>• *Trainees?*<br>• *What was their motivation/incentive for involvement?*<br>• *How were the trainings conducted?*<br>• *What were the expectations of the participants in training?*
8. What kind of evidence did you use to determine [changes/interventions]?<br><br>***~ Planning; Process ~***<br>*~ RQ5. Knowledge and Resource Sharing ~*
Follow up:<br><br>*(E.g., outbreaks, surveillance data).*<br><br>• *You mentioned* ________ *DATA SOURCE NAME(S)* *. What role did this information have in the push to increase vaccine coverage?*<br>• *Can you explain how* ________ *DATA SOURCE NAME(S)* *is collected?*<br>• *How is this* ________ *DATA SOURCE NAME(S)* *integrated into other systems?*<br>• *How does the surveillance system in* ________ *COUNTRY* *work?*<br>***~ Planning; Process~***<br>*~ RQ5. Knowledge and Resource Sharing~*
9. What do you see as critical for <u>implementation</u> of the [intervention(s)] to succeed?<br><br>***~ Reflecting and Evaluating; Process~***<br>*~ Q6. Workforce ~*

For peer review only

For peer review only - http://bmjopen.bmj.com/site/about/guidelines.xhtml

Date: ___________________________ *Interview Code:* ______ - ______ - ______

Probes:<br>• *What advice would you give to another person/region/country who was going to implement a similar program/project to yours?*<br>• *What are you most proud of in terms of this work?*
10. What challenges/difficulties did you encounter during <u>implementation</u> of the interventions?<br>***~ Reflecting and Evaluating; Process ~***
Follow up:<br>• *How were these challenges overcome?*<br>• *What contributed to these challenges?*<br>• *How were decisions made about what to do?*<br>• *Who contributed to this decision-making? Why?*<br>• *What challenges did you encounter with any new policies made?*<br>• *Of these challenges, which challenge was the greatest in this region?*<br>•
11. What areas of the district have seen the greatest improvement in vaccine rates? Can you share this data?<br>***~ Evaluation; Process~***<br>*~ RQ3. Information Systems ~*<br>*~ RQ4. Intent/Demand for Vaccines ~*
Follow up:<br>• *Was the project specifically tailored to this location?*<br>• *Was it targeted as a critical area for change?*<br>• *What areas saw the quickest improvement?*<br>• *Why do you think these areas had the greatest improvement?*
12. What areas did not experience significant changes?<br>***~ Evaluation; Process~***<br>*~ RQ3. Information Systems ~*<br>*~ RQ4. Intent/Demand for Vaccines ~*
Follow up:<br>• *Why do you think this is?* ***(EX: May have already had high coverage, may not relate to challenges)***<br>• *What were the challenges?*<br>○ *Why?*<br>• *What has been/might be done to mitigate this?*
13. What populations historically were the most vulnerable to low coverage? Why?<br>***~ Evaluation; Process~***
Follow up:<br>• *How did you identify these populations as vulnerable?*<br>○ *Both previously and now*

For peer review only - http://bmjopen.bmj.com/site/about/guidelines.xhtml

Date: ___________________________ Interview Code: ______ - ______ - ______

- *How did you reach out to these populations?*
- *What strategy/strategies do you use to reduce inequities?*
- *What strategy did you previously use to reduce inequities?*
- *What kind of data supports this?*
- *Is this data you can share with me?*

14. What interventions were offered through Community Health Workers?

~ Execution; Process ~
~ RQ6. Workforce ~

Follow up:

- *Outreach activities? Training?*
  - *Including any kind of reminder system for people to get vaccinations?*
    - ***What kind of system was this? (Cellphone texts, in-person reminders (by who?), church/religious institution reminders?)***
    - *How was technology a component of this system?*
    - *Did you develop or already have in place a clinic-based immunization record keeping system to track immunizations administered to individuals?*
- *Tools used?*
- *How were these developed?*
- *What led to the selection of these interventions?*
- *Was there variation in different geographic regions etc. If so, why?*
- *How were/are CHWs motivated/incentivized for involvement?*
- *What are the job requirements to be a CHW?*
- *How have responsibilities shifted over time? How do you think this has impacted their performance? Their motivation?*
- *What kind of current involvement does the Neighborhood Health Committee have in current CHW programming and responsibilities? What kind of role did they have in the past?*
- *What kinds of continued training/capacity building are provided to CHWs?*

~ Beneficiary Needs and Resources; Outer Setting ~

15. What interventions were offered at various levels of the health system? For providers?

~ Execution; Process~
~ RQ3. Information Systems ~
~ RQ6. Workforce ~

Follow up:

- *How were these developed?*
- *How were they implemented? Training*
- *Why were they implemented in the way that they were?*
- *What led to the selection of these interventions?*
- *Was there variation in different geographic regions etc. If so, why?*
- *What is the educational background of providers?*

16. What key interventions or programs were offered to parents or soon-to-be parents?

~ Intervention Characteristics~
~ Outer Setting ~
~ RQ4. Intent/Demand for Vaccines ~

For peer review only - http://bmjopen.bmj.com/site/about/guidelines.xhtml

Date: ____________________________ *Interview Code:* ______ - ______ - ______

<table>
<tr><td>

Follow up:

- *Were these tailored to different areas/cultures/religions/languages/etc.?*
- *How were these targeted specifically to mothers, fathers or alternative caretakers?*
- *How were they developed?*
- *Where did you get the idea for these interventions?*
  - *Did you use some other framework or intervention to inform your intervention?*
- *How were they delivered?*
- *Who was engaged in delivery?*
- *What determined their involvement?*
- *Who determines the programming/methods?*
- *Who approves the programming/methods?*
- *Who formulates the messages?*
- *What kinds of modalities were used?*
- *What kind of communication materials (if any) were utilized? Mass media? Dissemination through health centers? Other?*
- *How did you get individuals to participate in this program?*
- *What led to their selection?*
- *Could you share any materials with us?*

~ Beneficiary needs and resources ~
</td></tr>
<tr><td>
17. Did you make any changes to the supply chain in $\overline{YEAR(S)}$? If so, tell us how.

~ Execution; Process ~
~ Q6. Workforce ~
</td></tr>
<tr><td>

Follow up:

- *What worked well?*
  What were challenges?
</td></tr>
<tr><td>
18. What strategies did you use to reduce the drop-out rate?
</td></tr>
<tr><td>
19. How do you monitor the performance of immunization programs?

~ Evaluation; Process ~
~ RQ3. Information Systems ~
</td></tr>
<tr><td>
20. How does this differ from how immunization programs were monitored in the past?

~ Evaluation; Process ~
~ RQ3. Information Systems ~
</td></tr>
<tr><td>

Follow up:

- *Who is responsible for collecting data related to measures?*
- *How is data used?*
- *What measures are included?*
- *How often is data collected/shared?*
- *How is this information communicated on a local/regional/national level?*
- *How do you perceive the quality of data collected?*
</td></tr>
</table>

For peer review only - http://bmjopen.bmj.com/site/about/guidelines.xhtml

Date: ___________________________ *Interview Code: ______ - ______ - ______*

• *If data quality is poor, is it/how is it used? If poor, what would improve the quality?*<br>• *Are there "independent" checks of the reported data to ensure they are valid?*<br>• *Would any other types of data have been helpful in managing the program?*<br>• *Is there an independent group that evaluates the entire program periodically? If so, please give some details regarding the composition of the group, how it is chosen, and what activities they undertake to evaluate the program?*
21. Is there any recognition for being at the top levels of coverage in the country or for making major improvements? If so, can you give some examples of recognition/incentives?
Follow up:<br><br>• *Have you noticed any issues of misreporting due to the possibility of rewards?*
22. What factors have maintained sustainability of the immunization program?<br><br>***~ Evaluation; Process~***
Follow up:<br><br>• *Partnerships: Diverse organizations? Collaboration?*<br>• *Champion: Political advocacy or support?*<br>• *Funding: Sustained funding? What will happen to the program when Gavi funding is no longer present? What do you think about this planning?*<br>• *Evaluation: Informed future funding?*<br>• *Adaptation: How does new science and knowledge impact the program?*
a. **Communication**: How does the immunization program secure awareness of the issue of childhood vaccines?<br><br>***~ Evaluation; Process~***<br>*~ RQ3. Information Systems ~*<br>*~ RQ4. Intent/Demand for Vaccines ~*<br>*~ RQ5. Knowledge and Resource Sharing ~*<br>*~ RQ6. Workforce ~*
Follow up:<br><br>• *How does the immunization program maintain community support of childhood vaccines?*<br>• *To what extent is the immunization program resilient from rumors and misinformation regarding immunizations?*<br>• *How could resilience be improved?*
b. **Media**: How is vaccination portrayed and discussed by the media?<br><br>***~ Outer Setting ~***<br>*~ RQ3. Information Systems ~*<br>*~ RQ4. Intent/Demand for Vaccines ~*<br>*~ RQ5. Knowledge and Resource Sharing ~*
Follow up:<br><br>• *What role did the media play in improved coverage?*<br>• *How did the media become involved and how was that involvement sustained?*

For peer review only - http://bmjopen.bmj.com/site/about/guidelines.xhtml

Date: ____________________________ *Interview Code:* ______ - ______ - ______

c. **Strategic planning**: What kind of planning is there for future resource needs?<br><br>***~ Evaluation; Process ~***<br>*~ RQ5. Knowledge and Resource Sharing ~*
23. What are the perceptions of corruption in the public sector?<br>***~ Inner Setting ~***<br>*~ RQ2. Political Will ~*
Follow up:<br><br>• *Perceptions of quality of electoral processes?*<br>• *Judicial institutions?*<br>• *Anti-corruption efforts?*

For peer review only

For peer review only - http://bmjopen.bmj.com/site/about/guidelines.xhtml

Date: ___________________________ *Interview Code:* ______ - ______ - ______

SECTION I. CLINIC HEAD

PART 1 – HEALTH SYSTEM

Objectives:

- To understand the vaccine intervention characteristics during country-specific change points in DTP1/DTP3 coverage
- To identify key internal and external actors, stakeholders, and partnerships in the immunization program both now and in the past
- To identify the strategies behind current immunization programs to inform current context
- To identify previous immunization activities that were key during change points in the DTP1/DTP3 curve
- To understand key barriers and facilitators of interventions in the immunization sector
- To understand health system characteristics, currently and in the past
- To understand community norms and practices related to vaccinations

1. How would you describe the general condition of your health facility?

2. Are vaccines available when and where they are needed?

Follow up:

- *Are staff available when and where needed?*
- *Are supplies available when and where needed?* ***(EX: Syringes, other supplies)***.
- *Are vaccines availability tracked?*
  - *By location?*
  - *Quantity?*
  - *Timing?*
- *How do vaccines arrive? Are they in ideal conditions?*
- *Where are the vaccines usually stored, and at what temperature?*
- *What levels or conditions prompt restocking?*
  - *Are there minimum supply levels maintained?*

How long does it take to restock your supply of vaccine?

3. *How does* [COMMUNITY] *view* vaccines?

~ ***Decision-making process****~*
~Current factors behind immunization~
~Demand-side~

Follow up:

- *What are the main facilitators to people getting vaccines in your community?*
  - ***Examples****:*
    - *Encouragement from healthcare workers*
    - *Shame*
    - *Geography/Accessibility*
    - *Trust of vaccinators/healthcare workers*

For peer review only - http://bmjopen.bmj.com/site/about/guidelines.xhtml

Date: ___________________________ *Interview Code: ______ - ______ - ______*

- *Messaging/Increased Awareness*
- *Community expectation*
- *Seasonality*
- *Availability*

- *What are the main barriers to people getting vaccines in your community?*
  - ***Examples:***
    - *Geography/Accessibility*
    - *Mistrust of healthcare workers*
    - *Stock outs*
    - *Cold chain*
    - *Vaccine hesitancy (ex. Side effects)*
    - *Influence of community/religious leaders*
    - *Seasonality*
    - *Wastage*
- *How are these barriers addressed?*
- *Are there groups less likely to prioritize vaccines? Who and why?*
- *Are there children who receive some infant vaccines but don't receive others? Why?*
- *How are vaccines prioritized compared to other health interventions/measures?*

4. How have outbreaks of diseases affected vaccination rates?

~Evaluation; Process~
~RQ3. Information Systems~
~RQ4. Intent/Demand for Vaccines~

5. What services do you think the government is responsible for? Compared to other government responsibilities, how important are vaccines?

~Intervention; Outer setting~

Follow up:

- *Why are they important/not important?*
- *What is the most important to [COMMUNITY]?*
- *What is the least important to [COMMUNITY]?*

6. How has this health facility's ability to provide vaccines changed over time?

Follow up:

- *What changed?*
  - ***Examples:***
    - *Demand from community?*
    - *Supply*
      Improved systems?
    - *Training?*
    - *Government emphasis?*
- *About what year was this?*
  - *What other events happened around this time?*
    - *Political*

For peer review only - http://bmjopen.bmj.com/site/about/guidelines.xhtml

Date: ___________________________ *Interview Code: ______ - ______ - ______*

- *Infrastructure changes*
- *Other health issues*

- *Can you think of any certain events that increased or decreased vaccinations?*
  - *Strikes*
  - *Extreme weather*
  - *Outbreaks? What disease? Vaccine preventable (flu, measles, mumps, rubella, pertussis)?*

PART I2 – IMMUNIZATION PROGRAM

7. What is a specific intervention that contributed to the increase in vaccine coverage?

Follow up:

- *What was your involvement in this intervention(s) and the changes that followed?*
- *How were the changes received?*
- *Who were the key decision-makers of this intervention?*

8. What key interventions or programs were offered to parents or soon-to-be parents?

~ Intervention Characteristics~
~Outer Setting ~
~ RQ4. Intent/Demand for Vaccines ~

Follow up:

- *Were these tailored to different areas/cultures/religions/languages/etc.?*
- *How were these targeted specifically to mothers, fathers or alternative caretakers?*
- *How were they developed?*
- *Where did you get the idea for these interventions?*
  - *Did you use some other framework or intervention as to inform your intervention?*
- *How were they delivered?*
- *Who was engaged in delivery?*
- *What determined their involvement?*
- *Who determines the program/methods?*
- *Who formulates the messages?*
- *What kinds of modalities were used?*
- *What kind of communication materials (if any) were utilized? Mass media? Dissemination through health centers? Other?*
- *How did you get individuals to participate in this program?*
- *What led to their selection?*
- *What led to their selection?*
- *What encouraged parents to be more engaged in their children's health?*
- *Was this specific to immunization or general health?*
- *Were these interventions offered in combination with other health interventions?*
- *What are you doing to reduce dropout rates?*
- *What kind of awareness campaigns are there?*
- *Could you share any materials with us?*

~ Beneficiary needs and resources ~

9. What interventions were offered through Community Health Workers?

~ Execution; Process ~

For peer review only

For peer review only - http://bmjopen.bmj.com/site/about/guidelines.xhtml

Date: ___________________________   *Interview Code: ______ - ______ - ______*

<table>
<tr><td align="right">~ RQ6. Workforce ~</td></tr>
<tr><td>
Follow up:

Outreach activities? Training?

Including any kind of reminder system for people to get vaccinations?

What kind of system was this? (Cellphone texts, in-person reminders (by who?), church/religious institution reminders?)
How was technology a component of this system?
Did you develop or already have in place a clinic-based immunization record keeping system to track immunizations administered to individuals?

Tools used?
How were these developed?
What led to the selection of these interventions?
Was there variation in different geographic regions etc. If so, why?
How were/are CHWs motivated/incentivized for involvement?
What are the job requirements to be a CHW?
How have responsibilities shifted over time? How do you think this has impacted their performance? Their motivation?
Is there a correlation between incentives and outcomes?
What kind of current involvement does the Neighborhood Health Committee have in current CHW programming and responsibilities? What kind of role did they have in the past?
What kinds of continued training/capacity building are provided to CHWs?

~ Beneficiary Needs and Resources; Outer Setting ~
</td></tr>
<tr><td>
10. What interventions were offered at various levels of the health system? To providers?
~ Execution; Process~
~ RQ3. Information Systems ~
~ RQ6. Workforce ~
</td></tr>
<tr><td>
Follow up:

How were these developed?
How were they implemented? Training?
Why were they implemented in the way that they were?
What led to the selection of these interventions?
Was there variation in different geographic regions etc. If so, why?
What is the educational background of providers?

</td></tr>
<tr><td>11. How is your work monitored?</td></tr>
<tr><td>
12. How do you monitor the performance of immunization programs?
~ Evaluation; Process~
~ RQ3. Information Systems ~
</td></tr>
<tr><td>
13. How does this differ from how immunization programs were monitored in the past?
~ Evaluation; Process~
~ RQ3. Information Systems ~
</td></tr>
</table>

For peer review only - http://bmjopen.bmj.com/site/about/guidelines.xhtml

Date: ___________________________ *Interview Code: ______ - ______ - ______*

Follow up:

- *Who is responsible for collecting data related to measures?*
- *How is data used?*
- *What measures are included?*
- *How often is data collected/shared?*
- *How is this information communicated on a local/regional/national level?*
- *How do you perceive the quality of data collected?*
- *If data quality is poor, is it/how is it used? If poor, what would improve the quality?*
- *Are there "independent" checks of the reported data to ensure they are valid?*
- *Would any other types of data have been helpful in managing the program?*
- *Is there an independent group that evaluates the entire program periodically? If so, please give some details regarding the composition of the group, how it is chosen, and what activities they undertake to evaluate the program?*

14. Is there any recognition for being at the top levels of coverage in the country or for making major improvements? If so, can you give some examples of recognition/incentives?

Follow up:

- *Have you noticed any issues of misreporting due to the possibility of rewards?*

15. How do you measure the impact of immunization programs?

16. What was key to success when implementing interventions?

Reflecting and Evaluating; Process~
~ Q6. Workforce ~

Follow up:

- *What advice would you give to another person/region/country who was going to implement a similar program/project to yours?*
- *What are you most proud of in terms of this work?*

17. What challenges/difficulties did you encounter when implementing interventions?

~ Reflecting and Evaluating; Process~

Follow up:

- *How were these challenges overcome?*
- *What challenges did you encounter with any new policies made?*

18. What changes have occurred in programming since this intervention(s) was implemented?

~ Evaluation; Process~
~ RQ3. Information Systems ~

19. What changes have occurred in programming since ______ /these interventions were implemented?
YEAR(S)

~ Evaluation; Process~

20. What factors have maintained the sustainability of the immunization program?

~ Evaluation; Process~

For peer review only - http://bmjopen.bmj.com/site/about/guidelines.xhtml

Date: ___________________________ *Interview Code:* ______ - ______ - ______

Follow up:<br>• ***Partnerships:*** *Diverse organizations? Collaboration?*<br>• ***Champion:*** *Political advocacy or support?*<br>• ***Funding:*** *Sustained funding? What will happen to the program when Gavi funding is no longer present? What do you think about this planning?*<br>• ***Evaluation:*** *Informed future funding?*<br>• ***Adaptation:*** *How does new science and knowledge impact the program?*
d. **Communication**: How does the immunization program secure community support or awareness of the issue of childhood vaccines?<br>***~ Evaluation; Process ~***<br>*~ RQ3. Information Systems ~*<br>*~ RQ4. Intent/Demand for Vaccines ~*<br>*~ RQ5. Knowledge and Resource Sharing ~*<br>*~ RQ6. Workforce ~*
Follow up:<br>• *How do you define awareness? What is the community aware of (EX. Benefits, services offered, etc.)*<br>• *How does the immunization program maintain community support of childhood vaccines?*<br>• *To what extent is the immunization program resilient from rumors and misinformation regarding immunizations?*<br>• *How could resilience be improved?*
e. **Media**: How is vaccination portrayed and discussed by the media?<br>***~ Outer Setting ~***<br>*~ RQ3. Information Systems ~*<br>*~ RQ4. Intent/Demand for Vaccines ~*<br>*~ RQ5. Knowledge and Resource Sharing ~*
Follow up:<br>• *What role did the media play in improved coverage?*<br>• *How did the media become involved and how was that involvement sustained?*
f. **Strategic planning:** To what extent is there planning for future resource needs?<br>***~ Evaluation; Process~***<br>*~ RQ5. Knowledge and Resource Sharing ~*
21. What are the greatest barriers to sustained coverage?<br>***~ Reflection; Process~***
22. What are the perceptions of corruption in the public sector?<br>***~ Inner Setting ~***<br>*~ RQ2. Political Will ~*
Follow up:<br>• *Perceptions of quality of electoral processes?*<br>• *Judicial institutions?*<br>• *Anti-corruption efforts?*

For peer review only - http://bmjopen.bmj.com/site/about/guidelines.xhtml

Date: ___________________________ *Interview Code:* ______ - ______ - ______

SECTION J. COMMUNITY LEADERS (RELIGIOUS LEADERS, HEAD MAN, CHIEFS,

Objectives:

- To understand their role, if any, as it relates to health interventions, specifically immunization coverage
- To identify key community stakeholders who have a role in immunization programming
- To understand the cultural context and environmental context of the selected community
- To understand what drives community acceptance of immunizations
- To understand the motivations of key community stakeholders in vaccine intervention characteristics during country-specific change points in DTP1/DTP3 coverage
- To identify the strategies behind current immunization programs to inform current context
- To identify previous immunization activities — including specific interventions, delivery systems, policies, facilitators and barriers — that were key during change points in the DTP1/DTP3 curve
- To understand specific barriers and facilitators to implementation of interventions in the immunization sector

PART J1 – INTERVIEW INTRODUCTION

Interviewer Script: Thank you very much for your willingness to meet with us today and share your experience and knowledge regarding the immunization program in ____________ (COUNTRY). I'd like to begin by learning about you and your role related to the immunization program.

1. Can you tell me about your role in ________________ (THIS COMMUNITY)?

~ Individual Setting ~

Follow up:

- *How does your role relate to health care programming?*
- *How does your role relate to the immunization program?*
- *Responsibilities (past and present)*
- *What are the contributions you feel you've made in the immunization programming in your community?*
- *What has motivated you to make these contributions?*

2. Who are other community leaders/stakeholders who have had an impact on the immunization program?

~ Inner Setting ~

Follow up:

- *What role did they play?*
- *What were their key contributions?*
- *What motivated their involvement?*
- *What were their top three incentives for performing well?*
- *What are the stakeholders' party/group affiliation?*
- *Are their positions relevant to vaccinations?*

Interviewer Script: Now I'd like to learn about the health care services in your community, and how immunization coverage plays a part. I'd also like to hear a bit more about community attitudes towards immunizations.

For peer review only - http://bmjopen.bmj.com/site/about/guidelines.xhtml

Date: ___________________________ *Interview Code:* ______ - ______ - ______

3. How does *[COMMUNITY]* view vaccines?

~ Inner Setting ~
~ Evaluation; Process ~
~Decision making process; demand-side~
~current factors behind immunization~

Probes:

- *What are the main facilitators to people getting vaccines in your community]?*
  - *Encouragement from health care workers*
  - *Shame*
  - *Geography*
  - *Enforcement*
  - *Trust of vaccinators/health care workers*
  - *Messaging/Awareness*
  - *Community expectation?*
  - *Fundamental right?*
  - *Seasonality?*
- *What are the main barriers to people getting vaccines in your community?*
  - *Geography*
  - *Mistrust of health care workers*
  - *Stock out*
  - *Cold chain*
  - *Vaccine hesitancy (ex. side effects)*
  - *Influence of community/religious leaders*
  - *Seasonality*
  - *Wastage*
- *How are these barriers addressed?*
- *Are there groups less likely to prioritize vaccines? Who and why?*
- *Are there children who receive infant vaccines (ex. DTP1) but don't receive others (ex. DTP2, DTP3)? Why?*
- *How are vaccines prioritized compared to other health interventions/measures?*

4. How have outbreaks of diseases affected mandatory vaccination rates?

~ Evaluation; Process ~
~ RQ3. Information Systems ~
~ RQ4. Intent/Demand for Vaccines ~

Probes:

Did vaccination rates increase or decrease?

5. Compared to other government responsibilities - like public safety, roads and infrastructure, education – how important are vaccines in this community?

~Intervention; Outer Setting~

Probes:

- *Why are they important/not important?*
- *What is the most important to you / [COMMUNITY]?*
- *What is the least important to you / [COMMUNITY]?*
- *Who do you believe is responsible for providing vaccines?*

For peer review only - http://bmjopen.bmj.com/site/about/guidelines.xhtml

Date: ___________________________ *Interview Code: ______ - ______ - ______*

- *Government?*
- *NGOs and aid agencies?*

6. How has the health facility's ability to provide vaccines changed over time?

~Intervention; Inner setting~

Probes:

- *What changed?*
  - *Demand from community?*
  - *Supply?*
  - *Improved systems?*
  - *Training?*
  - *Governmental emphasis?*
- *About what year was this?*
  - *What other events happened around this time?*
    - *Political*
    - *Infrastructure changes*
    - *Other health issues*
- *Can you think of any certain events that increased or decreased vaccinations?*
  - *Strikes*
  - *Extreme weather*
  - *Outbreaks*
    - *What diseases?*
      - *Vaccine preventable (flu, measles, mumps, rubella, pertussis etc.)*
      - *Non-vaccine preventable?*

7. What are specific interventions/programming shifts that have happened in the last ten years?

[Intent: Identify past immunization activities and interventions; identify previous key actors in the program]

~ Evaluation; Process~

[CFIR – Intervention, Outer Setting, Inner Setting, Characteristics of Individuals, Process]

Probes:

- *What intervention had the greatest impact?*
- *How was this intervention implemented in your community?*
- *How did people react to this intervention?*
- *What were the facilitators to uptake?*
- *What were barriers to uptake?*

8. What factors have contributed to the sustainability of the immunization program?

~ Evaluation; Process~

a. **Organizational capacity:** Were there organizational systems in place to support the program/intervention needs?

~ Evaluation; Process~
~ RQ1a. Why and How? ~
~ RQ1b. Networks ~

For peer review only - http://bmjopen.bmj.com/site/about/guidelines.xhtml

Date: ___________________________ *Interview Code:* ______ - ______ - ______

b. **Communication:** How does the immunization program maintain community support or awareness of the issue of childhood vaccines?<br>***~ Evaluation; Process ~***<br>*~ RQ3. Information Systems ~*<br>*~ RQ4. Intent/Demand for Vaccines ~*<br>*~ RQ5. Knowledge and Resource Sharing ~*<br>*~ RQ6. Workforce ~*
Follow-up:<br><br>*To what extent is the immunization program resilient from rumors and misinformation regarding immunizations?*<br>*How could resilience be improved?*
c. **Media:** How is vaccination portrayed and discussed by the media?<br>***~ Outer Setting ~***<br>*~ RQ3. Information Systems ~*<br>*~ RQ4. Intent/Demand for Vaccines ~*<br>*~ RQ5. Knowledge and Resource Sharing ~*
Follow-up:<br><br>• *What role did the media play in improved coverage?*<br>• *How did the media become involved and how was that involvement sustained?*
d. **Strategic planning:** To what extent is there planning for future resource needs?<br><br>***~ Evaluation; Process~***<br>*~ RQ5. Knowledge and Resource Sharing ~*
8. What are the greatest risks to sustained coverage?<br><br>***~ Reflection; Process~***
9. What lessons have you learned from working on/creating the immunization program?<br><br>***~ Evaluation [IP]***

For peer review only - http://bmjopen.bmj.com/site/about/guidelines.xhtml

Date: ___________________________ *Interview Code:* ______ - ______ - ______

**PART IV – CONCLUSION**<br>**<u>Interviewer Script</u>: Before we end this interview, I want to ask if you have any questions, comments, or anything else you think is important for us to know.**<br>***(Allow time for questions, comments).***
1. What lessons have you learned from working on the immunization program?<br>***~ Evaluation; Process~***
2. During the time we were speaking, you mentioned the following people: ________ (*NAME(S)*) who were/are also stakeholders in the immunization program. Can you provide us introductions to these individuals? Who else would you suggest that we speak to about the immunization program?<br>***~ Characteristics of Individuals ~***
3. You also mentioned the following data sources: ________ (*DATA SOURCE NAME(S)*). How can I access this data? Are you able to assist me? Are there other relevant documents you think I should have? How can I access this?
**<u>Interviewer Script</u>: Our next steps in the project are to continue key informant interviews with other stakeholders, and to analyze some of the quantitative data we are collecting.**
4. What level of update/involvement would you like to have in the project moving forward?
**<u>Interviewer Script</u>: Thank you much for your time and for answering my questions today. You have provided great information that will helpful during our research. If there are no other questions or concerns, then I will turn off the recorder, and you are free to leave as you please. Thank you again.**

NOTES:

For peer review only - http://bmjopen.bmj.com/site/about/guidelines.xhtml

FGD Code: ______ - ______ - ______

VACCINE EXEMPLARS – NEPAL
Focus Group Discussion – Female Community Health Volunteers (FCHV)

Objectives:
- To understand the health services characteristics, including vaccine interventions, now and in the past;
- To identify the strategies behind vaccine programs;
- To identify role and responsibilities of the FCHVs in the immunization sector
- To identify FCHV immunization intervention-related motivations
- To understand barriers and facilitators to use of immunization services
- To understand the factors behind different levels of immunization coverage in their community, in the past and present

**PART 1 – BASIC INFORMATION**<br>*Can be asked by facilitator or filled out by note taker.*	
**A01**. **Researcher/Facilitator name:**	
**A01b. Note taker(s) name(s):**	
**A02. Consents provided by all:** Y / N<br><br>Time of consents ____ : ____ am/pm<br>Number of consents: ________	
**A03.** Date (dd/mm/yy): _____ /_____ / _____	**A04a.** Discussion start time ____ : ____ am/pm<br><br>**A04b.** Discussion end time ____ : ____ am/pm
**A05.** Recorder(s) used:	
**A06.** Location:	**A07.** Demographic surveys completed: Y / N

**PART 2a – INTRODUCTION**<br>*Facilitator to read aloud the following script:*

For peer review only - http://bmjopen.bmj.com/site/about/guidelines.xhtml

FGD Code: ______ - ______ - ______

We are gathering today because we are interested in learning what caregivers like you think about childhood vaccines in *[COMMUNITY]*. My name is ______________, and I will be leading the discussion. This is ___________, and they will be taking notes.

We are specifically interested in learning about your thoughts, opinions, experiences, and perceptions related to the vaccination programming in your community. Please answer to the best of your ability. There are no right or wrong answers. Please share your views and opinions even if they are different from others, and you may choose to not answer any questions you are not comfortable with. You will be reimbursed (X) for traveling to this discussion, and food and drinks are provided here today. This should take about an hour and a half to two hours of your time. This discussion will be audio recorded, and used for research at Emory University and Center for Molecular Dynamics, Nepal (CMDN) to understand how Nepal has achieved high vaccination coverage. We will be recording to make sure we have accurately collected all of the information told to us. Is everyone okay with this discussion, and with being recorded?

Before we start the discussion, we have a survey for everyone to fill out. It asks you a few questions about yourself, and about how you feel about vaccines.

Pass out demographic survey; wait to continue with ground rules until all have been completed and handed back to note taker.

PART 2b – GROUND RULES

Wait for all group members to respond to the previous question, and check that all consent forms have been signed. Read aloud the following script:

For our discussion today, we have some ground rules that I would like everyone to follow:

1. Respect all group members, as well as their views and opinions. It is okay to disagree, but do so respectfully.
2. Everyone deserves a chance to speak. Please do not interrupt.
3. Do not use your mobile phones during the focus group discussion.
4. Please avoid side conversations.
5. Whatever is said here today is said in confidence, and should not be discussed outside of this group.

Does everyone agree with these rules? Do you have any you think we should add?

PART 2c – OPENING

Add any suggested rules to the list of ground rules, and wait for agreement on the rules. Read the following script aloud:

Are there any other questions before we begin? If everyone is ready, we will turn on the audio recorder and start the discussion.

Turn on recorder once everyone is ready to start; Let everyone know that you have turned on the recorder.

Now that the recorder is turned on, is everyone okay with me recording our discussion today? To start, let's go around the room and introduce ourselves. What is your name, how did you

For peer review only - http://bmjopen.bmj.com/site/about/guidelines.xhtml

FGD Code: ______ - ______ - ______

get started in this position, and how many years have you been working in *[COMMUNITY]*? How many people do you serve in *[COMMUNITY]?*

**PART 3 – GENERAL HEALTH/HEALTH SYSTEM**<br>*Read the following script aloud:*	
I am now going to ask you some questions about health, and the health system in *[COMMUNITY]*.	
Q1. Where do people in *[COMMUNITY]* receive preventative healthcare?<br><br>*[Intent: Develop understanding of the resources that are available and that are being utilized]*<br><br>*[CFIR – Inner Setting]*	*Probes:*<br>• *And health services?*<br>• *Door to door, health facility, hospital, etc.?*<br>• *Do people use health facilities/hospital services for primary health care or emergency care? Why? How often?*<br>• *What other types of health services do you use?*<br>○ *Traditional healers, family traditions/cures*<br>• *What are the different staff types (nurse, volunteer) that work in the healthcare services?*<br>• *What are these people's responsibilities?*

**PART 4 – FCHV Roles in Vaccinations**<br>*Read the following script aloud:*	
Now, I'd like to ask you all about vaccines – specifically, vaccines that are given to children under 1 year old.	
Q2.What is a FCHV's background and training?	• *What level of education do you have?*<br>• *What was your past experience in community organizations or health care before you became an FCHV?*<br>• *What kind of training do FCHVs receive?*<br>○ *Who trains FCHVs, and how often?*<br>○ *How long of a session do you have?*<br>○ *Is this verbal, do you have written manuals, visual*

For peer review only - http://bmjopen.bmj.com/site/about/guidelines.xhtml

FGD Code: ______ - ______ - ______

	manuals? How do FCHVs practice message delivery?
Q3. Walk me through FCHV responsibilities in the vaccination process.<br><br>*[Intent: Identify current immunization activities and interventions; decision making process; identify key actors in the program]*<br><br>*[CFIR – Intervention, Inner Setting, Process]*	• *What vaccine promotion duties do FCHVs participate in?*<br>• *Is this part of routine immunization services, or an outreach effort?*<br>• *Who do you answer to?*<br>• *What kind of encouragement do you receive from your community/supervisor?*<br>• *How many children/families are FCHVs responsible for?*<br>• *What kind of difficulties do FCHVs have reaching out to families?*<br>• *How do FCHVs in your community reach different families?*<br>• *How far do FCHVs typically travel to reach families?*<br>• *How important is FCHV's work with vaccines relative to their other responsibilities?*<br>• *What kind of evaluation do FCHVs receive? What health indicators do FCHVs report on/are responsible for?*<br>• *What monitoring systems exist to track vaccination coverage?*<br>• *Are there any difficulties in data collection, recording, and/or submitting to the health post? What are they?*<br>• *What system do you use to track and report the number of children vaccinated?*<br>• *How have monitoring systems changed over the time you've been in your position?*<br>• *What are FCHVs' motivations for providing vaccines/increasing coverage?*<br>• *What kind of compensation do you receive for the work you are doing?*
Q4. *Are there any programs or methods that FCHVs utilize to motivate vaccination?*	*Probes:*<br>• *Are these programs/methods national or community determined?*

For peer review only - http://bmjopen.bmj.com/site/about/guidelines.xhtml

FGD Code: ______ - ______ - ______

[Intent: Decision making process; current factors behind immunization]<br><br>*[CFIR – Inner Setting]*	• *Who determines the programming/methods?*<br>• *Who approves the programming/methods?*<br>• *What messages are promoted in this community regarding vaccines?*<br>• *Who formulates the messages?*<br>• *Who approves the messaging?*<br>• *How are these messages communicated to community members?*
Q5. How does *[COMMUNITY]* view vaccines?<br><br>*[Intent: Decision making process; current factors behind immunization; demand-side]*<br><br>*[CFIR – Inner Setting]*	*Probes: What are the main facilitators to people getting vaccines in your community? Examples for reference:*<br>o *Encouragement from health care workers*<br>o *Shame*<br>o *Geography*<br>o *Enforcement*<br>o *Trust of vaccinators/health care workers*<br>o *Messaging/Awareness*<br>o *Community expectation?*<br>o *Seasonality*<br>o *Fundamental right*<br>o *Availability*<br>*What are the main barriers to people getting vaccines in your community? Examples for reference:*<br>o *Geography*<br>o *Mistrust of health care workers*<br>o *Stock out*<br>o *Cold chain*<br>o *Vaccine hesitancy (ex. side effects)*<br>o *Influence of community/religious leaders*<br>o *Seasonality*<br>o *Wastage*<br>• *How are these barriers addressed, if at all?*<br>• *Are there groups less likely to prioritize vaccines? Who and why?*

For peer review only - http://bmjopen.bmj.com/site/about/guidelines.xhtml

FGD Code: ______ - ______ - ______

	• *Are there children who receive infant vaccines (ex. DTP1) but don't receive others (ex. DTP2, DTP3)? Why?*<br>• *How are vaccines prioritized compared to other health interventions?*
Q7. Are vaccines available when and where they are needed?	*Probes:*<br>• *How about staff, syringes and other supplies?*<br>• *In what conditions do vaccines arrive?*<br>• *Where are vaccines stored?*<br>○ *Where is this storage relative to your clinic/where vaccines are administered?*<br>•
Q8. How has the immunization program changed over time?<br><br>*[Intent: Identify past immunization activities and interventions; identify previous key actors in the program; identify change points]*<br><br>*[CFIR – Intervention, Outer Setting, Inner Setting, Process]*	*Probes:*<br>• *What changed?*<br>○ *Demand from community?*<br>○ *Supply?*<br>○ *Improved systems?*<br>○ *Training?*<br>○ *Governmental emphasis?*<br>○ *Increased funding*<br>• *When did this happen? About what year was this?*<br>○ *What other events happened around this time?*<br>▪ *Political*<br>▪ *Infrastructure changes*<br>▪ *Other health issues*<br>• *Can you think of any certain events that increased or decreased vaccinations?*<br>○ *Strikes*<br>○ *Extreme weather*<br>• *How have FCHV's motivations to vaccinate changed over time?*<br>○ *Has this been affected by any certain events? **(NOTE: REFERENCE BACK TO ANSWER TO PREVIOUS PROBE)***

For peer review only - http://bmjopen.bmj.com/site/about/guidelines.xhtml

FGD Code: ______ - ______ - ______

Q9. How have outbreaks of diseases affected mandatory vaccination rates?<br><br>*[Intent: Identify past disease events that may have impacted past vaccination events; identify change points]*<br><br>*[CFIR – Intervention, Outer Setting, Inner Setting, Process]*	*Probes:*<br>• *Did vaccination rates increase or decrease?*
Q10. Can you think of a specific intervention that you believe contributed to increased vaccine coverage?<br><br>*[Intent: Identify past immunization activities and interventions; identify previous key actors in the program; identify change points]*<br><br>*[CFIR – Intervention, Outer Setting, Inner Setting, Characteristics of Individuals, Process]*	*Probes:*<br>• *How was this intervention implemented in your community?*<br>• *What kind of training was received to prepare different people for this intervention?*<br>• *What went well in the implementation process of thisintervention? were the facilitators to implementation?*<br>• *What was challenging in the implementation process of this intervention? What were the barriers to implementation?*
Q11. What was difficult about improving vaccine coverage in this community at that time?<br><br>*[Intent: Identify past immunization activities and interventions; identify previous key actors in the program]*<br><br>*[CFIR – Intervention, Outer Setting, Inner Setting, Characteristics of Individuals, Process]*	*Probes:*<br>• *How did this affect your community?*<br>• *What was implemented to address these specific difficulties?*<br>• *Why do you think this was successful?*<br>• *How were different people motivated for this success?*

**PART 5 – POLICY**	
Q12. What, if any,vaccines do children need to receive?<br><br>*[Intent: Identify current immunization activities and interventions]*	*Probes:*<br>• *Are the requirements clear?*<br>• *What vaccinations are required for children to be allowed to go to school?*

For peer review only - http://bmjopen.bmj.com/site/about/guidelines.xhtml

FGD Code: ______ - ______ - ______

[CFIR – Intervention, Outer Setting, Inner Setting]	• What affect have these laws/requirements had on FCHVs' jobs?
Q13. How have these requirements changed over time?<br><br>*[Intent: Identify past immunization activities and interventions; identify previous key actors in the program; identify change points]*<br><br>*[CFIR – Intervention, Outer Setting, Inner Setting, Process]*	*Probes:*<br>• *Why did requirements change?*<br>• *About what year/time was this?*<br>• *What other events happened around this time?*<br>• *Who worked with your community to adapt to the new regulations?*<br>• *What kind of assistance/training was received to enable you to deliver according to new requirements?*

**PART 4 – CLOSING**<br>*Read the following script aloud:*
Those are all of my questions. What other information about vaccinations do you have that you feel would be important for me to know? Do you have any other questions or comments you'd like to add before we end the discussion?<br><br>*Wait for group to answer.*<br><br>Thank you all very much for your time and for answering my questions today. You have all provided great information that will be of help during our research. If you have any other questions or concerns, please feel free to contact ____________. You can also find this information on the consent document that you signed when we started the discussion. If there are no other questions or concerns, then I will turn off the recorder, and we will give travel reimbursement. Thank you again for your help.

For peer review only

For peer review only - http://bmjopen.bmj.com/site/about/guidelines.xhtml

Key Informant/In-depth Interview Guide-Community Leaders

Objectives:

- To understand their role, if any, as it relates to health interventions, specifically immunization coverage
- To identify key community stakeholders who have a role in immunization programming
- To understand the cultural context and environmental context of the selected community
- To understand what drives community acceptance of immunizations
- To understand the motivations of key community stakeholders in vaccine intervention characteristics during country-specific change points in DTP1/DTP3 coverage
- To identify the strategies behind current immunization programs to inform current context
- To identify previous immunization activities — including specific interventions, delivery systems, policies, facilitators and barriers — that were key during change points in the DTP1/DTP3 curve
- To understand specific barriers and facilitators to implementation of interventions in the immunization sector

For peer review only

**PART 1 – DEMOGRAPHIC AND BASIC INFORMATION**<br>*Can be asked by interviewer or filled out by note taker.*	
**A01**. **Researcher/Interviewer name:**	
**A01b. Note taker(s) name(s):**	
**A01c. Translator name:**	
**A01d. Copy belongs to:**	
**A02. Consent given:** Y / N<br><br>Time of consent ____ : ____ am/pm<br>Time zone: _____	
**A03.** Date (dd/mm/yy): _____ /_____ / _____	**A04a.** Interview start time ____ : ____ am/pm<br>**A04b.** Interview end time ____ : ____ am/pm<br>Time zone: _____<br><br>**A05**. Recorder(s) used:
**A06.** Name of Respondent interviewed:	**A07.** Designation/title of Respondent interviewed:
**A08.** Organization name:	**A09.** Years in Organization:

PART 2 – INTERVIEW INTRODUCTION

<u>Interviewer Script</u>: Thank you very much for your willingness to meet with us today and share your experience and knowledge regarding the immunization program in ______ (COUNTRY). I'd like to begin by learning about you and your role related to the immunization program.

1. Can you tell me about your role in ______ (THIS COMMUNITY) ?

~ Individual Setting ~

Follow up:

- *How does your role relate to health care programming?*
- *How does your role relate to the immunization program?*
- *Responsibilities (past and present)*
- *What are the contributions you feel you've made in the immunization programming in your community?*
- *What has motivated you to make these contributions?*

2. Who are other community leaders/stakeholders who have had an impact on the immunization program?

~ Inner Setting ~

Follow up:

- *What role did they play?*
- *What were their key contributions?*
- *What motivated their involvement?*
- *What were their top three incentives for performing well?*
- *What are the stakeholders' party affiliation?*
- *Are their positions relevant to vaccinations?*

PART 3 – HEALTH SYSTEM & VACCINES

Read the following script aloud:

<u>Interviewer Script</u>: Now I'd like to learn about the health care services in your community, and how immunization coverage plays a part. I'd also like to hear a bit more about community attitudes towards immunizations.

3. How does *[COMMUNITY]* view vaccines?

[Intent: Decision making process; current factors behind immunization; demand-side]

~ Inner Setting ~

For peer review only

~ Evaluation; Process ~
• *Probes: What are the main facilitators to people getting vaccines in your community]?*<br>○ *Encouragement from health care workers*<br>○ *Shame*<br>○ *Geography*<br>○ *Enforcement*<br>○ *Trust of vaccinators/health care workers*<br>○ *Messaging*<br>○ *Community expectation?*<br>○ *Seasonality*<br>• *What are the main barriers to people getting vaccines in your community?*<br>○ *Geography*<br>○ *Mistrust of health care workers*<br>○ *Stock out*<br>○ *Cold chain*<br>○ *Vaccine hesitancy (ex. side effects)*<br>○ *Influence of community/religious leaders*<br>○ *Seasonality*<br>○ *Wastage*<br>• *How are these barriers addressed?*<br>• *Are there groups less likely to prioritize vaccines? Who and why?*<br>• *Are there children who receive infant vaccines (ex. DTP1) but don't receive others (ex. DTP2, DTP3)? Why?*<br>*How are vaccines prioritized compared to other health interventions/measures?*
4. How have outbreaks of diseases affected mandatory vaccination rates?<br>***~ Evaluation; Process ~***<br>*~ RQ3. Information Systems ~*<br>*~ RQ4. Intent/Demand for Vaccines ~*
Probes:<br>*Did vaccination rates increase or decrease?*
5. Compared to other government responsibilities - like public safety, roads and infrastructure, education – how important are vaccines?<br>*~Intervention; Outer Setting~*
Probes:<br>• *Why are they important/not important?*<br>• *What is the most important to you / [COMMUNITY]?*<br>• *What is the least important to you / [COMMUNITY]?*<br>• *Who do you believe is responsible for providing vaccines?*<br>○ *Government?*

For peer review only

NGOs and aid agencies?

6. How has the *[COMMUNITY'S]* ability to provide vaccines changed over time?

~Intervention; Inner setting~

Probes:

- *What changed?*
    - *Demand from community?*
    - *Supply?*
    - *Improved systems?*
    - *Training?*
    - *Governmental emphasis?*
- *About what year was this?*
    - *What other events happened around this time?*
        - *Political*
        - *Infrastructure changes*
        - *Other health issues*
- *Can you think of any certain events that increased or decreased vaccinations?*
    - *Strikes*
    - *Extreme weather*
    - *Outbreaks*
        - *What diseases?*
            - *Vaccine preventable (flu, measles, mumps, rubella, pertussis etc.)*

Non-vaccine preventable?

7. What are specific interventions/programming shifts that have happened in the last ten years (NOTE: depending on how long they have been in their positions)?

[Intent: Identify past immunization activities and interventions; identify previous key actors in the program; identify change points]

~ Evaluation; Process~

[CFIR – Intervention, Outer Setting, Inner Setting, Characteristics of Individuals, Process]

Probes:

- *What intervention had the greatest impact?*
- *How was this intervention implemented in your community?*
- *How did people react to this intervention?*
- *What were the facilitators to uptake?*
- *What were barriers?*
-

For peer review only

8. What factors have contributed to the sustainability of the immunization program?<br><br>***~ Evaluation; Process~***
a. Organizational capacity: Were there organizational systems in place to support the program needs?<br><br>***~ Evaluation; Process~***<br>*~ RQ1a. Why and How? ~*<br>*~ RQ1b. Networks ~*
b. Communication: How does the immunization program maintain community support or awareness of the issue of childhood vaccines?<br><br>***~ Evaluation; Process ~***<br>*~ RQ3. Information Systems ~*<br>*~ RQ4. Intent/Demand for Vaccines ~*<br>*~ RQ5. Knowledge and Resource Sharing ~*<br>*~ RQ6. Workforce ~*
Follow-up:<br><br>*To what extent is the immunization program resilient from rumors and misinformation regarding immunizations?*<br>*How could resilience be improved?*
c. Media: How is vaccination portrayed and discussed by the media?<br><br>***~ Outer Setting ~***<br>*~ RQ3. Information Systems ~*<br>*~ RQ4. Intent/Demand for Vaccines ~*<br>*~ RQ5. Knowledge and Resource Sharing ~*
Follow-up:<br><br>• *What role did the media play in improved coverage?*<br>• *How did the media become involved and how was that involvement sustained?*
d. Strategic planning: To what extent is there planning for future resource needs?<br><br>***~ Evaluation; Process~***<br>*~ RQ5. Knowledge and Resource Sharing ~*
9. What are the greatest risks to sustained coverage?<br><br>***~ Reflection; Process~***

For peer review only

10. What lessons have you learned from working on/creating the immunization program?<br><br>*~ Evaluation [IP]*
**PART 4 – CLOSING**<br>*Read the following script aloud:*
Those are all of my questions. Do you have any other questions or comments you'd like to add before we end the discussion?<br><br>*Wait for individual to answer.*<br><br>Thank you much for your time and for answering my questions today. You have provided great information that will helpful during our research. If you have any other questions or concerns, please feel free to contact _____________. You can also find this information on the consent document that you signed when we started the discussion. If there are no other questions or concerns, then I will turn off the recorder, and you are free to leave as you please. Thank you again.

For peer review only

FGD Code: ______ - ______ - ______

VACCINE EXEMPLARS
Focus Group Discussion – Parents/Caregivers

Note: Do not read red italics aloud

Objectives:
- To understand the vaccine intervention characteristics during country-specific change points in DTP1/DTP3 coverage
- To identify parental/caregiver motivation behind interventions during the change point within the immunization program
- To explore decision-making process of parents/caregivers in the change point
- To identify previous immunization activities - including specific interventions, delivery systems, policies, facilitators and barriers – that were key during change points in the DTP1/DTP3 curve and to understand barriers and facilitators to implementation
- To understand the current factors behind different levels of immunization coverage in their community
- To understand the factors behind different levels of immunization coverage in their community at change point

For peer review only

**PART 1 – BASIC INFORMATION**<br>*Can be asked by facilitator or filled out by note taker.*	
**A01. Researcher/Facilitator name:**	
**A01b. Note taker(s) name(s):**	
**A01c. Translator or observer:**	
**A01d. Copy belongs to:**	
**A02. Consents provided by all:** Y / N<br><br>Time of consents ____ : ____ am/pm<br>Number of consents: _______	
**A03.** Date (dd/mm/yy): _____ /_____ / _____	**A04a.** Discussion start time ____ : ____ am/pm<br><br>**A04b.** Discussion end time ____ : ____ am/pm<br><br>Time zone: _____
**A05.** Group (check all that apply):<br>☐ Mothers (under age 25)<br>☐ Mothers/Grandmothers (over age 25)<br>☐ Fathers<br>☐ Other - _____________	**A06.** Recorder(s) used:
**A07.** Location:	**A08.** Demographic surveys completed: Y / N

For peer review only - http://bmjopen.bmj.com/site/about/guidelines.xhtml

FGD Code: ______ - ______ - ______

**PART 2a – INTRODUCTION**<br>*Facilitator to read aloud the following script:*
We are gathering today because we are interested in learning what caregivers like you think about childhood vaccines in *[COMMUNITY]*. My name is ______________, and I will be leading the discussion. This is ___________, and they will be taking notes.<br><br>We are specifically interested in learning about your thoughts, opinions, experiences, and perceptions related to the vaccines your child/grandchild has or has not received. Please answer to the best of your ability. There are no right or wrong answers. Please share your views and opinions even if they are different from others, and you may choose to not answer any questions you are not comfortable with. You will be reimbursed (X) for traveling to this discussion, and food and drinks are provided here today. This should take about an hour to an hour and a half of your time. This discussion will be audio recorded, and used for research at Emory University and *[LOCAL PARTNER]* to understand how *[COUNTRY]* has achieved high vaccination coverage. We will be recording to make sure we have accurately collected all of the information told to us. Is everyone okay with this discussion, and with being recorded?<br><br>Before we start the discussion, we have a survey for everyone to fill out. It asks you a few questions about yourself, and about how you feel about vaccines.<br><br>*Pass out demographic survey; wait to continue with ground rules until all have been completed and handed back to note taker.*
**PART 2b – GROUND RULES**<br>*Wait for all group members to respond to the previous question, and check that all consent forms have been signed. Read aloud the following script:*
For our discussion today, we have some ground rules that I would like everyone to follow:<br><br>1. Respect all group members, as well as their views and opinions. It is okay to disagree, but do so respectfully.<br>2. Everyone deserves a chance to speak. Please do not interrupt.<br>3. There are no right or wrong answers.<br>4. Please avoid side conversations.<br>5. Whatever is said here today is said in confidence, and should not be discussed outside of this group.<br><br>Does everyone agree with these rules? Do you have any you think we should add?
**PART 2c – OPENING**<br>*Add any suggested rules to the list of ground rules, and wait for agreement on the rules. Read the following script aloud:*
Are there any other questions before we begin? If everyone is ready, we will turn on the audio recorder and start the discussion.<br><br>*Turn on recorder once everyone is ready to start; Let everyone know that you have turned on the recorder.*<br><br>Now that the recorder is turned on, is everyone okay with me recording our discussion today? To start, let's go around the room and introduce ourselves. What is your name, \how many children do you have, and what are their ages?

For peer review only - http://bmjopen.bmj.com/site/about/guidelines.xhtml

FGD Code: ______ - ______ - ______

**PART 3 – GENERAL HEALTH**<br>*Read the following script aloud:*	
I am now going to ask you some questions about health in general, and health in *[COMMUNITY]*.	
Q1. Please tell me about how people in *[COMMUNITY]* receive healthcare and health services?<br><br>*[Intent: Develop understanding of the resources that are available and that are being utilized; health literacy; general health interventions]*	*Probes:*<br>• *Local health clinic, nurse, doctor, community health workers?*<br>• *Friends and family?*<br>• *Where do you seek healthcare?*<br>○ *Why?*<br>• *Do you go to prevent disease, or only when you are sick?*<br>○ *Why?*<br>• *When do you seek health related information?*<br>○ Why?
Q2. How does *[COMMUNITY]* help families to stay healthy?<br><br>*[Intent: Develop understanding of the resources that are available and that are being utilized; health literacy; general health interventions]*	*Probes:*<br>• *What are the health activities you take part in within [COMMUNITY]?*<br>• *What are the health activities your family members take part in within [COMMUNITY]?*<br>• *What do you believe your role is in regards to vaccinations?*

**PART 4 – VACCINES**<br>*Read the following to participants:*	
We are now going to talk about vaccines. Specifically, the vaccines your child was given before they were 2 years old.	
Q3. What has *[COMMUNITY]* been told about vaccines?<br><br>*[Intent: Health literacy; decision making process of parents; motivation]*	*Probes:*<br>• *How did you get this information?*<br>○ *Mass media*<br>○ *Verbally (family, friends, community health worker, etc.)*<br>○ *Paper form*<br>• *What do you think about this information?*<br>• *How much do you trust the source of vaccination information?*<br>○ *Why?*

For peer review only - http://bmjopen.bmj.com/site/about/guidelines.xhtml

FGD Code: ______ - ______ - ______

Q4. What has *[COMMUNITY]* been told about the diseases vaccines prevent?<br><br>*[Intent: Health literacy; decision making process of parents; motivation]*	*Probes:*<br>• *How does your knowledge of this disease make you feel about vaccines (in general, or the vaccine that prevents the disease)*<br>• *Do you know what this disease is?*<br>• *Have you seen anyone get this disease before?*<br>• *Who told you about these diseases?*<br>• *Do people get this disease still?*
Q5. What do [*PROVIDERS*] at [*CLINIC*] tell mothers/caregivers about vaccinations?<br><br>*[Intent: Characteristics of community possibly leading to decision making process]*	*Probes:*<br>• *What vaccinations do providers talk about when you go to the clinic?*<br>• *Who else talks about vaccines?*<br>○ *Community health workers*<br>○ *Community leaders*<br>○ *Religious leader*<br>○ *Family (who)?*<br>○ *Friends, etc.?*
Q6. What affects mothers/caregivers decision to vaccinate or not to vaccinate their child?<br><br>*[Intent: Demand-side factors/motivation; decision making process]*	*Probes:*<br>• *How is this received by other community members?*<br>• *Is this needed for school?*<br>• *What are [COMMUNITY] beliefs surrounding vaccination?*<br>• *How convenient is it for caregivers to vaccinate children?*<br>• *Do you know of any leaders/movements/organizations that promote vaccination?*<br>○ *Any that oppose vaccination?*<br>• *Is it expensive to get vaccinated?*<br>• *When you go for vaccines, do you get other services or goods?*<br>○ *Examples – food, supplies for children, check-ups, etc.*
Q7. How important is it in *[COMMUNITY]* to get vaccinations for children?	*Probes:*<br>• *Why/why not?*
Q8. Tell me about a normal visit to get children vaccinated in *[COMMUNITY]*.<br><br>*[Intent: Find possible interventions in place within the community]*	*Probes:*<br>• *How far did you have to travel?*<br>○ *What kind of transportation did you use (walk, drive, bus, etc.)*<br>• *Did you have to present any documentation?*<br>• *About how long did getting your child vaccinated take?*<br>• *Were more than one of children getting vaccinated? Why/why not?*

For peer review only - http://bmjopen.bmj.com/site/about/guidelines.xhtml

FGD Code: ______ - ______ - ______

	• *How much time did it take to get to the vaccination site?*<br>• *How much of this time was spent waiting in line?*<br>• *How did this affect your daily routine?*<br>• *Was this a special vaccination day?*<br>○ *How did you know about this?*<br>○ *Are there any reminder systems?*<br>▪ *How do they work?*<br>▪ *Do you pay attention to these reminders?*<br>▪ *When did these reminders start?*<br>○ *Who told you about this?*

**PART 4 – HISTORY AND POLICY**	
Q9. Have the opinions of caregivers/mothers in *[COMMUNITY]* changed over time?<br><br>*[Intent: Develop entire history; set up a timeline of how vaccination has changed]*	*Probes:*<br>• Why?<br>• *What changed for you?*<br>• *About what year was this?*<br>○ *What other events happened around this time?*
Q10. What requirements are there for childhood vaccines?<br><br>*[Intent: Knowledge of policy and policy implications]*	*Probes:*<br>• *How did you learn about these requirements?*<br>• *Have you ever had to present your child's vaccination record to have access to services?*<br>○ *What vaccinations are required for children to be allowed to go to school?*<br>• *How do you feel about these laws/requirements?*<br>• *What affect have these laws/requirements had on your life?*
Q11. Compared to other government responsibilities - like public safety, roads and infrastructure, education – how important are vaccines?	*Probes:*<br>• *Why are they important/not important?*<br>• *What is the most important to you / [COMMUNITY]?*<br>• *What is the least important to you / [COMMUNITY]?*<br>• *Who do you believe is responsible for providing vaccines?*<br>○ *Government?*<br>○ *NGOs and aid agencies?*

For peer review only - http://bmjopen.bmj.com/site/about/guidelines.xhtml

FGD Code: ______ - ______ - ______

**PART 4 – CLOSING**<br>*Read the following script aloud:*
Those are all of my questions. Do you have any other questions or comments you'd like to add before we end the discussion?<br><br>*Wait for group to answer.*<br><br>Thank you all very much for your time and for answering my questions today. You have all provided great information that will helpful during our research. If you have any other questions or concerns, please feel free to contact _____________. You can also find this information on the consent document that you signed when we started the discussion. If there are no other questions or concerns, then I will turn off the recorder, and you are free to leave as you please. Thank you again.

For peer review only

Appendix 3 – Reflexivity Statement

1. How does this study address local research and policy priorities?
To address local needs, the Emory study team partnered with local research groups in Nepal, Zambia, and Senegal. This partnership ensured the study was aligned with the context and priorities of each country. Data collection tools were iterative and were created with advice and input from both in-country researchers, local stakeholders, and external technical advisors. The tailoring of the project to each country allowed for this hypothesis generating study to best reflect the main needs and priorities of the countries surveyed.

2. How were local researchers involved in study design?
Local researchers were key to the realization of this project. The in-country study teams helped draw our KII list of key stakeholders and past influential leaders, along with helping to secure meetings. We worked with the local teams to develop our tools, and to edit the questions or procedures as needed based on cultural and political considerations. Partners also helped to determine the best locations for our sample and were responsible for finding individuals for FGDs [1].

3. How has funding been used to support the local research team?
The local research teams were treated as sub-contractors on this project and were paid fairly for their time and effort on this project. We did not have any free or un-funded contributions of local researchers on behalf of the project.

4. How are research staff who conducted data collection acknowledged?
Research staff who conducted data collection were acknowledged as authors in their respective country case studies [2-4].

5. Do all members of the research partnership have access to study data?
In-country research partners have access to all KII and FGD transcripts from their respective countries.

6. How was data used to develop analytical skills within the partnership?
While we did not focus on capacity building during this study, the Emory research staff conducted trainings with each in-country research group. These trainings were over 7 – 14 days, and reviewed the study protocols, data collection guidelines, and general qualitative research best practices.

7. How have research partners collaborated in interpreting study data?
Within each country level case study, our in-country partners were actively engaged in interpretations; the study teams had weekly meetings to discuss the progress and findings of KIIs and FGDs. In addition, partners were involved in the drafting of the manuscripts – the case studies and this cross-cutting synthesis [2-4].

8. How were research partners supported to develop writing skills?
Our research partners collaborated on the manuscript drafts and were added as authors for their contributions. Additionally, several student graduate assistants were not American, and from LMICs; students were added as authors for their contributions.

9. How will research products be shared to address local needs?
Results from this study have already been disseminated to the respective leaders and stakeholders of each country; the meeting was led by the in-country PI and was attended by the Emory research team. A country-specific report, along with the manuscripts, were disseminated. We met with our TAG to disseminate results to global immunization leaders, and continue to collaborate with the in-country PIs on opportunities for additional outreach.

10. How is the leadership, contribution and ownership of this work by LMIC researchers recognised within the authorship?

On this cross-country synthesis, the in-country PIs are added as senior authors. For the individual case studies, all in-country researchers are included as authors [2-4].

11. How have early career researchers across the partnership been included within the authorship team?

In country research partners hired their own staff, and there were different levels of experience represented across the researchers. Early career researchers were included and mentored within each in-country research team, as well as within the Emory team.

12. How has gender balance been addressed within the authorship?

While the Emory and in-country PI's are men, most of the research staff was comprised of women. Guidance for authorship was drafted prior to writing manuscripts, and all were given opportunities to contribute. Those who met the criteria were included as authors; individual contributions, and specific authors, are included on the country case studies [2-4]. This manuscript is the synthesis, and country-level PIs and the Emory team are included as authors.

13. How has the project contributed to training of LMIC researchers?

This project had 7 – 14 day trainings for each country's research team, wherein best practices for qualitative research were taught along with the study's aims and tools. In-country PI's provided additional assistance and training to staff as needed, however, our collaborators were all well-trained professionals and knowledgeable in their areas of expertise.

14. How has the project contributed to improvements in local infrastructure?

This project was a hypothesis generating study, and therefore did not contribute to infrastructure improvement. However, we hope the findings of this study will be of use to program managers and funders in strengthening and building further immunization infrastructure that is both resilient and sustainable.

15. What safeguarding procedures were used to protect local study participants and researchers?

KII and FGD participants gave researchers signed, informed consent; their information was transcribed and de-identified. See our protocol and case study papers for further information [1-4]. There was only minimal risk to both participants and researchers; local PI's were responsible for any context specific safeguarding.

For peer review only

1. Bednarczyk, R.A., et al., *Exemplars in vaccine delivery protocol: a case-study-based identification and evaluation of critical factors in achieving high and sustained childhood immunisation coverage in selected low-income and lower-middle-income countries.* BMJ Open, 2022. **12**.
2. Micek, K., et al., *Critical success factors for routine immunization performance: A case study of Zambia 2000 to 2018.* Vaccine X, 2022. **11**.
3. Hester, K.A., et al., *Critical success factors for high routine immunization performance: A case study of Nepal.* Vaccine X, 2022. **12**(100214).
4. Sakas, Z., et al., *Critical success factors for high routine immunization performance: A case study of Senegal.* medRxiv, 2022: p. 2022.01.25.22269847.

For peer review only

Additional key informant quotes, by country and domain, illustrating drivers for vaccine coverage from the conceptual framework [15-17]

Domain	Nepal	Senegal	Zambia
Governance and financing<br><br>The processes that guide policies and operations within the health system, including collaboration (effective working relationships, including alignment of priorities) and commitment (dedication to activities and responsibilities).	*"A provision was made in the constitution, and [vaccines] became a fundamental right, so the government has a responsibility to provide. And management of currently running programs also [focuses on] children who have missed vaccines." (Nepal Health Research Council Staff)*	*"When we have a strategic document to work on, the partners get involved according to their area of expertise, and we work together when it comes to implementing and monitoring the vaccine program. In this teamwork, you cannot tell who is the partner or who is from the Ministry of Health, because we work hand in hand. We are in the same boat, we want to have success." (External partner at the national level)*	*"Once you have identified that there is an issue that needs attention, these policies are revised and updated so that they match what is current. [Policies] are more or less like living documents to match the prevailing circumstances. Our planning has improved quite a lot because it's now bottom-up. So, we are the ones that have a lot of input in these documents." (Provincial Health Officer)*
Health systems strengthening<br><br>Strategies that are designed to sustainably improve health system performance (across all sectors) through an integrated and horizontal approach, with a focus on equity.	*"No wards will be deprived of health centers, and if the health center isn't enough to provide health services, then village clinics, immunization centers (outreach clinics), and urban health clinics will be established. If services are provided from these health centers, quality of the vaccines are maintained, and health centers are accessible to all - then good immunization coverage can be achieved." (Department of Health Services within the MOH)*	*"We tried to see what was hidden behind the weak coverage - especially the poor availability of resources including health structures and sufficient staff... a diagnosis was made to improve the indicators, health structures, i.e. creating a lot of health posts, and recruiting staff to be set up in each health post, or at least one vaccination unit to really try to reach out to the entire population in order to have good coverage." (National level stakeholder)*	*"Previously, there was no public health department at a grassroots level, it was just at the National level... So, now the Public Health Specialist is on the ground to try to see that this primary health care activity is promoted. And I think with that supervision, with the introduction of the public health nurses, Health promotion departments, I think these interventions couldn't be achieved higher in terms of immunization activities." (District Health Director)*
Community engagement<br><br>Responsibility of the community to implement vaccination program activities through local leadership and volunteers, fostered by government initiatives and cultural values.	*"Mothers' groups are responsible to give education and information to the parents.... If they don't support the program, then it won't be successful. They are the ones who build awareness and give knowledge and information to the people at the grassroots level." (Department of Health Services in the Ministry of Health and Population)*	*"Community health workers play a very important role – they are in direct contact with the population. They are the ones who live in the community, and they identify more with the community. So, there is a relationship of trust between them and the population." (National level government stakeholder)*	*"We make sure that the community is fully engaged and aware of the benefit of ensuring that their children are vaccinated. It is important that we ensure that within the community, the key gatekeepers are helping us." (Provincial Health Officer)*

For peer review only

Adaptive capacity<br><br>Ability to sufficiently consider contextual factors, supported by timely evaluation of quality data, to tailor vaccine programming based on local needs and priorities.	*"Using HMIS, which is our biggest data source, we send daily, weekly, or monthly reports to districts using phones. We visit districts and contact the responsible person. We understand the situation and we try to solve those problems. We don't depend on the system. We explore, analyze, and address if there are issues outside the system. We do review meetings at the end of the year from the lessons we learned. We also make periodic updates." (Regional MoH Director)*	*"During regional-level meetings, we hear good ideas from the districts. When Dakar had a measles epidemic, we brought together all the districts to tell us how to plan in order to have good measles vaccine coverage. Some of the districts came up with strategies that we found very inspiring. One of them was to hold the vaccination sessions beyond regular work hours to reach the people who are not free during the day. The other strategy was to organize sessions on weekends. As soon as we put these strategies in place, we noticed that the coverage increased right away. It dawned on us that the parents did not refuse the vaccination but that they were busy by their daily occupations." (External partner at the national level)*	*"We have been advising facilities that they need to own the data; they have to make sure that they utilize it by analyzing... If they are not meeting their targets, they need to reflect on the data and see where they are going wrong... For those [health facilities] that are performing well, we find out what they are doing, and we try to replicate to the other facilities that are not doing as well." (District Health Officer)*

For peer review only

Critical success factors for high routine immunization performance –

Standards for Reporting Qualitative Research (SRQR) checklist

http://www.equator-network.org/reporting-guidelines/srqr/

Title and abstract

	Page/line no(s).
**Title** - Critical success factors for high routine immunization performance: Qualitative analysis of interviews and focus groups from Nepal, Senegal, and Zambia	Page 1; 1-2
**Abstract** -<br>INTRODUCTION: Vaccination averts an estimated 2-3 million deaths annually. Although vaccine coverage improvements across Africa and South Asia have remained relatively stagnant and below global targets, several countries have outperformed their peers with significant increases in routine immunization coverage. Examining these countries' vaccination programs provides an opportunity to identify and describe critical success factors that may have supported these improvements.<br><br>METHODS: We selected three high-performing countries in regard to childhood vaccination: Nepal, Senegal, and Zambia. This multiple case study analysis was conducted using data from the Exemplars in Vaccine Delivery project within the Exemplars in Global Health program. We used qualitative analysis to investigate factors that contributed to high vaccination coverage through 66 key informant interviews (KIIs) and 22 focus group discussions (FGDs) at the national, regional, district, health facility, and community levels – with a total of 678 participants. Qualitative data were collected from October 2019 to April 2021. We triangulated these findings with quantitative analyses using publicly available data, which are published elsewhere.<br><br>RESULTS: Our data revealed that the critical success factors for vaccine programming relied on the cultural, historical, and statutory context in which the interventions were delivered. In Nepal, Senegal, and Zambia, high immunization coverage was driven by 1) strong governance structures and healthy policy environments; 2) adjacent successes in health systems strengthening; 3) government-led community engagement initiatives; and 4) adaptation considering contextual factors at all levels of the health system.<br><br>CONCLUSION: Throughout the study, our analysis returned to the importance of defining and understanding the context, governance, financing, and health systems within a country, rather than focusing on any one intervention. This paper augments findings from existing literature by highlighting how contextual factors impact implementation decisions that have led to improvements in childhood vaccine delivery. Findings from this research may identify transferable lessons and support actionable recommendations to improve national immunization coverage in other settings.	Page 1; 23 -51

Introduction

**Problem formulation** –<br>DESCRIPTION AND SIGNIFICANCE OF THE PROBLEM/PHENOMENON STUDIED:<br>Lines 109 – 110; "Vaccination is recognized as one of the most influential public health interventions, averting an estimated 2-3 million deaths annually [1-4]."<br>Lines 122 – 125; "Within both [WHO] regions, several countries outperformed their peers with significant increases in routine immunization coverage since 2000 [10]. Examining these countries' vaccination programs provides an opportunity to identify and describe critical success factors that may have supported these improvements."<br><br>REVIEW OF RELEVANT THEORY AND EMPIRICAL WORK:<br>Lines 127 – 131; "The essential components of an effective vaccine delivery system are well-established and include strong governance and leadership, healthcare financing, human resources, a robust supply chain, community engagement, and information systems for evidence-based decision-making [11-13]. Research on behaviors related to routine	Page 3

For peer review only - http://bmjopen.bmj.com/site/about/guidelines.xhtml

immunization focuses on recognized determinants of coverage, including intent to vaccinate, community access, and health facility readiness [14]."<br><br>PROBLEM STATEMENT:<br>Lines 131 – 134; "Although existing literature describes vaccine delivery systems, there is a gap in knowledge related to how programs and policies are implemented, how strategies are operationalized, and how context influences programming."	
**Purpose or research question –**<br>"The purpose of this study was to identify critical success factors that contributed to catalytic growth in childhood routine immunization coverage. Findings from this research may identify transferable lessons and support actionable recommendations to improve national immunization coverage in other settings [20]."	Page 3; 145 - 148

Methods

**Qualitative approach and research paradigm –**<br>APPROACH -<br>"We used qualitative analysis to investigate factors that contributed to high vaccination coverage through key informant interviews (KIIs) and focus group discussions (FGDs) at the national, regional, district, health facility, and community levels. We triangulated these findings with quantitative analyses using publicly available data, which are published elsewhere and referenced throughout this paper [10, 21].<br><br>Prior to data collection, we developed a conceptual model (Figure 1) to organize factors that impact childhood vaccine coverage globally. This model was based on the work of Phillips et al. and LaFond et al. alongside a broader review of the vaccine literature [14, 22]. Details regarding the development of this model are available elsewhere [10]."<br><br>PARADIGM –<br>"The interview guides were informed by the Consolidated Framework for Implementation Research (CFIR) [25] and the Context and Implementation of Complex Interventions (CICI) framework [26]."	Page 4; 175 – 17 183<br>Page 5; 203-205
**Researcher characteristics and reflexivity –**<br>Reflexivity statement provided as an appendix.	Appendix 3
**Context –**<br>"Qualitative data were collected between October 2019 and April 2021 at the national, regional, district, health facility, and community levels in Nepal, Senegal, and Zambia (Table 1)."<br><br>*Case studies in Nepal, Senegal, and Zambia are cited in text and provide additional details on context in each country.	Page 4; 202 - 203<br><br>Page 6; 251 - 254
**Sampling strategy –**<br>"Our sampling approach aimed to include a diverse sample of participants regarding geographic location and demographic qualities. Caregivers and volunteer community health workers were recruited for FGDs from health facility catchment areas with the assistance of local health staff."	Page 5; 217 - 220
**Ethical issues pertaining to human subjects –**<br>See section 2.4 "Ethical approval" and "Ethics approval statement".	Page 7; 271 – 279<br><br>Page 15; 618 - 625
**Data collection methods -**<br>"Qualitative data were collected between October 2019 and April 2021 at the national, regional, district, health facility, and community levels in Nepal, Senegal, and Zambia (Table 1). The interview guides were informed by the Consolidated Framework for Implementation Research (CFIR) [25] and the Context and Implementation of Complex Interventions (CICI) framework [26]. KII and FGD guides were translated into local languages by research assistants from the	Page 4 – 5; 202 - 223

For peer review only - http://bmjopen.bmj.com/site/about/guidelines.xhtml

respective countries. All interview guides were piloted before use and adjusted iteratively throughout data collection. For each country, an initial list of KIIs was developed with local research partners and MoH officials with snowball sampling used to identify additional key informants. Our sampling approach aimed to include a diverse sample of participants regarding geographic location and demographic qualities. Caregivers and volunteer community health workers were recruited for FGDs from health facility catchment areas with the assistance of local health staff. The duration of KIIs and FGDs averaged one and a half hours. KIIs and FGDs were audio-recorded with the permission of participants. Research files, recordings, and transcriptions were de-identified and password protected. Data analysis processes were generally consistent in all three countries examined in order to allow for thoughtful comparison."	
**Data collection instruments and technologies -**<br>See above quote.	Page 4- 5; 202 - 223
**Units of study** –<br>See Table 1.	Page 6; 251 - 254
**Data processing** –<br>See section 2.2 "Qualitative data collection and analysis": due to the nature of the multiple case study, aspects of this are addressed throughout this part of the text.	Page 4- 5; 202 – 242
**Data analysis** –<br>See section 2.2 "Qualitative data collection and analysis": due to the nature of the multiple case study, aspects of this are addressed throughout this part of the text.	Page 4- 5; 202 – 242
**Techniques to enhance trustworthiness** –<br>"We applied a theory-informed thematic analysis of the transcripts to identify critical success factors. Data analysis was initially conducted individually for each case study, with separate members of our research team leading analysis for each country. However, to ensure consistency and coherence, all data analysts were involved in the review, coding, and summarization of data for all three case studies. We met frequently as a team to discuss overlap and to compare empirical findings. Transcripts were coded and analyzed using MaxQDA2020 software (Berlin, Germany). Coding was completed by researchers at Emory, some of whom also developed and revised the codebook throughout. Coding meetings were held frequently to discuss code definitions and to discuss emerging themes. Researchers from in-country teams were involved through additional meetings to discuss data and ground-truth findings. We considered setting and participant roles while identifying key points and further contextualized data using historical documents and an a priori literature review. Qualitative data collection and analysis tools, including topic guides and codebooks, can be found on our Open Sciences Framework (OSF) page [27]."<br><br>"Upon completion of each country case study, findings were disseminated to in-country experts and country governments, along with the TAG, allowing for the study team to ground-truth results. The research team at Emory University co-hosted regular meetings with the in-country research partners, experts in a variety of relevant fields, government officials, and other advisors to ask for feedback and contextualization. Researchers would present on methods, findings, and implications following an engaging discussion with advisors that would lead to improvements in our data analysis processes or presentation of findings. Revisions would be distributed through follow-up email conversations and subsequent phone calls with select advisors."	Page 5; 225 – 236<br><br>Page 7; 296 - 303

Results/findings

**Synthesis and interpretation** –<br>See section 3.1 "Drivers of successful vaccination programs in exemplar countries" in addition to Figure 2 and Table 2, and Table 3.	Page 8; 326 - 339<br><br>Page 8 341<br><br>Page 8- 9; 344 – 348<br><br>Page 12; 481 - 485

For peer review only - http://bmjopen.bmj.com/site/about/guidelines.xhtml

**Links to empirical data** – See quotes as included in-text in addition to quotes by country and domain in Appendix 2. Citations included where reports or other documents were utilized as part of the analysis.	Embedded<br><br>Appendix 2

Discussion

**Integration with prior work, implications, transferability, and contribution(s) to the field** – See section 4, Discussion, starting on page 13.	Page 13 – 14; 489 - 556
**Limitations** – See section 5, Limitations, on page 14.	Page 14; 561 - 568

Other

**Conflicts of interest** – "The authors declare that they have no known competing financial interests or personal relationships that could have appeared to influence the research reported in this paper."	Page 15; 606 - 607
**Funding** – "This work was supported by the Bill & Melinda Gates Foundation, Seattle, WA (OPP1195041) with a planning grant from Gates Ventures, LLC, Kirkland, WA.:	Page 15; 602 - 603

*The authors created the SRQR by searching the literature to identify guidelines, reporting standards, and critical appraisal criteria for qualitative research; reviewing the reference lists of retrieved sources; and contacting experts to gain feedback. The SRQR aims to improve the transparency of all aspects of qualitative research by providing clear standards for reporting qualitative research.

**The rationale should briefly discuss the justification for choosing that theory, approach, method, or technique rather than other options available, the assumptions and limitations implicit in those choices, and how those choices influence study conclusions and transferability. As appropriate, the rationale for several items might be discussed together.

Reference:

O'Brien BC, Harris IB, Beckman TJ, Reed DA, Cook DA. **Standards for reporting qualitative research: a synthesis of recommendations.** *Academic Medicine*, Vol. 89, No. 9 / Sept 2014
DOI: 10.1097/ACM.0000000000000388

For peer review only

BMJ Open

Critical success factors for high routine immunization performance: a qualitative analysis of interviews and focus groups from Nepal, Senegal, and Zambia

Journal:	*BMJ Open*
Manuscript ID	bmjopen-2022-070541.R2
Article Type:	Original research
Date Submitted by the Author:	10-Aug-2023

Complete List of Authors:	Sakas, Zoe; Rollins School of Public Health<br>Hester, Kyra; Emory University, Rollins School of Public Health<br>Ellis, Anna; Emory University, Rollins School of Public Health 1518 Clifton Rd Atlanta, GA, USA 30322<br>Ogutu, Emily; Emory University, Gangarosa Department of Environmental Health<br>Rodriguez, Katie; Emory University<br>Bednarczyk, Robert ; Emory University, Hubert Department of Global Health<br>Dixit, Sameer; Center for Molecular Dynamics Nepal<br>Kilembe, William; Emory University, Rwanda Zambia HIV Research Group<br>Sarr, Moussa; Institut de Recherche en Santé de Surveillance Epidémiologique et de Formations ,<br>Freeman, Matthew; Emory University, Gangarosa Department of Environmental Health

**Primary Subject Heading**:	Global health
Secondary Subject Heading:	Global health
Keywords:	Paediatric infectious disease & immunisation < PAEDIATRICS, PUBLIC HEALTH, QUALITATIVE RESEARCH

SCHOLARONE™
Manuscripts

I, the Submitting Author has the right to grant and does grant on behalf of all authors of the Work (as defined in the below author licence), an exclusive licence and/or a non-exclusive licence for contributions from authors who are: i) UK Crown employees; ii) where BMJ has agreed a CC-BY licence shall apply, and/or iii) in accordance with the terms applicable for US Federal Government officers or employees acting as part of their official duties; on a worldwide, perpetual, irrevocable, royalty-free basis to BMJ Publishing Group Ltd ("BMJ") its licensees and where the relevant Journal is co-owned by BMJ to the co-owners of the Journal, to publish the Work in this journal and any other BMJ products and to exploit all rights, as set out in our licence.

The Submitting Author accepts and understands that any supply made under these terms is made by BMJ to the Submitting Author unless you are acting as an employee on behalf of your employer or a postgraduate student of an affiliated institution which is paying any applicable article publishing charge ("APC") for Open Access articles. Where the Submitting Author wishes to make the Work available on an Open Access basis (and intends to pay the relevant APC), the terms of reuse of such Open Access shall be governed by a Creative Commons licence – details of these licences and which Creative Commons licence will apply to this Work are set out in our licence referred to above.

Other than as permitted in any relevant BMJ Author's Self Archiving Policies, I confirm this Work has not been accepted for publication elsewhere, is not being considered for publication elsewhere and does not duplicate material already published. I confirm all authors consent to publication of this Work and authorise the granting of this licence.

For peer review only

Critical success factors for high routine immunization performance: a qualitative analysis of interviews and focus groups from Nepal, Senegal, and Zambia

Zoe Sakas MPH[a], Kyra A. Hester MPH[a], Anna S. Ellis MDP[a], Emily Awino Ogutu MPH[a], Katie Rodriguez MPH[a], Robert A. Bednarczyk PhD[a], Sameer Dixit PhD[b], William Kilembe MD[c], Moussa Sarr MD[d], Matthew C. Freeman PhD[a*]

Affiliations
a Rollins School of Public Health, Emory University, Georgia, USA
b Center for Molecular Dynamics Nepal, Kathmandu, Nepal
c Center for Family Health Research in Zambia, Lusaka, Zambia
d Institut de Recherche en Santé de Surveillance Epidemiologique et de Formation, Dakar, Senegal

*Correspondence to:
Matthew C. Freeman, PhD
matthew.freeman@emory.edu

Abstract

Objectives: Vaccination averts an estimated 2-3 million deaths annually. Although vaccine coverage improvements across Africa and South Asia have remained below global targets, several countries have outperformed their peers with significant increases in coverage. The objective of this study was to examine these countries' vaccination programs and to identify and describe critical success factors that may have supported these improvements.

Design: Multiple case study design using qualitative research methods.

Setting: Three countries with high routine immunization rates: Nepal, Senegal, and Zambia.

Participants: We conducted 66 key informant interviews and 22 focus group discussions with a total of 678 participants. Participants were recruited from all levels, including government officials, health facility staff, frontline workers, community health workers, and parents. Participants were recruited from both urban and rural districts in Nepal, Senegal, and Zambia.

Results: Our data revealed that the critical success factors for vaccination programs relied on the cultural, historical, and statutory context in which the interventions were delivered. In Nepal, Senegal, and Zambia, high immunization coverage was driven by 1) strong governance structures and healthy policy environments; 2) adjacent successes in health systems strengthening; 3) government-led community engagement initiatives; and 4) adaptation considering contextual factors at all levels of the health system.

Conclusions: Throughout this project, our analysis returned to the importance of defining and understanding the context, governance, financing, and health systems within a country, rather than focusing on any one intervention. This paper augments findings from existing literature by highlighting how contextual factors impact implementation decisions that have led to improvements in childhood vaccine delivery. Findings from this research may help identify transferable lessons and support actionable recommendations to improve national immunization coverage in other settings.

For peer review only - http://bmjopen.bmj.com/site/about/guidelines.xhtml

Strengths and limitations of this study

- This study was conducted by a multi-disciplinary team with authors and advisors from a variety of sectors, including immunization, epidemiology, political science, implementation science, and behavioral science.
- Our team ensured the involvement of stakeholders, researchers, and policy makers in the global immunization sector during the design, implementation, and dissemination of this project.
- Through extensive qualitative data collection, we were able to gain insight from stakeholders at all levels of the vaccine delivery systems in our target countries – from mothers to community health workers, to national-level leadership.
- It was difficult to determine causation because we focused on countries that were successful in vaccine delivery but were unable to carry out a similar analysis in countries with lower vaccination coverage for comparison.
- Using qualitative methods to understand historical events was challenging; interviewees often spoke about current experiences rather than discussing historical factors.

For peer review only - http://bmjopen.bmj.com/site/about/guidelines.xhtml
For peer review only

Introduction

Vaccination is recognized as one of the most influential public health interventions, averting an estimated 2-3 million deaths annually [1-4]. From 2012 to 2020, the Global Vaccine Action Plan (GVAP) aimed to reach 90% country-level coverage and 80% district-level coverage of the third dose of diphtheria, tetanus, pertussis vaccine (DTP3) among 1-year-old children [5], a globally recognized proxy for vaccination system performance [6]. However, by 2018, only 95 of the 193 World Health Organization (WHO) Member States achieved country-level GVAP targets, and less than one third met district-level targets [7-9]. In the late 2000s, DTP3 coverage in Africa and South Asia was particularly low, at 74% (2029) and 78% (2016), respectively [8]. However, several countries outperformed their peers with significant increases in routine immunization coverage from 2000 to 2020 [10]. Examining the vaccination programs of these exemplary countries provides an opportunity to identify and describe critical success factors that may have supported these improvements.

The essential components of an effective vaccine delivery system are well-established and include strong governance and leadership, healthcare financing, human resources, a robust supply chain, community engagement, and information systems for evidence-based decision-making [11-13]. Research on behaviors related to routine immunization focuses on recognized determinants of coverage, including intent to vaccinate, community access, and health facility readiness [14]. Although existing literature describes vaccine delivery systems, there is a gap in knowledge related to how programs and policies are implemented, how strategies are operationalized, and how context influences programming. By closely examining the vaccine delivery systems of three countries with high vaccination rates, we can draw conclusions about *how* and *why* vaccine programs work in specific contexts, and how they may be tailored for success elsewhere. This paper augments findings from existing literature by highlighting how structural and contextual factors – including governance, collaboration, the environment, and cultural norms - impact implementation decisions that have led to improvements in childhood vaccine delivery.

The purpose of this project was to identify critical success factors that contributed to catalytic growth in childhood routine immunization coverage in three countries, and to describe how countries with high vaccination rates took similar paths to success [15-19]. We retrospectively analyzed improvements in vaccine delivery from 2000 to 2020. The COVID-19 pandemic sent shocks through vaccination programs worldwide, negatively impacting vaccination coverage. However, the global impact of the pandemic was not examined as part of this study due to our retrospective approach. Findings from this research may identify transferable lessons and support actionable recommendations to improve national immunization coverage in other settings [20].

Methods

This multiple case study analysis was conducted using data from the Exemplars in Vaccine Delivery project within the Exemplars in Global Health program, funded by the Gates Foundation and executed through Gates Ventures [10, 20, 21]. The Exemplars in Vaccine Delivery project involved qualitative examination of the national vaccine delivery systems of three low- and middle-income countries – namely Nepal, Senegal, and Zambia – with high childhood routine immunization coverage, compared to their peers.

This paper compares the findings from three individual country-level case studies. We identify and describe factors that impacted successful vaccine delivery in Nepal, Senegal, and Zambia while addressing differences based on contextual influence. The individual case study papers provide additional details regarding country-specific implementation [15, 16, 17].

For peer review only - http://bmjopen.bmj.com/site/about/guidelines.xhtml

We used qualitative analysis to investigate factors that contributed to high vaccination coverage through key informant interviews (KIIs) and focus group discussions (FGDs) at the national, regional, district, health facility, and community levels. We triangulated these findings with quantitative analyses using publicly available data, which are published elsewhere and referenced throughout this paper [10, 21].

Prior to data collection, we developed a conceptual framework (Figure 1) to organize factors that impact childhood vaccine coverage globally. This framework was based on the work of Phillips et al. and LaFond et al. alongside a broader review of the vaccine literature [14, 22]. Details regarding development are available in our protocol [10].

Study sites

Countries were selected based on DTP1 and DTP3 coverage estimates because DTP1 is commonly used as a proxy of access, and DTP3 as a proxy of continued utilization of immunization services (Appendix 1) [10, 23, 24]. Nepal, Senegal, and Zambia were selected as exemplary countries due to relatively high DTP1 and DTP3 coverage during the timeframe assessed in this study. Three regions within each country were identified in consultation with national stakeholders and available data (Table 1). Partner organizations and Ministry of Health (MoH)[1] officials facilitated site selection and data collection activities. Additional methods for country, region, and district selection are provided in our protocol and case studies [10, 15-17].

Qualitative data collection and analysis

Qualitative data were collected between October 2019 and April 2021 in Nepal, Senegal, and Zambia (Table 1). KIIs were held with government officials to understand their current and historical perspectives on the national vaccination program based on their official positions. Given inherent power dynamics, we decided that FGDs would have been inappropriate. At the community level, we conducted FGDs because we hoped that group conversations would allow for interactive discourse on the key themes. Interview guides were informed by the Consolidated Framework for Implementation Research (CFIR) [25] and the Context and Implementation of Complex Interventions (CICI) framework [26]. KII and FGD guides were translated into local languages by research assistants. All interview guides were piloted before use and adjusted iteratively throughout data collection. For each country, an initial list of KIIs was developed with local research partners and MoH officials with snowball sampling used to identify additional key informants. Our sampling approach aimed to include a diverse sample of participants regarding geographic location and demographic qualities. Caregivers and volunteer community health workers were recruited for FGDs from health facility catchment areas with the assistance of local health staff. The duration of KIIs and FGDs averaged one and a half hours. KIIs and FGDs were audio-recorded with the permission of participants. Research files, recordings, and transcriptions were de-identified and password protected. Data analysis processes were generally consistent in all three countries examined.

We applied thematic analysis of the transcripts to identify critical success factors. Analysis was informed by the domains outlined in Figure 1. Data analysis was initially conducted individually for each case study, with separate members of our research team leading analysis for each country. However, to ensure consistency and coherence, all data analysts were involved in the review, coding, and summarization of data from all three countries. We met frequently to discuss overlap and to compare empirical findings. Transcripts were coded and analyzed using MaxQDA2020 software (Berlin, Germany). Coding was

[1] *Note that each country has a slightly different name for their Ministry of Health (e.g., Ministry of Health and Population in Nepal; Ministry of Health and Social Action in Senegal), but all will be referred to as "MoH" throughout for simplicity.*

For peer review only - http://bmjopen.bmj.com/site/about/guidelines.xhtml

completed by researchers at Emory, some of whom also developed and revised the codebook. Coding meetings were held frequently to discuss code use and definitions. Researchers from in-country teams attended additional meetings to discuss data and to ground-truth findings. We considered setting and participant roles while identifying key points and further contextualized data using historical documents and a literature review. Qualitative data collection and analysis tools, including topic guides and codebooks, can be found in Appendix 2 and on our Open Sciences Framework (OSF) webpage [27].

Following analysis of individual case studies, we explored and discussed the similarities and differences between our findings. Using a multiple-case study approach, we developed conceptual ideas that pulled from all three countries to highlight the key components required for successful vaccine delivery (as per analysis of factors from three exemplary countries). Our discussions were rooted in the empirical data and we supported all conclusions with informative quotations and data summaries. When there were discrepancies, we revisited the raw data from individual case studies.

Table 1. Summary of countries, regions, districts selected for research, and data collection activities

**Country**	**Nepal**		**Senegal**		**Zambia**	
In Country Research Partner	Center for Molecular Dynamics, Nepal		Institut de Recherche en Santé de Surveillance, de Surveillance Epidémiologique et de Formation (IRESSEF)		Center for Family Health Research in Zambia	
Data collection period (MM/YYYY)	8/2019 – 12/2019		12/2020 - 4/2021		10/2019 – 02/2020	
**Regions and Districts**						
Region 1	Madhes*	Dhanusha, Bara, Mahottari	Ziguinchor	Ziguinchor, Oussouye, Diouloulou	Lusaka	Lusaka, Rufunsa, Chongwe
Region 2	Bagmati	Makwanpur, Dolakha, Kathmandu	Dakar	Rufisque, Mbao, Keur Massar	Central	Chibombo, Chitambo, Serenje
Region 3	Gandaki Pradesh	Kaski, Myagdi, Nawalparasi	Tambacounda	Tambacounda, Koumpentoum, Goudiry	Luapula	Chipili, Nchelenge, Samfya
**Key Informant Interviews**<br>*Key: Number of KIIs (Participants)*	**79 (79)**		**62 (63)**		**66 (85)**	
National level government staff	11 (11)		5 (5)		11 (12)	
Partner organization staff	8 (8)		4 (4)		11 (15)	
Regional health staff	5 (5)		7 (7)		6 (8)	
District health staff	15 (15)		38 (38)		10 (19)	
Health facility staff	23 (23)		6 (6)		7 (10)	
Community leaders	15 (15)		2 (2)		10 (10)	
Community health workers **	2 (2)		-		11 (11)	
**Focus Group Discussions**<br>*Key: Number of FGDs (Participants)*	**30 (191)**		**19 (128)**		**22 (132)**	
Community health workers **	9 (60)		10 (65)		10 (60)	
Mothers	9 (60)		9 (63)		8 (48)	
Fathers	6 (36)		-		1 (6)	

For peer review only - http://bmjopen.bmj.com/site/about/guidelines.xhtml

Grandparents	6 (35)	-	3 (18)
**Total (per country)**	**109 (270)**	**81 (191)**	**88 (217)**
**Total (across countries)**	**-**	**-**	**278 (678)**

** Madhes was referred to as Province 2 prior to 2022 (including during data collection)*
*** Includes volunteer community health workers, female community health volunteers (FCHV) – Nepal; vaccinators – Nepal; bajenu gox – Senegal; and neighborhood health committee members – Zambia.*

Supplemental quantitative data analysis

Quantitative data were collected through secondary datasets, which included information from the MOH in Zambia, Nepal, and Senegal among other partners. Data were used to estimate routine immunization coverage from 2000 to 2019 and to uncover trends related to significant improvements. Additional analyses were conducted to identify indicators that may be associated with immunization coverage success among low- and lower-middle-income countries using regression frameworks to statistically test financial, development, demographic, and other country-level indicators. These quantitative analyses were published elsewhere [28, 29] . Findings from the quantitative analyses provided additional context for the qualitative findings which informed the results presented here.

Patient and public involvement

Throughout the Vaccine Exemplars project – which included individual case studies and the pooled analysis presented in this paper – the study team ensured the involvement of stakeholders, researchers, and policy makers in the global immunization sector during the design, implementation, and dissemination of this project. We utilized our Technical Advisory Group (TAG) to develop an initial set of questions for scoping visits to our exemplar countries, wherein study members met with in-country partners and experts to further refine the direction and nature of the questions. Scoping visit findings were used to create the final data collection tools [10]. As this is a hypothesis generating study, and no intervention was provided, data collection was targeted to conform to general standards of burden. We adhered to standard best-practices for qualitative research, iteratively implementing feedback from experts and the TAG. While there was community involvement (FGDs), the primary focus of data collection was centered on historical stakeholders who played key roles in the immunization program [10]. Upon completion of each country case study, findings were disseminated to in-country experts and country governments, along with the TAG, allowing for the study team to ground-truth results. The research team at Emory University co-hosted regular meetings with the in-country research partners, experts in a variety of relevant fields, government officials, and other advisors to ask for feedback and contextualization. Researchers would present on methods, findings, and implications following an engaging discussion with advisors that would lead to improvements in our data analysis processes or presentation of findings. Revisions would be distributed through follow-up email conversations and subsequent phone calls with select advisors. Reports, and manuscripts, of findings were sent to those involved. Feedback from stakeholders was incorporated into this multiple case study analysis.

More information about how this study addresses local research and policy priorities is included in our Reflexivity Statement (Appendix 3).

Sex and gender considerations

For peer review only - http://bmjopen.bmj.com/site/about/guidelines.xhtml

Although we did not provide an intervention, the research team took gender differences into consideration. For our FGDs, we recruited both mothers and fathers. FGDs were separated by gender so that participants would feel comfortable sharing their thoughts.

Due to the historical nature of this study, our KII sample of key stakeholders and leaders leaned male due to prior gender biases and the tendency to place men in positions of importance or power. However, present-day positions were often filled by women, and we sampled women for KIIs as appropriate.

Results

Drivers of successful vaccination programs in exemplar countries

Following analysis of data from Nepal, Zambia, and Senegal, we revisited the conceptual framework developed during study conception (Figure 1). Although existing literature elaborates on determinants of vaccination coverage (e.g., intent to vaccinate, facility readiness, and community access), there is a gap in knowledge related to *how* policies and programs are operationalized to address these determinants – which relies heavily on the 1) existing governance and health systems, 2) engagement of community members (or end-users), and 3) ability to adapt to context to improve implementation.

We revised the initial conceptual framework (Figure 1) to more accurately reflect findings from empirical data and to highlight emergent success factors (Figure 2). Definitions for the key domains presented in the revised framework can be found in Table 2.

Table 2. Definitions for drivers of vaccine coverage

Domain	Definition
**Governance and financing** [a]	The processes that guide policies and operations within the health system, including collaboration (effective working relationships, including alignment of priorities), communication (open and frequent channels for sharing knowledge and feedback), and commitment (dedication to activities and responsibilities).
**Health systems strengthening** [a]	Strategies that are designed to sustainably improve health system performance (across all sectors) through an integrated and horizontal approach, with a focus on equity.
**Community engagement** [a]	Responsibility of the community to implement vaccination program activities through local leadership and volunteers, fostered by government initiatives and cultural values.
**Adaptive capacity** [a]	Capability to sufficiently consider contextual factors, supported by timely evaluation of quality data, to tailor vaccine programming based on local needs and priorities – and improve implementation quality.
**Intent to vaccinate** [b]	Demand for childhood vaccines on the part of the caregiver or guardian that would result in vaccination in the absence of other barriers.

For peer review only - http://bmjopen.bmj.com/site/about/guidelines.xhtml

**Facility readiness** [b]	Supply (by the health system) of health and vaccination services to adequately meet demand. Incorporates supplies (vials, syringes etc.), human resources, and the consistency of their availability.
**Community access** [b]	The ability (or inability) to successfully carry out the transaction of vaccine utilization – for example, a community member's ability to access vaccines should they decide to get vaccinated - i.e., barriers and facilitators between Intent and Readiness.

a. *Developed from our research, both via data analysis and desk review of existing literature*
b. *See Figure 1 (and reference Phillips et al. paper)*

Operationalization of successful vaccine delivery programs

In Nepal, Senegal, and Zambia, high immunization coverage was driven by 1) strong governance structures and healthy policy environments; 2) adjacent successes in health systems strengthening; 3) government-led community engagement initiatives; and 4) adaptation of programming based on contextual factors at all levels of the health system.

In Nepal, public codification of health (and therefore, vaccines) as a human right ensured the stability of vaccination programming, increased morale among health workers, and helped to generate demand for vaccines. Female Community Health Volunteers (FCHVs) and other community health workers lead a powerful community-based approach to demand generation, health education, and vaccine delivery, which was supported by cultural values including collective responsibility and community ownership of vaccine coverage.[16] In Senegal, strong political will supported the prioritization of vaccination programming, including urgent allocation of resources and cold chain improvements. Long-term partnerships between government agencies and external partners allowed for innovation, capacity building, and efficiency within the health system. Lastly, improved surveillance efforts led to timely and evidence-based decision making.[17] In Zambia, effective collaboration was essential to successful vaccine delivery. The Inter-Agency Coordinating Committee led long-term collaboration efforts at the national level and supported evidence-based programming through multiple technical working groups. At the local level, Neighborhood Health Committees spearheaded community-driven strategies via community action planning and strengthened the link between communities and health facilities.[15]

Below, we summarize similarities apparent in all three high-performing countries. More information on how policies and programs were implemented and adapted to local context is available elsewhere [15-17]. Informative quotes from each country and domain can be found in Appendix 4.

Strong governance structures and a healthy policy environment
Effective collaboration, open communication, and commitment to health and vaccines supported policy development and implementation of the vaccination programs in Nepal, Senegal, and Zambia.

Collaboration and communication enabled alignment of priorities, division of responsibilities, and coordination between levels. In all three countries, collaboration was apparent between external partners and the government, and between different levels of the health system. National fora were established for frequent and multi-disciplinary communication, including Inter-Agency Coordinating Committees (ICC) [30]. Regional, district, and community level reflection meetings were held to share and review data and gather feedback for strategic planning. Formal channels for supportive supervision and feedback, both top-down and bottom-up, supported service delivery improvements and reporting.

For peer review only

In all three countries, vaccination was a “priority” or “flagship” program recognized by the MOH. Prioritization of vaccination within the health sector supported sufficient resource allocation. In Nepal and Senegal, dedication to the health sector more broadly was codified through defining health as a right in their national constitutions. Commitment to health and vaccines underpinned policy development and enabled consistent service delivery. Community members could expect reliable and equitable access to vaccines due to the prioritization of activities by health personnel and the dedication of volunteer community health workers who supported outreach activities and spearheaded demand generation and educational campaigns.

> *“A provision was made in the constitution, and [vaccines] became a fundamental right, so the government has a responsibility to provide. And management of currently running programs also [focuses on] children who have missed vaccines.” (Nepal Health Research Council, Nepal)*

Adjacent successes in health systems strengthening

Improvements in the broader health systems of Nepal, Senegal, and Zambia – including health post expansion, training of volunteer community health workers, general capacity building, and prioritization of equitable health services – supported high immunization coverage.

Health post expansion and capacity building at subnational and community levels were mentioned by most key informants in all three countries as essential to improvements in vaccination coverage. Building health facilities in rural areas especially allowed parents to easily vaccinate their children, without having to travel far distances or rely on outreach. Improvements in vaccine service delivery also relied on improvements in general infrastructure including roads, electricity, and connectivity required for transportation, health facility operations, and data reporting. In all three countries, general health systems strengthening was centered around equity as a priority for decision making. Health post expansion and community health worker programs were strengthened to prioritize service delivery in hard-to-reach communities, minority populations, and remote areas.

> *“Previously, there was no public health department at a grassroots level, it was just at the National level... So, now the Public Health Specialist is on the ground to try to see that this primary health care activity is promoted. And I think with that supervision, with the introduction of the public health nurses, Health promotion departments, I think these interventions couldn’t be achieved higher in terms of immunization activities.” (District Health Director, Zambia)*

Ongoing efforts to address equity concerns include: 1) Overcoming geographical barriers through health post expansion and outreach services supported by national-level policy and external partner funding; and 2) Addressing social, economic, and cultural factors through tailored messaging and local ownership of vaccination programming.

Integrated decision making across health and social sectors, through a horizonal rather than vertical approach, facilitated effective allocation of resources (e.g., monetary, human, technical, educational) could be efficiently allocated across programs. For example, national fora and committees in all three countries included representatives from the immunization sector, maternal and child health, education and development, and financial offices. During meetings, sectors would align long-term priorities and discuss how to reach overall goals in terms of health and social outcomes.

Government-led community engagement initiatives

Community ownership of vaccination activities and corresponding health outcomes was apparent in Nepal, Senegal, and Zambia, and even more so in high-performing districts within these countries. Responsibility of the community to implement vaccination programming through local leadership and

For peer review only - http://bmjopen.bmj.com/site/about/guidelines.xhtml

volunteers was fostered by government initiatives and cultural values. National policies focused on involvement of traditional leaders and all national-level stakeholders reported on the importance of community actors. In some communities, female volunteers would work beyond their mandates to ensure all children were vaccinated – a decision motivated by dedication to their communities at large, empathy for the children in their neighborhoods, and a vague understanding of herd immunity.

Key informants from the national to community level reported that demand for vaccines was critical for improving routine immunization coverage. In addition to the work of volunteer community health workers, demand generation and public awareness were accomplished through national policies, media engagement, involvement of community actors, and school outreach activities.

> *"Community health workers play a very important role – they are in direct contact with the population. They are the ones who live in the community, and they identify more with the community. So, there is a relationship of trust between them and the population." (National level government stakeholder, Senegal)*

Further analysis of community health worker programs and demand generation activities in Nepal, Senegal, and Zambia will be detailed in our upcoming publications [31].

Adaptation at all levels of the health system

In Nepal, Senegal, and Zambia, decision-makers and implementers at the national, subnational, and community levels demonstrated their ability to consider contextual factors, evaluate or reflect on quality data, and tailor vaccine programming based on local needs and priorities.

Quality data were available for evidence-based decision making in all three countries. Data software was implemented at the district level in all countries - or in the case of Senegal, at the health post level - allowing stakeholders at all levels to utilize accurate and up-to-date coverage estimates. Lack of quality data in some areas may have hindered this process. However, when data were not available, community actors would adapt programming based on head counts conducted by volunteers and an anecdotal understanding of the contextual and cultural needs in their communities.

> *"We have been advising facilities that they need to own the data; they have to make sure that they utilize it by analyzing… If they are not meeting their targets, they need to reflect on the data and see where they are going wrong… For those [health facilities] that are performing well, we find out what they are doing, and we try to replicate to the other facilities that are not doing as well." (District Health Officer, Zambia)*

Adapting strategies to respond to context-specific needs was essential for reaching children. In all three countries, flexible vaccine delivery days and outreach services supported unique scheduling needs (e.g., in Senegal, vaccinating children in the late afternoon during farming season). Outreach strategies were also tailored to accommodate contextual factors and address barriers to access. Understanding cultural and social values enabled community actors to tailor demand generation activities so that community members would be more apt to adopt positive health behaviors. Some examples of this may be using a local language, involving religious or community leaders in engagement and sensitizing, or addressing cultural or social barriers directly through personal communication.

For peer review only - http://bmjopen.bmj.com/site/about/guidelines.xhtml

Interventions and programs across high-performing countries

Key informants discussed a myriad of interventions and programs that they believed led to the improved and sustained vaccination coverage in their countries (Table 3). Interventions are organized by implementation level; if an intervention was implemented at multiple levels of the health system, it may be repeated in the table. Although the specific components of vaccine programming were not identical across countries, many of the general themes overlapped (e.g., the scope of community-level health committees varied between countries, but the purpose/objective of these groups was similar).

Table 3. Key interventions and programs implemented to improve routine immunization coverage in Nepal, Senegal, and Zambia from 2000 to 2019, based on KII and FGD data

	**Key for reading the table below:**
Red	Not mentioned by key informants or community stakeholders who participated in this study
Green	Implemented; participants noted that there was room for improvement (e.g., gaps or challenges)
Blue	Implemented; participants described successful implementation (e.g., exceeds recommended guidelines)

**Category**	**Policy or intervention**	**Zambia**	**Nepal**	**Senegal**
**National level programming**<br>*Includes external partners and government*	Introduction of new vaccines	Blue	Blue	Blue
	Vaccination as "priority" program	Green	Blue	Green
	Strategic reallocation of targeted funds	Blue	Blue	Red
	Health post expansion	Green	Green	Green
	Constitution codifying health as a right	Red	Blue	Green
	Forums for decision-making (e.g., ICC)	Blue	Green	Green
	Media engagement	Blue	Blue	Blue
**Sub-national programming**<br>*Regional and district level*	RED/REC implementation	Blue	Green	Blue
	Training community health workers	Green	Green	Green
	Meetings for evidence-based decisions	Green	Green	Blue
	Data software, health post level	Red	Red	Green
	Data software, district level	Green	Green	Green
	Cold chain expansion	Blue	Green	Green
**Community programming**<br>*Health post and village/community level*	Community health worker program	Blue	Blue	Blue
	Community-level health committees	Blue	Green	Blue
	Outreach services	Blue	Blue	Blue
	Microplanning in health facilities	Blue	Green	Green
	Engagement of community leaders	Blue	Blue	Blue
	Support groups for caregivers	Green	Blue	Green
	School outreach for health education	Green	Green	Green
	Promotion through media (e.g., radio)	Blue	Blue	Blue

Discussion

Notions of strengthening national governance structures have historically been recognized through country-level application of global guidelines to improve vaccine delivery and health systems; however, existing research lacks concrete descriptions of what this entails. This paper augments findings from

For peer review only - http://bmjopen.bmj.com/site/about/guidelines.xhtml

existing literature by highlighting how structural and contextual factors – including governance, collaboration, the environment, and cultural norms - impact implementation decisions that have led to improvements in childhood vaccine delivery. We developed evidence-derived operational definitions of structures and processes that we identified as critical to the success of vaccine delivery systems in countries with high routine immunization coverage. We described *how* these processes were utilized in high-performing countries to drive catalytic improvements in coverage. Previous research has not explored the details or importance of governance, financing, health systems, or other contextual factors; our original framework based on preexisting literature (Figure 1) was missing complexity in these components, which is indicative of the limited knowledge in this area. In order to understand how global guidelines should be adapted to reflect local priorities and challenges, it was important for us to describe how national governance structures operate with the consideration of contextual factors. Ignoring a structured approach to understanding these factors could lead to repeated instances of sub-optimal program performance.

Our data points to the need for programmers and policy makers to better understand underlying governance structures. The structures and processes we examined in Nepal, Senegal, and Zambia supported adaptive decision-making based on context-specific needs and priorities. Adaptation at all levels enabled tailored and targeted programming based on evaluation of high-quality data. While health system improvements led by national governments were essential, our data also highlighted the importance of a deliberate and consistent focus on community engagement, which aligns with existing literature [1,27,28]. Community involvement in vaccine programming facilitated the government's understanding of cultural and social barriers and enablers which contributed to effective adaptation of interventions to increase coverage. Community ownership was fostered through national policies and resources for capacity building, engagement of traditional leaders, and the strength of the community health worker programs.

We found that many improvements in immunization coverage emerged from adjacent successes in health systems strengthening (HSS). While an unsurprising finding that health system strengthening would be an essential ingredient in support of vaccine system strengthening, it points to the need for a more integrated, rather than siloed, approach to child health. HSS has become a priority in recent decades as disruptions due to natural disasters, disease outbreaks, or civil unrest have exposed weaknesses in health systems [32, 33]. Although we focused on examining success factors for vaccine delivery, our data also suggests that there are still gaps and challenges in exemplar countries, including: 1) equitable access (e.g., lack of health posts in rural and remote areas); 2) long-term financing (e.g., most funding comes from external partners); 3) poor infrastructure (e.g., roads, electricity); and 4) a heavy reliance on volunteer community health workers which experienced vacancies and decreased morale in some cases.

In Nepal, Senegal, and Zambia, overall commitment to health and vaccines strengthened governance processes and supported vaccination efforts through prioritization of activities and resource allocation. Following the completion of GVAP in 2020, stakeholders reported that lack of consideration of existing structures and contextual factors limited the plan's success [34]. In addition, the monitoring and evaluation processes proposed through GVAP were often seen as unrealistic [9, 34, 35]. Suggested improvements included engaging with countries to accurately and sufficiently consider local context while setting global goals and targets and promoting country ownership of strategic planning [34, 35]. Our research revealed that national government ownership of strategic planning was critical to these countries' success, which aligns with the critical reflection of GVAP and other existing literature.

Overall, the strategies implemented by exemplar countries align with the WHO Comprehensive Framework of Strategies and Practices for Routine Immunization, which focus on managing the immunization program (e.g., political commitment, partnerships), mobilizing communities, generating demand, and monitoring progress [1]. Throughout the study, our analysis returned to the importance of

For peer review only - http://bmjopen.bmj.com/site/about/guidelines.xhtml

defining and understanding the context, governance, financing, and health systems within a country, rather than focusing on any one intervention. This highlights the importance of further research into these structural factors. We examined how these strategies were operationalized to improve coverage in Nepal, Senegal, and Zambia, and how they can be applied in other settings. Understanding how governance processes function in high-performing countries may allow others to build stronger systems, adapt guidelines, and reflect on progress made in the field. Our research introduces areas of inquiry for further investigation, as the critical success factors that primarily emerged in all three exemplar countries are not the current focus within existing literature around determinants for vaccine coverage. as the critical success factors that primarily emerged in all three exemplar countries are not the current focus within existing literature around determinants for vaccine coverage.

This study has several limitations. First, it was difficult to determine causation because we focused on countries that were successful in vaccine delivery but were unable to carry out a similar analysis in a country with lower vaccination coverage to compare. Second, the research tools focused on the factors that drove catalytic change and did not focus on interventions or policies that were unsuccessful. Third, using qualitative methods to understand historical events was challenging; interviewees often spoke about current experiences rather than discussing historical factors. However, we probed respondents to reflect on longitudinal changes in the immunization program. Additional country-specific limitations can be found elsewhere [15-17].

Conclusion

Through focusing on countries with high routine immunization coverage, we examined how vaccine delivery systems may leverage components of existing governance structures and health systems to accelerate and sustain coverage. Operational definitions for governance, health systems strengthening, community engagement, and adaptive capacity, along with descriptions of how these processes were implemented in high-performing countries, may help other countries implement similar improvements. Our findings highlight the need for program managers and policy makers to understand and consider the strengths and limitations of existing structures while adapting to the emergent needs and priorities relevant to the context. Our study looked retroactively on how and why countries succeeded in achieving high early childhood immunization coverage. The COVID-19 pandemic has highlighted the fragility in our health systems, and specifically how robust vaccine systems can rise to the challenge of emergent vaccination needs. While our findings are not immediately applicable to the current COVID vaccination needs, our underlying approach to understand context remain relevant to consider as countries strive to vaccinate their populations in the ongoing pandemic [36].

For peer review only - http://bmjopen.bmj.com/site/about/guidelines.xhtml

Acknowledgements

We gratefully acknowledge the participants who gave their time and insights to help us better understand the vaccine delivery systems of Zambia, Nepal, and Senegal, along with facilitators from their respective Ministries of Health.

Contributions were also made by Sarah Chesemore, Anna Rapp, Tove Ryan, and Ethan Wong from the Bill & Melinda Gates Foundation; Kate Buellesbach, Nancy Fullman, Nathaniel Gerthe, Gloria Ikilezi, Caitlyn Mason, David Phillips, and Oliver Rothschild, Jordan-Tate Thomas, and Angela Wang from Gates Ventures; and the Vaccine Exemplars Research Advisory Group, including Agnes Binagwaho, Laura Craw, Carolina Danovaro, Anuradha Gupta, Heidi Larson, Penelope Masumbu, Kate O'Brien, Helen Rees, Lora Shimp, and Aaron Wallace.

More information about how this study addresses local research and policy priorities is included in our Reflexivity Statement.

Funding

This work was supported by the Bill & Melinda Gates Foundation, Seattle, WA (OPP1195041) with a planning grant from Gates Ventures, LLC, Kirkland, WA.

Competing interests

The authors declare that they have no known competing financial interests or personal relationships that could have appeared to influence the research reported in this paper.

Data availability statement

Data are not publicly available as all data are confidential. De-identified data may be available upon request.

Contributors

KAH, ASE, SD, WK, MS, RAB, MCF: project conceptualization and methodology; KAH, ZS, ASE, KR, SD, WK, MS, RAB, MCF: investigation and data curation; KAH, ZS, ASE, KR, EAO, RAB, MCF: formal analysis; ZS, KAH: writing - original; KAH, ZS, ASE, EAO, KR, SD, WK, MS, RAB, MCF: writing – review and editing; all authors provided approval of the final version.

Ethics approval statement

The multi-country Vaccine Exemplars study – which included individual case studies and a pooled analysis - was considered exempt by the Institutional Review Board committee of Emory University, Atlanta, Georgia, USA (IRB00111474). Data collection for individual countries was approved by the Nepal Health Research Council (NHRC; Reg. no. 347/2019) in Kathmandu, Nepal; the National Ethical Committee for Health Research (CERNS; Comité National d'Ethique pour la Recherche en Santé) in Dakar, Senegal (00000174); the University of Zambia Biomedical Research Ethics Committee (Federal Assurance No. FWA00000338, REF. No. 166-2019); and the National Health Research Authority in Zambia. All ethics committees abide by the principles of the Declaration of Helsinki. All participants provided written consent.

For peer review only - http://bmjopen.bmj.com/site/about/guidelines.xhtml

References

1. World Health Organization, *Immunization Agenda 2030: A Global Strategy to Leave No One Behind*. 2020.
2. Vanderslott, S. and T. Marks, *Charting mandatory childhood vaccination policies worldwide.* Vaccine, 2021.
3. Gavi the Vaccine Alliance. *Sustainable Development Goals*. 2020 18 February, 2020; Available from: https://www.gavi.org/our-alliance/global-health-development/sustainable-development-goals.
4. Piot, P., et al., *Immunization: vital progress, unfinished agenda.* Nature, 2019. **575**(7781): p. 119-129.
5. World Health Organization, *Global Vaccine Action Plan 2011–2020* 2013: USA.
6. World Health Organization and UNICEF, *Global Immunization Vision and Strategy 2006-2015*. 2005: New York, NY.
7. World Health Organization. *Immunization coverage*. 2020 July 15, 2020 August 4, 2020]; Available from: https://www.who.int/news-room/fact-sheets/detail/immunization-coverage.
8. World Health Organization and UNICEF, *WHO-UNICEF estimates of DTP3 coverage*. 2020.
9. World Health Organization, *GVAP review and lessons learned: methodology, analysis and results of the stakeholder consultations*, in *Annex to the Global Vaccine Action Plan 2011-2020.* 2019: Geneva.
10. Bednarczyk, R.A., et al., *Protocol: Identification and evaluation of critical factors in achieving high and sustained childhood immunization coverage in selected low- and lower-middle income countries.* BMJ Open, 2021. **12**(e058321).
11. Shen, A.K., R. Fields, and M. McQuestion, *The future of routine immunization in the developing world: challenges and opportunities.* Global health, science and practice, 2014. **2**(4): p. 381-394.
12. World Health Organization, *Global Routine Immunization Strategies and Practices (GRISP): a companion document to the Global Vaccine Action Plan (GVAP)*. 2016, Geneva: World Health Organization.
13. World Health Organization, *Reaching Every District (RED): A guide to increasing coverage and equity in all communities in the African Region*. 2017.
14. Phillips, D.E., et al., *Determinants of effective vaccine coverage in low and middle-income countries: a systematic review and interpretive synthesis.* BMC health services research, 2017. **17**(1): p. 681-681.
15. Rodriguez, K., et al., *Critical success factors for routine immunization performance: A case study of Zambia 2000 to 2018.* Vaccine X, 2021. **11**(4): p. 100166.
16. Sakas, Z., et al., *Critical success factors for high routine immunization performance: A case study of Senegal.* Vaccine X, 2023. **14**(100296).
17. Hester, K.A., et al., *Critical success factors for high routine immunization performance: A case study of Nepal.* Vaccine X, 2022(7781): p. 100214.
18. Noor, A.M., *Country ownership in global health.* PLOS Global Public Health, 2022. **2**(2): p. e0000113.
19. WHO and UNICEF, *Immunization, DPT (% of children ages 12-23 months)*, W.a. UNICEF, Editor. 2022.
20. Carter, A., et al., *A framework for identifying and learning from countries that demonstrated exemplary performance in improving health outcomes and systems.* BMJ global health, 2020. **5**(12): p. e002938.
21. Exemplars in Global Health. *Making Better Decisions in Global Health: Understand Positive Outliers to Inform Policy and Practice*. 2021; Available from: https://www.exemplars.health/.
22. LaFond, A., et al., *Drivers of routine immunization coverage improvement in Africa: findings from district-level case studies.* Health policy and planning, 2015. **30**(3): p. 298-308.
23. World Health Organization and UNICEF, *WHO-UNICEF estimates of national immunization coverage (WUENIC)*. 2020: Geneva, Switzerland.
24. *Nepal Demographic Health Survey 2001, 2006, 2011, 2016, 2017*, DHS Program, Editor.
25. Damschroder, L.J., et al., *Fostering implementation of health services research findings into practice: a consolidated framework for advancing implementation science.* Implementation science : IS, 2009. **4**: p. 50-50.

For peer review only - http://bmjopen.bmj.com/site/about/guidelines.xhtml

26. Pfadenhauer, L.M., et al., *Making sense of complexity in context and implementation: the Context and Implementation of Complex Interventions (CICI) framework.* Implementation science : IS, 2017. **12**(1): p. 21-21.
27. Hester K, *Open Sciences Framework (OSF) page: Assessing sustainability factors for rural sanitation coverage following the SSH4A approach.* *https://osf.io/caz9b/?view_only=a1f16382d78b445ab34cc2afedca0cb0.*
28. Castillo Zunino, F., et al., *Association of childhood vaccination with family planning, healthcare access, and women education: analysis of Nepal, Senegal and Zambia.* medRxiv, 2023.
29. Smalley, H.K., et al., *Factors associated with vaccine coverage improvements in Senegal.* medRxiv, 2023.
30. Sakas, Z., et al., *The role of Zambia's expansive ICC in supporting efficient, effective, and evidence-based vaccine and health sector programming.* medRxiv, 2021: p. 2021.11.29.21267021.
31. Hester, K.A., et al., *Critical interventions for demand generation in Zambia, Nepal, and Senegal with regards to the 5C psychological antecedents of vaccination.* medRxiv, 2022: p. 2022.04.25.22274035.
32. Ozawa, S., L. Paina, and M. Qiu, *Exploring pathways for building trust in vaccination and strengthening health system resilience.* BMC health services research, 2016. **16**(Suppl 7): p. 639-639.
33. World Health Organization Maximizing Positive Synergies Collaborative Group, *An assessment of interactions between global health initiatives and country health systems.* The Lancet, 2009. **373**(9681): p. 2137-2169.
34. Hwang, A., et al., *Global Vaccine Action Plan Lessons Learned II: Stakeholder Perspectives.* Vaccine, 2020. **38**(33): p. 5372-5378.
35. Cherian, T., et al., *Global Vaccine Action Plan lessons learned III: Monitoring and evaluation/accountability framework.* Vaccine, 2020. **38**(33): p. 5379-5383.
36. Dixit, S.M., et al., *Addressing disruptions in childhood routine immunisation services during the COVID-19 pandemic: perspectives from Nepal, Senegal and Liberia.* BMJ Global Health, 2021. **6**(7): p. e005031.

FIGURE TITLES

Figure 1. A priori conceptual framework of the drivers of vaccine coverage

Figure 2. Revised conceptual framework for drivers of vaccine coverage

For peer review only

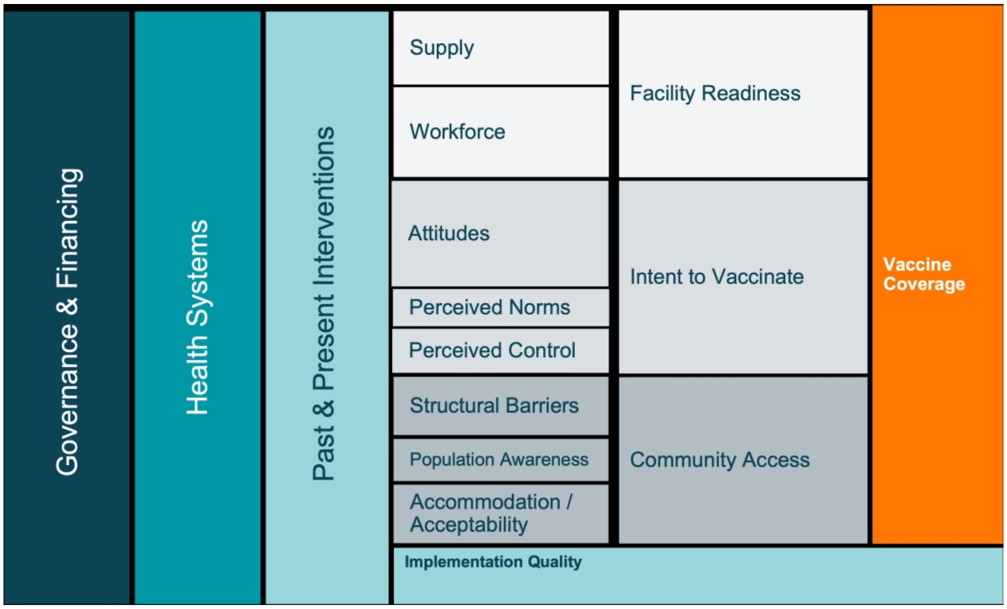

233x140mm (330 x 330 DPI)

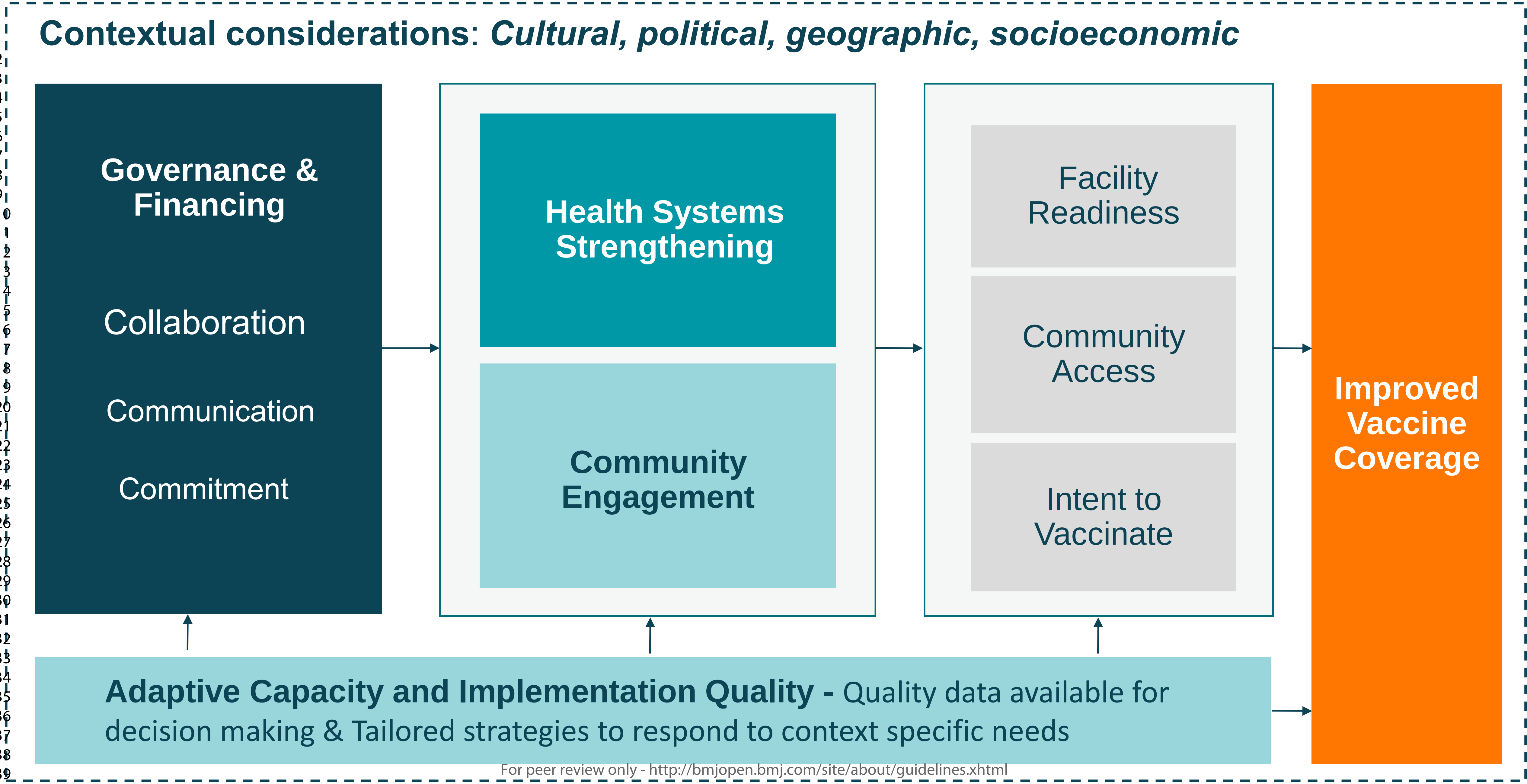
Contextual considerations: Cultural, political, geographic, socioeconomic
Governance & Financing
Collaboration
Communication
Commitment
Health Systems Strengthening
Community Engagement
Facility Readiness
Community Access
Intent to Vaccinate
Improved Vaccine Coverage
Adaptive Capacity and Implementation Quality - Quality data available for decision making & Tailored strategies to respond to context specific needs

Appendix 1: Coverage data for Nepal, Zambia, and Senegal

DHS DTP3 coverage in Zambia, by Province, 2000 – 2016

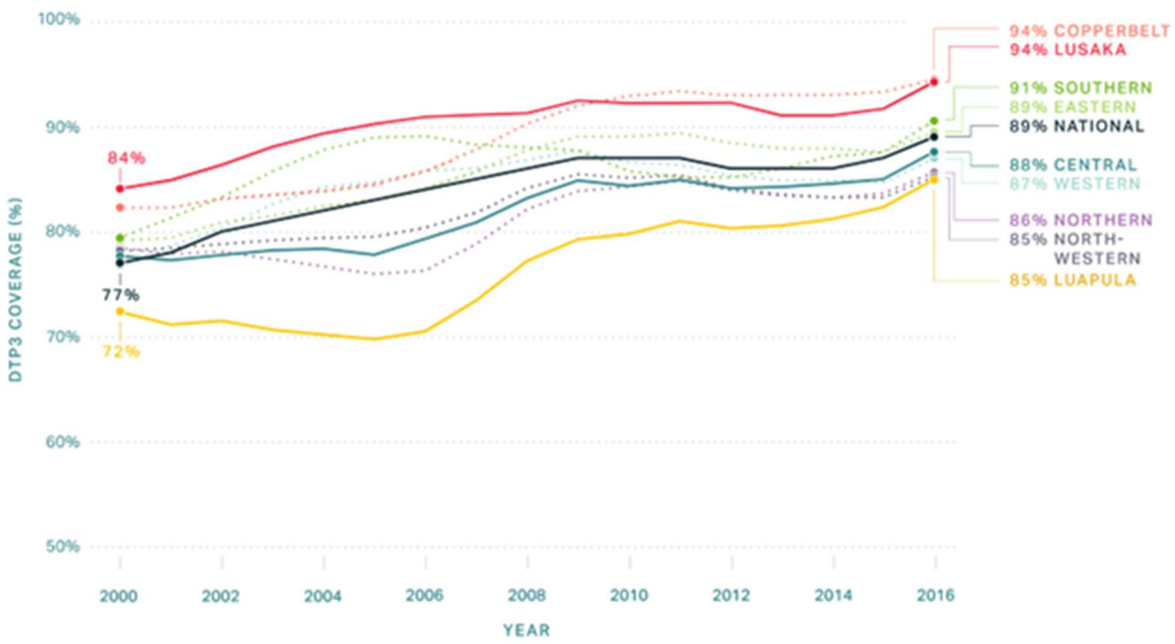

DHS DTP3 coverage in Nepal, by Province, 2000 – 2016

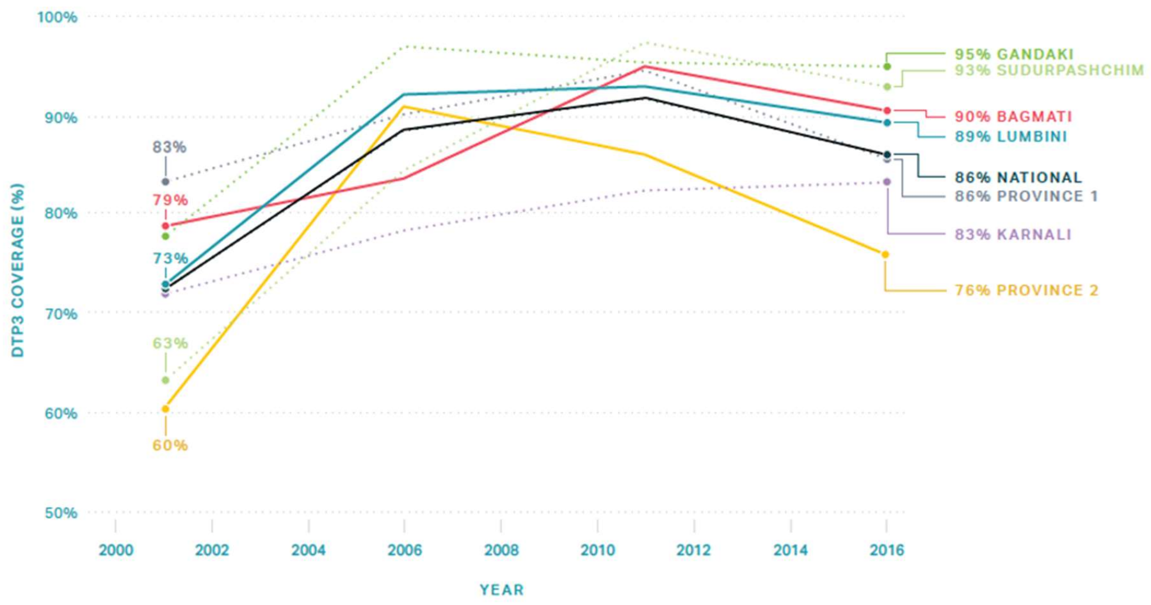

Data Source: Demographic and Health Survey (DHS)

DHS DTP3 coverage in Senegal, by Province, 2000 – 2016

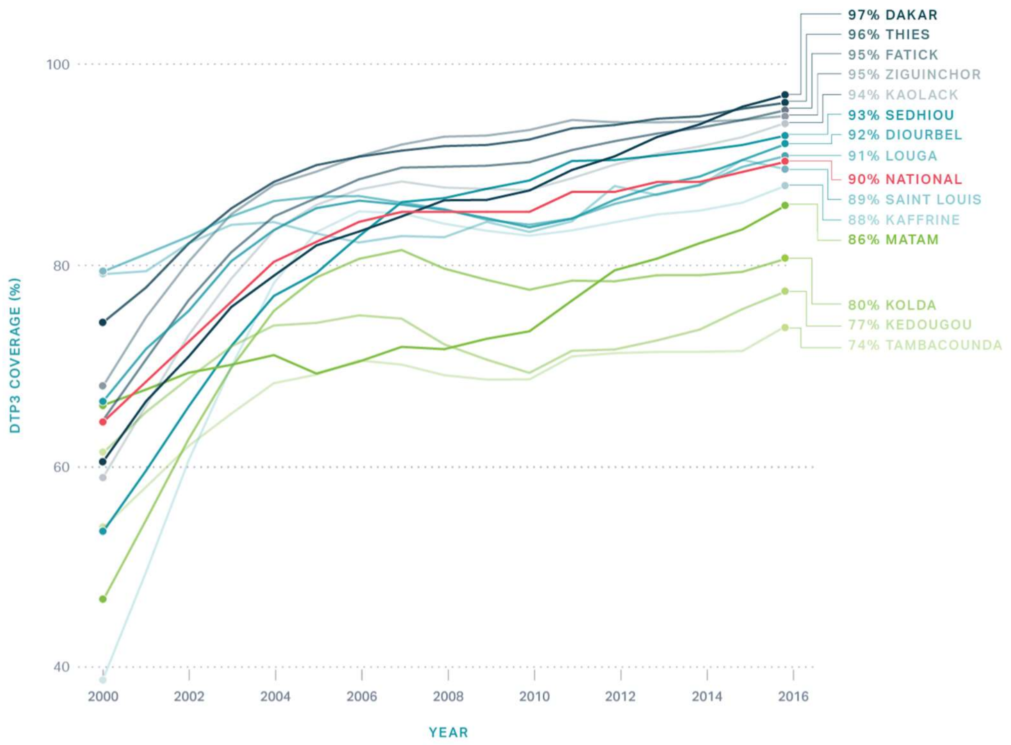

Data Source: IHME DPT Vaccine Coverage Geospatial Estimates

DTP3 coverage from 2000 to 2018 of Nepal, Senegal, and Zambia

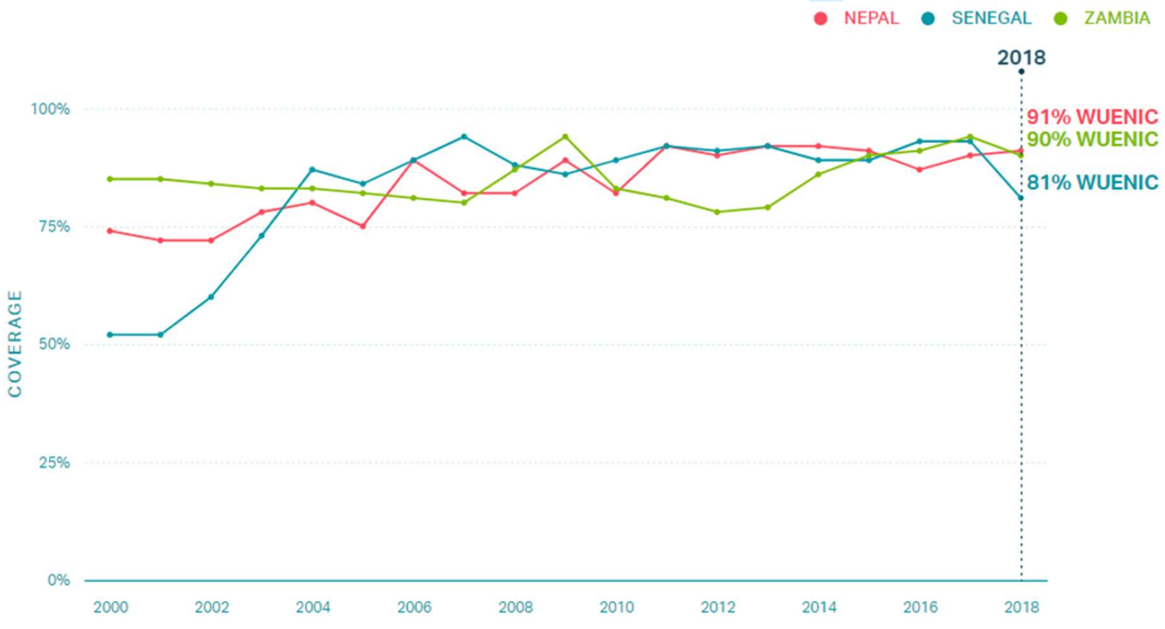

Date: ___________________________ *Interview Code:* ______ - ______ - ______

Appendix 2: Topic guides and tools for Vaccine Exemplars Project

VACCINE EXEMPLARS – NEPAL
Focus Group Discussion – Female Community Health Volunteers (FCHV)

Objectives:

- To understand the health services characteristics, including vaccine interventions, now and in the past;
- To identify the strategies behind vaccine programs;
- To identify role and responsibilities of the FCHVs in the immunization sector
- To identify FCHV immunization intervention-related motivations
- To understand barriers and facilitators to use of immunization services
- To understand the factors behind different levels of immunization coverage in their community, in the past and present

**PART 1 – BASIC INFORMATION**<br>*Can be asked by facilitator or filled out by note taker.*	
**A01**. **Researcher/Facilitator name:**	
**A01b. Note taker(s) name(s):**	
**A02. Consents provided by all:** Y / N<br><br>Time of consents ____ : ____ am/pm<br>Number of consents: ________	
**A03.** Date (dd/mm/yy): _____ /_____ / _____	**A04a.** Discussion start time ____ : ____ am/pm<br><br>**A04b.** Discussion end time ____ : ____ am/pm
**A05.** Recorder(s) used:	
**A06.** Location:	**A07.** Demographic surveys completed: Y / N

**PART 2a – INTRODUCTION**<br>*Facilitator to read aloud the following script:*

For peer review only - http://bmjopen.bmj.com/site/about/guidelines.xhtml

Date: ___________________________ *Interview Code:* ______ - ______ - ______

We are gathering today because we are interested in learning what caregivers like you think about childhood vaccines in *[COMMUNITY]*. My name is ______________, and I will be leading the discussion. This is ___________, and they will be taking notes.

We are specifically interested in learning about your thoughts, opinions, experiences, and perceptions related to the vaccination programming in your community. Please answer to the best of your ability. There are no right or wrong answers. Please share your views and opinions even if they are different from others, and you may choose to not answer any questions you are not comfortable with. You will be reimbursed (X) for traveling to this discussion, and food and drinks are provided here today. This should take about an hour and a half to two hours of your time. This discussion will be audio recorded, and used for research at Emory University and Center for Molecular Dynamics, Nepal (CMDN) to understand how Nepal has achieved high vaccination coverage. We will be recording to make sure we have accurately collected all of the information told to us. Is everyone okay with this discussion, and with being recorded?

Before we start the discussion, we have a survey for everyone to fill out. It asks you a few questions about yourself, and about how you feel about vaccines.

Pass out demographic survey; wait to continue with ground rules until all have been completed and handed back to note taker.

PART 2b – GROUND RULES

Wait for all group members to respond to the previous question, and check that all consent forms have been signed. Read aloud the following script:

For our discussion today, we have some ground rules that I would like everyone to follow:

1. Respect all group members, as well as their views and opinions. It is okay to disagree, but do so respectfully.
2. Everyone deserves a chance to speak. Please do not interrupt.
3. Do not use your mobile phones during the focus group discussion.
4. Please avoid side conversations.
5. Whatever is said here today is said in confidence, and should not be discussed outside of this group.

Does everyone agree with these rules? Do you have any you think we should add?

PART 2c – OPENING

Add any suggested rules to the list of ground rules, and wait for agreement on the rules. Read the following script aloud:

For peer review only

For peer review only - http://bmjopen.bmj.com/site/about/guidelines.xhtml

Date: ___________________________ *Interview Code:* ______ - ______ - ______

Are there any other questions before we begin? If everyone is ready, we will turn on the audio recorder and start the discussion.<br><br>*Turn on recorder once everyone is ready to start; Let everyone know that you have turned on the recorder.*<br><br>Now that the recorder is turned on, is everyone okay with recording our discussion today? To start, let's go around the room and introduce ourselves. What is your name, how did you get started in this position, and how many years have you been working in *[COMMUNITY]*? How many people do you serve in *[COMMUNITY]?*

**PART 3 – GENERAL HEALTH/HEALTH SYSTEM**<br>*Read the following script aloud:*	
I am now going to ask you some questions about health, and the health system in *[COMMUNITY].*	
Q1. Where do people in *[COMMUNITY]* receive preventative healthcare?<br><br>*[Intent: Develop understanding of the resources that are available and that are being utilized]*<br><br>*[CFIR – Inner Setting]*	*Probes:*<br>• *And health services?*<br>• *Door to door, health facility, hospital, etc.?*<br>• *Do people use health facilities/hospital services for primary health care or emergency care? Why? How often?*<br>• *What other types of health services do you use?*<br>○ *Traditional healers, family traditions/cures*<br>• *What are the different staff types (nurse, volunteer) that work in the healthcare services?*<br>• *What are these people's responsibilities?*

**PART 4 – FCHV Roles in Vaccinations**<br>*Read the following script aloud:*	
Now, I'd like to ask you all about vaccines – specifically, vaccines that are given to children under 1 year old.	
Q2.What is a FCHV's background and training?	• *What level of education do you have?*<br>• *What was your past experience in community organizations or health care before you became an FCHV?*<br>• *What kind of training do FCHVs receive?*<br>○ *Who trains FCHVs, and how often?*<br>○ *How long of a session do you have?*<br>○ *Is this verbal, do you have written manuals, visual manuals? How do FCHVs practice message delivery?*

For peer review only - http://bmjopen.bmj.com/site/about/guidelines.xhtml

Date: ___________________________ *Interview Code:* ______ - ______ - ______

Q3. Walk me through FCHV responsibilities in the vaccination process.<br><br>*[Intent: Identify current immunization activities and interventions; decision making process; identify key actors in the program]*<br><br>*[CFIR – Intervention, Inner Setting, Process]*	• *What vaccine promotion duties do FCHVs participate in?*<br>• *Is this part of routine immunization services, or an outreach effort?*<br>• *Who do you answer to?*<br>• *What kind of encouragement do you receive from your community/supervisor?*<br>• *How many children/families are FCHVs responsible for?*<br>• *What kind of difficulties do FCHVs have reaching out to families?*<br>• *How do FCHVs in your community reach different families?*<br>• *How far do FCHVs typically travel to reach families?*<br>• *How important is FCHV's work with vaccines relative to their other responsibilities?*<br>• *What kind of evaluation do FCHVs receive? What health indicators do FCHVs report on/are responsible for?*<br>• *What monitoring systems exist to track vaccination coverage?*<br>• *Are there any difficulties in data collection, recording, and/or submitting to the health post? What are they?*<br>• *What system do you use to track and report the number of children vaccinated?*<br>• *How have monitoring systems changed over the time you've been in your position?*<br>• *What are FCHVs' motivations for providing vaccines/increasing coverage?*<br>• *What kind of compensation do you receive for the work you are doing?*
Q4. *Are there any programs or methods that FCHVs utilize to motivate vaccination?*<br><br>*[Intent: Decision making process; current factors behind immunization]*<br><br>*[CFIR – Inner Setting]*	*Probes:*<br><br>• *Are these programs/methods national or community determined?*<br>• *Who determines the programming/methods?*<br>• *Who approves the programming/methods?*<br>• *What messages are promoted in this community regarding vaccines?*<br>• *Who formulates the messages?*<br>• *Who approves the messaging?*

For peer review only - http://bmjopen.bmj.com/site/about/guidelines.xhtml

Date: ___________________________ *Interview Code: ______ - ______ - ______*

	• *How are these messages communicated to community members?*
Q5. How does *[COMMUNITY]* view vaccines?<br><br>*[Intent: Decision making process; current factors behind immunization; demand-side]*<br><br>*[CFIR – Inner Setting]*	*Probes: What are the main facilitators to people getting vaccines in your community? Examples for reference:*<br>○ *Encouragement from health care workers*<br>○ *Shame*<br>○ *Geography*<br>○ *Enforcement*<br>○ *Trust of vaccinators/health care workers*<br>○ *Messaging/Awareness*<br>○ *Community expectation?*<br>○ *Seasonality*<br>○ *Fundamental right*<br>○ *Availability*<br>*What are the main barriers to people getting vaccines in your community? Examples for reference:*<br>○ *Geography*<br>○ *Mistrust of health care workers*<br>○ *Stock out*<br>○ *Cold chain*<br>○ *Vaccine hesitancy (ex. side effects)*<br>○ *Influence of community/religious leaders*<br>○ *Seasonality*<br>○ *Wastage*<br>• *How are these barriers addressed, if at all?*<br>• *Are there groups less likely to prioritize vaccines? Who and why?*<br>• *Are there children who receive infant vaccines (ex. DTP1) but don't receive others (ex. DTP2, DTP3)? Why?*<br>• *How are vaccines prioritized compared to other health interventions?*
Q7. Are vaccines available when and where they are needed?	*Probes:*<br>• *How about staff, syringes and other supplies?*<br>• *In what conditions do vaccines arrive?*<br>• *Where are vaccines stored?*<br>○ *Where is this storage relative to your clinic/where vaccines are administered?*<br>•

For peer review only - http://bmjopen.bmj.com/site/about/guidelines.xhtml

Date: ____________________________ *Interview Code:* ______ - ______ - ______

Q8. How has the immunization program changed over time?<br><br>*[Intent: Identify past immunization activities and interventions; identify previous key actors in the program; identify change points]*<br><br>*[CFIR – Intervention, Outer Setting, Inner Setting, Process]*	*Probes:*<br>• *What changed?*<br>○ *Demand from community?*<br>○ *Supply?*<br>○ *Improved systems?*<br>○ *Training?*<br>○ *Governmental emphasis?*<br>○ *Increased funding*<br>• *When did this happen? About what year was this?*<br>○ *What other events happened around this time?*<br>▪ *Political*<br>▪ *Infrastructure changes*<br>▪ *Other health issues*<br>• *Can you think of any certain events that increased or decreased vaccinations?*<br>○ *Strikes*<br>○ *Extreme weather*<br>• *How have FCHV's motivations to vaccinate changed over time?*<br>○ *Has this been affected by any certain events? (**NOTE: REFERENCE BACK TO ANSWER TO PREVIOUS PROBE**)*
Q9. How have outbreaks of diseases affected mandatory vaccination rates?<br><br>*[Intent: Identify past disease events that may have impacted past vaccination events; identify change points]*<br><br>*[CFIR – Intervention, Outer Setting, Inner Setting, Process]*	*Probes:*<br>• *Did vaccination rates increase or decrease?*
Q10. Can you think of a specific intervention that you believe contributed to increased vaccine coverage?<br><br>*[Intent: Identify past immunization activities and interventions; identify previous key actors in the program; identify change points]*<br><br>*[CFIR – Intervention, Outer Setting, Inner Setting, Characteristics of Individuals, Process]*	*Probes:*<br>• *How was this intervention implemented in your community?*<br>• *What kind of training was received to prepare different people for this intervention?*<br>• *What went well in the implementation process of this intervention? were the facilitators to implementation?*<br>• *What was challenging in the implementation process of this*

For peer review only - http://bmjopen.bmj.com/site/about/guidelines.xhtml

Date: ___________________________ *Interview Code:* ______ - ______ - ______

	intervention? What were the barriers to implementation?
Q11. What was difficult about improving vaccine coverage in this community at that time?<br><br>*[Intent: Identify past immunization activities and interventions; identify previous key actors in the program]*<br><br>*[CFIR – Intervention, Outer Setting, Inner Setting, Characteristics of Individuals, Process]*	*Probes:*<br>• *How did this affect your community?*<br>• *What was implemented to address these specific difficulties?*<br>• *Why do you think this was successful?*<br>• *How were different people motivated for this success?*

**PART 5 – POLICY**	
Q12. What, if any, vaccines do children need to receive?<br><br>*[Intent: Identify current immunization activities and interventions]*<br><br>*[CFIR – Intervention, Outer Setting, Inner Setting]*	*Probes:*<br>• *Are the requirements clear?*<br>• *What vaccinations are required for children to be allowed to go to school?*<br>• *What affect have these laws/requirements had on FCHVs' jobs?*
Q13. How have these requirements changed over time?<br><br>*[Intent: Identify past immunization activities and interventions; identify previous key actors in the program; identify change points]*<br><br>*[CFIR – Intervention, Outer Setting, Inner Setting, Process]*	*Probes:*<br>• *Why did requirements change?*<br>• *About what year/time was this?*<br>• *What other events happened around this time?*<br>• *Who worked with your community to adapt to the new regulations?*<br>• *What kind of assistance/training was received to enable you to deliver according to new requirements?*

**PART 4 – CLOSING**<br>*Read the following script aloud:*
Those are all of my questions. What other information about vaccinations do you have that you feel would be important for me to know? Do you have any other questions or comments you'd like to add before we end the discussion?<br><br>*Wait for group to answer.*

For peer review only - http://bmjopen.bmj.com/site/about/guidelines.xhtml

Date: ___________________________ *Interview Code:* ______ - ______ - ______

Thank you all very much for your time and for answering my questions today. You have all provided great information that will be of help during our research. If you have any other questions or concerns, please feel free to contact _____________. You can also find this information on the consent document that you signed when we started the discussion. If there are no other questions or concerns, then I will turn off the recorder, and we will give travel reimbursement. Thank you again for your help.

For peer review only

For peer review only - http://bmjopen.bmj.com/site/about/guidelines.xhtml

Date: ___________________________ *Interview Code:* ______ - ______ - ______

Key Informant/In-depth Interview Guide-Community Leaders

Objectives:

- To understand their role, if any, as it relates to health interventions, specifically immunization coverage
- To identify key community stakeholders who have a role in immunization programming
- To understand the cultural context and environmental context of the selected community
- To understand what drives community acceptance of immunizations
- To understand the motivations of key community stakeholders in vaccine intervention characteristics during country-specific change points in DTP1/DTP3 coverage
- To identify the strategies behind current immunization programs to inform current context
- To identify previous immunization activities — including specific interventions, delivery systems, policies, facilitators and barriers — that were key during change points in the DTP1/DTP3 curve
- To understand specific barriers and facilitators to implementation of interventions in the immunization sector

**PART 1 – DEMOGRAPHIC AND BASIC INFORMATION**<br>*Can be asked by interviewer or filled out by note taker.*	
**A01**. **Researcher/Interviewer name:**	
**A01b. Note taker(s) name(s):**	
**A01c. Translator name:**	
**A01d. Copy belongs to:**	
**A02. Consent given:** Y / N<br><br>Time of consent ____ : ____ am/pm<br>Time zone: _____	
**A03.** Date (dd/mm/yy): _____ /_____ / _____	**A04a.** Interview start time ____ : ____ am/pm<br>**A04b.** Interview end time ____ : ____ am/pm<br>Time zone: _____<br><br>**A05**. Recorder(s) used:
**A06.** Name of Respondent interviewed:	**A07.** Designation/title of Respondent interviewed:
**A08.** Organization name:	**A09.** Years in Organization:

For peer review only

For peer review only - http://bmjopen.bmj.com/site/about/guidelines.xhtml

Date: ___________________________ *Interview Code:* ______ - ______ - ______

PART 2 – INTERVIEW INTRODUCTION

<u>Interviewer Script</u>: Thank you very much for your willingness to meet with us today and share your experience and knowledge regarding the immunization program in ______ (COUNTRY). I'd like to begin by learning about you and your role related to the immunization program.

1. Can you tell me about your role in ______ (THIS COMMUNITY) ?

Follow up:

- *How does your role relate to health care programming?*
- *How does your role relate to the immunization program?*
- *Responsibilities (past and present)*
- *What are the contributions you feel you've made in the immunization programming in your community?*
- *What has motivated you to make these contributions?*

2. Who are other community leaders/stakeholders who have had an impact on the immunization program?

Follow up:

- *What role did they play?*
- *What were their key contributions?*
- *What motivated their involvement?*
- *What were their top three incentives for performing well?*
- *What are the stakeholders' party affiliation?*
- *Are their positions relevant to vaccinations?*

PART 3 – HEALTH SYSTEM & VACCINES

Read the following script aloud:

<u>Interviewer Script</u>: Now I'd like to learn about the health care services in your community, and how immunization coverage plays a part. I'd also like to hear a bit more about community attitudes towards immunizations.

3. How does *[COMMUNITY]* view vaccines?

[Intent: Decision making process; current factors behind immunization; demand-side]

For peer review only - http://bmjopen.bmj.com/site/about/guidelines.xhtml

Date: ___________________________ Interview Code: ______ - ______ - ______

- *Probes: What are the main facilitators to people getting vaccines in your community]?*
  - *Encouragement from health care workers*
  - *Shame*
  - *Geography*
  - *Enforcement*
  - *Trust of vaccinators/health care workers*
  - *Messaging*
  - *Community expectation?*
  - *Seasonality*
- *What are the main barriers to people getting vaccines in your community?*
  - *Geography*
  - *Mistrust of health care workers*
  - *Stock out*
  - *Cold chain*
  - *Vaccine hesitancy (ex. side effects)*
  - *Influence of community/religious leaders*
  - *Seasonality*
  - *Wastage*
- *How are these barriers addressed?*
- *Are there groups less likely to prioritize vaccines? Who and why?*
- *Are there children who receive infant vaccines (ex. DTP1) but don't receive others (ex. DTP2, DTP3)? Why?*

How are vaccines prioritized compared to other health interventions/measures?

4. How have outbreaks of diseases affected mandatory vaccination rates?

Probes:

Did vaccination rates increase or decrease?

5. Compared to other government responsibilities - like public safety, roads and infrastructure, education – how important are vaccines?

Probes:

- *Why are they important/not important?*
- *What is the most important to you / [COMMUNITY]?*
- *What is the least important to you / [COMMUNITY]?*
- *Who do you believe is responsible for providing vaccines?*
  - *Government?*

NGOs and aid agencies?

For peer review only - http://bmjopen.bmj.com/site/about/guidelines.xhtml

Date: ___________________________ *Interview Code:* ______ - ______ - ______

6. How has the *[COMMUNITY'S]* ability to provide vaccines changed over time?

Probes:

- *What changed?*
  - *Demand from community?*
  - *Supply?*
  - *Improved systems?*
  - *Training?*
  - *Governmental emphasis?*
- *About what year was this?*
  - *What other events happened around this time?*
    - *Political*
    - *Infrastructure changes*
    - *Other health issues*
- *Can you think of any certain events that increased or decreased vaccinations?*
  - *Strikes*
  - *Extreme weather*
  - *Outbreaks*
    - *What diseases?*
      - *Vaccine preventable (flu, measles, mumps, rubella, pertussis etc.)*

Non-vaccine preventable?

7. What are specific interventions/programming shifts that have happened in the last ten years (NOTE: depending on how long they have been in their positions)?

Probes:

- *What intervention had the greatest impact?*
- *How was this intervention implemented in your community?*
- *How did people react to this intervention?*
- *What were the facilitators to uptake?*
- *What were barriers?*

8. What factors have contributed to the sustainability of the immunization program?

   a. Organizational capacity: Were there organizational systems in place to support the program needs?

For peer review only - http://bmjopen.bmj.com/site/about/guidelines.xhtml

Date: ____________________________ *Interview Code:* ______ - ______ - ______

b. Communication: How does the immunization program maintain community support or awareness of the issue of childhood vaccines?
Follow-up:<br><br>*To what extent is the immunization program resilient from rumors and misinformation regarding immunizations?*<br>*How could resilience be improved?*
c. Media: How is vaccination portrayed and discussed by the media?
Follow-up:<br><br>• *What role did the media play in improved coverage?*<br>• *How did the media become involved and how was that involvement sustained?*
d. Strategic planning: To what extent is there planning for future resource needs?
9. What are the greatest risks to sustained coverage?
10. What lessons have you learned from working on/creating the immunization program?
**PART 4 – CLOSING**<br>*Read the following script aloud:*
Those are all of my questions. Do you have any other questions or comments you'd like to add before we end the discussion?<br><br>*Wait for individual to answer.*<br><br>Thank you much for your time and for answering my questions today. You have provided great information that will helpful during our research. If you have any other questions or concerns, please feel free to contact ______________. You can also find this information on the consent document that

For peer review only - http://bmjopen.bmj.com/site/about/guidelines.xhtml

Date: ___________________________ *Interview Code:* ______ - ______ - ______

you signed when we started the discussion. If there are no other questions or concerns, then I will turn off the recorder, and you are free to leave as you please. Thank you again.

For peer review only

For peer review only - http://bmjopen.bmj.com/site/about/guidelines.xhtml

Date: ___________________________ *Interview Code:* ______ - ______ - ______

VACCINE EXEMPLARS
Focus Group Discussion – Parents/Caregivers

Note: Do not read red italics aloud

Objectives:

- To understand the vaccine intervention characteristics during country-specific change points in DTP1/DTP3 coverage
- To identify parental/caregiver motivation behind interventions during the change point within the immunization program
- To explore decision-making process of parents/caregivers in the change point
- To identify previous immunization activities - including specific interventions, delivery systems, policies, facilitators and barriers – that were key during change points in the DTP1/DTP3 curve and to understand barriers and facilitators to implementation
- To understand the current factors behind different levels of immunization coverage in their community
- To understand the factors behind different levels of immunization coverage in their community at change point

Framework Application Key:
WHAT – Solutions
HOW – Mechanisms of Change
WHY – Problem Identification

**PART 1 – BASIC INFORMATION**<br>*Can be asked by facilitator or filled out by note taker.*	
**A01. Researcher/Facilitator name:**	
**A01b. Note taker(s) name(s):**	
**A01c. Translator or observer:**	
**A01d. Copy belongs to:**	
**A02. Consents provided by all:** Y / N<br><br>Time of consents ____ : ____ am/pm<br>Number of consents: _______	
**A03.** Date (dd/mm/yy): _____ /_____ / _____	**A04a.** Discussion start time ____ : ____ am/pm<br><br>**A04b.** Discussion end time ____ : ____ am/pm<br><br>Time zone: _____
**A05.** Group (check all that apply):<br>☐ Mothers (under age 25)<br>☐ Mothers/Grandmothers (over age 25)<br>☐ Fathers<br>☐ Other - _____________	**A06.** Recorder(s) used:

For peer review only - http://bmjopen.bmj.com/site/about/guidelines.xhtml

Date: ___________________________ *Interview Code:* ______ - ______ - ______

**A07.** Location:	**A08.** Demographic surveys completed: Y / N

PART 2a – INTRODUCTION
Facilitator to read aloud the following script:

We are gathering today because we are interested in learning what caregivers like you think about childhood vaccines in *[COMMUNITY]*. My name is ______________, and I will be leading the discussion. This is ___________, and they will be taking notes.

We are specifically interested in learning about your thoughts, opinions, experiences, and perceptions related to the vaccines your child/grandchild has or has not received. Please answer to the best of your ability. There are no right or wrong answers. Please share your views and opinions even if they are different from others, and you may choose to not answer any questions you are not comfortable with. You will be reimbursed (X) for traveling to this discussion, and food and drinks are provided here today. This should take about an hour to an hour and a half of your time. This discussion will be audio recorded, and used for research at Emory University and *[LOCAL PARTNER]* to understand how *[COUNTRY]* has achieved high vaccination coverage. We will be recording to make sure we have accurately collected all of the information told to us. Is everyone okay with this discussion, and with being recorded?

Before we start the discussion, we have a survey for everyone to fill out. It asks you a few questions about yourself, and about how you feel about vaccines.

Pass out demographic survey; wait to continue with ground rules until all have been completed and handed back to note taker.

PART 2b – GROUND RULES
Wait for all group members to respond to the previous question, and check that all consent forms have been signed. Read aloud the following script:

For our discussion today, we have some ground rules that I would like everyone to follow:

6. Respect all group members, as well as their views and opinions. It is okay to disagree, but do so respectfully.
7. Everyone deserves a chance to speak. Please do not interrupt.
8. There are no right or wrong answers.
9. Please avoid side conversations.
10. Whatever is said here today is said in confidence, and should not be discussed outside of this group.

Does everyone agree with these rules? Do you have any you think we should add?

PART 2c – OPENING
Add any suggested rules to the list of ground rules, and wait for agreement on the rules. Read the following script aloud:

For peer review only

For peer review only - http://bmjopen.bmj.com/site/about/guidelines.xhtml

Date: ____________________________ *Interview Code:* ______ - ______ - ______

Are there any other questions before we begin? If everyone is ready, we will turn on the audio recorder and start the discussion.<br><br>*Turn on recorder once everyone is ready to start; Let everyone know that you have turned on the recorder.*<br><br>Now that the recorder is turned on, is everyone okay with me recording our discussion today? To start, let's go around the room and introduce ourselves. What is your name, how many children do you have, and what are their ages?

**PART 3 – GENERAL HEALTH**<br>*Read the following script aloud:*	
I am now going to ask you some questions about health in general, and health in *[COMMUNITY]*.	
Q1. Please tell me about how people in *[COMMUNITY]* receive healthcare and health services?<br><br>*[Intent: Develop understanding of the resources that are available and that are being utilized; health literacy; general health interventions]*	*Probes:*<br>• *Local health clinic, nurse, doctor, community health workers?*<br>• *Friends and family?*<br>• *Where do you seek healthcare?*<br>○ *Why?*<br>• *Do you go to prevent disease, or only when you are sick?*<br>○ *Why?*<br>• *When do you seek health related information?*<br>○ Why?
Q2. How does *[COMMUNITY]* help families to stay healthy?<br><br>*[Intent: Develop understanding of the resources that are available and that are being utilized; health literacy; general health interventions]*	*Probes:*<br>• *What are the health activities you take part in within [COMMUNITY]?*<br>• *What are the health activities your family members take part in within [COMMUNITY]?*<br>• *What do you believe your role is in regards to vaccinations?*

**PART 4 – VACCINES**<br>*Read the following to participants:*
We are now going to talk about vaccines. Specifically, the vaccines your child was given before they were 2 years old.

For peer review only - http://bmjopen.bmj.com/site/about/guidelines.xhtml

Date: ___________________________ *Interview Code:* ______ - ______ - ______

Q3. What has *[COMMUNITY]* been told about vaccines?<br><br>*[Intent: Health literacy; decision making process of parents; motivation]*	*Probes:*<br>• *How did you get this information?*<br>○ *Mass media*<br>○ *Verbally (family, friends, community health worker, etc.)*<br>○ *Paper form*<br>• *What do you think about this information?*<br>• *How much do you trust the source of vaccination information?*<br>○ *Why?*
Q4. What has *[COMMUNITY]* been told about the diseases vaccines prevent?<br><br>*[Intent: Health literacy; decision making process of parents; motivation]*	*Probes:*<br>• *How does your knowledge of this disease make you feel about vaccines (in general, or the vaccine that prevents the disease)*<br>• *Do you know what this disease is?*<br>• *Have you seen anyone get this disease before?*<br>• *Who told you about these diseases?*<br>• *Do people get this disease still?*
Q5. What do [*PROVIDERS*] at [*CLINIC*] tell mothers/caregivers about vaccinations?<br><br>*[Intent: Characteristics of community possibly leading to decision making process]*	*Probes:*<br>• *What vaccinations do providers talk about when you go to the clinic?*<br>• *Who else talks about vaccines?*<br>○ *Community health workers*<br>○ *Community leaders*<br>○ *Religious leader*<br>○ *Family (who)?*<br>○ *Friends, etc.?*
Q6. What affects mothers/caregivers decision to vaccinate or not to vaccinate their child?<br><br>*[Intent: Demand-side factors/motivation; decision making process]*	*Probes:*<br>• *How is this received by other community members?*<br>• *Is this needed for school?*<br>• *What are [COMMUNITY] beliefs surrounding vaccination?*<br>• *How convenient is it for caregivers to vaccinate children?*<br>• *Do you know of any leaders/movements/organizations that promote vaccination?*<br>○ *Any that oppose vaccination?*<br>• *Is it expensive to get vaccinated?*<br>• *When you go for vaccines, do you get other services or goods?*<br>○ *Examples – food, supplies for children, check-ups, etc.*

For peer review only - http://bmjopen.bmj.com/site/about/guidelines.xhtml

Date: ___________________________ Interview Code: ______ - ______ - ______

Q7. How important is it in *[COMMUNITY]* to get vaccinations for children?	*Probes:*<br>• *Why/why not?*
Q8. Tell me about a normal visit to get children vaccinated in *[COMMUNITY]*.<br><br>*[Intent: Find possible interventions in place within the community]*	*Probes:*<br>• *How far did you have to travel?*<br>○ *What kind of transportation did you use (walk, drive, bus, etc.)*<br>• *Did you have to present any documentation?*<br>• *About how long did getting your child vaccinated take?*<br>• *Were more than one of children getting vaccinated? Why/why not?*<br>• *How much time did it take to get to the vaccination site?*<br>• *How much of this time was spent waiting in line?*<br>• *How did this affect your daily routine?*<br>• *Was this a special vaccination day?*<br>○ *How did you know about this?*<br>○ *Are there any reminder systems?*<br>▪ *How do they work?*<br>▪ *Do you pay attention to these reminders?*<br>▪ *When did these reminders start?*<br>○ *Who told you about this?*

**PART 4 – HISTORY AND POLICY**	
Q9. Have the opinions of caregivers/mothers in *[COMMUNITY]* changed over time?<br><br>*[Intent: Develop entire history; set up a timeline of how vaccination has changed]*	*Probes:*<br>• Why?<br>• *What changed for you?*<br>• *About what year was this?*<br>○ *What other events happened around this time?*
Q10. What requirements are there for childhood vaccines?<br><br>*[Intent: Knowledge of policy and policy implications]*	*Probes:*<br>• *How did you learn about these requirements?*<br>• *Have you ever had to present your child's vaccination record to have access to services?*<br>○ *What vaccinations are required for children to be allowed to go to school?*<br>• *How do you feel about these laws/requirements?*

For peer review only - http://bmjopen.bmj.com/site/about/guidelines.xhtml

Date: ___________________________ *Interview Code:* ______ - ______ - ______

	• *What affect have these laws/requirements had on your life?*
Q11. Compared to other government responsibilities - like public safety, roads and infrastructure, education – how important are vaccines?	*Probes:*<br><br>• *Why are they important/not important?*<br>• *What is the most important to you / [COMMUNITY]?*<br>• *What is the least important to you / [COMMUNITY]?*<br>• *Who do you believe is responsible for providing vaccines?*<br>○ *Government?*<br>○ *NGOs and aid agencies?*

**PART 4 – CLOSING**<br>*Read the following script aloud:*
Those are all of my questions. Do you have any other questions or comments you'd like to add before we end the discussion?<br><br>*Wait for group to answer.*<br><br>Thank you all very much for your time and for answering my questions today. You have all provided great information that will helpful during our research. If you have any other questions or concerns, please feel free to contact _____________. You can also find this information on the consent document that you signed when we started the discussion. If there are no other questions or concerns, then I will turn off the recorder, and you are free to leave as you please. Thank you again.

For peer review only

For peer review only - http://bmjopen.bmj.com/site/about/guidelines.xhtml

Date: ___________________________ *Interview Code:* ______ - ______ - ______

VACCINE EXEMPLARS
Key Informant Interview Guide – Tool 1b ALL COUNTRIES

Objectives:

- To understand the vaccine intervention characteristics during country-specific change points in DTP1/DTP3 coverage
- To identify key internal and external actors, stakeholders, and partnerships in the immunization program both now and in the past
- To identify the strategies behind current immunization programs to inform current context
- To identify previous immunization activities that were key during change points in the DTP1/DTP3 curve
- To understand key barriers and facilitators of interventions in the immunization sector

**PART I – DEMOGRAPHIC AND BASIC INFORMATION**<br>*Can be asked by interviewer or filled out by note taker.*	
**A01. Researcher/Interviewer name:**	
**A01b. Note taker(s) name(s):**	
**A01c. Translator name:**	
**A01d. Copy belongs to:**	
**A02. Consent given:** Y / N<br>Time of consent ____ : ____ am/pm	
**A03.** Date (dd/mm/yy): _____ /_____ / _____	**A04a.** Interview start time ____ : ____ am/pm<br>**A04b.** Interview end time ____ : ____ am/pm<br>**A05**. Recorder(s) used:
**A06.** Name of Respondent interviewed:	**A07.** Designation/title of Respondent interviewed:
**A08.** Organization name:	**A09.** Years in Organization:

For peer review only - http://bmjopen.bmj.com/site/about/guidelines.xhtml

Date: ___________________________ *Interview Code:* ______ - ______ - ______

PART II – INTERVIEW INTRODUCTION

<u>Interviewer Script</u>: Thank you very much for your willingness to meet with us today and share your experience and knowledge regarding the immunization program in ______ (COUNTRY). I'd like to begin by learning about you and your role related to the immunization program.

10. Can you tell me about your role in ______ (ORGANIZATION) ?

~ Individual Setting ~

Follow up:

- *How does your role relate to the immunization program?*
- *Responsibilities (past and present)?*
- *How long have you been working on vaccines/with the immunization program(s)?*
- *What drew your interest to vaccines/immunization programs? What type of projects have you worked on?*

PART IIa – IMMUNIZATION PROGRAM

Read the following script aloud:

<u>Interviewer Script</u>: Now I'd like to learn about the immunization program, and about the increase in coverage since the year 2000. I'd also like to know about what relevant events that may have contributed to this change, and how the immunization program is funded.

11. Will you tell me a bit about the current state of the vaccination program in ______ (COUNTRY). ?

~ Outer Setting ~

Follow up:

- *Who are the main decision makers at this point in time?*

12. Who are the main government actors involved in the current immunization program?

~ Outer Setting ~
~ Engaging; Process ~
~ RQ2. Political Will ~
~ RQ5. Knowledge and Resource Sharing ~
~RQ6. Workforce ~

Follow up:

- *If there were multiple governmental agencies, how were they related and what responsibility does each take in regard to vaccination efforts?*
- *What other activities did governmental agencies engaged in vaccination undertake?*
- *Which organizations or ministries are responsible for how the immunization system performs from top to bottom, inclusive of the regional level?*

For peer review only - http://bmjopen.bmj.com/site/about/guidelines.xhtml

Date: ___________________________ *Interview Code: ______ - ______ - ______*

<table>
<tr><td>13. How does the chain of command/structure within the immunization sector work?</td></tr>
<tr><td>Follow up:
• What kind of directives come from the ministry?
• How do you report changes that are made in programming?
• Who do you report this to?
• What kind of changes in programming do people at different levels (national, provincial, district, health facility) contribute to?
• How are you made aware of changes in personnel within the immunization program?
• Who within the program reports to you?
• Who do you report to?</td></tr>
<tr><td>14. Who are the main partner organizations involved in the current immunization program?
(NOTE: DON'T EXPECT TOO MUCH FOLLOW UP FOR G, H, I)
~ Outer Setting ~
~ Engaging; Process ~
~ RQ5. Knowledge and Resource Sharing ~
~RQ6. Workforce ~</td></tr>
<tr><td>Follow up:
• What are their roles?
• What are the local donor, individuals, and local NGOs that contribute to the sector?
• Who makes up the internal and external-workgroups? Can you define these actors - Ministries, INGOs, local NGOs, private sector?
• How do the organizations/stakeholders (individual actors) coordinate with each other?</td></tr>
<tr><td>15. When do you think the vaccination program started to show improvements?
~ Outer Setting ~
~ Inner Setting~</td></tr>
<tr><td>Follow up:
• What happened in ______ (YEAR(S)) to affect vaccine coverage?
• Why do you think this is when the program improved? What are the events?
• How would you describe the environment surrounding the vaccine program during this period? How did this make the change possible?
• What do you think catalyzed this change? (If examples needed, use beneficiary needs, policy, legislation, individual champions, politicians?
• Who were the main decision makers at this point in time? What were their incentives for performing well? Which party was in power at this time?
• Do other individuals agree with this assessment, or is there something else that is believed to be behind change? For example, do politicians think this is also the driver behind the increase in coverage?
• Which, of the reasons you gave me, do you consider the most important in improving immunization coverage? Why?</td></tr>
<tr><td>16. (NOTE: ONLY SHOW/THIS to CONNECT TO PREVIOUS ANSWER) This graph shows the DTP1/DTP3 coverage trend since 2000 in ______ (COUNTRY). In addition to what you've already said, what else can you tell us about these time points where coverage increased? What important or relevant events do you believe contributed to the increases?</td></tr>
<tr><td>17. Who were the key stakeholders during the time of improvement?</td></tr>
</table>

For peer review only - http://bmjopen.bmj.com/site/about/guidelines.xhtml

Date: ___________________________ *Interview Code:* ______ - ______ - ______

~ Outer Setting ~<br>*~ RQ2. Political Will ~*<br>*~ RQ5. Knowledge and Resource Sharing ~*<br>*~ RQ6. Workforce ~*
Follow up:<br>• *Ministries, partner organizations?*<br>• *What role did they play?*<br>• *If there were multiple governmental agencies, how were they related and what responsibility does each take in regard to vaccination efforts?Who made up the internal and external-workgroups?*<br>○ *Can you define these actors* - *Ministries, INGOs, local NGOs, private sector?*<br>• *What other activities did governmental agencies engaged in vaccination undertake?*<br>• *What has the turnover in these positions been like?*<br>• *Have the key people identified been in the same position for extended periods? What was the mandated term?*<br>○ *What are their motivations in involvement?*<br>○ *Can you connect me to XXX?*
•
18. What do you consider to be the main barriers to the improvement in immunization coverage in ______ (*YEAR(S)*) before the increase?<br>***~ Outer Setting ~***
Follow up:<br>• *How was each barrier addressed?* ***(NOTE: REFER BACK TO SPECIFIC BARRIERS MENTIONED BY PARTICIPANT)***<br>• *What was the motivation behind addressing the barriers?*
19. What were the main sources of funding for immunization programming during this time period? ***(NOTE: FOR TOOLS H and I, JUST ASK THE MAIN QUESTION. DON'T EXPECT TO PROBE)***<br>***~ Available Resources; Inner Setting ~***<br>*~ RQ2. Political Will ~*

For peer review only - http://bmjopen.bmj.com/site/about/guidelines.xhtml

Date: ___________________________ *Interview Code: ______ - ______ - ______*

Follow up:

Ask these questions at each level -

- *Is there a fund specifically for vaccines, or is there just a general health fund?*
  - *How are these funds spent?*
- *How much of the overall health budget is allocated to vaccination activities?*
- *Is there an overall vaccine budget, or is it separated by vaccine?*
- *Are there financial barriers to increasing the vaccination rates? If so, what?*
  - *Which specific barrier(s) require more funding?*
- *Which level of government is responsible for spending the resources* ***? (EXAMPLES: Province, district, health facility)***
  - *How much power do you have to spend the money the way that you want to?*
- *Is there an advisory board/audit that checks how money is spent? Does the advisory board check the expenses for each level; regional, district, health center?*
- *Are there any restrictions on how contracts are assigned?* ***(EXAMPLES: Grants, distributors for supplies or vaccine, etc.)***
- *Can you share supporting documentation with me? If not, where can I access this, and do you know who may be able to assist me?*

For peer review only

For peer review only - http://bmjopen.bmj.com/site/about/guidelines.xhtml

Date: ___________________________ *Interview Code:* ______ - ______ - ______

PART III – INTERVIEWER TO ASK SECTIONS BELOW AS DETERMINED BY TITLE/DEPARTMENT OF RESPONDENT:

Central -

A. Minister of Health, Minister Representative, or General Secretary …………………...….…. 7
B. Ministry Departments of Epidemiology, Surveillance, EPI, Vaccines, Family Health…….. 10
C. Ministry Departments (or liaisons) of Planning, Statistics, Research, Human Resources, Communication and Development ……………………………………………………….… .20
D. Ministry Departments (or liaisons) of Infrastructure, Equipment, Finance ……………...…. 24
E. Partner Organizations, such as WHO, UNICEF, CDC, USAID etc.; NITAG, Professional Organization members ……………………………………………………...………….. 26

Subnational -

G. Region Head, Surveillance, Prevention, and Vaccination Personnel …………………….… 30
H. District Head, Surveillance, Prevention, Vaccination Personnel ……………………....….38
I. Clinic Head ……………………………………………………………………………….….46
J. Community Leader……………………………………………………………………………53

PART IV – CONCLUSION ………………………………………………………………………57

For peer review only

For peer review only - http://bmjopen.bmj.com/site/about/guidelines.xhtml

Date: ___________________________ *Interview Code:* ______ - ______ - ______

SECTION A. MINISTER OF HEALTH, MINISTER REPRESENTATIVE, OR GENERAL SECRETARY

Objectives:

- To understand the vaccine intervention characteristics during country-specific change points in DTP1/DTP3 coverage
- To identify key internal and external actors, stakeholders, and partnerships in the immunization program both now and in the past
- To explore decision-making process in selecting the strategies and activities in the immunization program that created change points in DTP1/DTP3 coverage
- To identify the strategies behind current immunization programs to inform current context
- To identify previous immunization activities that were key during change points in the DTP1/DTP3 curve
- To understand key barriers and facilitators of interventions in the immunization sector

PART A1 – POLICY

1. What is the current state of legislation regarding vaccines and immunizations (laws, constitutional requirements)?

~ External Policies and Incentives; Outer Setting ~
~ RQ2. Political Will ~

Follow up:

- *Where would I find copies/documentation of that legislation?*
- ***If there is no knowledge of current legislation:*** *Is there past legislation that is being followed?*

2. How was legislation on vaccines and immunization developed?

~ External Policies and Incentives; Outer Setting ~
~ RQ2. Political Will ~

NOTE: BASE QUESTION ON THE PREVIOUS ANSWER.

Follow up:

- *When was key legislation passed?*
- *Who played a role in this (organization or person)?*

3. What national policies do you think were important in increasing vaccine coverage?

~ External Policies and Incentives; Outer Setting ~
~ RQ2. Political Will ~
~ RQ7. Vaccine Policy and Policy Enforcement ~

Follow up:

- *Which do you think has been the most important piece of legislation in contributing to the current rates of immunization coverage?*
- *What rule/regulation did you find to be most important/effective in increasing vaccine coverage?*
- *What role does the Ministry of Finance have in determining these rules and regulations?*
- *What role does the Ministry of Finance have in implementing the rules and regulations?*
- *Which supporting documents could you share with me?*

PART A2 – STAKEHOLDERS

4. How have financial investment decisions regarding the immunization sector been made?

~ Outer Setting ~
~ RQ1b. Immunization System Context Within the Health Sector ~

For peer review only - http://bmjopen.bmj.com/site/about/guidelines.xhtml

Date: ___________________________ *Interview Code: ______ - ______ - ______*

Follow up:<br>• *How is this relative to other areas of the healthcare system/sector?*<br>• *Who determines this?*<br>• *What percentage/amount of funding is spent on vaccinations as opposed to other health sector areas, including disease surveillance, coverage measurement, accountability?*<br>• ***(NOTE: Some probes may have been addressed in Part 2. Make sure to follow up on relevant information but don't repeat questions)***
**PART A3 – IMMUNIZATION PROGRAM**
5. What were key activities in planning changes for the immunization program? ***(NOTE: Refer to answers in section 2).***<br>***~ Planning; Process ~***<br>*~ RQ5. Knowledge and Resource Sharing ~*<br>*~ RQ6. Workforce ~*
Follow up:<br>• *Meeting, working group*<br>• *Did you have an action plan?*<br>• *A theory of change or log frame?*
6. What challenges did you face in the early planning phases?<br>***~ Planning; Process ~***
Follow up:<br>• *How were these challenges overcome* ***(EX: Internal to agency/external to agency)****?*
7. What kind of evidence or information **(EX: outbreaks, surveillance data)** informed changes in programming or interventions?<br>***~ Planning; Process ~***<br>*~ RQ5. Knowledge and Resource Sharing ~*
Follow up:<br>• *You mentioned ________ (DATA SOURCE NAME(S)). What role did this information have in the push to increase vaccine coverage?*<br>• *Can you explain how ________ (DATA SOURCE NAME(S)) is collected?*<br>• *How is this ________ (DATA SOURCE NAME(S)) integrated into other health systems?*<br>• *[If surveillance system not mentioned] Does ________ (COUNTRY) have a surveillance system?*<br>***~ Planning; Process~***<br>*~ RQ5. Knowledge and Resource Sharing~*
8. What areas of the country have seen the greatest improvement in vaccine rates?<br>***~ Reflecting and Evaluating; Process ~***<br>*~ RQ3. Information Systems ~*<br>*~ RQ4. Intent/Demand for Vaccines ~*

For peer review only - http://bmjopen.bmj.com/site/about/guidelines.xhtml

Date: ___________________________ *Interview Code: ______ - ______ - ______*

Follow up:<br><br>• *Why do you think these areas had the greatest improvement?*<br>○ *Was a project/intervention specifically tailored to this location?*<br>○ *Was it targeted as a critical area for change?*<br>• *What areas saw the quickest improvement?*<br>○ *Was a project/intervention specifically tailored to this location?*<br>○ *Was it targeted as a critical area for change?*
9. What areas of the country did not see significant changes?<br><br>***~ Reflecting and Evaluating; Process ~***<br>*~ RQ3. Information Systems ~*<br>*~ RQ4. Intent/Demand for Vaccines ~*
Follow up:<br><br>• *Why do you think this is?* (**NOTE: May have already had high coverage, may not relate to challenges)**<br>• *What were the challenges?*<br>○ *Why?*<br>○ *Was a project/intervention specifically tailored to this location?*<br>▪ *Implementation failure*<br>▪ *Theory failure*<br>○ *Was it targeted as a critical area for change?*<br>• *What has been/might be done to mitigate this?*
10. What are the greatest barriers to sustained performance?<br><br>***~ Reflecting and Evaluating; Process~***
11. What are the perceptions of corruption in the public sector?
Follow up:<br><br>• *Are you aware of any anti-corruption measures that have been put in places?*<br>• *What kind of accountability measures are in place that would prevent corruption?*<br>• *Perceptions of quality of electoral processes?*<br>• *Judicial institutions?*<br>• *Anti-corruption efforts?*

For peer review only - http://bmjopen.bmj.com/site/about/guidelines.xhtml

Date: ____________________________ *Interview Code:* ______ - ______ - ______

SECTION B. MINISTRY DEPARTMENTS OF EPIDEMIOLOGY, SURVEILLANCE, EPI, VACCINES, FAMILY HEALTH, GAVI REPRESENTATIVE

Objectives:

- To understand the vaccine intervention characteristics during country-specific change points in DTP1/DTP3 coverage
- To identify key internal and external actors, stakeholders, and partnerships in the immunization program both now and in the past
- To explore decision-making process in selecting the strategies and activities in the immunization program that created change points in DTP1/DTP3 coverage
- To identify the strategies behind current immunization programs to inform current context
- To identify previous immunization activities that were key during change points in the DTP1/DTP3 curve
- To understand key barriers and facilitators of interventions in the immunization sector

PART B1 – POLICY

1. What is the current state of legislation regarding vaccines and immunization?

~ External Policies; Outer Setting ~
~ RQ2. Political Will ~

NOTE: EXAMPLES INCLUDE LAWS, CONSTITUTIONAL REQUIRMENTS

Follow up:

- *Where would I find copies/documentation of that legislation?*

2. How was legislation on vaccines and immunization developed? What year was it started?

~ External Policies; Outer Setting ~
~ RQ2. Political Will ~

Follow up:

- *When was key legislation passed?*
- *Which do you think has been the most important piece of legislation in contributing to the current rates of DTP3 coverage?*
- *Where would I find copies/documentation of that legislation?*

3. What policies were made at the national level that you think were important in increasing vaccine coverage during ______ (YEAR(S))?

~ External Policies; Outer Setting ~
~ RQ2. Political Will ~
~ RQ7. Vaccine Policy and Policy Enforcement ~

Follow up:

- *How were these policies informed?*
- *What was the reason/rationale for creating these new policies?*
- *What is the policy making process, and how are new policies or recommendations disseminated and implemented once approved?*
- *How do they work in both vertical (e.g. across ministries) and horizontal (e.g. within different levels of a particular ministry)?*
- *What led to the selection of these policies?*

For peer review only - http://bmjopen.bmj.com/site/about/guidelines.xhtml

Date: ___________________________ *Interview Code: ______ - ______ - ______*

• *What challenges did you encounter with any new policies made?*
4. Are there relevant polices at the subnational/regional level? If so, what?<br><br>***~ External Policies; Outer Setting ~***<br>*~ RQ2. Political Will ~*<br>*~ RQ7. Vaccine Policy and Policy Enforcement ~*
Follow up:<br><br>• *If there are no policies at the subnational/regional level, why?*<br>○ *Do all regions need to follow the national law, or can they create their own?*<br>• *How were these policies informed?*<br>• *What led to the selection of these policies?*<br>• *What data were most important in setting policy?*<br>• *What kind of global/regional resources were utilized?*<br>• *How were policies implemented?*<br>•
**PART B2 – STAKEHOLDERS**
5. How have decisions on types of financial investments in the immunization sector been made, relative to other areas of the healthcare system/sector?<br><br>***~ Outer Setting ~***<br>*~ RQ1b. Immunization System Context Within the Health Sector ~*
Follow up:<br><br>• *Who determines this?*<br>• *What percentage/amount of funding is spent on vaccinations as opposed to other health sector areas, including disease surveillance, coverage measurement, accountability?*
**PART B3 – IMMUNIZATION PROGRAM**
6. What sort of intervention(s) do you attribute to the increase in immunization coverage?<br>***~ Planning; Process ~***<br>*~ RQ4. Intent/Demand for Vaccines ~*<br>*~ RQ5. Knowledge and Resource Sharing ~*<br>*~ RQ6. Workforce ~*
Follow up:<br><br>• *Prior to the intervention, what kind of piloting or testing of the intervention occurred?*<br>• *Did the service delivery of vaccinations change after the intervention? How?*<br>• *Where did this take place nationally?*<br>• *What was learned in this process?*

For peer review only - http://bmjopen.bmj.com/site/about/guidelines.xhtml

Date: ___________________________ *Interview Code:* ______ - ______ - ______

7. What were key activities in planning changes for the program?<br><br>***~ Planning; Process ~***<br>*~ RQ5. Knowledge and Resource Sharing ~*<br>*~ RQ6. Workforce ~*
Follow up:<br><br>• *What was your role?*<br>•<br>• *Did you have an action plan?*<br>• *A theory of change or log frame?*
8. What challenges did you face in the early planning phases?<br>***~ Planning; Process ~***
Follow up:<br><br>• *How were these challenges overcome* ***(EX: Internal to agency/external to agency)****?*
9. What were key training activities of [intervention/activities mentioned in Q6]?<br><br>***~ Planning & Executing; Process ~***<br>*~ RQ5. Knowledge and Resource Sharing ~*<br>*~ RQ6. Workforce ~*
Follow up:<br>*Do you have the following information, and can we access it?*<br>• *Who was involved in trainings* ***(NOTE: at all levels)****?*<br>• *Who taught the training?*<br>• *How were they selected*<br>• *Trainers*<br>• *Trainees?*<br>• *What was their motivation/incentive for involvement?*<br>• *How were the trainings conducted?*<br>• *Were individuals participating in the training given opportunities to provide feedback?*<br>• *If so, how?*<br>• *If not, why?*<br>• *If so, did this change the implementation of the program in the region*?
10. If no training activities, what modality did the intervention have?
Follow up:<br><br>• *What modalities were used for different aspects of the project, generally?*<br>***(NOTE: FOLLOW UP IF THE INTERVENTION SEEMS UNCLEAR, E.G. IS MORE FUNDING AVAILABLE? LOW TURNOVER OF STAFF? ETC.)***
11. What kind of evidence/information (***EX: outbreaks, surveillance data***) did you use to inform changes/interventions?<br>***~ Planning; Process ~***<br>*~ RQ5. Knowledge and Resource Sharing ~*

For peer review only - http://bmjopen.bmj.com/site/about/guidelines.xhtml

Date: ___________________________ *Interview Code: ______ - ______ - ______*

Follow up:<br>• *You mentioned ______ (DATA SOURCE NAME(S)). What role did this information have in the push to increase vaccine coverage?*<br>• *Can you explain how ______ (DATA SOURCE NAME(S)) is collected?*<br>• *How is this ______ (DATA SOURCE NAME(S)) integrated into other health systems?*<br>• *[IF SURVEILLANCE SYSTEM NOT MENTIONED] How does the surveillance system in ______ (COUNTRY) work?*<br>***~ Planning; Process ~***<br>*~ RQ5. Knowledge and Resource Sharing~*
12. What measures did you find critical for success when implementing interventions?<br>***~ Reflecting and Evaluating; Process ~***<br>*~ Q6. Workforce ~*
Follow up:<br>• *What advice would you give to another person/region/country who was going to implement a similar program/project to yours?*
13. What challenges/difficulties did you encounter when implementing interventions?<br>***~ Reflecting and Evaluating; Process ~***
Follow up:<br>• *How were these challenges overcome?*
14. What were the greatest challenges in project implementation?<br>***~ Reflecting and Evaluating; Process ~***
Follow up:<br>• *Of those challenges you mentioned, which was the greatest?*<br>• *Was the project specifically tailored to this location?*<br>• *Was it targeted as a critical area for change?*<br>• *How were decisions made about what to do?*<br>• *Who contributed to this decision-making? Why?*
15. What areas of the country have seen the greatest improvement in vaccine coverage that you would attribute to this intervention?<br>***~ Evaluation; Process~***<br>*~ RQ3. Information Systems ~*<br>*~ RQ4. Intent/Demand for Vaccines ~*
Follow up:<br>• *Was the project specifically tailored to this location?*<br>• *Was it targeted as a critical area for change?*<br>• *What areas saw the quickest improvement?*<br>• *Why do you think these areas had the greatest improvement?*
16. During the improvement period, did you recommend any new vaccine introductions ***(EX: PCV, rotavirus)***?<br>***~ Planning; Process ~***

For peer review only

For peer review only - http://bmjopen.bmj.com/site/about/guidelines.xhtml

Date: ___________________________ *Interview Code: ______ - ______ - ______*

~ RQ4. Intent/Demand for Vaccines ~
~ RQ5. Knowledge and Resource Sharing ~
~ RQ6. Workforce ~

Follow up:

- *How did they affect the overall delivery system?*

-

17. What areas did not experience significant change in coverage after implementation of the intervention?

~ Evaluation; Process~
~ RQ3. Information Systems ~
~ RQ4. Intent/Demand for Vaccines ~

Follow up:

- *Why do you think this is? (**EX: May have already had high coverage, may not relate to challenges)***
- *What were the challenges? Why?*
- *What has been/might be done to mitigate this?*

18. What populations are currently the most vulnerable to low coverage?

~ Evaluation; Process~

19. What populations historically were the most vulnerable to low coverage?

~ Evaluation; Process~

Follow up:

- *How did you identify these populations as vulnerable?*
  - *Both previously and now*
- *How did you reach out to these populations?*
- *What strategy/strategies do you use to reduce inequities?*
- *What strategy did you previously use to reduce inequities?*
- *What kind of data supports this?*
- *Is this data you can share with me?*

For peer review only - http://bmjopen.bmj.com/site/about/guidelines.xhtml
For peer review only

Date: ___________________________ *Interview Code:* ______ - ______ - ______

•

20. What interventions were offered through community health workers (CHWs)?

~ Executing; Process ~
~ RQ6. Workforce ~

Follow up:

- *How were/are CHWs motivated/incentivized for involvement?*
  - *Financial*
  - *Respect*
  - *Opportunities for learning/capacity building*
  - *Recruited by trusted person*
    - *Unable to say*
- *What kind of trainings are offered to:*
  - *Increase learning?*
  - *Build capacity*
- *What are the qualifications to be a CHW?*
- *Are their responsibilities increased over time?*
- *How do they prioritize responsibilities?*
- *How do CHWs become better at what they do over time?*

21. What interventions were offered at various levels of the health system? To providers?

~ Execution; Process ~
~ RQ3. Information Systems ~
~ RQ6. Workforce ~

Follow up:

- *Province/district/health post*
- *How were these developed?*
- *How were they implemented?*
  - *Training?*
- *Why were they implemented in the way that they were?*
- *Who oversaw these interventions?*
- *What led to the selection of these interventions?*
- *Was there variation in different geographic regions etc. If so, why?*

•

•

22. What programs were offered to parents or first-time parents?

~ Intervention Characteristics~
~Outer Setting ~
~ RQ4. Intent/Demand for Vaccines ~

Follow up:

- *Were these tailored to different areas/cultures/religions/languages/etc.?*

For peer review only - http://bmjopen.bmj.com/site/about/guidelines.xhtml

Date: ____________________________ *Interview Code: ______ - ______ - ______*

- *How were these targeted specifically to mothers, fathers or alternative caretakers?*
- *How were they developed?*
- *Where did you get the idea for these interventions?*
  - *Did you use a framework or intervention to inform your intervention?*
- *How were they delivered?*
- *Who was engaged in delivery?*
- *What determined their involvement?*
- *What kinds of modalities were used?*
- *What kind of communication materials (if any) were utilized?*
  - *Mass media?*
  - *Dissemination through health centers?*
  - *Other?*
- *How did you get individuals to participate in this program?*
- *What led to their selection?*
- *Could you share any materials with us?*

**IF PARTICIPANT IS UNABLE TO ANSWER, NOT NECESSARY TO GO THROUGH EACH PROBE.*

~ Beneficiary needs and resources ~

23. Were the programs used in combination with other health interventions?

~ Outer Setting ~
~ RQ4. Intent/Demand for Vaccines ~

Follow up:

- *Were there any communication materials, strategies, or campaigns in conjunction with the other healthcare interventions?*
- *Or communications (IEC materials, mass media, etc.)?*
- *How was this coordinated?*
- *Who took ultimate responsibility for outcomes?*

24. Did you make any changes to the supply chain in ________________________________
$\mathit{YEAR(S)\ or\ during\ specific\ interventions\ mentioned}$

~ Execution; Process ~
~ Q6. Workforce ~

Follow up:

- *How were these changes made?*
- *Why was this change made?*
  - *Has this change had the anticipated impact (on coverage or otherwise)?*

25. Were there any changes made to the data systems in ____________________________ ?
$\mathit{YEAR(S) or\ specific\ intervetions\ mentioned}$

~ Execution; Process ~
~ Q3. Information Systems ~
~ Q6. Workforce ~

For peer review only - http://bmjopen.bmj.com/site/about/guidelines.xhtml

Date: ___________________________ *Interview Code: ______ - ______ - ______*

Follow up:

- *How were these changes made?*
- *Why was this change made?*
    - *Has this change had the anticipated impact (on coverage or otherwise)?*
- *Did you develop or already have in place a clinic-based immunization record keeping system to track immunizations administered to individuals?*
- *Who kept these records? Did parents hold records?*
- *What are parents' involvement in record keeping for their child's vaccinations?*
- *To what extent was technology a component of record keeping (computer, phone data collection)?*

26. During those years, did you have any kind of reminder/recall system for children's vaccinations?

~ Execution; Process~
~ Q3. Information Systems ~
~ Q6. Workforce ~

NOTE: REMINDER SYSTEM DEFINED AS A MESSAGE SENT BEFORE APPOINTMENTS TO REMIND PARENTS TO COME; RECALL SYSTEM DEFINED AS A NOTICE SENT TO PARENTS WHO FAIL TO KEEP THEIR APPOINTMENTS.

Follow up:

- *If so, how was this implemented?*
- *What kind of system was this?*
    - *Cell phone text reminders?*
    - *Community health worker in-person reminders?*
    - *Church/religious institution reminders?*
- *Was technology a component of this system?*
- *Who carries out the reminders or recall?*
- *Were there interventions offered through the CHWs?*

-

-

-

For peer review only - http://bmjopen.bmj.com/site/about/guidelines.xhtml

Date: ___________________________ *Interview Code: ______ - ______ - ______*

-

-

27. How do you monitor the performance of immunization programs?

~ Evaluation; Process~
~ RQ3. Information Systems ~

28. How does this differ from how immunization programs were monitored in the past?

~ Evaluation; Process ~
~ RQ3. Information Systems ~

Follow up:

- *Who is responsible for collecting data related to measures?*
- *How is data used?*
- *What measures are included?*
- *How often is data collected/shared?*
- *How is this information communicated on a local/regional/national level?*
- *How do you perceive the quality of data collected?*
- *If data quality is poor, is it/how is it used? If poor, what would improve the quality?*
- *Are there "independent" checks of the reported data to ensure they are valid?*
- *Would any other types of data have been helpful in managing the program?*
- *Is there an independent group that evaluates the entire program periodically? If so, please give some details regarding the composition of the group, how it is chosen, and what activities they undertake to evaluate the program?*

29. Is there any recognition for being at the top levels of coverage in the country or for making major improvements? If so, can you give some examples of recognition/incentives?

Follow up:

- *If yes, have you noticed any issues of misreporting due to the possibility of recognition/incentives?*

30. How do you measure the impact of immunization programs?

~ Evaluation; Process ~
~ RQ3. Information Systems ~

For peer review only - http://bmjopen.bmj.com/site/about/guidelines.xhtml

Date: ____________________________ *Interview Code:* ______ - ______ - ______

31. Is there public opinion data on vaccination?<br><br>***~ Evaluation; Process ~***<br>*~ RQ3. Information Systems ~*
Follow up:<br>• *If yes, how can we access this data?*
32. What factors have maintained the sustainability of the immunization program?<br><br>***~ Evaluation; Process ~***
a. **Partnerships:** Does the program have diverse organizations invested in its success?<br>b. **Partnerships**: What does the collaboration between these diverse organizations look like?<br>c. **Organizational capacity**: Were there organizational systems in place to support the program needs?<br>d. **Champion**: Does the program have strong champions? ***(EX: Political or advocacy support outside of the program?)***<br>e. **Funding**: Does the program have sustained funding?<br>i. *Follow up: How important was support of the legislature to program success **(EX. appropriating additional funding not initially requesting by the executive branch**)?*<br>f. **Evaluation**: How do evaluation results inform future program planning, implementation, and funding?<br>i. *Follow up: What kind of data drives this?*<br>ii. *Can you share any data with us?*
g. **Adaptation**: How does the immunization program adapt to new evidence?<br><br>***~ Evaluation; Process~***<br>*~RQ1. Why and How? ~*<br>*~ RQ 3. Information Systems ~*
Follow up:<br>• *Where do you learn about new evidence?*
h. **Adaptation**: How does the immunization program adapt to new information?
i. **Communication**: How does the immunization program secure community support or awareness of the issue of childhood vaccines?<br><br>***~ Evaluation; Process~***<br>*~ RQ3. Information Systems ~*<br>*~ RQ4. Intent/Demand for Vaccines ~*<br>*~ RQ5. Knowledge and Resource Sharing ~*<br>*~ RQ6. Workforce ~*
Follow up:<br>• *How does the immunization program maintain community support of childhood vaccines?*<br>• *How does the immunization program maintain awareness of childhood vaccines?*

For peer review only - http://bmjopen.bmj.com/site/about/guidelines.xhtml

Date: ___________________________ *Interview Code: ______ - ______ - ______*

• *To what extent is the immunization program resilient from rumors and misinformation regarding immunizations?*<br>• *How could resilience be improved?*
j. **Media**: How is vaccination portrayed and discussed by the media?<br><br>***~ Outer Setting ~***<br>*~ RQ3. Information Systems ~*<br>*~ RQ4. Intent/Demand for Vaccines ~*<br>*~ RQ5. Knowledge and Resource Sharing ~*
Follow up:<br>• *What role did the media play in improved coverage?*<br>• *How did the media become involved and how was that involvement sustained?*
33. What kind of planning is there for future resource needs?<br><br>***~ Evaluation; Process~***<br>*~ RQ5. Knowledge and Resource Sharing ~*
34. What are the greatest barriers to sustained coverage?<br><br>***~ Reflection; Process~***
35. What are the perceptions of corruption in the public sector?<br><br>***~ Inner Setting ~***<br>*~ RQ2. Political Will ~*
Follow up:<br>• *Perceptions of quality of electoral processes?*<br>• *Judicial institutions?*<br>• *Anti-corruption efforts?*

For peer review only

For peer review only - http://bmjopen.bmj.com/site/about/guidelines.xhtml

Date: ____________________________ *Interview Code:* ______ - ______ - ______

SECTION C. MINISTRY DEPARTMENTS (OR LIAISONS) OF PLANNING, STATISTICS, RESEARCH, HUMAN RESOURCES, COMMUNICATION AND DEVELOPMENT

Objectives:

- To understand the vaccine intervention characteristics during country-specific change points in DTP1/DTP3 coverage
- To identify key internal and external actors, stakeholders, and partnerships in the immunization program both now and in the past
- To explore decision-making process in selecting the strategies and activities in the immunization program that created change points in DTP1/DTP3 coverage
- To identify the strategies behind current immunization programs to inform current context
- To identify previous immunization activities that were key during change points in the DTP1/DTP3 curve
- To understand key barriers and facilitators of interventions in the immunization sector

PART C1 – IMMUNIZATION PROGRAM

1. What were key activities in planning changes for the immunization program?

~ Planning; Process ~
~ RQ5. Knowledge and Resource Sharing ~
~ RQ6. Workforce ~

NOTE: REFER TO ANSWERS GIVEN IN SECTION 2.
IF NOT INVOLVED, SKIP TO Q4.

Follow up:

- *Did you have an action plan?*
- *A theory of change or log frame?*

2. What challenges did you face in the early planning phases?

~ Planning; Process ~

Follow up:

- *How were these challenges overcome* ***(EX. Internal to agency/external to agency)****?*

3. What kind of evidence/information **(EX: outbreaks, surveillance data)** did you use to determine changes/interventions?

~ Planning; Process~
~ RQ5. Knowledge and Resource Sharing ~

Follow up:

- *You mentioned* ______________ (DATA SOURCE NAME(S)). *What role did this information have in the push to increase vaccine coverage?*
- *Can you explain how* ______________ (DATA SOURCE NAME(S)) *is collected?*
- *How is this* ______________ (DATA SOURCE NAME(S)) *integrated into other systems?*
- *[If surveillance system not mentioned] How does the surveillance system in* ______________ (COUNTRY) *work?*

4. Prior to the critical changes/intervention, what kind of piloting/testing of the intervention occurred?

~ Planning; Process~
~ RQ4. Intent/Demand for Vaccines ~
~ RQ5. Knowledge and Resource Sharing ~

For peer review only - http://bmjopen.bmj.com/site/about/guidelines.xhtml

Date: ____________________________ *Interview Code:* ______ - ______ - ______

~ RQ6. Workforce ~
Follow up:<br>• *Did the service delivery of vaccinations change?*<br>• *Where did this take place?*<br>• *What was learned in this process?*
5. What were key training or technical assistance activities of the intervention?<br>***~ Planning, Executing; Process ~***<br>*~ RQ5. Knowledge and Resource Sharing ~*<br>*~ RQ6. Workforce ~*
Follow up:<br>• *Who was involved in trainings* ***(NOTE: at all levels)****?*<br>• *Who taught the training?*<br>• *How were they selected (trainers and trainees)?*<br>• *What was their motivation/incentive for involvement?*<br>• *How were the trainings conducted?*
6. If no training activities, what modality did the intervention have?
Follow up:<br>• *What modalities were used for different aspects of the project, generally?*
7. Did you make any changes to the supply chain in $\overline{YEAR(S)}$? If so, tell us how.<br>***~ Execution; Process~***<br>*~ Q6. Workforce ~*
Follow up:<br>• *What worked well?*<br>• *What were the challenges?*
8. Did you make any changes to data systems or their use in $\overline{YEAR(S)}$? If so, what kind of changes were made and how?<br>***~ Execution; Process~***<br>*~ Q3. Information Systems ~*<br>*~ Q6. Workforce ~*
9. How do you monitor the performance of immunization programs?<br>***~ Evaluation; Process~***<br>*~ RQ3. Information Systems ~*
10. How does this differ from how immunization programs were monitored in the past?<br>***~ Evaluation; Process~***<br>*~ RQ3. Information Systems ~*

For peer review only - http://bmjopen.bmj.com/site/about/guidelines.xhtml

Date: ___________________________ *Interview Code:* ______ - ______ - ______

Follow up:

- *Who is responsible for collecting data related to measures?*
- *How is data used?*
- *What measures are included?*
- *How often is data collected/shared?*
- *How is this information communicated on a local/regional/national level?*
- *How do you perceive the quality of data collected?*
- *If data quality is poor, is it/how is it used? If poor, what would improve the quality?*
- *Are there "independent" checks of the reported data to ensure they are valid?*
- *Would any other types of data have been helpful in managing the program?*
- *Is there an independent group that evaluates the entire program periodically? If so, please give some details regarding the composition of the group, how it is chosen, and what activities they undertake to evaluate the program?*

11. Is there any recognition for being at the top levels of coverage in the country or for making major improvements? If so, can you give some examples of recognition/incentives?

Follow up:

- *If yes, have you noticed any issues of misreporting due to the possibility of recognition/incentives?*

12. What changes have occurred in programming since $\overline{YEAR(S)}$?

13. What factors have helped to ensure the sustainability of the immunization program?

~ Evaluation; Process~

a. **Partnerships:** Does the program have diverse organizations invested in its success? *What does the collaboration between these diverse organizations look like?*

b. **Organizational capacity:** Were there organizational systems in place to support the program needs?

c. **Evaluation:** How do evaluation results inform future program planning, implementation, and funding?

d. **Communication:** How does the immunization program secure or maintain community support of childhood vaccines?
How does the immunization program secure or maintain awareness of childhood vaccines?
How do you define awareness?
To what extent is the immunization program resilient from rumors and misinformation regarding immunizations?
How could resilience be improved?

e. **Funding**: Does the program have sustained funding?
*How important was support of the legislature to program success (**EX. appropriating additional funding not initially requesting by the executive branch**)?*

f. **Adaptation**: How does the immunization program adapt to new evidence?

For peer review only - http://bmjopen.bmj.com/site/about/guidelines.xhtml

Date: ___________________________ *Interview Code:* ______ - ______ - ______

Where do you learn about new evidence?<br><br>g. **Adaptation**: How does the immunization program adapt to new information?<br><br>h. **Media:** How is vaccination portrayed and discussed by the media?<br>*What role did the media play in improved coverage?*<br>*How did the media become involved?*<br>*How was that involvement sustained?*
i. **Strategic planning:** What kind of planning is there for future resource needs?<br><br>***~ Evaluation; Process~***<br>*~ RQ5. Knowledge and Resource Sharing ~*
14. What are the perceptions of corruption in the public sector?<br><br>***~ Inner Setting ~***<br>*~ RQ2. Political Will ~*
Follow up:<br>• *Perceptions of quality of electoral processes?*<br>• *Judicial institutions?*<br>• *Anti-corruption efforts?*

For peer review only

Date: ____________________________ *Interview Code:* ______ - ______ - ______

SECTION D. MINISTRY DEPARTMENTS (OR LIAISONS) OF INFRASTRUCTURE, EQUIPMENT, FINANCE

Objectives:

- To understand the vaccine intervention characteristics during country-specific change points in DTP1/DTP3 coverage
- To identify key internal and external actors, stakeholders, and partnerships in the immunization program both now and in the past
- To explore decision-making process in selecting the strategies and activities in the immunization program that created change points in DTP1/DTP3 coverage
- To identify the strategies behind current immunization programs to inform current context
- To identify previous immunization activities that were key during change points in the DTP1/DTP3 curve
- To understand key barriers and facilitators of interventions in the immunization sector
- To understand current and historical investments in the immunization sector

PART D1 – POLICY

1. What are the current regulations related to expenditures in health services?

~ External Policies; Outer Setting ~
~ RQ2. Political Will ~

Follow up:

- *What are the current regulations related to vaccination in particular?*

2. How have these regulations changed since 2000?

~ External Policies; Outer Setting ~
~ RQ2. Political Will ~

Follow up:

- *What regulation did you find to be most important/effective in increasing vaccine coverage?*
- *Which supporting documents could you share with me?*
- *What role does the Ministry of Finance have in determining these rules and regulations?*
- *What role does the Ministry of Finance have in implementing the rules and regulations?*
- *What barriers exist?*
- *How are these barriers addressed?*
- *How does this differ from the past?*

3. How have decisions on types of investments in the immunization sector been made?

~ Outer Setting ~
~ RQ1b. Immunization System Context Within the Health Sector ~

Follow up:

- *How are these decisions relative to other investments in other healthcare sectors?*
- *Who determines this?*
- *What percentage/amount of funding is spent on vaccinations as opposed to other health sector areas, including disease surveillance, coverage measurement, accountability?*

PART D2 – IMMUNIZATION PROGRAM

4. Did you make any changes to the supply chain in $\overline{\hspace{2cm}}_{YEAR(S)}$? If so, tell us how.

~ Execution; Process~
~ Q6. Workforce ~

For peer review only - http://bmjopen.bmj.com/site/about/guidelines.xhtml

Date: ___________________________ *Interview Code:* ______ - ______ - ______

5. What changes have occurred in immunization programming since $\frac{}{YEAR(S)}$?<br>***~ Evaluation; Process ~***
6. What factors have maintained the sustainability of the immunization program?<br>***~ Evaluation; Process~***
a. **Funding**: Does the program have sustained funding?<br>***~ Evaluation; Process~***<br>*~ RQ7. Vaccine Policy and Policy Enforcement ~*
b. **Adaptation**: How does the immunization program adapt to new evidence?<br>***~ Evaluation; Process~***<br>*~RQ1. Why and How? ~*<br>*~ RQ 3. Information Systems ~*
Probes:<br>• *Where do you learn about new evidence?*
c. **Adaptation**: How does the immunization program adapt to new information?
Probes:<br>• *Where do you learn about new information?*
d. **Evaluation**: How do evaluation results inform future program planning, and implementation, and funding?<br>***~ Evaluation; Process~***<br>*~ RQ3. Information Systems ~*
Follow up:<br>• *What kind of data drives this?*<br>• *Can you share any data with us?*
j. What are the perceptions of corruption in the public sector?<br>***~ Inner Setting ~***<br>*~ RQ2. Political Will ~*
Follow up:<br>• *Perceptions of quality of electoral processes?*<br>• *Judicial institutions?*<br>• *Anti-corruption efforts?*

For peer review only

For peer review only - http://bmjopen.bmj.com/site/about/guidelines.xhtml

Date: ___________________________ *Interview Code:* ______ - ______ - ______

SECTION E. PARTNER ORGANIZATIONS, SUCH AS WHO, UNICEF, CDC, USAID, ETC; NITAG, PROFESSIONAL ORGANIZATION MEMBERS

Objectives:

- To understand the vaccine intervention characteristics during country-specific change points in DTP1/DTP3 coverage
- To identify key internal and external actors, stakeholders, and partnerships in the immunization program both now and in the past
- To explore decision-making process in selecting the strategies and activities in the immunization program that created change points in DTP1/DTP3 coverage
- To identify the strategies behind current immunization programs to inform current context
- To identify previous immunization activities that were key during change points in the DTP1/DTP3 curve
- To understand key barriers and facilitators of interventions in the immunization sector

PART E1 – STAKEHOLDERS

1. What determines opportunities for career advancement for stakeholders involved in vaccination efforts?

~ Relative Priority, Incentives and Rewards; Process ~
~ RQ2. Political Will ~

Follow up:

- *Is there a way of systematically knowing partisan affiliations or who appointed them? Specifically, those involved in agencies relevant to vaccination.*

PART E2 – POLICY

2. What national policies do you think were important in increasing vaccination coverage during ______________?
   YEAR(S)

~ External Policies; Outer Setting~
~ RQ2. Political Will ~
~ RQ7. Vaccine Policy and Policy Enforcement ~

Follow up:

- *How were these policies informed?*
- *What was the reason/rationale for creating these new policies?*
- *What is the policy making process, and how are new policies or recommendations disseminated and implemented once approved?*
- *How do they work in both vertical* ***(EX. across ministries)*** *and horizontal* ***(EX. within different levels of a particular ministry)****?*
- *What led to the selection of these policies?*

PART E3 – IMMUNIZATION PROGRAM

3. What areas of the country have seen the greatest improvement in vaccine rates?

~ Evaluation; Process ~

For peer review only - http://bmjopen.bmj.com/site/about/guidelines.xhtml

Date: ___________________________ *Interview Code:* ______ - ______ - ______

~ RQ3. Information Systems ~<br>*~ RQ4. Intent/Demand for Vaccines ~*
Follow up:<br>• *Was the project specifically tailored to this location?*<br>• *Was it targeted as a critical area for change?*<br>• *What areas saw the quickest improvement?*<br>• *Why do you think these areas had the greatest improvement?*
4. What areas of the country did not see significant changes?<br>***~ Evaluation; Process~***<br>*~ RQ3. Information Systems ~*<br>*~ RQ4. Intent/Demand for Vaccines ~*
Follow up:<br>• *Why do you think this is?* ***(EX: May have already had high coverage, may not relate to challenges)***<br>• *What were the challenges?*<br>○ *Why?*<br>• *What has been/might be done to mitigate this?*
5. What populations are currently the most vulnerable to low coverage?<br>***~ Evaluation; Process~***
6. What populations historically were the most vulnerable to low coverage?<br>***~ Evaluation; Process~***
Follow up:<br>• *How did you identify these populations as vulnerable?*<br>○ *Both previously and now*<br>• *How did you reach out to these populations?*<br>• *What strategy/strategies do you use to reduce inequities?*<br>• *What strategy did you previously use to reduce inequities?*<br>• *What kind of data supports this?*<br>• *Is this data you can share with me?*
7. How do you monitor the performance of immunization programs?
8. How does this differ from how immunization programs were monitored in the past?
Follow up:<br>• *Who is responsible for collecting data related to measures?*<br>• *How is data used?*<br>• *What measures are included?*<br>• *How often is data collected/shared?*<br>• *How is this information communicated on a local/regional/national level?*<br>• *How do you perceive the quality of data collected?*

For peer review only - http://bmjopen.bmj.com/site/about/guidelines.xhtml

Date: ____________________________ *Interview Code: ______ - ______ - ______*

• *If data quality is poor, is it/how is it used? If poor, what would improve the quality?*<br>• *Are there "independent" checks of the reported data to ensure they are valid?*<br>• *Would any other types of data have been helpful in managing the program?*<br>• *Is there an independent group that evaluates the entire program periodically? If so, please give some details regarding the composition of the group, how it is chosen, and what activities they undertake to evaluate the program?*
9. Is there any recognition for being at the top levels of coverage in the country or for making major improvements? If so, can you give some examples of recognition/incentives?
Follow up:<br>• *If yes, have you noticed any issues of misreporting due to the possibility of recognition/incentives?*
10. How do you measure the impact of immunization programs?
11. What factors have helped to ensure the sustainability of the immunization program?<br>***~ Evaluation; Process~***
a. **Partnerships:** Does the program have diverse organizations invested in its success?<br>b. **Partnerships**: What does the collaboration between these diverse organizations look like?<br>c. **Organizational capacity**: Were there organizational systems in place to support the program needs?<br>d. **Champion**: Does the program have strong champions? ***(EX: Political or advocacy support outside of the program?)***<br>e. **Funding**: Does the program have sustained funding?<br>ii. *Follow up: How important was support of the legislature to program success (**EX. appropriating additional funding not initially requesting by the executive branch**)?*<br>f. **Evaluation**: How do evaluation results inform future program planning, implementation, and funding?<br>iii. *Follow up: What kind of data drives this?*<br>iv. *Can you share any data with us?*
g. **Media**: How is vaccination portrayed and discussed by the media?<br>***~ Outer Setting ~***<br>*~ RQ3. Information Systems ~*<br>*~ RQ4. Intent/Demand for Vaccines ~*<br>*~ RQ5. Knowledge and Resource Sharing ~*
Follow up:<br>• *What role did the media play in improved coverage?*<br>• *How did the media become involved?*<br>• *How was that involvement sustained?*
h. **Adaptation**: How does the immunization program adapt to new evidence?<br>***~ Evaluation; Process~***<br>*~RQ1. Why and How? ~*<br>*~ RQ 3. Information Systems ~*

For peer review only - http://bmjopen.bmj.com/site/about/guidelines.xhtml

Date: ___________________________ *Interview Code:* ______ - ______ - ______

Follow up:<br>• *Where do you learn about new evidence?*
i. **Adaptation**: How does the immunization program adapt to new information?
j. **Communication**: How does the immunization program secure community support or awareness of the issue of childhood vaccines?<br>***~ Evaluation; Process~***<br>*~ RQ3. Information Systems ~*<br>*~ RQ4. Intent/Demand for Vaccines ~*<br>*~ RQ5. Knowledge and Resource Sharing ~*<br>*~ RQ6. Workforce ~*
Follow up:<br>• *How do you define awareness?*<br>• *How does the immunization program maintain community support of childhood vaccines?*<br>• *How does the immunization program maintain awareness of childhood vaccines?*<br>• *To what extent is the immunization program resilient from rumors and misinformation regarding immunizations?*<br>• *How could resilience be improved?*
12. **Strategic planning**: What kind of planning is there for future resource needs?<br>***~ Evaluation; Process~***<br>*~ RQ5. Knowledge and Resource Sharing ~*
13. What are the perceptions of corruption in the public sector?<br>***~ Inner Setting ~***<br>*~ RQ2. Political Will ~*
Follow up:<br>• *Perceptions of quality of electoral processes?*<br>• *Judicial institutions?*<br>• *Anti-corruption efforts?*

For peer review only

For peer review only - http://bmjopen.bmj.com/site/about/guidelines.xhtml

Date: ___________________________ *Interview Code:* ______ - ______ - ______

SECTION G. PROVINCIAL HEALTH DIRECTOR, SURVEILLANCE, PREVENTION, AND VACCINATION PERSONNEL

Objectives:

- To understand the vaccine intervention characteristics during country-specific change points in DTP1/DTP3 coverage
- To identify key internal and external actors, stakeholders, and partnerships in the immunization program both now and in the past
- To identify the strategies behind current immunization programs to inform current context
- To identify previous immunization activities that were key during change points in the DTP1/DTP3 curve
- To understand key barriers and facilitators of interventions in the immunization sector
- To understand health system characteristics, currently and in the past
- To identify and/or confirm project district selection

PART G1 – HEALTH SYSTEM

1. Previously, we poke about general success/increase in coverage at the national level. What are some successes in the districts?
   a.

Follow up:

- *Which specific districts would you classify as exemplar districts?*
- *Why?*
- *Which districts have sustained high coverage from 2011 – 2017?*
  - *Was the project specifically tailored to this location?*
  - *Was it targeted as a critical area for change?? Why?*
  - *What areas saw the quickest improvement?*
  - *Why do you think these areas had the greatest improvement?*
- *Which areas did not experience significant change?*
- *Which populations area most vulnerable to low coverage??*
  - *How did you identify these populations as vulnerable? Both previously and now?*
  - *How did you reach out to these populations?*
  - *What strategy/strategies do you use to reduce inequities?*
  - *What kind of data supports this?*
  - *Is this data you can share with me?*

2. Here are some districts we have selected based on heterogeneity within the province (with a focus on greater populations). What do you think about these selections?

Follow up:

- *Would you make an argument for a different selected/districts to be included? Why?*

3. How many health facilities are available per 1,000 people?

PART G2 – IMMUNIZATION PROGRAM

4. What is a <u>specific intervention</u> that contributed to the increase in vaccine coverage in your province?

Follow up:

For peer review only - http://bmjopen.bmj.com/site/about/guidelines.xhtml

Date: ____________________________ *Interview Code: ______ - ______ - ______*

• *Some people have mentioned specific interventions/stakeholders as being critical to the improved coverage, such as ____________*
5. What was your involvement in this intervention(s) and the changes that followed? (IF NO DIRECT INVOLVEMENT IN PLANNING ACTIVITIES, SKIP TO Q9)
Follow up:<br>• *Were these directives from the ministry?*<br>• *How did you decide which changes to make?*<br>• *How did this intervention affect people's roles and responsibilities at different levels in the health system?*<br>• *How were these changes received at different levels of the health system, including parents/end-users?*
6. What were key activities in <u>planning</u> changes for the program?<br>***~ Planning; Process ~***<br>*~ RQ5. Knowledge and Resource Sharing ~*<br>*~ RQ6. Workforce ~*
Follow up:<br>• *Did you have an action plan?*<br>• *A theory of change or log frame?*<br>• *What challenges did you face in the early <u>planning</u> phases?*<br>○ *How were these challenges overcome **(EX: Internal to agency/external to agency)**?*
7. Prior to the intervention, what kind of piloting or testing of the intervention occurred?<br>***~ Planning; Process~***<br>*~ RQ4. Intent/Demand for Vaccines ~*<br>*~ RQ5. Knowledge and Resource Sharing ~*<br>*~ RQ6. Workforce ~*
Follow up:<br>• *Did the service delivery of vaccinations change?*<br>• *Where did this take place?*<br>• *What was learned in this process?*
8. What were key training activities?<br>***~ Planning, Executing; Process ~***<br>*~ RQ5. Knowledge and Resource Sharing ~*<br>*~ RQ6. Workforce ~*
Follow up:<br>• *If no training activities, what modality did the intervention have?*<br>• *If training activities, do you have the following information, and can we access it?*<br>• *Who was involved in trainings **(NOTE: at all levels)**?*<br>• *Who taught the training?*<br>• *How were they selected*<br>• *Trainers*<br>• *Trainees?*<br>• *What was their motivation/incentive for involvement?*

For peer review only - http://bmjopen.bmj.com/site/about/guidelines.xhtml

Date: ___________________________ *Interview Code: ______ - ______ - ______*

• *How were the trainings conducted?*<br>• *What were the expectations of the participants in training?*
9. What kind of evidence did you use to determine [changes/interventions]?<br>***~ Planning; Process~***<br>*~ RQ5. Knowledge and Resource Sharing ~*
Follow up:<br>*(E.g., outbreaks, surveillance data).*<br>• *You mentioned* ______ (*DATA SOURCE NAME(S)*) *. What role did this information have in the push to increase vaccine coverage?*<br>• *Can you explain how* ______ (*DATA SOURCE NAME(S)*) *is collected?*<br>• *How is this* ______ (*DATA SOURCE NAME(S)*) *integrated into other systems?*<br>• *How does the surveillance system in* ______ (*COUNTRY*)*. work?*<br>***~ Planning; Process~***<br>*~ RQ5. Knowledge and Resource Sharing~*
10. What do you see as critical for implementation of the [intervention(s)] to succeed?<br>***~ Reflecting and Evaluating; Process ~***<br>*~ Q6. Workforce ~*
Follow up:<br>• *When people give commands from top down, what authority do you have to make changes?*<br>• *What advice would you give to another person/region/country who was going to implement a similar program/project to yours?What are you most proud of in terms of this work?*
11. What challenges/difficulties did you encounter when implementing interventions?<br>***~ Reflecting and Evaluating; Process~***
Follow up:<br>• *How were these challenges overcome?*<br>○ *What contributed to these challenges?*<br>○ *How were decisions made about what to do?*<br>○ *What challenges did you encounter with any new policies made?*<br>○ *Of these challenges, which challenge was the greatest in this province?*<br>○ *Was the project specifically tailored to this location?*<br>• *Who contributed to this decision-making? Why?*
12. What interventions were offered through community health workers?<br>***~ Execution; Process~***<br>*~ RQ6. Workforce ~*
Follow up:<br>• *Outreach activities? Training?*<br>• *Including any kind of reminder system for people to get vaccinations?*

For peer review only - http://bmjopen.bmj.com/site/about/guidelines.xhtml

Date: ___________________________ *Interview Code:* ______ - ______ - ______

• *What kind of system was this? (Cellphone texts, in-person reminders (by who?), church/religious institution reminders?)*<br>• *How was technology a component of this system?*<br>• *Did you develop or already have in place a clinic-based immunization record keeping system to track immunizations administered to individuals?*<br>• *Tools used?*<br>• *How were these developed?*<br>• *What led to the selection of these interventions?*<br>• *Was there variation in different geographic regions etc. If so, why?*<br>• *How were/are CHWs motivated/incentivized for involvement?*<br>• *What are the job requirements to be a CHW?*<br>• *How have responsibilities shifted over time? How do you think this has impacted their performance? Their motivation?*<br>• *What kind of current involvement does the Neighborhood Health Committee have in current CHW programming and responsibilities? What kind of role did they have in the past?*<br>• *What kinds of continued training/capacity building are provided to CHWs?*<br>***~ Beneficiary Needs and Resources; Outer Setting ~***
13. What interventions were offered at various levels of the health system? To providers?<br>***~ Execution; Process~***<br>*~ RQ3. Information Systems ~*<br>*~ RQ6. Workforce ~*
Follow up:<br>• *How were these developed?*<br>• *How were they implemented? Training?*<br>• *Why were they implemented in the way that they were?*<br>• *What led to the selection of these interventions?*<br>• *Was there variation in different geographic regions etc. If so, why?*
14. What programs were offered to parents or soon-to-be parents?<br>***~ Intervention Characteristics~***<br>***~ Outer Setting ~***<br>*~ RQ4. Intent/Demand for Vaccines ~*
Follow up:<br>• *Were these tailored to different areas/cultures/religions/languages/etc.?*<br>• *How were these targeted specifically to mothers, fathers or alternative caretakers?*<br>• *How were they developed?*<br>• *Where did you get the idea for these interventions?*<br>○ *Did you use some other framework or intervention as to inform your intervention?*<br>• *How were they delivered?*

For peer review only - http://bmjopen.bmj.com/site/about/guidelines.xhtml

Date: ___________________________ *Interview Code: ______ - ______ - ______*

• *Who was engaged in delivery?*<br>• *What determined their involvement?*<br>• *What kinds of modalities were used?*<br>• *What kind of communication materials (if any) were utilized? Mass media? Dissemination through health centers? Other?*<br>• *How did you get individuals to participate in this program?*<br>• *What led to their selection?*<br>• *Could you share any materials with us?*<br>*~ Beneficiary needs and resources ~*
15. Did you make any changes to the supply chain in $\overline{\quad YEAR(S) \quad}$?
Follow up:<br>• *What worked well?*<br>• *What were challenges?*
16. Are there relevant polices at the subnational/regional level? If so, what?<br>***~ External Policies; Outer Setting ~***<br>*~ RQ2. Political Will ~*<br>*~ RQ7. Vaccine Policy and Policy Enforcement ~*
Follow up:<br>• *If there no policies at the subnational/regional level, do all regions need to follow the national law, or can they create their own?*<br>• *How were these policies informed?*<br>• *What data were most important in setting policy?*<br>• *What kind of global/regional resources were utilized?*<br>• *How were policies implemented?*<br>• *What led to the selection of these policies?*
17. What policies were made at the local level?<br>***~ External Policies; Outer Setting ~***<br>*~ RQ2. Political Will ~*<br>*~ RQ7. Vaccine Policy and Policy Enforcement ~*
Follow up:<br>• *How were these policies informed?*<br>• *How were they implemented?*<br>• *What led to the selection of these policies?*
18. Did you make any changes to data systems in $\overline{\quad YEAR(S) \quad}$?<br>***~ Execution; Process~***<br>*~ Q3. Information Systems ~*<br>*~ Q6. Workforce ~*

For peer review only

For peer review only - http://bmjopen.bmj.com/site/about/guidelines.xhtml

Date: ___________________________ *Interview Code: ______ - ______ - ______*

Follow up:<br>• *How were these changes made?*<br>• *Why was this change made?*<br>○ *Has this change had the anticipated impact* ***(EX: on coverage or otherwise)****?*<br>• *Did you develop or already have in place a clinic-based immunization record keeping system to track immunizations administered to individuals?*<br>• *Were parents responsible for keeping track of records?*<br>• *To what extent was technology a component of record keeping* ***(EX: computer, phone data collection)****?*
NOTE: REMINDER SYSTEM DEFINED AS A MESSAGE SENT BEFORE APPOINTMENTS TO REMIND PARENTS TO COME; RECALL SYSTEM DEFINED AS A NOTICE SENT TO PARENTS WHO FAIL TO KEEP THEIR APPOINTMENTS.
19. What changes were made to human resources in ______ ?<br>YEAR(S)<br>***~ Execution; Process~***<br>*~ Q6. Workforce ~*
Follow up:<br>• *If so, what kind of changes were made and how?*
20. How do you monitor the performance of immunization programs?<br>***~ Evaluation; Process ~***<br>*~ RQ3. Information Systems ~*
Follow up:<br>• *How is this different from other health systems?*
21. How does this differ from how immunization programs were monitored in the past?<br>***~ Evaluation; Process ~***<br>*~ RQ3. Information Systems ~*
Follow up:<br>• *Who is responsible for collecting data related to measures?*<br>• *How is data used?*<br>• *What measures are included?*<br>• *How often is data collected/shared?*<br>• *How is this information communicated on a local/regional/national level?*<br>• *How do you perceive the quality of data collected?*<br>• *If data quality is poor, is it/how is it used? If poor, what would improve the quality?*<br>• *Are there "independent" checks of the reported data to ensure they are valid?*<br>• *Would any other types of data have been helpful in managing the program?*<br>• *Is there an independent group that evaluates the entire program periodically? If so, please give some details regarding the composition of the group, how it is chosen, and what activities they undertake to evaluate the program?*

For peer review only - http://bmjopen.bmj.com/site/about/guidelines.xhtml

Date: ___________________________ *Interview Code: ______ - ______ - ______*

22. Is there any recognition for being at the top levels of coverage in ______ (COUNTRY). or for making major improvements? If so, can you give some examples of recognition/incentives?
Follow up:<br>• *If yes, have you noticed any issues of misreporting due to the possibility of recognition/incentives?*
23. How do you measure the impact of immunization programs?<br>***~ Evaluation; Process~***<br>*~ RQ3. Information Systems ~*
24. What factors have maintained the sustainability of the immunization program?<br>***~ Evaluation; Process~***
Follow up:<br>• ***Partnerships:*** *Diverse organizations? Collaboration?*<br>• ***Champion:*** *Political advocacy or support?*<br>• ***Funding:*** *Sustained funding? What will happen to the program when Gavi funding is no longer present? What do you think about this planning?*<br>• ***Evaluation:*** *Informed future funding?*<br>• ***Adaptation:*** *How does new evidence and information impact the program?*
25. **Communication**: How does the immunization program maintain community support of childhood vaccines?<br>***~ Evaluation; Process~***<br>*~ RQ3. Information Systems ~*<br>*~ RQ4. Intent/Demand for Vaccines ~*<br>*~ RQ5. Knowledge and Resource Sharing ~*<br>*~ RQ6. Workforce ~*
Follow up:<br>• *To what extent is the immunization program resilient from rumors and misinformation regarding immunizations?*<br>• *What kind of misinformation regarding immunizations exist?*<br>• *How could resilience be improved?*
26. **Media**: How is vaccination portrayed and discussed by the media?<br>***~ Outer Setting ~***<br>*~ RQ3. Information Systems ~*<br>*~ RQ4. Intent/Demand for Vaccines ~*<br>*~ RQ5. Knowledge and Resource Sharing ~*
Follow up:<br>• *What role did the media play in improved coverage?*<br>• *How did the media become involved?*<br>• *How is that involvement sustained?*
27. **Strategic planning:** What kind of planning is there for future resource needs?<br>***~ Evaluation; Process ~***<br>*~ RQ5. Knowledge and Resource Sharing ~*
28. What are the perceptions of corruption in the public sector?<br>***~ Inner Setting ~***<br>*~ RQ2. Political Will ~*
Follow up:<br>• *Perceptions of quality of electoral processes?*

For peer review only - http://bmjopen.bmj.com/site/about/guidelines.xhtml

For peer review only

Date: ___________________________ *Interview Code:* ______ - ______ - ______

- *Judicial institutions?*
- *Anti-corruption efforts?*

For peer review only

For peer review only - http://bmjopen.bmj.com/site/about/guidelines.xhtml

Date: ___________________________ *Interview Code:* ______ - ______ - ______

SECTION H. DISTRICT HEAD, SURVEILLANCE, PREVENTION, VACCINATION PERSONNEL

Objectives:

- To understand the vaccine intervention characteristics during country-specific change points in DTP1/DTP3 coverage
- To identify key internal and external actors, stakeholders, and partnerships in the immunization program both now and in the past
- To identify the strategies behind current immunization programs to inform current context
- To identify previous immunization activities that were key during change points in the DTP1/DTP3 curve
- To understand key barriers and facilitators of interventions in the immunization sector
- To understand health system characteristics, currently and in the past

PART H1 – HEALTH SYSTEM

1. What is the general condition of the health facilities in your district? How does this vary at different levels?
2. Are vaccines available when they are needed? Are vaccines available where they are needed?

Follow up:

- *Are staff available when and where needed?*
- *Are supplies available when and where needed?* ***(EX: Syringes, other supplies?)***
- *Are vaccines availability tracked?*
    - *By location?*
    - *Quantity?*
    - *Timing?*
- *How do vaccines arrive? Are they in ideal conditions?*
- *Where are the vaccines usually stored, and at what temperature?*
- *What levels or conditions prompt restocking?*
    - *Are the minimum supply levels maintained?*
- *How long does it take to restock your supply of vaccines?*

3. How many clinics are available per 1,000 people?

Follow up:

- *What is the average distance/length of time that a person must travel to get to the clinic?*
- *On average, how long does someone wait to receive vaccines?*
- *Are there other services included in vaccine visits?*

PART H2 – IMMUNIZATION PROGRAM

4. What is a specific intervention that contributed to the increase in vaccine coverage?
5. What was your involvement in the intervention(s) and the changes that followed? *(IF NO DIRECT INVOLVEMENT, SKIP TO Q7)*

Follow up:

- *Were these directives from the ministry?*
- *How did you decide which changes to make?*
- *How did this intervention affect people's roles and responsibilities at different levels in the health system?*

For peer review only - http://bmjopen.bmj.com/site/about/guidelines.xhtml

Date: ____________________________ *Interview Code: ______ - ______ - ______*

6. What were key activities in planning changes for the program?

~ Planning; Process ~
~ RQ5. Knowledge and Resource Sharing ~
~ RQ6. Workforce ~

Follow up:

- *Did you have an action plan?*
- *A theory of change or log frame?*
- *What challenges did you face in the early planning phases?*
    - *How were these challenges overcome **(EX: Internal to agency/external to agency)**?*

7. What were key training activities?

~ Planning, Executing; Process ~
~ RQ5. Knowledge and Resource Sharing ~
~ RQ6. Workforce ~

Follow up:

- *If no training activities, what modality did the intervention have?*
- *If training activities, do you have the following information, and can we access it?*
    - *Who was involved in trainings **(NOTE: at all levels)**?*
    - *Who taught the training?*
    - *How were they selected*
        - *Trainers*
        - *Trainees?*
    - *What was their motivation/incentive for involvement?*
    - *How were the trainings conducted?*
    - *What were the expectations of the participants in training?*

8. What kind of evidence did you use to determine [changes/interventions]?

~ Planning; Process ~
~ RQ5. Knowledge and Resource Sharing ~

Follow up:

(E.g., outbreaks, surveillance data).

- *You mentioned ______ (DATA SOURCE NAME(S)). What role did this information have in the push to increase vaccine coverage?*
- *Can you explain how ______ (DATA SOURCE NAME(S)) is collected?*
- *How is this ______ (DATA SOURCE NAME(S)) integrated into other systems?*
- *How does the surveillance system in ______ (COUNTRY) work?*

~ Planning; Process~
~ RQ5. Knowledge and Resource Sharing~

9. What do you see as critical for implementation of the [intervention(s)] to succeed?

~ Reflecting and Evaluating; Process~
~ Q6. Workforce ~

For peer review only - http://bmjopen.bmj.com/site/about/guidelines.xhtml

Date: ___________________________ *Interview Code:* ______ - ______ - ______

Probes:<br>• *What advice would you give to another person/region/country who was going to implement a similar program/project to yours?*<br>• *What are you most proud of in terms of this work?*
10. What challenges/difficulties did you encounter during implementation of the interventions?<br>***~ Reflecting and Evaluating; Process ~***
Follow up:<br>• *How were these challenges overcome?*<br>• *What contributed to these challenges?*<br>• *How were decisions made about what to do?*<br>• *Who contributed to this decision-making? Why?*<br>• *What challenges did you encounter with any new policies made?*<br>• *Of these challenges, which challenge was the greatest in this region?*<br>•
11. What areas of the district have seen the greatest improvement in vaccine rates? Can you share this data?<br>***~ Evaluation; Process~***<br>*~ RQ3. Information Systems ~*<br>*~ RQ4. Intent/Demand for Vaccines ~*
Follow up:<br>• *Was the project specifically tailored to this location?*<br>• *Was it targeted as a critical area for change?*<br>• *What areas saw the quickest improvement?*<br>• *Why do you think these areas had the greatest improvement?*
12. What areas did not experience significant changes?<br>***~ Evaluation; Process~***<br>*~ RQ3. Information Systems ~*<br>*~ RQ4. Intent/Demand for Vaccines ~*
Follow up:<br>• *Why do you think this is?* ***(EX: May have already had high coverage, may not relate to challenges)***<br>• *What were the challenges?*<br>○ *Why?*<br>• *What has been/might be done to mitigate this?*
13. What populations historically were the most vulnerable to low coverage? Why?<br>***~ Evaluation; Process~***
Follow up:<br>• *How did you identify these populations as vulnerable?*<br>○ *Both previously and now*

For peer review only - http://bmjopen.bmj.com/site/about/guidelines.xhtml

Date: ___________________________ Interview Code: ______ - ______ - ______

- *How did you reach out to these populations?*
- *What strategy/strategies do you use to reduce inequities?*
- *What strategy did you previously use to reduce inequities?*
- *What kind of data supports this?*
- *Is this data you can share with me?*

14. What interventions were offered through Community Health Workers?

~ Execution; Process ~
~ RQ6. Workforce ~

Follow up:

- *Outreach activities? Training?*
  - *Including any kind of reminder system for people to get vaccinations?*
    - ***What kind of system was this? (Cellphone texts, in-person reminders (by who?), church/religious institution reminders?)***
    - *How was technology a component of this system?*
    - *Did you develop or already have in place a clinic-based immunization record keeping system to track immunizations administered to individuals?*
- *Tools used?*
- *How were these developed?*
- *What led to the selection of these interventions?*
- *Was there variation in different geographic regions etc. If so, why?*
- *How were/are CHWs motivated/incentivized for involvement?*
- *What are the job requirements to be a CHW?*
- *How have responsibilities shifted over time? How do you think this has impacted their performance? Their motivation?*
- *What kind of current involvement does the Neighborhood Health Committee have in current CHW programming and responsibilities? What kind of role did they have in the past?*
- *What kinds of continued training/capacity building are provided to CHWs?*

~ Beneficiary Needs and Resources; Outer Setting ~

15. What interventions were offered at various levels of the health system? For providers?

~ Execution; Process~
~ RQ3. Information Systems ~
~ RQ6. Workforce ~

Follow up:

- *How were these developed?*
- *How were they implemented? Training*
- *Why were they implemented in the way that they were?*
- *What led to the selection of these interventions?*
- *Was there variation in different geographic regions etc. If so, why?*
- *What is the educational background of providers?*

16. What key interventions or programs were offered to parents or soon-to-be parents?

~ Intervention Characteristics~
~ Outer Setting ~
~ RQ4. Intent/Demand for Vaccines ~

For peer review only - http://bmjopen.bmj.com/site/about/guidelines.xhtml

Date: ___________________________ *Interview Code:* ______ - ______ - ______

Follow up:

- *Were these tailored to different areas/cultures/religions/languages/etc.?*
- *How were these targeted specifically to mothers, fathers or alternative caretakers?*
- *How were they developed?*
- *Where did you get the idea for these interventions?*
  - *Did you use some other framework or intervention to inform your intervention?*
- *How were they delivered?*
- *Who was engaged in delivery?*
- *What determined their involvement?*
- *Who determines the programming/methods?*
- *Who approves the programming/methods?*
- *Who formulates the messages?*
- *What kinds of modalities were used?*
- *What kind of communication materials (if any) were utilized? Mass media? Dissemination through health centers? Other?*
- *How did you get individuals to participate in this program?*
- *What led to their selection?*
- *Could you share any materials with us?*

~ Beneficiary needs and resources ~

17. Did you make any changes to the supply chain in $\underset{YEAR(S)}{\_\_\_\_\_\_\_\_}$? If so, tell us how.

~ Execution; Process ~
~ Q6. Workforce ~

Follow up:

- *What worked well?*
  What were challenges?

18. What strategies did you use to reduce the drop-out rate?

19. How do you monitor the performance of immunization programs?

~ Evaluation; Process ~
~ RQ3. Information Systems ~

20. How does this differ from how immunization programs were monitored in the past?

~ Evaluation; Process ~
~ RQ3. Information Systems ~

Follow up:

- *Who is responsible for collecting data related to measures?*
- *How is data used?*
- *What measures are included?*
- *How often is data collected/shared?*
- *How is this information communicated on a local/regional/national level?*
- *How do you perceive the quality of data collected?*

For peer review only - http://bmjopen.bmj.com/site/about/guidelines.xhtml

Date: ___________________________ *Interview Code: ______ - ______ - ______*

• *If data quality is poor, is it/how is it used? If poor, what would improve the quality?*<br>• *Are there "independent" checks of the reported data to ensure they are valid?*<br>• *Would any other types of data have been helpful in managing the program?*<br>• *Is there an independent group that evaluates the entire program periodically? If so, please give some details regarding the composition of the group, how it is chosen, and what activities they undertake to evaluate the program?*
21. Is there any recognition for being at the top levels of coverage in the country or for making major improvements? If so, can you give some examples of recognition/incentives?
Follow up:<br><br>• *Have you noticed any issues of misreporting due to the possibility of rewards?*
22. What factors have maintained sustainability of the immunization program?<br><br>***~ Evaluation; Process~***
Follow up:<br><br>• *Partnerships: Diverse organizations? Collaboration?*<br>• *Champion: Political advocacy or support?*<br>• *Funding: Sustained funding? What will happen to the program when Gavi funding is no longer present? What do you think about this planning?*<br>• *Evaluation: Informed future funding?*<br>• *Adaptation: How does new science and knowledge impact the program?*
a. **Communication**: How does the immunization program secure awareness of the issue of childhood vaccines?<br><br>***~ Evaluation; Process~***<br>*~ RQ3. Information Systems ~*<br>*~ RQ4. Intent/Demand for Vaccines ~*<br>*~ RQ5. Knowledge and Resource Sharing ~*<br>*~ RQ6. Workforce ~*
Follow up:<br><br>• *How does the immunization program maintain community support of childhood vaccines?*<br>• *To what extent is the immunization program resilient from rumors and misinformation regarding immunizations?*<br>• *How could resilience be improved?*
b. **Media**: How is vaccination portrayed and discussed by the media?<br><br>***~ Outer Setting ~***<br>*~ RQ3. Information Systems ~*<br>*~ RQ4. Intent/Demand for Vaccines ~*<br>*~ RQ5. Knowledge and Resource Sharing ~*
Follow up:<br><br>• *What role did the media play in improved coverage?*<br>• *How did the media become involved and how was that involvement sustained?*

For peer review only - http://bmjopen.bmj.com/site/about/guidelines.xhtml

Date: ___________________________ *Interview Code: ______ - ______ - ______*

c. **Strategic planning**: What kind of planning is there for future resource needs?<br><br>***~ Evaluation; Process ~***<br>*~ RQ5. Knowledge and Resource Sharing ~*
23. What are the perceptions of corruption in the public sector?<br>***~ Inner Setting ~***<br>*~ RQ2. Political Will ~*
Follow up:<br><br>• *Perceptions of quality of electoral processes?*<br>• *Judicial institutions?*<br>• *Anti-corruption efforts?*

For peer review only

For peer review only - http://bmjopen.bmj.com/site/about/guidelines.xhtml

Date: ___________________________ *Interview Code:* ______ - ______ - ______

SECTION I. CLINIC HEAD

PART 1 – HEALTH SYSTEM

Objectives:

- To understand the vaccine intervention characteristics during country-specific change points in DTP1/DTP3 coverage
- To identify key internal and external actors, stakeholders, and partnerships in the immunization program both now and in the past
- To identify the strategies behind current immunization programs to inform current context
- To identify previous immunization activities that were key during change points in the DTP1/DTP3 curve
- To understand key barriers and facilitators of interventions in the immunization sector
- To understand health system characteristics, currently and in the past
- To understand community norms and practices related to vaccinations

1. How would you describe the general condition of your health facility?

2. Are vaccines available when and where they are needed?

Follow up:

- *Are staff available when and where needed?*
- *Are supplies available when and where needed?* ***(EX: Syringes, other supplies)***.
- *Are vaccines availability tracked?*
  - *By location?*
  - *Quantity?*
  - *Timing?*
- *How do vaccines arrive? Are they in ideal conditions?*
- *Where are the vaccines usually stored, and at what temperature?*
- *What levels or conditions prompt restocking?*
  - *Are there minimum supply levels maintained?*

How long does it take to restock your supply of vaccine?

3. *How does* [COMMUNITY] *view* vaccines?

~ ***Decision-making process****~*
~Current factors behind immunization~
~Demand-side~

Follow up:

- *What are the main facilitators to people getting vaccines in your community?*
  - ***Examples****:*
    - *Encouragement from healthcare workers*
    - *Shame*
    - *Geography/Accessibility*
    - *Trust of vaccinators/healthcare workers*

For peer review only - http://bmjopen.bmj.com/site/about/guidelines.xhtml

Date: ___________________________ *Interview Code: ______ - ______ - ______*

- *Messaging/Increased Awareness*
- *Community expectation*
- *Seasonality*
- *Availability*

- *What are the main barriers to people getting vaccines in your community?*
  - ***Examples**:*
    - *Geography/Accessibility*
    - *Mistrust of healthcare workers*
    - *Stock outs*
    - *Cold chain*
    - *Vaccine hesitancy (ex. Side effects)*
    - *Influence of community/religious leaders*
    - *Seasonality*
    - *Wastage*
- *How are these barriers addressed?*
- *Are there groups less likely to prioritize vaccines? Who and why?*
- *Are there children who receive some infant vaccines but don't receive others? Why?*
- *How are vaccines prioritized compared to other health interventions/measures?*

4. How have outbreaks of diseases affected vaccination rates?

~Evaluation; Process~
~RQ3. Information Systems~
~RQ4. Intent/Demand for Vaccines~

5. What services do you think the government is responsible for? Compared to other government responsibilities, how important are vaccines?

~Intervention; Outer setting~

Follow up:

- *Why are they important/not important?*
- *What is the most important to [COMMUNITY]?*
- *What is the least important to [COMMUNITY]?*

6. How has this health facility's ability to provide vaccines changed over time?

Follow up:
- *What changed?*
  - ***Examples**:*
    - *Demand from community?*
    - *Supply*
    Improved systems?
    - *Training?*
    - *Government emphasis?*
- *About what year was this?*
  - *What other events happened around this time?*
    - *Political*

For peer review only - http://bmjopen.bmj.com/site/about/guidelines.xhtml

Date: ___________________________ *Interview Code: ______ - ______ - ______*

- *Infrastructure changes*
- *Other health issues*
- *Can you think of any certain events that increased or decreased vaccinations?*
  - *Strikes*
  - *Extreme weather*
  - *Outbreaks? What disease? Vaccine preventable (flu, measles, mumps, rubella, pertussis)?*

PART I2 – IMMUNIZATION PROGRAM

7. What is a specific intervention that contributed to the increase in vaccine coverage?

Follow up:

- *What was your involvement in this intervention(s) and the changes that followed?*
- *How were the changes received?*
- *Who were the key decision-makers of this intervention?*

8. What key interventions or programs were offered to parents or soon-to-be parents?

~ Intervention Characteristics~
~Outer Setting ~
~ RQ4. Intent/Demand for Vaccines ~

Follow up:

- *Were these tailored to different areas/cultures/religions/languages/etc.?*
- *How were these targeted specifically to mothers, fathers or alternative caretakers?*
- *How were they developed?*
- *Where did you get the idea for these interventions?*
  - *Did you use some other framework or intervention as to inform your intervention?*
- *How were they delivered?*
- *Who was engaged in delivery?*
- *What determined their involvement?*
- *Who determines the program/methods?*
- *Who formulates the messages?*
- *What kinds of modalities were used?*
- *What kind of communication materials (if any) were utilized? Mass media? Dissemination through health centers? Other?*
- *How did you get individuals to participate in this program?*
- *What led to their selection?*
- *What led to their selection?*
- *What encouraged parents to be more engaged in their children's health?*
- *Was this specific to immunization or general health?*
- *Were these interventions offered in combination with other health interventions?*
- *What are you doing to reduce dropout rates?*
- *What kind of awareness campaigns are there?*
- *Could you share any materials with us?*

~ Beneficiary needs and resources ~

9. What interventions were offered through Community Health Workers?

~ Execution; Process ~

For peer review only - http://bmjopen.bmj.com/site/about/guidelines.xhtml

Date: ___________________________ *Interview Code: ______ - ______ - ______*

~ RQ6. Workforce ~

Follow up:

- *Outreach activities? Training?*
  - *Including any kind of reminder system for people to get vaccinations?*
    - ***What kind of system was this? (Cellphone texts, in-person reminders (by who?), church/religious institution reminders?)***
    - *How was technology a component of this system?*
    - *Did you develop or already have in place a clinic-based immunization record keeping system to track immunizations administered to individuals?*
- *Tools used?*
- *How were these developed?*
- *What led to the selection of these interventions?*
- *Was there variation in different geographic regions etc. If so, why?*
- *How were/are CHWs motivated/incentivized for involvement?*
- *What are the job requirements to be a CHW?*
- *How have responsibilities shifted over time? How do you think this has impacted their performance? Their motivation?*
- *Is there a correlation between incentives and outcomes?*
- *What kind of current involvement does the Neighborhood Health Committee have in current CHW programming and responsibilities? What kind of role did they have in the past?*
- *What kinds of continued training/capacity building are provided to CHWs?*

~ Beneficiary Needs and Resources; Outer Setting ~

10. What interventions were offered at various levels of the health system? To providers?

~ Execution; Process~
~ RQ3. Information Systems ~
~ RQ6. Workforce ~

Follow up:

- *How were these developed?*
- *How were they implemented? Training?*
- *Why were they implemented in the way that they were?*
- *What led to the selection of these interventions?*
- *Was there variation in different geographic regions etc. If so, why?*
- *What is the educational background of providers?*

11. How is your work monitored?

12. How do you monitor the performance of immunization programs?

~ Evaluation; Process~
~ RQ3. Information Systems ~

13. How does this differ from how immunization programs were monitored in the past?

~ Evaluation; Process~
~ RQ3. Information Systems ~

For peer review only - http://bmjopen.bmj.com/site/about/guidelines.xhtml

Date: ___________________________ *Interview Code: ______ - ______ - ______*

Follow up:<br>• *Who is responsible for collecting data related to measures?*<br>• *How is data used?*<br>• *What measures are included?*<br>• *How often is data collected/shared?*<br>• *How is this information communicated on a local/regional/national level?*<br>• *How do you perceive the quality of data collected?*<br>• *If data quality is poor, is it/how is it used? If poor, what would improve the quality?*<br>• *Are there "independent" checks of the reported data to ensure they are valid?*<br>• *Would any other types of data have been helpful in managing the program?*<br>• *Is there an independent group that evaluates the entire program periodically? If so, please give some details regarding the composition of the group, how it is chosen, and what activities they undertake to evaluate the program?*
14. Is there any recognition for being at the top levels of coverage in the country or for making major improvements? If so, can you give some examples of recognition/incentives?
Follow up:<br>• *Have you noticed any issues of misreporting due to the possibility of rewards?*
15. How do you measure the impact of immunization programs?
16. What was key to success when implementing interventions?<br>***Reflecting and Evaluating; Process~***<br>*~ Q6. Workforce ~*
Follow up:<br>• *What advice would you give to another person/region/country who was going to implement a similar program/project to yours?*<br>• *What are you most proud of in terms of this work?*
17. What challenges/difficulties did you encounter when implementing interventions?<br>***~ Reflecting and Evaluating; Process~***
Follow up:<br>• *How were these challenges overcome?*<br>• *What challenges did you encounter with any new policies made?*
18. What changes have occurred in programming since this intervention(s) was implemented?<br>***~ Evaluation; Process~***<br>*~ RQ3. Information Systems ~*
19. What changes have occurred in programming since ______ (YEAR(S)) /these interventions were implemented?<br>***~ Evaluation; Process~***
20. What factors have maintained the sustainability of the immunization program?<br>***~ Evaluation; Process~***

For peer review only - http://bmjopen.bmj.com/site/about/guidelines.xhtml

Date: ___________________________ *Interview Code:* ______ - ______ - ______

Follow up:<br><br>• ***Partnerships:*** *Diverse organizations? Collaboration?*<br>• ***Champion:*** *Political advocacy or support?*<br>• ***Funding:*** *Sustained funding? What will happen to the program when Gavi funding is no longer present? What do you think about this planning?*<br>• ***Evaluation:*** *Informed future funding?*<br>• ***Adaptation:*** *How does new science and knowledge impact the program?*
d. **Communication**: How does the immunization program secure community support or awareness of the issue of childhood vaccines?<br><br>***~ Evaluation; Process ~***<br>*~ RQ3. Information Systems ~*<br>*~ RQ4. Intent/Demand for Vaccines ~*<br>*~ RQ5. Knowledge and Resource Sharing ~*<br>*~ RQ6. Workforce ~*
Follow up:<br><br>• *How do you define awareness? What is the community aware of (EX. Benefits, services offered, etc.)*<br>• *How does the immunization program maintain community support of childhood vaccines?*<br>• *To what extent is the immunization program resilient from rumors and misinformation regarding immunizations?*<br>• *How could resilience be improved?*
e. **Media**: How is vaccination portrayed and discussed by the media?<br>***~ Outer Setting ~***<br>*~ RQ3. Information Systems ~*<br>*~ RQ4. Intent/Demand for Vaccines ~*<br>*~ RQ5. Knowledge and Resource Sharing ~*
Follow up:<br><br>• *What role did the media play in improved coverage?*<br>• *How did the media become involved and how was that involvement sustained?*
f. **Strategic planning:** To what extent is there planning for future resource needs?<br><br>***~ Evaluation; Process~***<br>*~ RQ5. Knowledge and Resource Sharing ~*
21. What are the greatest barriers to sustained coverage?<br>***~ Reflection; Process~***
22. What are the perceptions of corruption in the public sector?<br>***~ Inner Setting ~***<br>*~ RQ2. Political Will ~*
Follow up:<br><br>• *Perceptions of quality of electoral processes?*<br>• *Judicial institutions?*<br>• *Anti-corruption efforts?*

For peer review only - http://bmjopen.bmj.com/site/about/guidelines.xhtml

Date: ____________________________ *Interview Code:* ______ - ______ - ______

SECTION J. COMMUNITY LEADERS (RELIGIOUS LEADERS, HEAD MAN, CHIEFS,

Objectives:

- To understand their role, if any, as it relates to health interventions, specifically immunization coverage
- To identify key community stakeholders who have a role in immunization programming
- To understand the cultural context and environmental context of the selected community
- To understand what drives community acceptance of immunizations
- To understand the motivations of key community stakeholders in vaccine intervention characteristics during country-specific change points in DTP1/DTP3 coverage
- To identify the strategies behind current immunization programs to inform current context
- To identify previous immunization activities — including specific interventions, delivery systems, policies, facilitators and barriers — that were key during change points in the DTP1/DTP3 curve
- To understand specific barriers and facilitators to implementation of interventions in the immunization sector

PART J1 – INTERVIEW INTRODUCTION

<u>Interviewer Script</u>: Thank you very much for your willingness to meet with us today and share your experience and knowledge regarding the immunization program in ____________ (COUNTRY). I'd like to begin by learning about you and your role related to the immunization program.

1. Can you tell me about your role in ____________ (THIS COMMUNITY) ?

~ Individual Setting ~

Follow up:

- *How does your role relate to health care programming?*
- *How does your role relate to the immunization program?*
- *Responsibilities (past and present)*
- *What are the contributions you feel you've made in the immunization programming in your community?*
- *What has motivated you to make these contributions?*

2. Who are other community leaders/stakeholders who have had an impact on the immunization program?

~ Inner Setting ~

Follow up:

- *What role did they play?*
- *What were their key contributions?*
- *What motivated their involvement?*
- *What were their top three incentives for performing well?*
- *What are the stakeholders' party/group affiliation?*
- *Are their positions relevant to vaccinations?*

<u>Interviewer Script</u>: Now I'd like to learn about the health care services in your community, and how immunization coverage plays a part. I'd also like to hear a bit more about community attitudes towards immunizations.

For peer review only - http://bmjopen.bmj.com/site/about/guidelines.xhtml

Date: ___________________________ *Interview Code:* ______ - ______ - ______

3. How does *[COMMUNITY]* view vaccines?

~ Inner Setting ~
~ Evaluation; Process ~
~Decision making process; demand-side~
~current factors behind immunization~

Probes:

- *What are the main facilitators to people getting vaccines in your community]?*
  - *Encouragement from health care workers*
  - *Shame*
  - *Geography*
  - *Enforcement*
  - *Trust of vaccinators/health care workers*
  - *Messaging/Awareness*
  - *Community expectation?*
  - *Fundamental right?*
  - *Seasonality?*
- *What are the main barriers to people getting vaccines in your community?*
  - *Geography*
  - *Mistrust of health care workers*
  - *Stock out*
  - *Cold chain*
  - *Vaccine hesitancy (ex. side effects)*
  - *Influence of community/religious leaders*
  - *Seasonality*
  - *Wastage*
- *How are these barriers addressed?*
- *Are there groups less likely to prioritize vaccines? Who and why?*
- *Are there children who receive infant vaccines (ex. DTP1) but don't receive others (ex. DTP2, DTP3)? Why?*
- *How are vaccines prioritized compared to other health interventions/measures?*

4. How have outbreaks of diseases affected mandatory vaccination rates?

~ Evaluation; Process ~
~ RQ3. Information Systems ~
~ RQ4. Intent/Demand for Vaccines ~

Probes:

Did vaccination rates increase or decrease?

5. Compared to other government responsibilities - like public safety, roads and infrastructure, education – how important are vaccines in this community?

~Intervention; Outer Setting~

Probes:

- *Why are they important/not important?*
- *What is the most important to you / [COMMUNITY]?*
- *What is the least important to you / [COMMUNITY]?*
- *Who do you believe is responsible for providing vaccines?*

For peer review only - http://bmjopen.bmj.com/site/about/guidelines.xhtml

Date: ___________________________ *Interview Code:* ______ - ______ - ______

- *Government?*
- *NGOs and aid agencies?*

6. How has the health facility's ability to provide vaccines changed over time?

~Intervention; Inner setting~

Probes:

- *What changed?*
  - *Demand from community?*
  - *Supply?*
  - *Improved systems?*
  - *Training?*
  - *Governmental emphasis?*
- *About what year was this?*
  - *What other events happened around this time?*
    - *Political*
    - *Infrastructure changes*
    - *Other health issues*
- *Can you think of any certain events that increased or decreased vaccinations?*
  - *Strikes*
  - *Extreme weather*
  - *Outbreaks*
    - *What diseases?*
      - *Vaccine preventable (flu, measles, mumps, rubella, pertussis etc.)*
      - *Non-vaccine preventable?*

7. What are specific interventions/programming shifts that have happened in the last ten years?

[Intent: Identify past immunization activities and interventions; identify previous key actors in the program]

~ Evaluation; Process~

[CFIR – Intervention, Outer Setting, Inner Setting, Characteristics of Individuals, Process]

Probes:

- *What intervention had the greatest impact?*
- *How was this intervention implemented in your community?*
- *How did people react to this intervention?*
- *What were the facilitators to uptake?*
- *What were barriers to uptake?*

8. What factors have contributed to the sustainability of the immunization program?

~ Evaluation; Process~

e. **Organizational capacity:** Were there organizational systems in place to support the program/intervention needs?

~ Evaluation; Process~
~ RQ1a. Why and How? ~
~ RQ1b. Networks ~

For peer review only - http://bmjopen.bmj.com/site/about/guidelines.xhtml

Date: ___________________________ *Interview Code: ______ - ______ - ______*

f. **Communication:** How does the immunization program maintain community support or awareness of the issue of childhood vaccines?<br>***~ Evaluation; Process ~***<br>*~ RQ3. Information Systems ~*<br>*~ RQ4. Intent/Demand for Vaccines ~*<br>*~ RQ5. Knowledge and Resource Sharing ~*<br>*~ RQ6. Workforce ~*
Follow-up:<br><br>*To what extent is the immunization program resilient from rumors and misinformation regarding immunizations?*<br>*How could resilience be improved?*
g. **Media:** How is vaccination portrayed and discussed by the media?<br>***~ Outer Setting ~***<br>*~ RQ3. Information Systems ~*<br>*~ RQ4. Intent/Demand for Vaccines ~*<br>*~ RQ5. Knowledge and Resource Sharing ~*
Follow-up:<br><br>• *What role did the media play in improved coverage?*<br>• *How did the media become involved and how was that involvement sustained?*
h. **Strategic planning:** To what extent is there planning for future resource needs?<br><br>***~ Evaluation; Process~***<br>*~ RQ5. Knowledge and Resource Sharing ~*
8. What are the greatest risks to sustained coverage?<br><br>***~ Reflection; Process~***
9. What lessons have you learned from working on/creating the immunization program?<br><br>***~ Evaluation [IP]***

For peer review only

For peer review only - http://bmjopen.bmj.com/site/about/guidelines.xhtml

Date: ___________________________ *Interview Code:* ______ - ______ - ______

PART IV – CONCLUSION
Interviewer Script: Before we end this interview, I want to ask if you have any questions, comments, or anything else you think is important for us to know.
(Allow time for questions, comments).

1. What lessons have you learned from working on the immunization program?

~ Evaluation; Process~

2. During the time we were speaking, you mentioned the following people: ________ (NAME(S)) who were/are also stakeholders in the immunization program. Can you provide us introductions to these individuals? Who else would you suggest that we speak to about the immunization program?

~ Characteristics of Individuals ~

3. You also mentioned the following data sources: ________ (DATA SOURCE NAME(S)). How can I access this data? Are you able to assist me? Are there other relevant documents you think I should have? How can I access this?

Interviewer Script: Our next steps in the project are to continue key informant interviews with other stakeholders, and to analyze some of the quantitative data we are collecting.

4. What level of update/involvement would you like to have in the project moving forward?

Interviewer Script: Thank you much for your time and for answering my questions today. You have provided great information that will helpful during our research. If there are no other questions or concerns, then I will turn off the recorder, and you are free to leave as you please. Thank you again.

NOTES:

For peer review only - http://bmjopen.bmj.com/site/about/guidelines.xhtml

Appendix 3 – Reflexivity Statement

1. How does this study address local research and policy priorities?
To address local needs, the Emory study team partnered with local research groups in Nepal, Zambia, and Senegal. This partnership ensured the study was aligned with the context and priorities of each country. Data collection tools were iterative and were created with advice and input from both in-country researchers, local stakeholders, and external technical advisors. The tailoring of the project to each country allowed for this hypothesis generating study to best reflect the main needs and priorities of the countries surveyed.

2. How were local researchers involved in study design?
Local researchers were key to the realization of this project. The in-country study teams helped draw our KII list of key stakeholders and past influential leaders, along with helping to secure meetings. We worked with the local teams to develop our tools, and to edit the questions or procedures as needed based on cultural and political considerations. Partners also helped to determine the best locations for our sample and were responsible for finding individuals for FGDs [1].

3. How has funding been used to support the local research team?
The local research teams were treated as sub-contractors on this project and were paid fairly for their time and effort on this project. We did not have any free or un-funded contributions of local researchers on behalf of the project.

4. How are research staff who conducted data collection acknowledged?
Research staff who conducted data collection were acknowledged as authors in their respective country case studies [2-4].

5. Do all members of the research partnership have access to study data?
In-country research partners have access to all KII and FGD transcripts from their respective countries.

6. How was data used to develop analytical skills within the partnership?
While we did not focus on capacity building during this study, the Emory research staff conducted trainings with each in-country research group. These trainings were over 7 – 14 days, and reviewed the study protocols, data collection guidelines, and general qualitative research best practices.

7. How have research partners collaborated in interpreting study data?
Within each country level case study, our in-country partners were actively engaged in interpretations; the study teams had weekly meetings to discuss the progress and findings of KIIs and FGDs. In addition, partners were involved in the drafting of the manuscripts – the case studies and this cross-cutting synthesis [2-4].

8. How were research partners supported to develop writing skills?
Our research partners collaborated on the manuscript drafts and were added as authors for their contributions. Additionally, several student graduate assistants were not American, and from LMICs; students were added as authors for their contributions.

9. How will research products be shared to address local needs?
Results from this study have already been disseminated to the respective leaders and stakeholders of each country; the meeting was led by the in-country PI and was attended by the Emory research team. A country-specific report, along with the manuscripts, were disseminated. We met with our TAG to disseminate results to global immunization leaders, and continue to collaborate with the in-country PIs on opportunities for additional outreach.

For peer review only

10. How is the leadership, contribution and ownership of this work by LMIC researchers recognised within the authorship?

On this cross-country synthesis, the in-country PIs are added as senior authors. For the individual case studies, all in-country researchers are included as authors [2-4].

11. How have early career researchers across the partnership been included within the authorship team?

In country research partners hired their own staff, and there were different levels of experience represented across the researchers. Early career researchers were included and mentored within each in-country research team, as well as within the Emory team.

12. How has gender balance been addressed within the authorship?

While the Emory and in-country PI's are men, most of the research staff was comprised of women. Guidance for authorship was drafted prior to writing manuscripts, and all were given opportunities to contribute. Those who met the criteria were included as authors; individual contributions, and specific authors, are included on the country case studies [2-4]. This manuscript is the synthesis, and country-level PIs and the Emory team are included as authors.

13. How has the project contributed to training of LMIC researchers?

This project had 7 – 14 day trainings for each country's research team, wherein best practices for qualitative research were taught along with the study's aims and tools. In-country PI's provided additional assistance and training to staff as needed, however, our collaborators were all well-trained professionals and knowledgeable in their areas of expertise.

14. How has the project contributed to improvements in local infrastructure?

This project was a hypothesis generating study, and therefore did not contribute to infrastructure improvement. However, we hope the findings of this study will be of use to program managers and funders in strengthening and building further immunization infrastructure that is both resilient and sustainable.

15. What safeguarding procedures were used to protect local study participants and researchers?

KII and FGD participants gave researchers signed, informed consent; their information was transcribed and de-identified. See our protocol and case study papers for further information [1-4]. There was only minimal risk to both participants and researchers; local PI's were responsible for any context specific safeguarding.

For peer review only

1. Bednarczyk, R.A., et al., *Exemplars in vaccine delivery protocol: a case-study-based identification and evaluation of critical factors in achieving high and sustained childhood immunisation coverage in selected low-income and lower-middle-income countries.* BMJ Open, 2022. **12**.
2. Micek, K., et al., *Critical success factors for routine immunization performance: A case study of Zambia 2000 to 2018.* Vaccine X, 2022. **11**.
3. Hester, K.A., et al., *Critical success factors for high routine immunization performance: A case study of Nepal.* Vaccine X, 2022. **12**(100214).
4. Sakas, Z., et al., *Critical success factors for high routine immunization performance: A case study of Senegal.* medRxiv, 2022: p. 2022.01.25.22269847.

For peer review only

Appendix 4: Additional key informant quotes, by country and domain, illustrating drivers for vaccine coverage from the conceptual framework [15-17]

Domain	Nepal	Senegal	Zambia
Governance and financing<br><br>The processes that guide policies and operations within the health system, including collaboration (effective working relationships, including alignment of priorities) and commitment (dedication to activities and responsibilities).	*"A provision was made in the constitution, and [vaccines] became a fundamental right, so the government has a responsibility to provide. And management of currently running programs also [focuses on] children who have missed vaccines." (Nepal Health Research Council Staff)*	*"When we have a strategic document to work on, the partners get involved according to their area of expertise, and we work together when it comes to implementing and monitoring the vaccine program. In this teamwork, you cannot tell who is the partner or who is from the Ministry of Health, because we work hand in hand. We are in the same boat, we want to have success." (External partner at the national level)*	*"Once you have identified that there is an issue that needs attention, these policies are revised and updated so that they match what is current. [Policies] are more or less like living documents to match the prevailing circumstances. Our planning has improved quite a lot because it's now bottom-up. So, we are the ones that have a lot of input in these documents." (Provincial Health Officer)*
Health systems strengthening<br><br>Strategies that are designed to sustainably improve health system performance (across all sectors) through an integrated and horizontal approach, with a focus on equity.	*"No wards will be deprived of health centers, and if the health center isn't enough to provide health services, then village clinics, immunization centers (outreach clinics), and urban health clinics will be established. If services are provided from these health centers, quality of the vaccines are maintained, and health centers are accessible to all - then good immunization coverage can be achieved." (Department of Health Services within the MOH)*	*"We tried to see what was hidden behind the weak coverage - especially the poor availability of resources including health structures and sufficient staff... a diagnosis was made to improve the indicators, health structures, i.e. creating a lot of health posts, and recruiting staff to be set up in each health post, or at least one vaccination unit to really try to reach out to the entire population in order to have good coverage." (National level stakeholder)*	*"Previously, there was no public health department at a grassroots level, it was just at the National level... So, now the Public Health Specialist is on the ground to try to see that this primary health care activity is promoted. And I think with that supervision, with the introduction of the public health nurses, Health promotion departments, I think these interventions couldn't be achieved higher in terms of immunization activities." (District Health Director)*
Community engagement<br><br>Responsibility of the community to implement vaccination program activities through local leadership and volunteers, fostered by government initiatives and cultural values.	*"Mothers' groups are responsible to give education and information to the parents.... If they don't support the program, then it won't be successful. They are the ones who build awareness and give knowledge and information to the people at the grassroots level." (Department of Health Services in the Ministry of Health and Population)*	*"Community health workers play a very important role – they are in direct contact with the population. They are the ones who live in the community, and they identify more with the community. So, there is a relationship of trust between them and the population." (National level government stakeholder)*	*"We make sure that the community is fully engaged and aware of the benefit of ensuring that their children are vaccinated. It is important that we ensure that within the community, the key gatekeepers are helping us." (Provincial Health Officer)*

For peer review only

Adaptive capacity<br><br>Ability to sufficiently consider contextual factors, supported by timely evaluation of quality data, to tailor vaccine programming based on local needs and priorities.	*"Using HMIS, which is our biggest data source, we send daily, weekly, or monthly reports to districts using phones. We visit districts and contact the responsible person. We understand the situation and we try to solve those problems. We don't depend on the system. We explore, analyze, and address if there are issues outside the system. We do review meetings at the end of the year from the lessons we learned. We also make periodic updates." (Regional MoH Director)*	*"During regional-level meetings, we hear good ideas from the districts. When Dakar had a measles epidemic, we brought together all the districts to tell us how to plan in order to have good measles vaccine coverage. Some of the districts came up with strategies that we found very inspiring. One of them was to hold the vaccination sessions beyond regular work hours to reach the people who are not free during the day. The other strategy was to organize sessions on weekends. As soon as we put these strategies in place, we noticed that the coverage increased right away. It dawned on us that the parents did not refuse the vaccination but that they were busy by their daily occupations." (External partner at the national level)*	*"We have been advising facilities that they need to own the data; they have to make sure that they utilize it by analyzing… If they are not meeting their targets, they need to reflect on the data and see where they are going wrong… For those [health facilities] that are performing well, we find out what they are doing, and we try to replicate to the other facilities that are not doing as well." (District Health Officer)*

For peer review only

Critical success factors for high routine immunization performance – Standards for Reporting Qualitative Research (SRQR) checklist

http://www.equator-network.org/reporting-guidelines/srqr/

Page/line no(s).

Title and abstract

**Title** - Critical success factors for high routine immunization performance: Qualitative analysis of interviews and focus groups from Nepal, Senegal, and Zambia	Page 1; 1-2
**Abstract** -<br>INTRODUCTION: Vaccination averts an estimated 2-3 million deaths annually. Although vaccine coverage improvements across Africa and South Asia have remained relatively stagnant and below global targets, several countries have outperformed their peers with significant increases in routine immunization coverage. Examining these countries' vaccination programs provides an opportunity to identify and describe critical success factors that may have supported these improvements.<br><br>METHODS: We selected three high-performing countries in regard to childhood vaccination: Nepal, Senegal, and Zambia. This multiple case study analysis was conducted using data from the Exemplars in Vaccine Delivery project within the Exemplars in Global Health program. We used qualitative analysis to investigate factors that contributed to high vaccination coverage through 66 key informant interviews (KIIs) and 22 focus group discussions (FGDs) at the national, regional, district, health facility, and community levels – with a total of 678 participants. Qualitative data were collected from October 2019 to April 2021. We triangulated these findings with quantitative analyses using publicly available data, which are published elsewhere.<br><br>RESULTS: Our data revealed that the critical success factors for vaccine programming relied on the cultural, historical, and statutory context in which the interventions were delivered. In Nepal, Senegal, and Zambia, high immunization coverage was driven by 1) strong governance structures and healthy policy environments; 2) adjacent successes in health systems strengthening; 3) government-led community engagement initiatives; and 4) adaptation considering contextual factors at all levels of the health system.<br><br>CONCLUSION: Throughout the study, our analysis returned to the importance of defining and understanding the context, governance, financing, and health systems within a country, rather than focusing on any one intervention. This paper augments findings from existing literature by highlighting how contextual factors impact implementation decisions that have led to improvements in childhood vaccine delivery. Findings from this research may identify transferable lessons and support actionable recommendations to improve national immunization coverage in other settings.	Page 1; 23 -51

Introduction

**Problem formulation** –<br>DESCRIPTION AND SIGNIFICANCE OF THE PROBLEM/PHENOMENON STUDIED:<br>Lines 109 – 110; "Vaccination is recognized as one of the most influential public health interventions, averting an estimated 2-3 million deaths annually [1-4]."<br>Lines 122 – 125; "Within both [WHO] regions, several countries outperformed their peers with significant increases in routine immunization coverage since 2000 [10]. Examining these countries' vaccination programs provides an opportunity to identify and describe critical success factors that may have supported these improvements."<br><br>REVIEW OF RELEVANT THEORY AND EMPIRICAL WORK:<br>Lines 127 – 131; "The essential components of an effective vaccine delivery system are well-established and include strong governance and leadership, healthcare financing, human resources, a robust supply chain, community engagement, and information systems for evidence-based decision-making [11-13]. Research on behaviors related to routine	Page 3

For peer review only - http://bmjopen.bmj.com/site/about/guidelines.xhtml

immunization focuses on recognized determinants of coverage, including intent to vaccinate, community access, and health facility readiness [14]."<br><br>PROBLEM STATEMENT:<br>Lines 131 – 134; "Although existing literature describes vaccine delivery systems, there is a gap in knowledge related to how programs and policies are implemented, how strategies are operationalized, and how context influences programming."	
**Purpose or research question –**<br>"The purpose of this study was to identify critical success factors that contributed to catalytic growth in childhood routine immunization coverage. Findings from this research may identify transferable lessons and support actionable recommendations to improve national immunization coverage in other settings [20]."	Page 3; 145 - 148

Methods

**Qualitative approach and research paradigm –**<br>APPROACH -<br>"We used qualitative analysis to investigate factors that contributed to high vaccination coverage through key informant interviews (KIIs) and focus group discussions (FGDs) at the national, regional, district, health facility, and community levels. We triangulated these findings with quantitative analyses using publicly available data, which are published elsewhere and referenced throughout this paper [10, 21].<br><br>Prior to data collection, we developed a conceptual model (Figure 1) to organize factors that impact childhood vaccine coverage globally. This model was based on the work of Phillips et al. and LaFond et al. alongside a broader review of the vaccine literature [14, 22]. Details regarding the development of this model are available elsewhere [10]."<br><br>PARADIGM –<br>"The interview guides were informed by the Consolidated Framework for Implementation Research (CFIR) [25] and the Context and Implementation of Complex Interventions (CICI) framework [26]."	Page 4; 175 – 17 183<br>Page 5; 203-205
**Researcher characteristics and reflexivity –**<br>Reflexivity statement provided as an appendix.	Appendix 3
**Context –**<br>"Qualitative data were collected between October 2019 and April 2021 at the national, regional, district, health facility, and community levels in Nepal, Senegal, and Zambia (Table 1)."<br><br>*Case studies in Nepal, Senegal, and Zambia are cited in text and provide additional details on context in each country.	Page 4; 202 - 203<br><br>Page 6; 251 - 254
**Sampling strategy –**<br>"Our sampling approach aimed to include a diverse sample of participants regarding geographic location and demographic qualities. Caregivers and volunteer community health workers were recruited for FGDs from health facility catchment areas with the assistance of local health staff."	Page 5; 217 - 220
**Ethical issues pertaining to human subjects –**<br>See section 2.4 "Ethical approval" and "Ethics approval statement".	Page 7; 271 – 279<br><br>Page 15; 618 - 625
**Data collection methods -**<br>"Qualitative data were collected between October 2019 and April 2021 at the national, regional, district, health facility, and community levels in Nepal, Senegal, and Zambia (Table 1). The interview guides were informed by the Consolidated Framework for Implementation Research (CFIR) [25] and the Context and Implementation of Complex Interventions (CICI) framework [26]. KII and FGD guides were translated into local languages by research assistants from the	Page 4 – 5; 202 - 223

For peer review only - http://bmjopen.bmj.com/site/about/guidelines.xhtml

respective countries. All interview guides were piloted before use and adjusted iteratively throughout data collection. For each country, an initial list of KIIs was developed with local research partners and MoH officials with snowball sampling used to identify additional key informants. Our sampling approach aimed to include a diverse sample of participants regarding geographic location and demographic qualities. Caregivers and volunteer community health workers were recruited for FGDs from health facility catchment areas with the assistance of local health staff. The duration of KIIs and FGDs averaged one and a half hours. KIIs and FGDs were audio-recorded with the permission of participants. Research files, recordings, and transcriptions were de-identified and password protected. Data analysis processes were generally consistent in all three countries examined in order to allow for thoughtful comparison."	
**Data collection instruments and technologies -** See above quote.	Page 4- 5; 202 - 223
**Units of study** – See Table 1.	Page 6; 251 - 254
**Data processing** – See section 2.2 "Qualitative data collection and analysis": due to the nature of the multiple case study, aspects of this are addressed throughout this part of the text.	Page 4- 5; 202 – 242
**Data analysis** – See section 2.2 "Qualitative data collection and analysis": due to the nature of the multiple case study, aspects of this are addressed throughout this part of the text.	Page 4- 5; 202 – 242
**Techniques to enhance trustworthiness** – "We applied a theory-informed thematic analysis of the transcripts to identify critical success factors. Data analysis was initially conducted individually for each case study, with separate members of our research team leading analysis for each country. However, to ensure consistency and coherence, all data analysts were involved in the review, coding, and summarization of data for all three case studies. We met frequently as a team to discuss overlap and to compare empirical findings. Transcripts were coded and analyzed using MaxQDA2020 software (Berlin, Germany). Coding was completed by researchers at Emory, some of whom also developed and revised the codebook throughout. Coding meetings were held frequently to discuss code definitions and to discuss emerging themes. Researchers from in-country teams were involved through additional meetings to discuss data and ground-truth findings. We considered setting and participant roles while identifying key points and further contextualized data using historical documents and an a priori literature review. Qualitative data collection and analysis tools, including topic guides and codebooks, can be found on our Open Sciences Framework (OSF) page [27]."<br><br>"Upon completion of each country case study, findings were disseminated to in-country experts and country governments, along with the TAG, allowing for the study team to ground-truth results. The research team at Emory University co-hosted regular meetings with the in-country research partners, experts in a variety of relevant fields, government officials, and other advisors to ask for feedback and contextualization. Researchers would present on methods, findings, and implications following an engaging discussion with advisors that would lead to improvements in our data analysis processes or presentation of findings. Revisions would be distributed through follow-up email conversations and subsequent phone calls with select advisors."	Page 5; 225 – 236<br><br>Page 7; 296 - 303

Results/findings

**Synthesis and interpretation** – See section 3.1 "Drivers of successful vaccination programs in exemplar countries" in addition to Figure 2 and Table 2, and Table 3.	Page 8; 326 - 339<br><br>Page 8 341<br><br>Page 8- 9; 344 – 348<br><br>Page 12; 481 - 485

For peer review only - http://bmjopen.bmj.com/site/about/guidelines.xhtml

**Links to empirical data** – See quotes as included in-text in addition to quotes by country and domain in Appendix 2. Citations included where reports or other documents were utilized as part of the analysis.	Embedded<br><br>Appendix 2

Discussion

**Integration with prior work, implications, transferability, and contribution(s) to the field** – See section 4, Discussion, starting on page 13.	Page 13 – 14; 489 - 556
**Limitations** – See section 5, Limitations, on page 14.	Page 14; 561 - 568

Other

**Conflicts of interest** – "The authors declare that they have no known competing financial interests or personal relationships that could have appeared to influence the research reported in this paper."	Page 15; 606 - 607
**Funding** – "This work was supported by the Bill & Melinda Gates Foundation, Seattle, WA (OPP1195041) with a planning grant from Gates Ventures, LLC, Kirkland, WA.:	Page 15; 602 - 603

*The authors created the SRQR by searching the literature to identify guidelines, reporting standards, and critical appraisal criteria for qualitative research; reviewing the reference lists of retrieved sources; and contacting experts to gain feedback. The SRQR aims to improve the transparency of all aspects of qualitative research by providing clear standards for reporting qualitative research.

**The rationale should briefly discuss the justification for choosing that theory, approach, method, or technique rather than other options available, the assumptions and limitations implicit in those choices, and how those choices influence study conclusions and transferability. As appropriate, the rationale for several items might be discussed together.

Reference:

O'Brien BC, Harris IB, Beckman TJ, Reed DA, Cook DA. **Standards for reporting qualitative research: a synthesis of recommendations.** *Academic Medicine*, Vol. 89, No. 9 / Sept 2014
DOI: 10.1097/ACM.0000000000000388

For peer review only
